# Synthesis and Anticancer Activity of New Quinazolin-4(3*H*)-one Derivatives: Identification of a Tumor-Selective Anticancer Agent with Potential Inhibition of TGF-βRI (ALK5)

**DOI:** 10.3390/ph19070996

**Published:** 2026-06-26

**Authors:** Nahed N. E. El-Sayed, Sami A. Al-Hussain, Marwa A. Ibrahim, Mohamed R. Elnagar, Zainab M. Almarhoon, Magdi E. A. Zaki

**Affiliations:** 1 Egyptian Drug Authority (EDA), 51 Wezaret El-Zeraa St., Giza 35521, Egypt; 2 Chemistry Department, Faculty of Science, Imam Mohammad Ibn Saud Islamic University (IMSIU), Riyadh 11623, Saudi Arabia; mezaki@imamu.edu.sa; 3 Department of Pharmaceutical Chemistry, Faculty of Pharmacy, Cairo University, Cairo 11562, Egypt; marwa.abdelaziz@pharma.cu.edu.eg; 4 Department of Pharmacology and Toxicology, Faculty of Pharmacy, Al-Azhar University, Cairo 011823, Egypt; mohamed.r.elnagar@azhar.edu.eg; 5 Department of Pharmacology, College of Pharmacy, The Islamic University, Najaf 54001, Iraq; 6 Department of Chemistry, College of Sciences, King Saud University, P.O. Box 2455, Riyadh 11451, Saudi Arabia; zalmarhoon@ksu.edu.sa

**Keywords:** hallmarks of cancer, multitarget-directed therapy, quinazolin-4(3*H*)-one, apoptosis, cell cycle, transforming growth factor-beta (TGF-β) type I receptor, ADME

## Abstract

**Background/Objectives**: Cancer is a multifactorial disease in which drug resistance and limited selectivity remain major therapeutic challenges, highlighting the need for novel anticancer agents. As a privileged scaffold for multitarget anticancer drug discovery, quinazolin-4(3*H*)-one was selected for the design, synthesis, and evaluation of new derivatives as potential anticancer agents, together with investigation of their mechanisms of action and molecular targets. **Methods**: Fifteen new quinazolin-4(3*H*)-one derivatives were synthesized and screened using the NCI-60 human cancer cell line panel. The mechanism of action of the most active compound was investigated through cell cycle, apoptosis, and RT-qPCR analyses. A potential molecular target was identified from transcriptomic data in the Human Protein Atlas, focusing on highly expressed cancer-implicated genes in the most responsive cell lines, followed by molecular docking, molecular dynamics simulations, and in vitro kinase studies. Safety and pharmacokinetic properties were evaluated using an MTT cytotoxicity assay in normal WI-38 fibroblasts and in silico ADME analyses. **Results:** Compound **3e** emerged as the most active and tumor-selective derivative, exhibiting GI_50_ values ranging from 2.63 to 17.12 µM across 31 cancer cell lines. In A549 cells, selected as a representative responsive model, **3e** (GI_50_ = 10.8 µM, 72 h) induced G2/M cell-cycle arrest (59.58% vs. 26.96% in control), increased early apoptosis (43.94% vs. 0.11% in control), reduced viable cells (49.71% vs. 98.66%), elevated the *Bax/Bcl-2* ratio (7.91), and upregulated the expression of *caspase-9* and *caspase-3* by 2.5- and 4.6-fold, respectively. Integrated target identification studies and an in vitro kinase assay (IC_50_ = 21.34 nM) suggested TGF-βRI (ALK5) as a plausible molecular target. Compound **3e** also showed low cytotoxicity toward WI-38 fibroblasts (IC_50_ = 88.3 µM) and favorable predicted pharmacokinetic properties; nevertheless, high plasma protein binding and potential CYP2C9 inhibition are anticipated. **Conclusions:** Compound **3e** is a promising tumor-selective anticancer lead with potential TGF-βRI inhibitory activity. Its antiproliferative effects in A549 cells appear to be mediated through G2/M cell-cycle arrest and activation of the intrinsic apoptotic pathway, supporting further development and pharmacokinetic optimization of this scaffold for anticancer therapy.

## 1. Introduction

Cancer remains one of the leading causes of mortality and represents a socioeconomic burden worldwide in the twenty-first century. According to the World Health Organization, the global incidence of cancer is projected to exceed 35 million new cases by 2050, representing an approximately 77% increase compared with 2022 estimates [1]. Despite substantial advances in cancer therapy, conventional treatment modalities—including surgery, radiotherapy, and chemotherapy—remain central to cancer management. However, these approaches continue to suffer from major limitations, including poor selectivity, severe systemic toxicity, tumor heterogeneity, multidrug resistance, and limited efficacy against advanced or metastatic disease [2]. Although immunotherapy has transformed the therapeutic landscape of several malignancies, its clinical success is still hindered by immune-related adverse events, resistance development, and variable patient responsiveness [3], underscoring the need for novel therapeutic agents. In this context, understanding the multifaceted biology of cancer is essential, as tumor development and progression are governed by interconnected signaling pathways that cannot be effectively addressed through single-target modulation.

**Figure 1:**
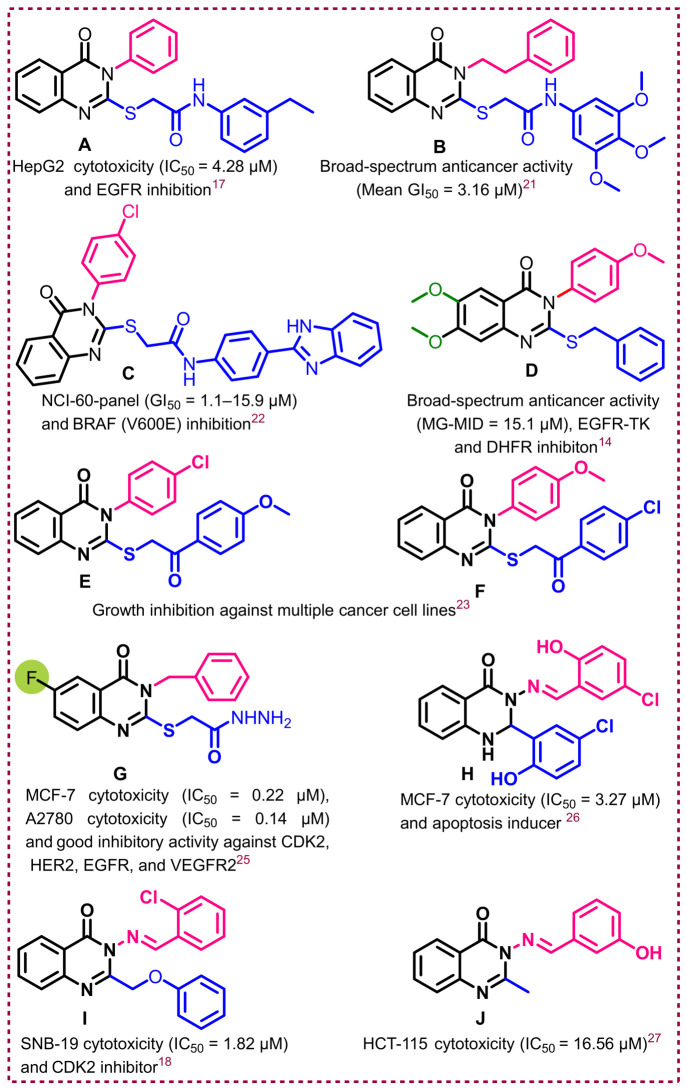
Chemical structures of reported quinazolin-4(3*H*)-one derivatives with anticancer activity and the key molecular targets implicated in their mechanisms of action.

Cancer is a complex and heterogeneous disease driven by genetic and epigenetic alterations [4], which lead to deregulation of key cellular functions known as the hallmarks of cancer, including sustained proliferative signaling, evasion of growth suppressors, resistance to cell death, metabolic reprogramming, angiogenesis, and invasion and metastasis [5,6,7]. These processes are orchestrated by interconnected pathways such as MAPK, PI3K/AKT, NF-κB, JAK/STAT, Wnt/β-catenin, TGF-β, and Notch [8], whose crosstalk contributes to tumor heterogeneity, progression, and therapeutic resistance. Consequently, multitarget-directed therapeutic strategies have emerged as a promising alternative to the one-target-one-drug paradigm in cancer research [9,10,11]. Therefore, approved and investigational anticancer agents were analyzed to identify scaffolds suitable for such approaches. These analyses revealed quinazoline and its analogues as privileged scaffolds in anticancer drug discovery. In particular, the quinazolin-4(3*H*)-one scaffold has attracted considerable interest owing to its ability to modulate multiple molecular targets involved in tumor growth and survival [12,13]. Its planar aromatic framework enables π–π stacking interactions with molecular targets, including EGFR-TK [14] and DHFR [15], while the ring heteroatoms facilitate hydrogen bonding within kinase ATP-binding sites. These interactions have been demonstrated in several cancer-related kinases, including EGFR [16,17], CDK2 [18], and VEGFR-2, BRAF_wt_, and BRAF_V600E_ [19], resulting in inhibition of phosphorylation processes essential for tumor growth. Moreover, quinazolin-4(3*H*)-one derivatives have been explored as DNA-intercalating agents that interfere with DNA replication and cell proliferation [20]. Furthermore, structural optimization of the quinazolin-4(3*H*)-one scaffold through incorporation of diverse pharmacophoric elements has generated numerous anticancer leads (Figure 1). For example, incorporation of 2-thio-substituted anilide functionalities significantly improved antiproliferative activity of compounds **A** (IC_50_ = 4.28 µM) and **B** (GI_50_ = 3.16 µM), compared with doxorubicin (32.02 µM) and 5-fluorouracil (22.60 µM) [17,21].

Likewise, the 2-anilidobenzimidazole–quinazolin-4(3*H*)-one hybrid **C** displayed superior activity against the NCI-60 cancer cell panel (GI_50_ = 1.1–15.9 µM), compared with related 2-anilidobenzimidazole-thiopyrimidine or pyrimidinone conjugates, highlighting the significant contribution of the quinazolin-4(3*H*)-one core to anticancer efficacy [22]. Introduction of a 2-thiobenzyl substituent rather than a 2-thiomethyl, as in compound **D**, enhanced broad-spectrum antitumor activity [mean-graph midpoint (MG-MID) of 15.1 µM] together with induction of G1/S arrest and apoptosis [14]. In addition, compounds **E** and **F**, bearing 3-substituted phenyl and 2-thiophenacyl moieties with mixed electron-donating and electron-withdrawing groups, demonstrated promising antitumor activity in vitro [23].

Fluorine substitution has been widely utilized in anticancer drug design to improve pharmacokinetic (PK) properties [24]. Notably, fluorine-substituted derivatives such as **G** exhibited strong cytotoxicity against MCF-7 (IC_50_ = 0.22 µM) and A2780 (IC_50_ = 0.14 µM) cells associated with potent inhibitory activity against EGFR, HER2, VEGFR2, and CDK2 [25].

Quinazolin-4(3*H*)-one Schiff bases have also shown promising anticancer activity; for example, compound **H** selectively inhibited MCF-7 breast cancer cell viability (IC_50_ = 3.27 µM) through ROS generation, mitochondrial dysfunction, cytochrome c release, caspase activation, Bid modulation, and NF-κB suppression [26]. Compound **I** exhibited potent cytotoxicity against SNB-19 CNS cancer cells (IC_50_ = 1.82 µM) along with CDK2 inhibitory activity and induction of G1-phase arrest [18], whereas compound **J** demonstrated cytotoxicity against HCT-115 cells (IC_50_ = 16.56 µM) [27].

The formamidine moiety (R^1^N=CH–NR^2^R^3^) has attracted considerable attention in medicinal chemistry as a bioisostere for hydrolytically labile amide bonds [28] and as valuable pharmacophoric motifs in anticancer drug design [29]. In addition, the presence of a tertiary amino group can impart favorable physicochemical properties, including increased polarity, ionizability, and aqueous solubility, which may contribute to improved pharmacokinetic behavior [30]. 

Furthermore, the carbamate moiety (–NHCOO–) is widely used in anticancer prodrugs and FDA-approved therapies, including mitomycin C, docetaxel, colchicine, and irinotecan [31,32].

### Rational Design

**Figure 2:**
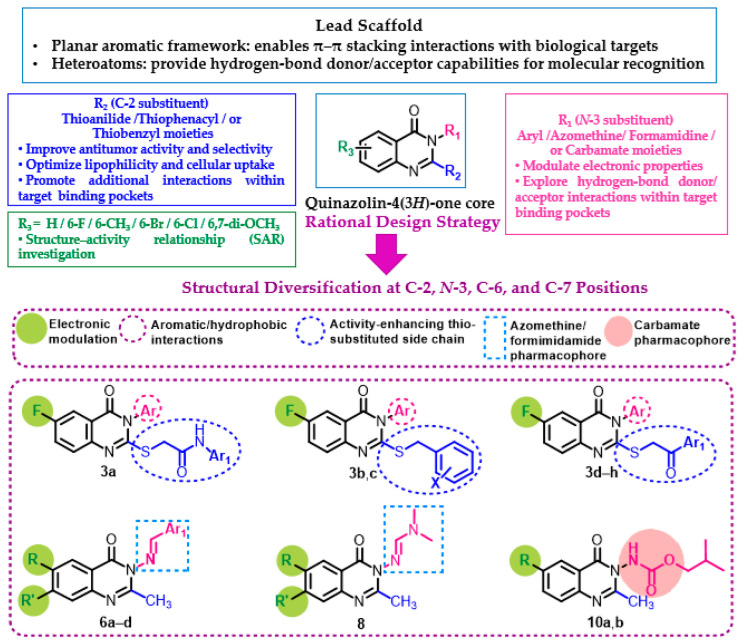
Rational design strategy of the target new quinazolin-4(3*H*)-one derivatives.

The design strategy was guided by a hypothesis-driven multitarget anticancer approach, taking into consideration the multifactorial nature of cancer and the limitations of single-target therapies. Accordingly, two complementary design principles were adopted to guide the development of potential multitarget-directed anticancer agents, as illustrated in Figure 2. The first entailed the selection of the quinazolin-4(3*H*)-one scaffold as the central framework owing to its established anticancer activity and its ability to engage in π–π stacking and hydrogen-bonding interactions with multiple biological targets implicated in tumorigenesis. The second involved systematic structural diversification at positions 2, 3, 6, and 7 through the introduction of various substituents and pharmacophoric elements, guided by the structural features of previously reported anticancer quinazolin-4(3*H*)-one derivatives (**A**–**J**, Figure 1) and key structural motifs found in approved drugs and prodrugs, with the aim of generating derivatives with improved multitargeted anticancer activity and drug-like properties. On this basis, compounds **3a**–**h** were designed to incorporate a 6-fluoro substituent to potentially enhance PK properties, together with aryl substitution at C-3 to strengthen hydrophobic interactions. Importantly, sulfur-containing functionalities at C-2, including thioanilide, thiobenzyl, and thiophenacyl moieties, were introduced because of their reported association with enhanced anticancer activity. The anilide and phenacyl fragments were proposed to provide additional hydrogen-bonding capacity and polarity, whereas the benzyl moiety was designed to enhance hydrophobic interactions within target binding pockets. Notably, Schiff base derivatives **6a**–**d** and their analog *N*,*N*-dimethylformimidamide derivative **8** were designed by introducing an azomethine functionality at position 3 bearing either a substituted phenyl group or a polar and ionizable tertiary amino group [–N(CH_3_)_2_], respectively, to evaluate their influence on anticancer potency and selectivity. Finally, carbamate-containing derivatives **10a**,**b** were designed to improve physicochemical and PK properties, particularly aqueous solubility, membrane permeability, and metabolic stability. Throughout the series, both electron-donating (hydroxy, methyl, and methoxy) and electron-withdrawing (fluoro, chloro, bromo, iodo, cyano, and carboxyl) substituents were incorporated to explore structure–activity relationships and assess the influence of electronic and steric factors on anticancer activity.

Based on these considerations, the designed target quinazolin-4(3*H*)-one derivatives were synthesized, characterized, and evaluated for their in vitro antitumor activity through the NCI-60 screening program. Following initial single-dose screening, the most active compound was further subjected to five-dose testing to determine its antiproliferative potency across the full panel. To investigate its mechanism of action, apoptosis and cell-cycle analyses were performed on a representative sensitive cell line. Potential molecular targets were then explored using transcriptomic data obtained from the Human Protein Atlas (https://www.proteinatlas.org, accessed on 25 April 2025), focusing on highly expressed cancer-implicated genes in the most responsive cell lines. The prioritized target was subsequently subjected to molecular docking and molecular dynamics simulations to assess binding interactions and stability of the proposed target–ligand complex. Finally, an in vitro kinase inhibition assay was conducted to experimentally validate the plausible molecular target. In addition, the selectivity profile against normal WI-38 fibroblasts was evaluated to determine the safety and therapeutic potential of that active candidate, while ADME profile was predicted to assess its pharmacokinetic properties and drug-likeness.

## 2. Results and Discussion

### 2.1. Synthesis and Structural Characterization

**Scheme 1:**
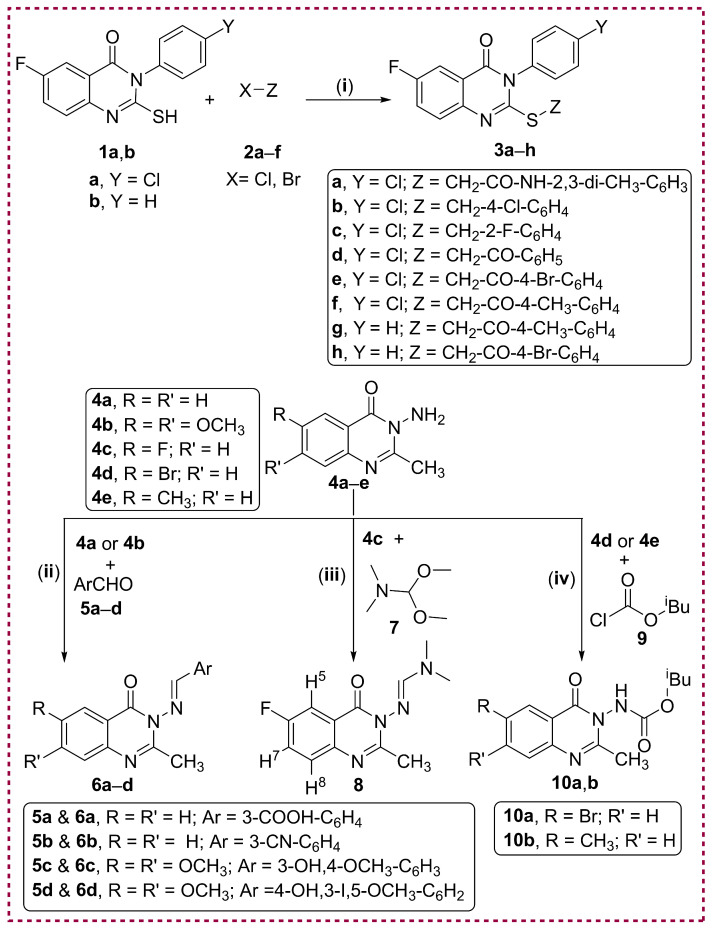
Reagents and conditions: (**i**) dry acetone, K2CO3, reflux 12 h; (**ii**) absolute ethanol, catalytic amount of glacial acetic acid, reflux 10 h; (**iii**) absolute ethanol, reflux 5 h; (**iv**) reflux 10 h.

Fifteen new quinazolin-4(3*H*)-one derivatives were synthesized as outlined in Scheme 1, and their structures were fully characterized uisng IR, ^1^H NMR, ^13^C NMR, and MS analyses.

The starting 6-fluoro-2-mercapto-3-arylquinazolin-4(3*H*)-ones **1a**,**b** were prepared following the reported procedure [33]. Subsequent arylation of compounds **1a** and **1b** was achieved by refluxing with various halo compounds—namely 2-chloro-*N*-(2,3-dimethylphenyl)acetamide (**2a**) [34], *p*-chlorobenzyl chloride (**2b**), 2-fluorobenzyl bromide (**2c**), and phenacyl bromides (**2d**–**f**)—in dry acetone and in the presence of an excess of activated K_2_CO_3_, affording the corresponding sulfanyl quinazolinones **3a**–**h**.

The IR (KBr) spectra confirmed the expected transformations, as evidenced by the disappearance of the NH absorption bands at ν = 3236 and 3228 cm^−1^ (observed in starting materials **1a**,**b**). Compound **3a** exhibited new stretching bands at ν 3272 and 1696 cm^−1^, attributable to the NH and C=O groups of the acetamide fragment, respectively. Compounds **3d**–**h** displayed strong absorptions at ν 1689, 1688, 1691, 1685, and 1690 cm^−1^, characteristic of the carbonyl groups of the phenacyl moieties. The ^1^H NMR spectrum (300 MHz; DMSO-*d*_6_) of **3a** revealed three diagnostic singlets at *δ*_H_ 4.11, 2.24, and 2.06 ppm corresponding to a methylene and two methyl groups, along with a downfield signal at *δ*_H_ 9.74 ppm attributable to the proton of the *sec*-amino group in the HN-(2,3-dimethylphenyl)acetamide moiety. The ^13^C NMR spectrum supported this assignment, as indicated by the disappearance of the thioxo carbon resonance of **1a** (*δ*_C_ 175.9 ppm) and emergence of new signals at *δ*_C_ 161.1 (HN–CO), 36.9 (methylene), 20.5 and 14.5 ppm (two methyl carbons). For derivatives **3b** and **3c**, the benzylic methylene groups appeared at *δ*_H_ 4.37 (*δ*_C_ 35.7 ppm) and 4.42 (*δ*_C_ 30.1 ppm), respectively.

Compounds **3d**–**h** showed characteristic signals of the methylene protons at *δ*_H_ 4.63, 4.52, 4.73, 4.71, and 4.46 ppm, with corresponding carbon resonances at *δ*_C_ 39.1, 39.5, 39.5, 39.6, and 39.3 ppm, respectively. In addition, ketonic carbonyl signals were observed at *δ*_C_ 193.2, 192.5, 192.9, 192.9, and 192.6 ppm, respectively. All other aliphatic and aromatic resonances were consistent with the proposed structures.

Notably, the carbon spectra of these fluorinated analogs exhibited the distinctive ^13^C–^19^F coupling signals. For example, compound **3c**, bearing two fluorine atoms, exhibited two doublet signals in the ^13^C NMR spectrum (150 MHz, CDCl_3_) at *δ*_C_ 161.1 and 160.2 ppm, with ^1^*J*_C–F_ coupling constants of 247.2 and 245.0 Hz, respectively, attributable to the two *ipso* carbons. Similarly, the carbon atoms at the *ortho* and *meta*-positions showed the characteristic ^13^C–^19^F coupling signals at *δ*_C_ 129.4 (d, ^3^*J*_C–F_ = 8.1 Hz, *meta*-CH), 128.5 (d, ^3^*J*_C–F_ = 6.9 Hz, *meta*-CH), 123.1 (d, ^2^*J*_C–F_ = 24.2 Hz, 2 × *ortho*-CH), 121.0 (d, ^3^*J*_C–F_ = 8.1 Hz, *meta*-CH), 115.4 (d, ^2^*J*_C–F_ = 21.9 Hz, *ortho*-CH), and 112.0 (d, ^2^*J*_C–F_ = 23.0 Hz, *ortho*-CH).

In a separate sequence, condensation of 3-amino-2-methyl-3*H*-quinazolin-4-one **4a** and 6,7-dimethoxy-3-amino-2-methyl-3*H*-quinazolin-4-one **4b** with aryl aldehydes **5a**–**d** in boiling absolute ethanol containing a catalytic amount of glacial acetic acid afforded the corresponding Schiff bases **6a**–**d**. Their IR spectra confirmed successful transformations, as evidenced by the disappearance of the amino absorption bands at ν (cm^−1^) 3540, 3302 (for **4a**) and 3303, 3202 (for **4b**). New characteristic absorptions were observed at ν 1721 (carboxylic-C=O, **6a**), 2231 (C≡N, **6b**), and a broad band at 3408 (OH, **6c**). The azomethine (HC=N) absorption overlapped with the imine (C=N of the quinazolinone core) absorption and appeared at ν 1598 (**6a**), 1604 (**6b**), 1611 (**6c**), and 1608 (**6d**) cm^−1^. The ^1^H NMR spectra further supported the formation of the Schiff bases, showing the disappearance of the amino proton signal in **4a** (*δ*_H_ 5.82 ppm) and **4b** (*δ*_H_ 5.76 ppm), and the emergence of azomethine singlets at *δ*_H_ 9.14, 9.29, 8.81, and 8.68 ppm, respectively. Phenolic OH resonances were observed at *δ*_H_ 2.24 (**6c**) and 10.49 ppm (**6d**). In the ^13^C NMR spectra, azomethine carbons appeared at *δ*_C_ 153.8, 154.1, 154.9, and 155.0 ppm, respectively, along with diagnostic signals for carboxyl carbon at *δ*_C_ 169.1 ppm (**6a**), nitrile carbon at *δ*_C_ 117.9 ppm (**6b**), and quaternary iodine-bearing carbon at *δ*_C_ 84.8 ppm (**6d**).

Next, compound **4c** (6-fluoro-3-amino-2-methyl-3*H*-quinazolin-4-one) was transformed into the corresponding *N*,*N*-dimethylformimidamide derivative **8** by refluxing with excess DMF-DMA **7**. The IR spectrum of compound **8** showed the disappearance of the primary amino absorption bands of **4c** at 3301 and 3187 cm^−1^ coupled with the appearance of a new absorption band for the imine (HC=N) group at ν 1587 cm^−1^. In the ^1^H NMR spectrum (300 MHz, CDCl_3_), the methyl group attached to the quinazolinone core resonated at *δ*_H_ 2.49 ppm. The aromatic protons exhibited characteristic fluorine couplings. In particular, the proton at C-7 of the quinazolinone ring, resonating at *δ*_H_ 7.36 ppm, appeared as a triplet of doublets due to coupling with the *ortho*-fluorine atom at C-6 and the adjacent proton at C-8, with identical coupling constants (^3^*J*_H_^7^_-F_ = ^3^*J*_H_^7^_-H_^8^ = 8.7 Hz), together with a long-range coupling to the proton at C-5 (^4^*J*_H_^7^_-H_^5^ = 3.0 Hz). The proton at *δ*_H_ 7.78 ppm, assigned to H-5, displayed a doublet of doublets (^3^*J*_H_^5^_-F_ = 8.6 Hz, ^4^*J*_H_^5^_-H_^7^ = 3.2 Hz), whereas H-8 at *δ*_H_ 7.56 ppm appeared as a doublet of doublets (^3^*J*_H_^8^_-H_^7^ = 9.0 Hz, ^4^*J*_H_^8^_-F_ = 5.1 Hz). The *N*,*N*-dimethylformimidamide moiety [–N═CH–N(CH_3_)_2_] showed an azomethine proton signal at *δ*_H_ 7.71 ppm, while the two methyl groups appeared as two distinct singlets of equal intensity at *δ*_H_ 3.04 and 3.09 ppm. The ^13^C NMR spectrum (75 MHz, CDCl_3_) further confirmed the structure and exhibited extensive ^13^C–^19^F coupling. The *ipso*-C-F (C-6) exhibited a doublet at *δ*_C_ 159.7 (^1^*J*_C–F_ = 244.4 Hz), while the aromatic methine carbons at *ortho*- and *meta*-positions showed three doublets at *δ*_C_ 128.5 (d, ^3^*J*_C–F_ = 8.0 Hz, *meta*-CH), 121.7 (d, ^2^*J*_C–F_ = 24.1 Hz, *ortho*-CH), and 110.7 (d, ^2^*J*_C–F_ = 23.5 Hz, *ortho*-CH). The lactam C═O, C_q_═N, CH═N, C_q8a_, and C_q4a_ carbons were detected at *δ*_C_ 161.6, 159.1, 154.0, 142.9, and 122.0 ppm, respectively. The NMe_2_ carbons were nonequivalent, resonating at *δ*_C_ 34.2 and 40.6 ppm, whereas the quinazolinone methyl carbon appeared at *δ*_C_ 22.2 ppm.

Finally, condensation of 6-bromo-3-amino-2-methyl-3*H*-quinazolin-4-one **4d** and 6-methyl-3-amino-2-methyl-3*H*-quinazolin-4-one **4e** with excess isobutyl chloroformate afforded the corresponding carbamates **10a** and **10b**. Their IR spectra showed the disappearance of the NH_2_ absorption bands of the starting materials at ν 3284, 3112 cm^−1^ (**4d**) and 3419, 3314 cm^−1^ (**4e**), accompanied by the appearance of new bands at ν 3250 and 3259 cm^−1^ (*sec*-amide NH), along with absorptions at ν 1751 and 1790 cm^−1^ (C=O), respectively.

In the ^1^H NMR spectra, the primary amino signals of **4d** (*δ*_H_ 4.89 ppm) and **4e** (*δ*_H_ 5.65 ppm) disappeared, while new *sec*-amide NH resonances were observed at *δ*_H_ 10.63 (**10a**) and 10.40 ppm (**10b**). The isobutyl group [–CH_2_–CH(CH_3_)_2_] displayed three characteristic signals. For **10a**, the ^1^H NMR spectrum (300 MHz, DMSO-*d*_6_) showed signals at *δ*_H_ 3.96 (2H, d, *J* = 6.0 Hz, CH_2_), 1.95 (1H, apparent quintet, *J* = 6.6 Hz, CH), and 0.92 (6H, d, *J* = 6.6 Hz, 2 × CH_3_), with corresponding ^13^C NMR (75 MHz, DMSO-*d*_6_) signals at *δ_C_* 71.5 (CH_2_), 27.7 (CH), and 18.7 (2 × CH_3_). For **10b**, the ^1^H NMR spectrum (500 MHz, DMSO-*d*_6_) displayed signals at *δ*_H_ 3.99 (2H, d, *J* = 6.5 Hz, CH_2_), 1.79 (1H, apparent septet, *J* = 6.5 Hz, CH), and 0.72 (6H, d, *J* = 7.0 Hz, 2 × CH_3_), with corresponding ^13^C NMR (125 MHz, DMSO-*d*_6_) resonances at *δ_C_* 74.0 (CH_2_), 27.5 (CH), and 18.6 (2 × CH_3_).

The mass spectra of all newly synthesized compounds exhibited the expected molecular ion peaks.

The spectral data for selected representative compounds are provided in the Supplementary Materials. Notably, any signals observed in the ^1^H NMR spectra recorded in CDCl_3_, particularly in the 1.0–2.2 ppm region, are attributable to trace residual solvents present in the deuterated solvent and should not be interpreted as impurities of the synthesized compounds.

The stereochemical configuration of the azomethine groups in Schiff base derivatives **6a**–**d** was assigned the *E*-configuration based on the observed chemical shifts (*δ*_H_ 8.68–9.29 ppm), which are consistent with the reported values for -CH═N- protons in *E*-isomers (*δ*_H_ 8.62–9.51 ppm) [35]. Furthermore, energy minimization calculations (Table S1, Supplementary Materials) indicated that *E*-isomers possess lower total energy than their *Z*-counterparts, attributable to reduced steric strain and enhanced resonance stabilization from planarity, thereby establishing the *E*-form as the thermodynamically preferred geometry.

**Figure 3:**
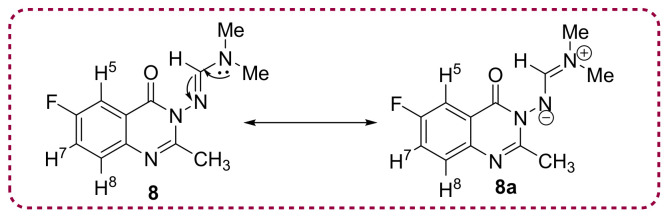
The restricted rotation about the NMe_2_–C bond in the *E*-isomer of formamidine **8**.

Similarly, the stereochemical configuration of *N*,*N*-dimethylformimidamide derivative **8** was assigned as *E* based on multiple lines of evidence: (**i**) literature reports indicate that *Z*-formamidines are thermodynamically unstable and undergo complete isomerization to the corresponding *E*-isomers upon heating in inert solvents [36]; (**ii**) the sterically congested *Z*-configuration of amidines typically displays a single broad NMe_2_ signal over a wide temperature range (34 to −80 °C) due to free rotation about the NMe_2_–C bond, whereas compound **8** exhibited two distinct NMe_2_ resonances in both ^1^H and ^13^C NMR spectra, indicating restricted rotation about the NMe_2_–C bond, which may be attributed to contributions of resonance structure such as **8a** (Figure 3) [36]; and (**iii**) energy minimization calculations demonstrated that the *E*-isomer is more stable, possessing lower total energy than the *Z*-isomer (Table S1, Supplementary Materials). Together, these results establish that compound **8** preferentially adopts the thermodynamically favored *E*-configuration.

### 2.2. Anticancer Evaluation

To evaluate the anticancer potential of the synthesized quinazolin-4(3*H*)-one derivatives, a comprehensive strategy integrating in vitro screening, structure–activity relationship (SAR) analysis, cellular mechanistic investigations, and molecular target identification with subsequent experimental validation was employed.

#### 2.2.1. In Vitro Anticancer Screening

##### One-Dose Screening Against the NCI-60 Human Tumor Cell Line Panel

The starting materials **1a**,**b**, along with compounds **3a**–**h**; **6a**–**d**; **8**; and **10a**,**b** were submitted to the NCI-60 screening program for preliminary anticancer evaluation at a single high dose (10 µM) across the full panel of 60 human tumor cell lines, representing nine cancer types (leukemia, non-small cell lung (NSCLC), colon, CNS, melanoma, ovarian, renal, prostate, and breast).

The primary results are presented as one-dose mean graphs showing growth percent (G%), which represents the percentage of cell growth relative to both untreated controls and the cell number at the time of treatment (time zero), thereby allowing the detection of both growth inhibition (100 > G% > 0) and lethality (0 > G%; i.e., negative values). From these screening data, three additional parameters were analyzed: (i) mean growth percent, representing the mean growth perecent across all 60 cell lines relative to untreated controls; (ii) delta, the maximum deviation of an individual cell line from the mean, reflecting the degree of response heterogeneity; a high delta value indicates pronounced differences in sensitivity among cell lines, whereas a low delta value corresponds to more homogenous responses; and (iii) range, the difference between the most resistant (highest G%) and most sensitive cell lines (lowest G%), reflecting the degree of tumor-type selectivity, with large range values indicating highly variable responses across tumor types and, therefore, greater selectivity, whereas moderate or small ranges imply similar behavior across tumors (i.e., low selectivity) [37]. These metrics were used to compare the anticancer profiles of the seventeen tested compounds and to identify the most sensitive and resistant cell lines, as well as those exhibiting cytotoxic responses (G% < 0) and cytostatic responses (0 < G% ≤ 10 and 10 < G% ≤ 50) following treatment with each test compound (Table S2).

Compounds **1a**, **1b**, **3h**, **6a**, **6b**, **6c**, and **10a** exhibited the weakest activity in the series, showing G% values > 50% across all cell lines, with even their most responsive cell lines displaying G% values ranging from 58.88 to 85.69. This poor activity is further supported by their high mean growth values (93.06–106.82%) and relatively low delta (21.13–39.89) and range (38.75–69.36) values, consistent with uniform inactivity and weak selectivity (Table S2).

Compounds **3a**–**3d**, **3g**, **6d**, **8**, and **10b** also exhibited high mean G% values ranging from 62.58 to 104.28, indicating overall weak growth inhibition with some instances of slight growth promotion (G% > 100) at the tested concentration, as further supported by the G% values of their resistant cell lines (103.58–140.11). Their relatively high delta values (42.60–84.14) reveal pronounced variability in the response of individual cell lines, reflecting substantial heterogeneity in antiproliferative effects. This observation is further supported by the large range values (77.72–130.01), which demonstrate marked differences between the most sensitive and most resistant cell lines, indicating that some cell lines showed measurable inhibition while others remained resistant or even proliferative (Table S2). Indeed, measurable antiproliferative effects (G% < 50) across multiple cell lines were observed, as summarized in Table 1: compounds **3a** (HOP-62: 26.46, HOP-92: 26.92, NCI-H226: 21.49, HCT-116: 44.79, SF-539: 25.74, SNB-19: 7.05, SNB-75: −20.71, U251: 48.82, OVCAR-8: 32.45, 786-0: 34.72, ACHN: 37.79, CAKI-1: −21.56, RXF 393: 18.82, UO-31: 39.65, HS 578T: 25.70, and BT-549: 31.98); **3b** (HOP-92: 44.61, NCI-H226: 37.97, and HS 578T: 47.99); **3c** (SNB-19: 33.27, SNB-75: 21.47, ACHN: 47.53, and HS 578T: 34.47); **3d** (SNB-19: 45.71, SNB-75: 41.65, 786-0: 48.97, and HS 578T: 40.73); **3g** (SNB-75: 45.03 and HS 578T: 45.61); **6d** (CCRF-CEM: 49.53, RPMI-8226: 24.73, SR: 40.49, LOX IMVI: 48.44, and SN12C: 42.91); **8** (HCC-2998: 38.61); and **10b** (CCRF-CEM: 45.99, HL-60(TB): 43.76, K-562: 34.25, and RPMI-8226: 33.56), indicating partial growth inhibition.

Notably, compounds **3e** and **3f** displayed the lowest mean growth percentages (33.47, 48.84, respectively) compared to the other derivatives. They also exhibited high delta values (82.86 and 70.73) and wide ranges (144.86 and 139.39), indicative of heterogeneity and pronounced tumor-type selectivity, with CNS, renal cancer, and lung cancers being the most common sensitive tumor panels. 

**Figure A1:**
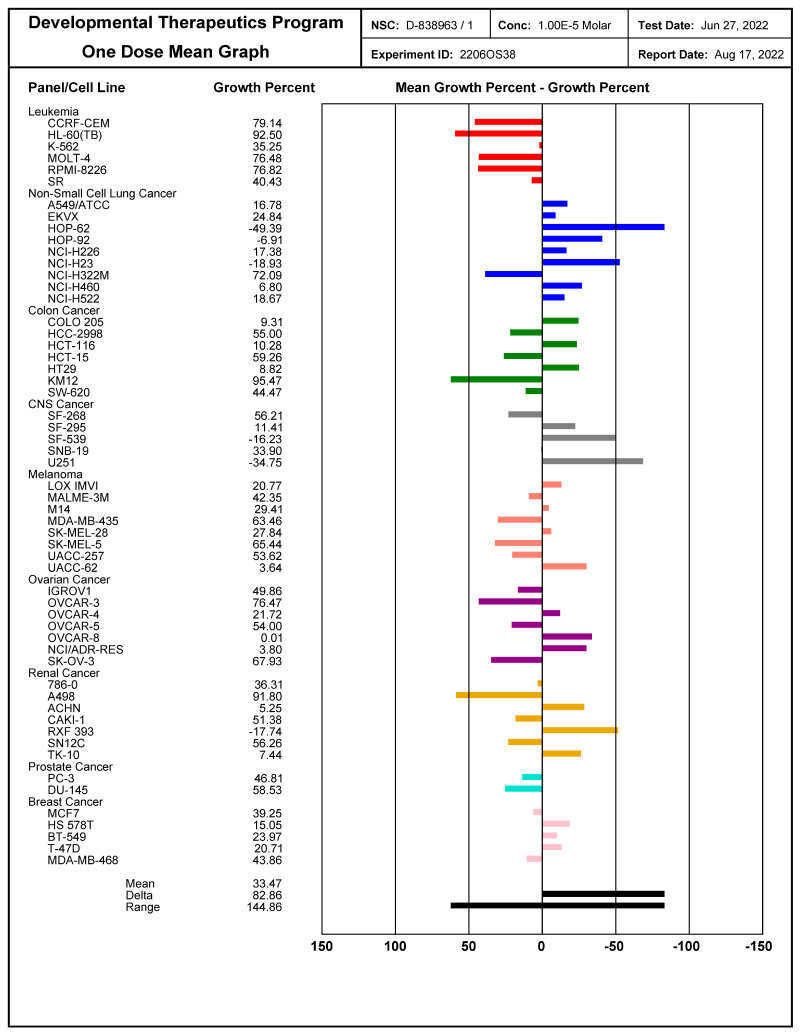
One-dose mean graph of compound **3e**.

Compound **3e** (Appendix A, Figure A1) exhibited strong cytotoxic effects (G% < 0) against six cell lines (HOP-62: −49.39, HOP-92: −6.91, NCI-H23: −18.93, SF-539: −16.23, U251: −34.75, and RXF 393: −17.74). Meanwhile, it displayed potent cytostatic (0 < G% < 10) effects against eight cell lines (NCI-H460: 6.80; COLO 205: 9.31; HT29: 8.82; UACC-62: 3.64; OVCAR-8: 0.01; NCI/ADR-RES: 3.80; ACHN: 5.25; and TK-10: 7.44). Collectively, these results suggest a tumor-selective spectrum of antitumor activity, with preferential sensitivity in lung (4 cell lines), colon (3 cell lines), and renal (3 cell lines) cancers. Other sensitive cell lines (10% < G% < 50) include K-562: 35.25; SR: 40.43; A549/ATCC: 16.78; EKVX: 24.84; NCI-H226: 17.38; NCI-H522: 18.67; HCT-116: 10.28; SW-620: 44.47; SF-295: 11.41; SNB-19: 33.90; LOX IMVI: 20.77; MALME-3M: 42.35; M14: 29.41; SK-MEL-28: 27.84; IGROV1: 49.86; OVCAR-4: 21.72; 786-0: 36.31; PC-3: 46.81; MCF7: 39.25; HS 578T: 15.05; BT-549: 23.97; T-47D: 20.71; and MDA-MB-468: 43.86, as summarized in Table 1 and Table S2.

Similarly, compound **3f** demonstrated notable selectivity against non-small cell lung carcinoma cells, with G% values < 0 against HOP-62: −17.14; HOP-92: −2.96, and NCI-H226: −21.89, as well as sensitivity in CNS (SNB-75: −19.41), renal (RXF 393: −10.65), and breast (HS 578T: −18.89) cancers, along with G% < 10 responses against SF-295: 4.69; SF-539: 2.03; and BT-549: 1.44 cell lines. 

In addition, **3f** demonstrated 10% < G% < 50 responses against NCI-H23: 46.04; NCI-H460: 22.42; HCT-116: 24.43; HT29: 49.94; SF-268: 28.90; U251: 19.60; MALME-3M: 39.64; OVCAR-4: 30.97; OVCAR-8: 37.76; NCI/ADR-RES: 28.27; SK-OV-3: 45.95; 786-0: 10.73; ACHN: 33.34; CAKI-1: 10.14; PC-3: 25.92; MDA-MB-231/ATCC: 13.66; and MDA-MB-468: 44.71 (Table 1 and Table S2).

##### Structure-Activity Relationship (SAR) Analysis

**Figure 4:**
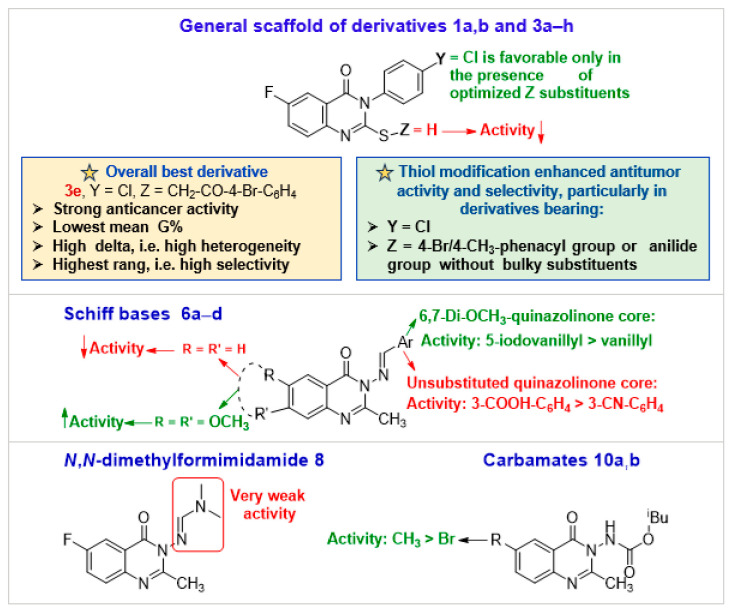
Summary of the key SAR findings for the synthesized quinazolin-4(3*H*)-one derivatives.

The one-dose screening data enabled SAR analysis across the synthesized scaffolds, revealing that anticancer activity is strongly influenced by the nature and position of substituents on the quinazolinone core, as illustrated in Figure 4.

First, the 3-(4-substituted/unsubstituted aryl)-6-fluoro-2-mercaptoquinazolin-4(3*H*)-one precursors **1a**,**b** (particularly **1b**) were the least active members, as evidenced by their high mean G% values. They also exhibited relatively low delta and range values compared to their corresponding S-substituted derivatives **3a**–**3h**, reflecting weak and uniform activity across the cell panel. This observation suggests that the modification of the mercaptan group (–SH) generally enhances anticancer activity.

Within the **3a**–**3h** series, featuring variation at the Y position (Cl or H) and the Z fragment (acetanilide, benzyl or 4-unsubstituted/4-substituted phenacyl; Scheme 1), structural comparison highlights several determinants of potency and selectivity as follows:

- Compared to the precursor **1a**, incorporation of a 4-substituted phenacyl moiety at Z, as exemplified by **3e** (4-bromophenacyl) and **3f** (4-methylphenacyl), resulted in greater anticancer activity (lower mean G%) than the bulky *N*-(2,3-dimethylphenyl)acetamide substituent in compound **3a**. Moreover, these phenacyl-containing derivatives exhibited comparatively lower response heterogeneity (lower delta) along with enhanced tumor-type selectivity (higher range values), indicating selective activity toward specific cell lines.
- Other derivatives, such as **3b** and **3c** (lacking the carbonyl functionality in Z), **3d** (unsubstituted phenacyl at Z), and **3g** and **3h** (lacking chlorine at Y) showed reduced activity (higher G%) along with lower delta and narrower range values. These findings confirm that the absence of key structural features—namely, the benzoyl carbonyl group and halogen substitution—diminishes both potency and the extent of response heterogeneity as well as selectivity.

Taken together, these results indicate that chlorine substitution at Y combined with a 4-substituted phenacyl group at Z (particularly with halogens or mildly electron-donating substituents), while avoiding excessive steric bulk, enhances both antiproliferative activity and tumor-type selectivity. These features therefore represent favorable design elements for further optimization.

The Schiff base derivatives **6a**–**d** displayed overall weak cytotoxicity, accompanied by limited response variability and selectivity. However, compounds incorporating a 6,7-dimethoxy-2-methylquinazolin-4(3*H*)-one core (**6c** and **6d**) demonstrated improved activity compared to their unsubstituted counterparts (**6a** and **6b**). Among them, **6d** (Ar = 5-iodovanillyl group) exhibited superior potency (lower mean G%) relative to **6c** (Ar = vanillyl group), along with higher delta and broader range values. This profile indicates increased response heterogeneity and enhanced tumor-type selectivity, suggesting that **6d** exerts more pronounced effects in specific cell lines rather than uniform activity. This behavior may be attributed to the increased steric bulk and polarizability of iodine, potentially enhancing target interactions.

The *N*,*N*-dimethylformimidamide derivative **8**, featuring a 6-fluoro-2-methylquinazolin-4(3*H*)-one core and a formamidine moiety composed of an azomethine and a tertiary amine functionality, showed very weak activity. Although associated with elevated delta and range values, this pattern likely reflects inconsistent and generally weak effects rather than meaningful tumor-type selectivity.

In the carbamate derivatives **10a**,**b**, although **10b** (R = Me) remained weakly active, it exhibited improved antiproliferative activity compared to its bromo-substituted analog **10a**. Additionally, **10b** showed higher delta and range values, indicating increased response heterogeneity and a more differentiated activity profile across the cell line panel.

These findings suggest that incorporation of a smaller alkyl substituent promotes modest improvements in activity while also increasing variability in cell line responses and selectivity toward specific cell types.

Overall, the one-dose SAR analysis (Figure 4) indicates that among the quinazolin-4(3*H*)-one scaffolds, the 6-fluoro-2-((2-oxo-2-arylethyl)thio)-3-arylquinazolin-4(3*H*)-one emerged as the most promising framework. Within this scaffold, compound **3e** exhibited the strongest anticancer activity. Its high delta value indicates substantial variability in response across different cancer cell lines, implying pronounced response heterogeneity and the potential for selective effectiveness in specific tumor types. Moreover, the high range value reflects a marked difference between the most sensitive and most resistant cell lines, further supporting strong tumor-type selectivity. Accordingly, these features suggest that compound **3e** is not only highly potent but also exhibits a selective anticancer activity profile, making it a promising candidate for targeting specific tumor subtypes rather than acting as a non-selective agent.

These findings suggest that fine-tuning the balance between electronic effects and steric demand at the R_1_ and R_2_ positions in the quinazolin-4(3*H*)-one scaffold (Figure 2) represents a rational strategy for designing next-generation derivatives with enhanced potency and selectivity.

##### NCI-60 Five-Dose Screening Evaluation

Quinazolin-4(3*H*)-one **3e**, which demonstrated significant growth inhibition in the one-dose screen and satisfied the NCI threshold criterion (at least 8 cell lines exhibiting G% values below 10), was advanced to the standard five-dose evaluation across the 60-cell line panel. The compound was tested at five concentrations spanning a log_10_ scale from −4 to −8 M (10^−4^–10^−8^ M; 100 to 0.01 µM).

**Figure A2:**
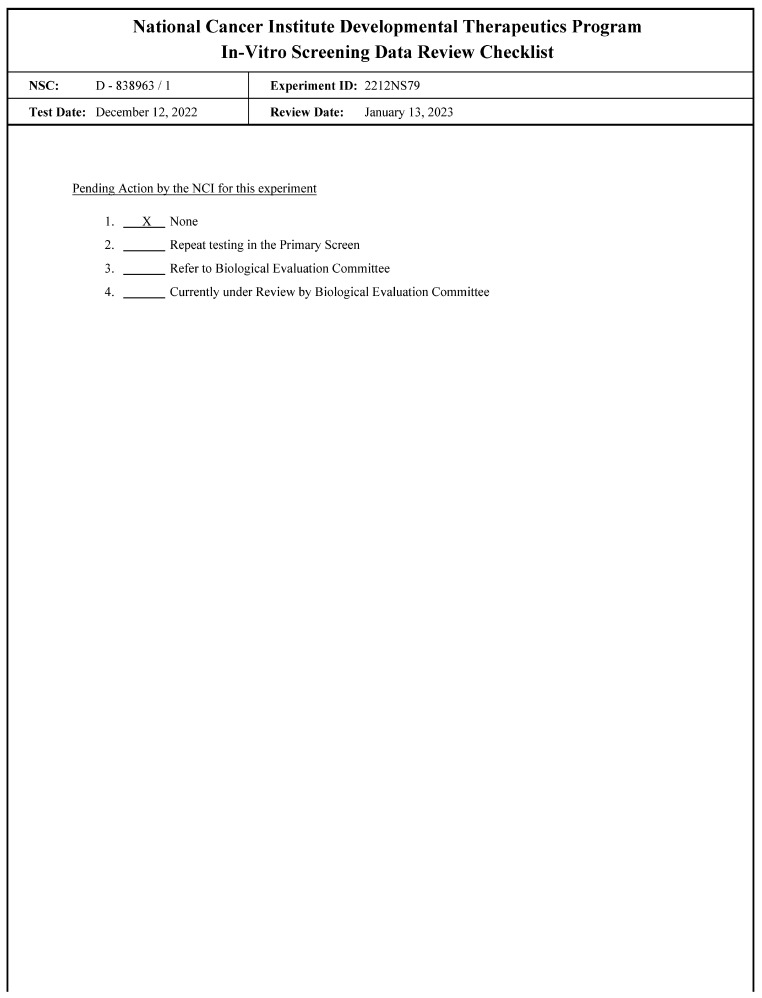
Dose–response curves across nine tumor subpanels.

**Figure A3:**
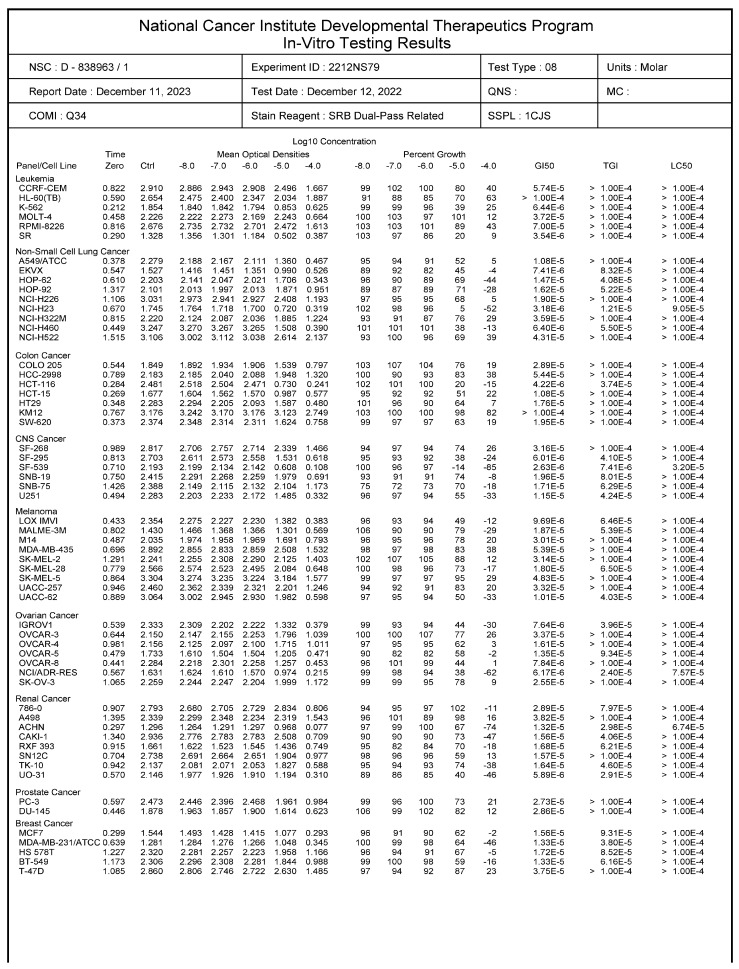
Mean graphs for GI_50_, TGI, and LC_50_ values across the NCI-60 cancer cell line panel.

**Figure A4:**
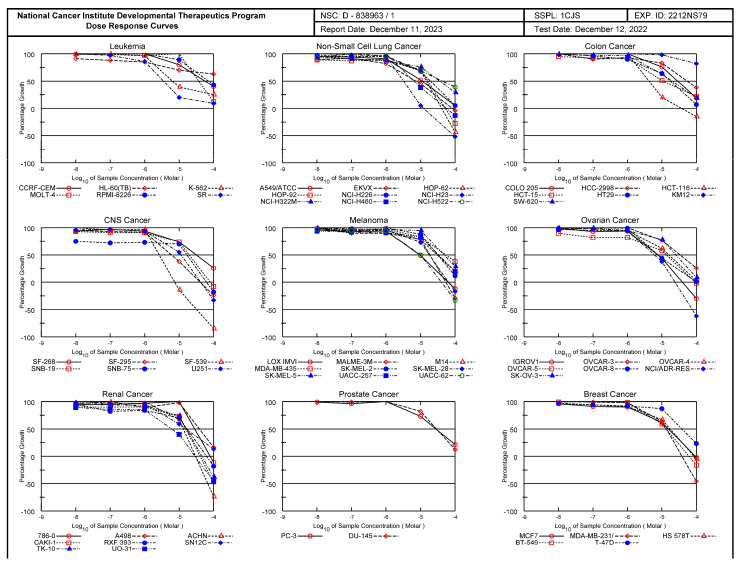
Composite representation of dose–response curves across the NCI-60 cancer cell line panel.

**Figure A5:**
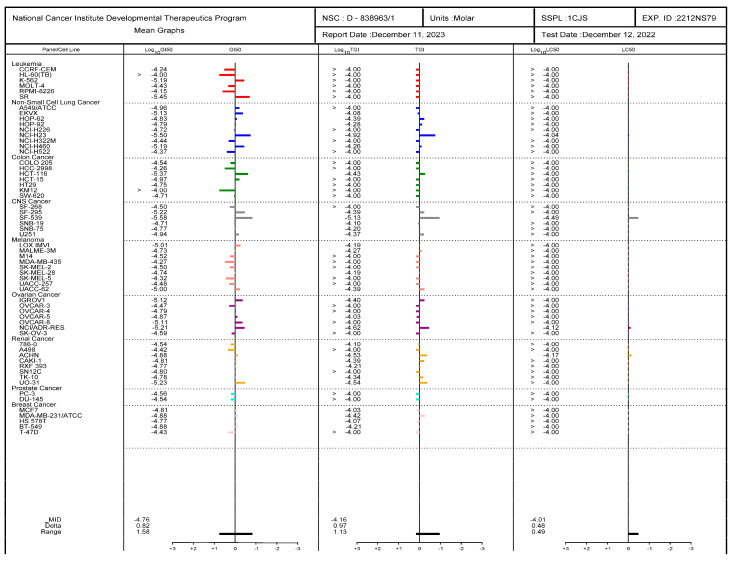
Waterfall graph for GI_50_.

**Figure A6:**
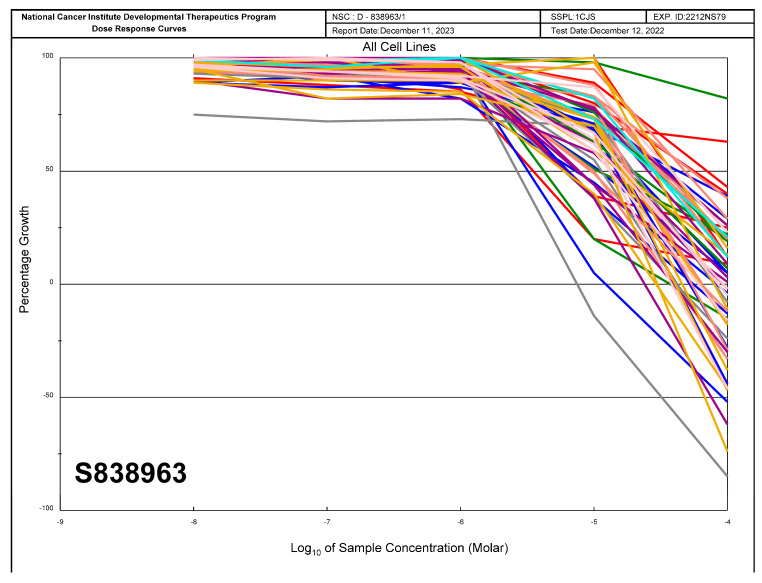
Waterfall graph for TGI.

**Figure A7:**
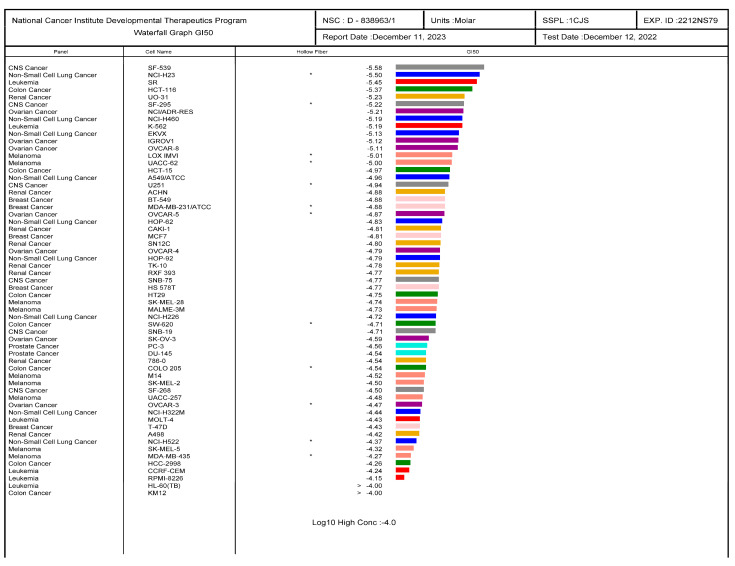
Waterfall graph for LC_50_.

The complete NCI five-dose screening data package for compound **3e** is provided in Appendix B (8 pages) and comprises five components (Table A1 and Figure A2, Figure A3, Figure A4, Figure A5, Figure A6 and Figure A7). 

The first component (Table A1, page 2) presents the primary data sheet containing the raw experimental values for each cell line, including: (i) the average of 24 optical density (OD) measurements of SRB-derived color at time zero (prior to drug exposure), (ii) the average of 4 OD measurements of untreated control cells after 48 h (Ctrl), and (iii) the average of 2 OD measurements after 48 h of exposure to **3e** at each of the five tested concentrations (expressed on a log_10_ scale). From these data, the percentage growth (PG) values at each dose were calculated, and three standard response parameters were derived: GI_50_ (concentration causing a 50% reduction in net protein during the drug incubation relative to controls), TGI (the drug concentration resulting in total growth inhibition, i.e., cytostasis), and LC_50_ (concentration causing a 50% reduction in the measured protein at the end of the drug treatment as compared to that at the beginning, indicating cytotoxicity). When activity thresholds were not reached, results were reported as greater or less than the highest or lowest concentration tested.

Compound **3e** exhibited growth inhibitory activity ranging from strong to weak across the NCI-60 panel, with GI_50_ values spanning 2.63 to >100 µM. The median potency, expressed as the full-panel mean graph midpoint (MG-MID; Figure A3, page 4), was log_10_GI_50_ = −4.76 M, corresponding to 17.38 µM, indicating moderate overall antiproliferative potency. This value was used as an empirical reference threshold for relative sensitivity. Notably, 31 cell lines showed enhanced sensitivity with GI_50_ < 17.38 µM, as summarized in Table 2. These include SR and K-562 (leukemia); A549/ATCC, EKVX, HOP-62, HOP-92, NCI-H23, and NCI-H460 (NSCLC); HCT-116 and HCT-15 (colon); SF-295, SF-539, SNB-75, and U251 (CNS); LOX IMVI and UACC-62 (melanoma); IGROV1, OVCAR-4, OVCAR-5, OVCAR-8, and NCI/ADR-RES (ovarian); ACHN, CAKI-1, RXF-393, SN12C, TK-10, and UO-31 (renal); and MCF7, MDA-MB-231/ATCC, HS-578T, and BT-549 (breast).

Moreover, six cell lines (NCI-H226, HT29, SW-620, SNB-19, MALME-3M, and SK-MEL-28) exhibited GI_50_ values of 19.0, 17.6, 19.5, 19.6, 18.7, 18.0 µM, respectively. The remaining cell lines showed GI_50_ ranging from 25.5 µM (SK-OV-3) to >100 µM (KM12 and HL-60(TB)). The delta value of 0.82 log units, corresponding to approximately a 6.6-fold deviation from the mean GI_50_, reflects moderate variability in cellular response, supporting a degree of tumor-type selectivity. The range of 1.58 log units corresponds to GI_50_ values spanning from 2.63 µM (log_10_GI_50_ = −5.58; most sensitive) to >100 µM (log_10_GI_50_ > −4.0; most resistant), representing approximately a 38-fold variation in activity across the panel. This substantial spread highlights pronounced tumor-type selectivity, indicating preferential activity of compound **3e** toward specific cancer subtypes.

For complete growth arrest (TGI) and 50% lethality (LC_50_), higher concentrations were required, ranging from 24.1 to >100 µM and 32 to >100 µM, respectively, supporting a predominantly cytostatic profile.

The second component of the NCI report comprises dose–response curves (Figure A2, page 3), generated by plotting PG values against log_10_ concentration for each cell line and grouped into nine tumor subpanels.

The third component includes mean graphs for GI_50_, TGI, and LC_50_ (Figure A3, page 4), which enable visualization of selectivity patterns across tumor types. In these plots, bars extending to the right of the MID indicate greater sensitivity (lower log_10_GI_50_), whereas those extending to the left indicate reduced sensitivity. Because the axis is logarithmic, a shift of one unit corresponds to a 10-fold change in potency.

The fourth component comprises a composite representation of dose–response curves across the NCI-60 cancer cell line panel (Figure A4, page 5). 

The fifth component presents the waterfall graphs for GI_50_, TGI, and LC_50_ (Figure A5, Figure A6 and Figure A7, pages 6–8, respectively), in which individual cell lines are ranked in descending order of sensitivity for each response parameter, thereby enabling direct visualization of subpanel selectivity [37,38]. Accordingly, the most sensitive cell lines appear at the top of the respective waterfall plot.

#### 2.2.2. Anticancer Mechanism

Given the predicted limited blood–brain barrier (BBB) penetration of compound **3e** (see ADME characteristics, Section 2.2.4), the limited availability of additional cancer cell line panels exhibiting significant expression of transforming growth factor-β (TGF-β) type I receptor (see analysis of gene expression profiles of the most responsive cell lines below), and the high clinical relevance of lung cancer as the most prevalent (12.4% of all cancers globally) and deadliest malignancy worldwide (1.8 million deaths, 18.7%) [1], the A549 human lung adenocarcinoma cell line was employed as a representative responsive cell line to elucidate the antitumor mode of action of compound **3e** at both the cellular and molecular levels. Cellular investigations included cell cycle distribution analysis and assessment of apoptosis induction. Furthermore, the expression of apoptosis-regulating genes—namely *Bax*, *Bcl-2*, *caspase-9*, and *caspase-3*—was analyzed to characterize the apoptotic pathway involved. In addition, molecular studies aimed at target identification were conducted to identify the potential molecular target underlying the anticancer activity of compound **3e**. The predicted target was subsequently investigated and validated using computational and biochemical approaches.

##### Cell Cycle Analysis and Detection of Apoptosis

Cancer cells frequently exhibit dysregulated cell cycle control and apoptotic signaling due to accumulated genetic and epigenetic alterations, resulting in sustained proliferation, evasion of programmed cell death, and genomic instability [4]. Normal proliferating cells progress sequentially through four major phases of the cell cycle: G1 (gap 1), S (DNA synthesis), G2 (gap 2), and M (mitosis). These phases are tightly regulated by checkpoints, particularly at the G1/S and G2/M transitions, which coordinate DNA replication, repair, and mitosis to maintain genomic integrity. In response to cellular stress, these checkpoints can activate apoptotic pathways to eliminate damaged cells.

Apoptosis is mainly mediated through intrinsic mitochondrial and extrinsic death receptor pathways. The intrinsic pathway is governed by *BCL-2* family proteins, including anti-apoptotic proteins (*Bcl-2*, Bcl-xL, Mcl-1), pro-apoptotic effectors (*Bax*, Bak), and BH3-only proteins (Bid, Bad, PUMA, Noxa). The balance between pro-apoptotic and anti-apoptotic proteins determines cell fate, with *Bax* promoting mitochondrial outer membrane permeabilization and cytochrome c release, whereas *Bcl-2* suppresses this process. Cytochrome c release subsequently activates *caspase-9*, leading to *caspase-3*–mediated execution of apoptosis [39]. The extrinsic pathway is initiated through activation of death receptors such as Fas and TRAIL receptors, which trigger caspase-8 activation via death-inducing signaling complexes. Both apoptosis cascades converge on the activation of executioner caspases such as *caspase-3* and caspase-7, producing the characteristic biochemical and morphological features of apoptosis [39]. In cancer cells, these pathways are commonly dysregulated through overexpression of anti-apoptotic proteins, loss of pro-apoptotic mediators, defective caspase activation, impaired upstream regulation such as p53, or defects in death receptor signaling (DR4/DR5), thereby enabling malignant cells to evade apoptosis and resist therapy [39,40]. Therefore, inducing cell cycle arrest and restoring apoptotic signaling—by inhibiting anti-apoptotic proteins, activating pro-apoptotic effectors, or directly engaging caspase cascades—are considered key anticancer mechanisms [12,39,41].

**Figure 5:**
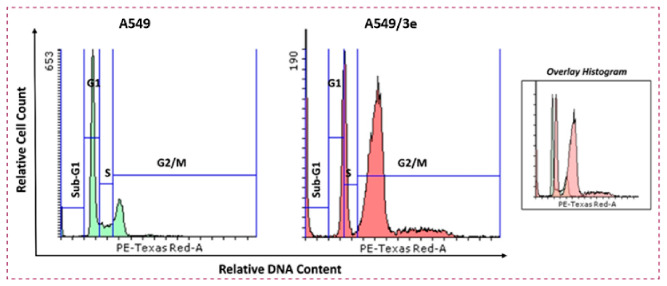
Flow cytometric analysis of A549 cells showing changes in cell cycle distribution after treatment with compound **3e** (10.8 µM, 72 h).

Accordingly, the effects of compound **3e** on these key anticancer mechanisms were subsequently investigated in A549 cells following 72 h of treatment at its IC_50_ concentration (10.8 µM). The results of flow cytometric analysis of cell-cycle distribution are presented as histograms (Figure 5) and summarized numerically in Table 3. Control cells exhibited a normal proliferative distribution, with a small apoptotic sub-G1 population (4.13%), the majority residing in the G1 phase (57.17%), and the remainder distributed across the S (11.74%) and G2/M (26.96%) phases. In contrast, treatment with compound **3e** markedly perturbed this profile. The sub-G1 fraction increased to 17.06%, consistent with DNA fragmentation and apoptosis induction. This was accompanied by a sharp reduction in the G1 population to 10.03%, indicating impaired early cell cycle progression, with a concomitant accumulation of cells in the later phases of the cell cycle. A modest rise in the S-phase fraction (13.06%) suggested interference with DNA synthesis. Most notably, cells accumulated in the G2/M phase (59.58%), demonstrating a pronounced G2/M checkpoint arrest, which may contribute to the induction of apoptosis. These results indicate that while A549 cells are capable of completing DNA replication, they fail to progress into mitosis.

Together, these findings suggest that compound **3e** exerts its anticancer activity in A549 cells by promoting apoptosis and reducing proliferative capacity through perturbation of cell cycle regulation. The observed depletion of the G1 population and enforcement of G2/M arrest are associated with impaired cell cycle progression and suppression of uncontrolled proliferation, in line with the inhibitory profile obtained in the one-dose assay.

**Figure 6:**
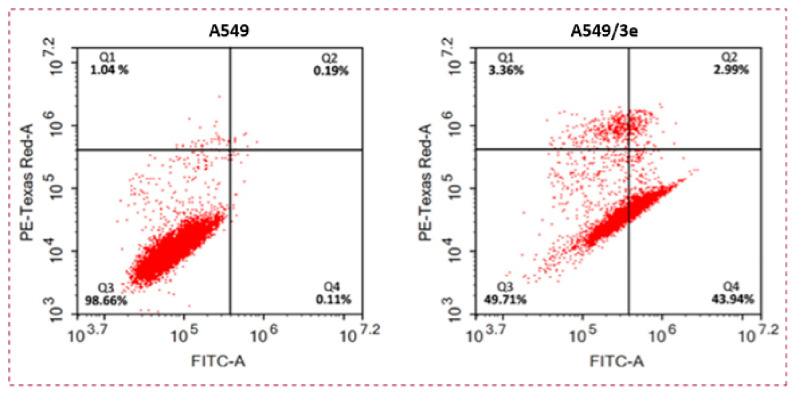
Flow cytometric analysis of apoptosis in A549 cells following treatment with compound **3e** (10.8 µM, 72 h), as determined by Annexin V-FITC/PI dual staining.

To further validate apoptosis induction, annexin V-FITC/PI double staining was performed and analyzed using flow cytometry. As shown in Figure 6 and Table 4, treatment of A549 cells with compound **3e** markedly reduced the viable population (from 98.66% in control to 49.71%), with the majority of cells undergoing early apoptosis (43.94% vs. 0.11% in control) and a smaller fraction progressing to late apoptosis (2.99% vs. 0.19% in control). Necrosis remained relatively low (3.36% vs. 1.04% in control). 

Collectively, the results of both analyses complement each other and indicate that treatment with compound **3e** primarily induces G2/M arrest, which is accompanied by apoptosis in A549 cells. 

##### Real-Time Quantitative Polymerase Chain Reaction (RT-qPCR) Analysis of Apoptosis-Mediating Genes

To elucidate the molecular mechanism underlying apoptosis, RT-qPCR was employed to assess the mRNA expression of four key apoptosis-related genes: the pro-apoptotic *Bax*, the anti-apoptotic *Bcl-2*, the initiator *caspase-9*, and the effector *caspase-3*.

**Figure 7:**
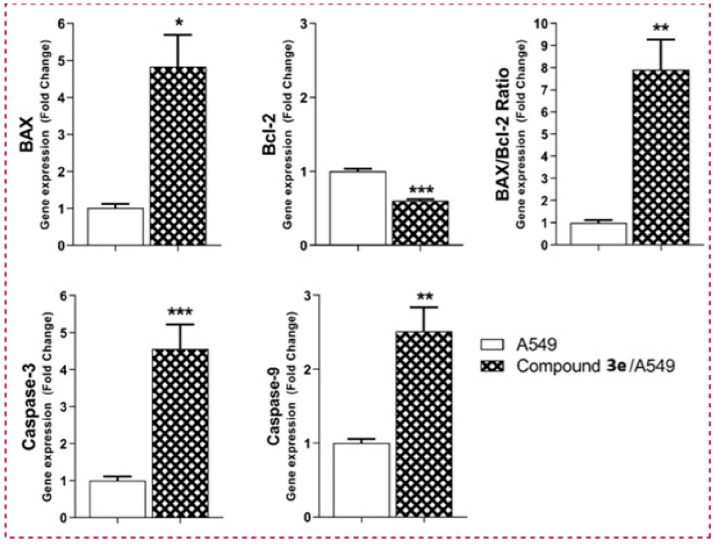
Gene expression analysis of *Bax, Bcl-2*, *Caspase–3*, and *Caspase–9* following treatment of A549 cells with compound **3e** (10.8 μM, 72 h). Data from three independent experiments are presented as a ratio of target gene/GAPDH expression (relative mRNA levels) and represent the mean ± SEM. Normalized data are expressed as fold changes, with control set to ‘1’. * *p* < 0.05, ** *p* < 0.01 and *** *p* < 0.001 indicate statistically significant differences from the corresponding control in unpaired *t*-tests.

As shown in Figure 7 and Table 5, treatment of A549 cells with compound **3e** at its IC_50_ (10.8 µM, 72 h) significantly altered gene expression. *Bax* mRNA increased by 4.83-fold, while *Bcl-2* expression decreased to 0.60-fold of the control, leading to a pronounced elevation of the *Bax*/*Bcl-2* ratio to 7.91. This modulation is consistent with activation of the intrinsic apoptotic pathway and further supports the anticancer potential of compound **3e**, based on previous reports linking a high *Bax*/*Bcl-2* ratio to enhanced chemosensitivity and improved therapeutic responses in both hematological and solid tumors [42,43]. In parallel, the transcriptional levels of *caspase-9* and *caspase-3* increased by 2.51- and 4.56-fold, respectively, further supporting involvement of the mitochondrial apoptotic cascade.

Together with the results of Annexin V/PI staining, these findings suggest that compound **3e** induces apoptosis in A549 cells predominantly through the intrinsic mitochondrial pathway. The observed modulation of the *Bax*/*Bcl-2* balance and activation of downstream caspase signaling contributes to suppression of tumor cell survival, providing a mechanistic basis for the cytotoxic effect in this cellular model and supporting its potential as a lead scaffold for further anticancer investigation.

A limitation of the present work is that cell cycle, apoptosis, and apoptosis-related gene expression analyses were performed only in A549 cells; therefore, validation in additional cancer cell lines is required to confirm the broader applicability of the present findings.

##### Molecular Target Identification for **3e**

Analysis of gene expression profiles of the most responsive cell lines

To gain insight into the potential molecular target(s) for the most active antiproliferative agent **3e**, transcriptomic data (Figure S1, Supplementary Materials) retrieved from the Human Protein Atlas (HPA) [44] were analyzed to profile gene expression in the most responsive 31 cell lines (GI_50_ values below the mean inhibitory dose of 17.38 µM). The analysis revealed that 20 of the 28 evaluable cell lines exhibited overexpression of the transforming growth factor-β type I receptor (TGF-βRI/ALK5), as indicated by positive Z-scores (Table 2). The renal (RXF 393 and SN12C) and ovarian (NCI/ADR-RES) cell lines were not included in the HPA database and therefore could not be evaluated. Nevertheless, an independent microarray-derived transcriptomic profiling study identified TGF-βRI as the most downregulated gene in the NCI/ADR-RES cell line [45]. Collectively, these findings suggest TGF-βRI as a plausible molecular target underlying the anticancer activity of compound **3e**.

**Figure 8:**
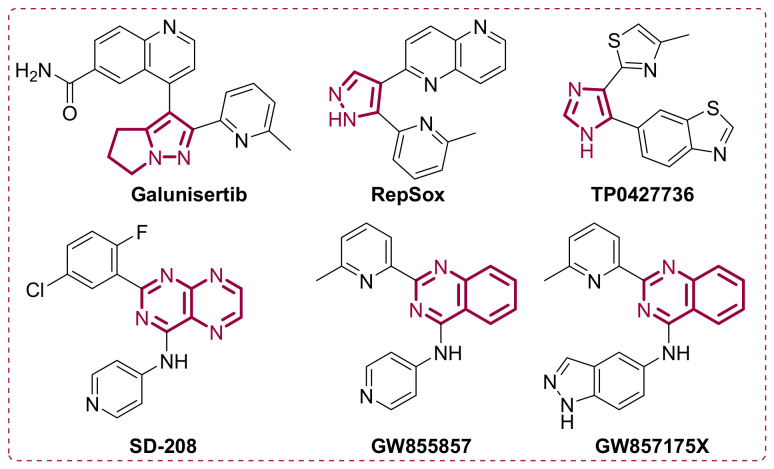
Reported ALK5/TGF-βRI inhibitors incorporating diverse heterocyclic core scaffolds.

The TGF-β signaling pathway regulates cell growth and differentiation, extracellular matrix production, cell motility, angiogenesis, cellular immunity, and apoptosis. Members of the TGF-β family exhibit dual physiological activity; in normal cells and in early stages of tumorigenesis, TGF-β functions as a tumor suppressor via inhibiting cell cycle progression and proliferation of normal and transformed cells by arresting them in the G1 phase [46]. However, in advanced malignancies, the sustained proliferating signaling in tumor cells is linked to sustained TGF-β signaling [7], which frequently promotes tumor progression by enhancing epithelial–mesenchymal transition (EMT), immune evasion, angiogenesis, and metastatic dissemination [47,48,49]. EMT, in particular, is a critical process associated with cytoskeletal remodeling, increased cell motility, invasion, resistance to apoptosis, and acquisition of stem cell–like properties. Among the three TGF-β isoforms, TGF-βI is the most abundant and plays a dominant role in regulating these processes. Consequently, targeting the TGF-β signaling pathway to block its oncogenic effects—particularly through inhibition of the TGF-βRI kinase—has emerged as a promising therapeutic strategy in cancer treatment [47,49,50]. These inhibitors primarily bind to the ATP-binding site of the TGF-βRI/ALK5 kinase domain, thereby preventing the phosphorylation of the intracellular signal transducers SMAD2/3 and disrupting downstream signaling cascades, such as the MAPK, PI3K/AKT, and Rho/ROCK pathways, as well as the expression of target genes involved in regulating cellular processes such as EMT, cell cycle progression, and apoptosis [49]. Several TGF-βRI inhibitors (Figure 8) currently in clinical trials or preclinical development feature diverse heterocyclic chemotypes (such as dihydropyrrolopyrazole, pyrazole, imidazole, pteridine, and quinazoline) as central cores [51,52].

The quinazoline-based inhibitors GW855857 and GW857175X have shown strong TGF-βRI inhibition, with IC_50_ values as low as 0.025 µM [53]. Structural analyses revealed that the pyridyl-*N* and indazolyl-*N*^2^ moieties form hydrogen bonds with the key hinge region residue His283, while the quinazoline-*N*^1^ engages in a hydrogen bond with the gatekeeper residue Lys232 [51].

Molecular docking simulations

Molecular docking simulations play a crucial role in target fishing by predicting the interactions of small molecules with potential protein-binding sites. By analyzing binding patterns and affinities, these simulations assist in identifying the most probable biological targets. This computational approach significantly enhances the efficiency of drug discovery by guiding experimental efforts toward the most promising protein targets [54].

In the current study, a molecular docking approach was used to guide the target fishing of compound **3e**.

**Figure 9:**
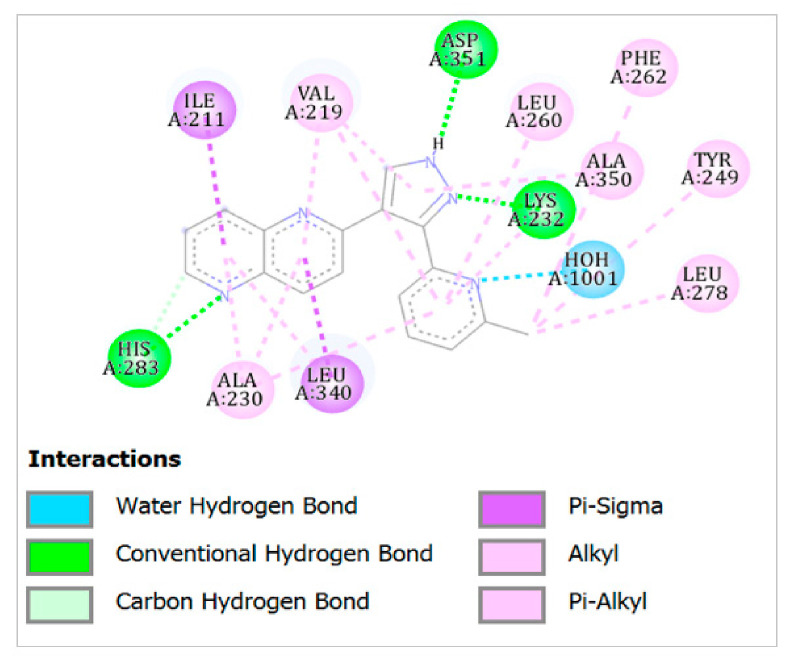
2D interaction diagram showing RepSox (E-616452) binding interactions with the key amino acids in the TGF-ꞵ type I active site.

The X-ray co–crystal structure of the pyrazole derivative E-616452 (RepSox) in complex with the TGF-βRI (PDB ID: 1VJY) was selected as the template for the molecular docking study. In this structure, the naphthyridine-*N*^5^ of the cognate ligand fits into the ATP-binding site and forms a hydrogen bond with the essential hinge-region residue His283. The *N*^1^ and *N*^2^ atoms of the pyrazole ring are involved in two additional hydrogen bonds with residues Lys232 and Asp351. Moreover, the pyridyl-*N*^1^ further engages in a hydrogen bond with a water molecule (W1001) [55], as illustrated in Figure 9 and Figure S2. Docking validation successfully reproduced these interactions, with a low RMSD of 0.323 Å and a docking score of −10.3 Kcal/mol (Table 6, Figure S2), confirming the reliability of the docking protocol.

**Figure 10:**
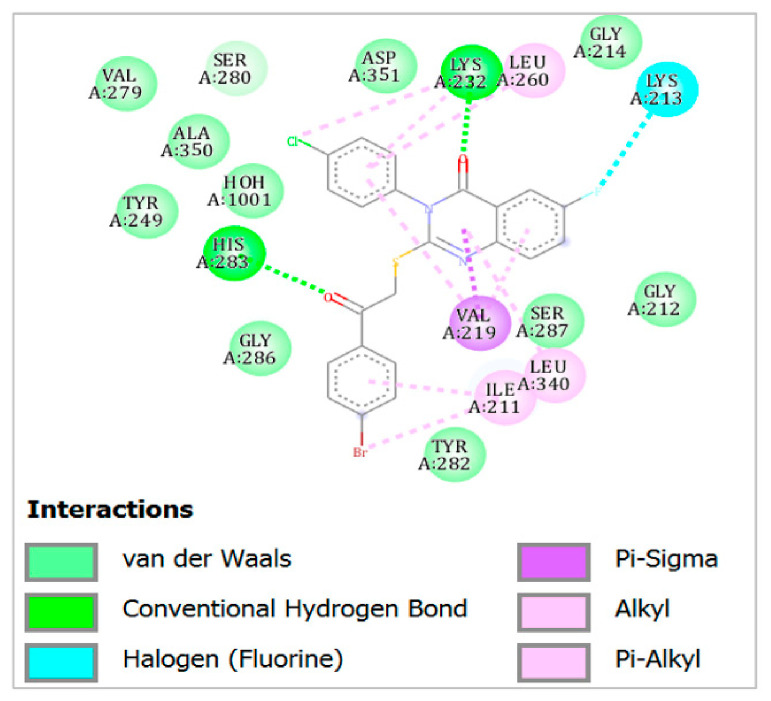
2D interaction diagram showing compound **3e** docking pose within TGF-ꞵ type I receptor kinase domain (PDB ID: 1VJY).

Analysis of the binding mode of compound **3e** (Figure 10 and Figure S3) revealed interactions comparable to those of the co-crystallized ligand with a docking score of −9.5 Kcal/mol (Table 6). Specifically, the side-chain carbonyl oxygen of **3e** forms a hydrogen bond with the hotspot residue His283 at the hinge region. The quinazolin-4(3*H*)-one core fits well within the binding pocket, establishing a hydrogen bond between its 4-carbonyl group and the gatekeeper residue Lys232. Additionally, the quinazolin-4(3*H*)-one ring is further stabilized through an extra halogen bond with Lys213 (Figure 10). These findings suggest that the quinazolin-4(3*H*)-one congener **3e** may function as an ATP-competitive inhibitor by mimicking adenine’s hydrogen-bonding and aromatic stacking interactions within the TGF-βRI kinase hinge region.

Overall, these encouraging computational results are consistent with the potential involvement of TGF-βRI as a plausible anticancer target for compound **3e**.

Molecular dynamics of **3e**

**Figure 11:**
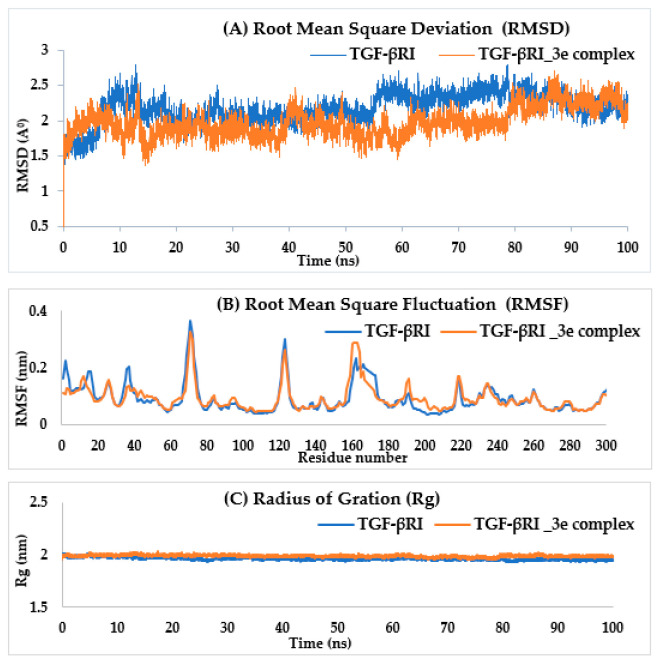
Structural dynamics analysis from 100 ns MD simulations. (**A**) RMSD plot showing the backbone stability of TGF-βRI in complex with compound **3e**. (**B**) RMSF plot illustrating residue flexibility of TGF-βRI upon binding with compound **3e**. (**C**) Rg plot indicating the compactness of the TGF-βRI–compound **3e** complex throughout the simulation.

Molecular dynamics (MD) presents an essential post-docking step that provides deeper insights into the dynamic behaviour and the stability of the protein–ligand complexes under physiologically relevant conditions. Unlike molecular docking, which offers a static view, MD captures atomic-level movements over time. It enables the assessment of structural integrity and the persistence of key interactions [56,57]. In the present study, MD simulations were conducted to validate and further support the reliability of docking predictions, supporting the potential of compound **3e** as a promising lead inhibitor for TGF-βRI with favorable binding characteristics. MD simulations were performed for 100 ns on the TGF-βRI–compound **3e** complex, starting from the docked structure. For comparison, a separate MD simulation was also conducted on the apo-form of the TGF-βRI protein as shown in Figure 11.

The obtained RMSD plot, as depicted in Figure 11A, reveals that the TGF-βRI–compound **3e** complex undergoes a brief equilibration phase during the first 10 ns of the simulation, followed by stable fluctuations between 1.5–2.5 Å, suggesting a well-maintained structural conformation and stable ligand binding throughout the simulation. Supporting this, the RMSF plot (Figure 11B) shows that binding of compound **3e** does not induce significant disturbance in the protein structure, as it displays relatively low atomic fluctuations across most residues with values less than 0.3 nm. The Rg plot indicates consistent compactness of the protein throughout the simulation, with Rg values fluctuating between 1.9–2.2 nm (Figure 11C). These findings together suggest that compound **3e** forms a stable complex with TGF-βR1 throughout the entire 100 ns MD simulation.

TGF-βRI (ALK5) Kinase inhibitory assay

**Figure 12:**
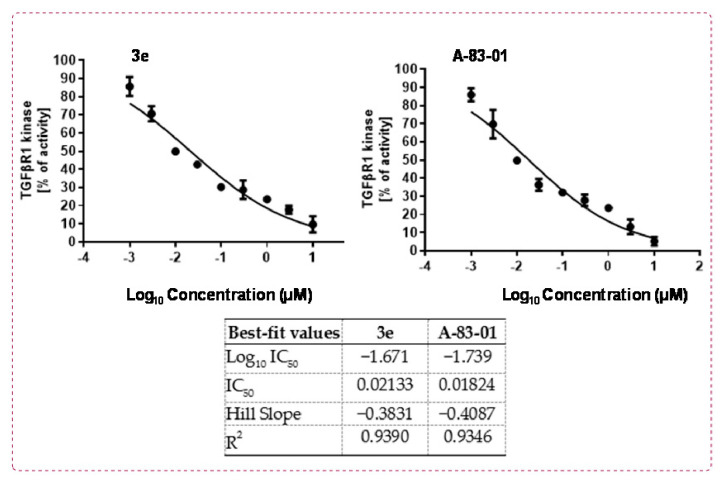
Dose-response curves of compound **3e** and **A-83-01** against TGFβ-RI (ALK5) Kinase.

To corroborate the molecular docking results, a TGFβ-RI kinase inhibition assay was performed. As shown in Figure 12, compound **3e** exhibited potent inhibition of TGF-βRI kinase with an IC_50_ of 0.02134 µM (equivalent to 21.34 nM), closely matching that of the reference inhibitor **A-83-01** (IC_50_ = 0.01824 µM, equivalent to 18.24 nM). 

These results support the ability of compound **3e** to inhibit TGF-βRI kinase activity under the assay conditions. The observed in vitro TGF-βRI kinase inhibitory activity of compound **3e** as a low-micromolar TGF-βRI inhibitor is noteworthy, as most clinically approved kinase inhibitors to date have been directed against receptor tyrosine kinases (RTKs), while receptor serine/threonine kinases such as TGF-βRI remain comparatively less explored despite their critical role in tumor progression, invasion, and immune evasion [58]. Given the frequent challenges associated with RTK inhibitors, including off-target effects and resistance development, the quinazolinone framework of **3e** may represent a valuable starting point for further optimization of TGF-βRI-targeted inhibitors.

#### 2.2.3. Assessment of Cytotoxicity of **3e** in WI-38 Normal Human Lung Fibroblasts

The cytotoxicity of compound **3e** against normal human lung fibroblast WI-38 cells was evaluated using the MTT assay after 72 h exposure to concentrations ranging from 0.1 to 300 µM (0.1, 0.3, 1, 3, 10, 30, 100, and 300 µM). Cell viability (%) was determined relative to untreated control cells, and data from three independent experiments were used to construct concentration–response curves. IC_50_ values were obtained by nonlinear regression analysis and reported as the mean ± SD. Compound **3e** exhibited a mean IC_50_ of 88.32 ± 4.78 µM in WI-38 cells (Figure S4), compared to 10.8 µM in A549 lung cancer cells, resulting in a selectivity index (SI) of 8.2. For the most sensitive cancer cell lines, IC_50_ values ranged from 2.63 to 17.2 µM, corresponding to SI values between 33.58 and 5.13, respectively (Table 2). Since an SI > 1 indicates preferential cytotoxicity toward specific cancer cell lines over normal cells [59], these findings underscore the favorable selectivity profile of **3e**, supporting its potential as a promising lead for the development of low-toxicity anticancer agents.

#### 2.2.4. In Silico Prediction of ADME Parameters of 

Evaluation of ADME properties is a crucial part of drug development. Fortunately, reliable tools like the PreADME web server provide in silico predictions for drug-likeness and ADME profiles. Using this platform, key pharmacokinetic parameters, including HIA (human intestinal absorption), Caco-2 and MDCK permeability, PPB (plasma protein binding), BBB penetration, interactions with important CYP isoforms and P-glycoprotein inhibition, were predicted for the most active compound **3e**, with results summarized in Table 7.

Regarding intestinal absorption, compound **3e** exhibited excellent intestinal absorption with a predicted value of 98%. It demonstrated good permeability in the Caco-2 cell model (43 nm/s), but poor permeability in the MDCK model (0.02 nm/s). Additionally, it displayed limited blood–brain barrier (BBB) penetration, indicating low potential for central nervous system (CNS) activity. Its high plasma protein binding (100%) indicates strong affinity to plasma proteins, which may result in reduced clearance. Compound **3e** was also predicted to be metabolized primarily by the CYP3A4 enzyme. While derivative **3e** was not predicted to inhibit CYP2C19, CYP2D6, or CYP3A4, it may inhibit the CYP2C9 isoform, raising the potential for drug–drug interactions (DDIs) mediated by this enzyme. Furthermore, it was recognized as a potential inhibitor of P-glycoprotein, potentially improving the bioavailability of concurrently administered drugs.

In conclusion, while compound **3e** exhibits excellent oral absorption and avoids major CYP-mediated drug interactions, its high plasma protein binding and CYP2C9 inhibition pose potential risks for drug efficacy and DDIs, indicating the need for further optimization.

## 3. Experimental

### 3.1. Chemistry

Melting points (°C) were determined on an Electrothermal electronic melting point apparatus (UK) and are uncorrected. IR spectra were recorded as KBr discs on Perkin Elmer spectrum BX FT-IR spectrometer and are reported in wave number (cm^−1^). NMR spectra were obtained in DMSO-*d*_6_ or CDCl_3_ using a Joel Eclipse 300 MHz FT NMR for ^1^H-NMR and at 75 MHz for ^13^C, JNM-Ecx500II FT NMR (500 MHz) for ^1^H-NMR and at 125 MHz for ^13^C, and 600 MHz Varian-Agilent-NMR for ^1^H-NMR and at 150 MHz for ^13^C spectrometers. Chemical shifts (*δ*) are reported in ppm relative to TMS as an internal standard. Signal multiplicities are abbreviated as s (singlet), d (doublet), dd (doublet of doublets), t (triplet), q (quartet), m (multiplet), td (triplet of doublets), br. (broad), and app (apparent). Mass spectra were recorded using a single quadrupole mass analyzer (Thermo Scientific GCMS) model (ISO Lt), operating with Thermo X-Calibur software, or using a Time-of-Flight (TOF) mass spectrometer (AccuTOF LC-Plus), which determines the mass-to-charge ratio by accelerating ionized species and measuring their flight time.

#### 3.1.1. General Procedures for Synthesis of 2-(4-Oxo-3-aryl-3,4-dihydroquinazolin-2-ylsulfanyl) Derivatives **3a**–**h**

An equimolar mixture (0.001958 mol) of 3-(aryl)-6-fluoro-2-mercaptoquinazolin-4(3*H*)-one derivative **1a** or **1b** and halo-compound—namely 2-(diethylamino)-*N*-(2,6-dimethylphenyl)acetamide (**2a**), *p*-chlorobenzyl chloride (**2b**), 2-fluorobenzyl bromide (**2c**), or phenacyl bromides (**2d**–**f**)—was dissolved in dry acetone (20 mL). Potassium carbonate (3 equiv., 0.005874 mol, 0.81 g) was added, and the resulting mixture was refluxed for 12 h. The hot mixture was filtered to remove inorganic salts, and the solvent was concentrated under reduced pressure. The resulting crude solids were washed with water, air-dried, and recrystallized from the appropriate solvent to afford compounds **3a**–**h** in pure form.

*2-[3-(4-Chloro-phenyl)-6-fluoro-4-oxo-3,4-dihydro-quinazolin-2-ylsulfanyl]-N-(2,3-dimethyl-phenyl)-acetamide* **3a**

White powder (MeOH/CHCl_3_); yield (78%); m.p. 236–238 °C; IR (KBr)/cm^−1^, ν: 3272 (NH), 3098 & 3060 (CH-aromatic), 2987 & 2923 (CH-aliphatic), 2373, 1696 (C═O), 1652 (C═O), 1587 (C═N), 1550, 1482, 1404, 1340, 1305, 1268, 1223, 1165, 1093, 1019, 966, 923, 824, 780, 756, 705, 651, 585, 512, 449; ^1^H NMR (300 MHz; DMSO-*d*_6_) *δ*_H_: 9.74 (br. s, 1H, NH), 7.85–7.50 (m, 7H, 7 × Ar-H), 7.15–6.97 (m, 3H, 3 × Ar-H), 4.11 (s, 2H, CH_2_), 2.24 (s, 3H, CH_3_), 2.06 (s, 3H, CH_3_); ^13^C NMR (150 MHz; DMSO-*d*_6_) *δ*_C_: 166.1 (NH-C═O), 160.5 (C═O), 159.9 (d, ^1^*J*_C–F_ = 243.8 Hz, *ipso-*C-F), 156.7 (C═N), 144.6, 137.4, 136.3, 135.3, 135.0, 131.8, 130.1, 127.6, 125.6 (7 × CH-Ar, 6 × C_q_-Ar), 129.2 (app. d, *meta*-CH), 123.8 (d, ^2^*J*_C–F_ = 23.0 Hz, *ortho*-CH), 121.1 (app. d, *meta*-C_q_), 111.7 (d, ^2^*J*_C–F_ = 24.2 Hz, *ortho*-CH), 36.9 (CH_2_), 20.5 & 14.5 (2 × CH_3_); MS (EI) *m*/*z* (%) [M^+^ + 1] 468.70 (16.31) and [M^+^] 467.26 (46.73) for C_24_H_19_ClFN_3_O_2_S, 450.55 (52.67), 481.49 (37.16), 410.07 (40.11), 387.43 (28.92), 368.44 (100.00), 353.66 (55.97), 314.36 (43.66), 297.66 (56.06), 280.35 (36.44), 232.77 (28.60), 175.31 (28.13), 125.35 (26.23), 108.19 (36.57), 91.11 (63.15), 81.34 (63.00).

*2-(4-Chloro-benzylsulfanyl)-3-(4-chloro-phenyl)-6-fluoro-3H-quinazolin-4-one* **3b**

White powder (MeOH/CHCl_3_); yield (86%), m.p. 200–202 °C; IR (KBr)/cm^−1^, ν: 3095 & 3057 (CH-aromatic), 2979 (CH-aliphatic), 2371, 1686 (C═O), 1587 (C═N), 1549, 1482, 1403, 1340, 1302, 1257, 1221, 1171, 1015, 967, 919, 863, 829, 730, 641, 605, 556, 510, 448; ^1^H NMR (300 MHz; CDCl_3_) *δ*_H_: 7.92–7.82 (m, 1H, Ar-H), 7.67 (dd, 1H, *J* = 8.7, 4.5 Hz, Ar-H), 7.50 (d, 3H, *J* = 7.5, 3 × Ar-H), 7.38–7.18 (m, 6H, 6 × Ar-H), 4.37 (s, 2H, benzylic-CH_2_); ^13^C NMR (75 MHz; CDCl_3_) *δ*_C_: 160.3 (C═O), 159.8 (d, ^1^*J*_C–F_ = 257.6 Hz, *ipso-*C-F), 155.1 (C═N), 143.6, 135.8, 134.2, 133.2, 132.9, 130.0, 129.8, 129.5, 128.1 (8 × CH-Ar & 5 × C_q_-Ar), 127.8 (d, ^3^*J*_C–F_ = 8.0 Hz, *meta*-CH), 122.7 (d, ^2^*J*_C–F_ = 23.5 Hz, *ortho*-CH), 120.2 (app. d, *meta*-C_q_), 111.6 (d, ^2^*J*_C–F_ = 23.5 Hz, *ortho*-CH), 35.7 (benzylic-CH_2_); MS (EI) *m*/*z* (%) [M^+^ + 1, ^37^Cl] 333.27 (13.59) and [M^+^, ^37^Cl] 432.71 (27.48), [M^+^ + 1, ^35^Cl] 430.85 (44.13) and [M^+^, ^35^Cl] 430.24 (11.13), [M^+^ − 1, ^35^Cl] 429.35 (51.83) for C_21_H_13_Cl_2_FN_2_OS, 417.20 (53.59), 415.75 (39.52), 396.31 (35.53), 394.23 (22.90), 381.92 (33.00), 379.53 (100.00), 348.11 (35.36), 334.70 (45.42), 313.00 (27.93), 301.15 (25.98), 267.63 (19.15), 242.71 (16.65), 218.23 (27.31), 181.70 (31.41), 173.25 (39.68), 153.26 (59.76), 108.88 (43.37), 91.48 (25.02).

*6-Fluoro-2-(2-fluoro-benzylsulfanyl)-3-phenyl-3H-quinazolin-4-one* **3c**

White powder (EtOH/CHCl_3_); yield (91%), m.p. 158–160 °C; IR (KBr)/cm^−1^, ν: 3064(CH-aromatic), 2927 (CH-aliphatic), 1955, 1682 (C═O), 1582 (C═N), 1549, 1479, 1401, 1341, 1294, 12657, 1217, 1095, 1070, 1027, 965, 946, 872, 830, 750, 689, 635, 552, 524,499, 459; ^1^H NMR (600 MHz; CDCl_3_) *δ*_H_: 7.85 (s, 1H, Ar-H), 7.67 (s, 1H, Ar-H), 7.56–7.40 (m, 5H, 5 × Ar-H), 7.32–7. 16 (m, 3H, 3 × Ar-H), 7.08–6.95 (m, 2H, 2 × Ar-H), 4.42 (s, 2H, benzylic-CH_2_); ^13^C NMR (150 MHz; CDCl_3_) *δ*_C_: 161.1 (C═O), 161.1 (d, ^1^*J*_C–F_ = 247.2 Hz, *ipso*-C-F), 160.2 (d, ^1^*J*_C–F_ = 245.0 Hz, *ipso*-C-F), 156.2 (C═N), 144.5, 135.4, 131.4, 130.1, 129.7, 129.0, 124.1, 123.7, 123.6 (6 × CH-Ar & 3 × C_q_-Ar), 129.4 (d, ^3^*J*_C–F_ = 8.1 Hz, *meta*-CH), 128.5 (d, ^3^*J*_C–F_ = 6.9 Hz, *meta*-CH), 123.1 (d, ^2^*J*_C–F_ = 24.2 Hz, 2 × *ortho*-CH), 121.0 (d, ^3^*J*_C–F_ = 8.1 Hz, *meta*-C_q_), 115.4 (d, ^2^*J*_C–F_ = 21.9 Hz, *ortho*-CH), 112.0 (d, ^2^*J*_C–F_ = 23.0 Hz, *ortho*-CH), 30.1 (benzylic-CH_2_); MS (EI) *m*/*z* (%) [M^+^] 380.24 (22.07) for C_21_H_14_F_2_N_2_OS, 367.70 (21.89), 343.38 (27.39), 325.27 (50.07), 296.17 (25.77), 284.33 (100.00), 272.09 (37.10), 237.43 (54.10), 199.92 (32.58), 165.50 (34.27), 122.01 (38.65), 95.26 (68.21), 83.10 (43.01), 61.06 (45.63), 42.10 (95.17).

*3-(4-Chloro-phenyl)-6-fluoro-2-(2-oxo-2-phenyl-ethylsulfanyl)-3H-quinazolin-4-one* **3d**

White powder (EtOH/CHCl_3_); yield (82%); m.p. 196–198 °C; IR (KBr)/cm^−1^, ν: 3055 (CH-aromatic), 2922 (CH-aliphatic), 2369, 1689 (ketonic-C═O), 1586 (lactamic-C═O), 1548 (C═N), 1479, 1400, 1373, 1339, 1298, 1270, 1227, 1204, 1169, 1093, 1087, 997, 971, 926, 882, 830, 778, 742, 687, 649, 599, 561, 510, 447, 412; ^1^H NMR (300 MHz; CDCl_3_) *δ*_H_: 8.07 (dd, 2H, *J* = 7.2, 1.2 Hz, 2 × Ar-H), 7.83 (dd, 1H, *J* = 8.4, 3.0 Hz, Ar-H), 7.70–7.61 (m, 1H, Ar-H), 7.56–7.51 (m, 4H, 4 × Ar-H), 7.38–7.22 (m, 4H, 4 × Ar-H), 4.63 (s, 2H, CH_2_); ^13^C NMR (150 MHz; CDCl_3_) *δ*_C_: 193.2 (ketonic-C═O), 160.8 (lactamic-C═O), 160.2 (d, ^1^*J*_C–F_ = 246.2 Hz, *ipso*-C-F), 155.3 (C═N), 144.1, 136.5, 136.3, 133.8, 133.7, 130.5, 130.2, 128.8, 128.4 (9 × CH-Ar, 4 × C_q_-Ar), 128.3 (d, ^3^*J*_C–F_ = 8.1 Hz, *meta*-CH) 123.2 (d, ^2^*J*_C–F_ = 23.0 Hz, *ortho*-CH), 120.8 (d, ^3^*J*_C–F_ = 8.1 Hz, *meta*-C_q_), 112.1 (d, ^2^*J*_C–F_ = 23.0 Hz, *ortho*-CH), 39.7 (CH_2_); MS (EI) *m*/*z* (%) [M^+^] 424.36 (19.43) for C_22_H_14_ClFN_2_O_2_S, 396.94 (24.28), 385.34 (29.74), 339.59 (43.22), 328.00 (65.56), 262.49 (30.86), 250.08 (38.52), 234.68 (100.00), 223.13 (25.33), 208.01 (45.96), 168.69 (33.59), 149.90 (52.50), 87.93 (37.86), 78.37 (68.49), 61.26 (60.75).

*2-[2-(4-Bromo-phenyl)-2-oxo-ethylsulfanyl]-3-(4-chloro-phenyl)-6-fluoro-3H-quinazolin-4-one* **3e**

White powder (MeOH/CHCl_3_); yield (84%); m.p. 206–208 °C; IR (KBr)/cm^−1^, ν: 3064 (CH-aromatic), 2906 (CH-aliphatic), 2373, 1688 (ketonic-C═O), 1582 (lactamic-C═O), 1551 (C═N), 1481, 1399, 1340, 1298, 1270, 1199, 1174, 1071, 1013, 989, 968, 928, 836, 811, 779, 736, 703, 648, 605, 556, 510, 448; ^1^H NMR (300 MHz; CDCl_3_) *δ*_H_: 7.94 (d, 2H, *J* = 8.4 Hz, 2 × Ar-H), 7.83 (dd, 1H, *J* = 8.1, 3.0 Hz, Ar-H), 7.70 (d, 2H, *J* = 8.7 Hz, Ar-H), 7.56 (d, 2H, *J* = 8.7 Hz, 2 × Ar-H), 7.41–7.18 (m, 4H, 4 × Ar-H), 4.52 (s, 2H, CH_2_); ^13^C NMR (125 MHz; CDCl_3_) *δ*_C_: 192.5 (ketonic- C═O), 160.9 (lactamic-C═O), 160.4 (d, ^1^*J*_C–F_ = 246.8 Hz, *ipso*-C-F), 155.2 (C═N), 144.1, 136.7, 135.1, 133.9, 132.2, 130.5, 130.3, 130.0, 129.0 (8 × CH-Ar, 5 × C_q_-Ar), 128.3 (d, ^3^*J*_C–F_ = 8.3 Hz, *meta*-CH), 123.4 (d, ^2^*J*_C–F_ = 23.8 Hz, *ortho*-CH), 120.9 (d, ^3^*J*CF = 8.4 Hz, *meta*-C_q_), 112.3 (d, ^2^*J*_C–F_ = 23.8 Hz, *ortho*-CH), 39.5 (CH_2_); MS (EI) *m*/*z* (%) [M^+^, ^37^Cl] 504.22 (12.29), [M^+^, ^35^Cl] 501.62 (21.65) for C_22_H_13_BrClFN_2_O_2_S, 485.73 (14.82), 454.17 (31.13), 408.55 (32.87), 387.35 (26.58), 353.92 (60.31), 347.68 (38.22), 333.21 (62.70), 274.90 (32.03), 254.67 (89.11), 252.05 (100.00), 246.39 (40.85), 237.95 (43.34), 225.37 (52.34), 215.16 (43.81), 213.99 (53.01), 163.14 (35.27), 161.73 (30.92), 143.58 (29.27), 132.67 (43.11), 128.80 (68.24), 119.75 (40.88), 114.78 (40.11), 100.78 (26.23), 89.21 (73.49), 88.36 (56.69), 68.99 (49.08), 62.83 (34.53), 54.37 (22.75).

*3-(4-Chloro-phenyl)-6-fluoro-2-(2-oxo-2-p-tolyl-ethylsulfanyl)-3H-quinazolin-4-one* **3f**

White powder (Me_2_CO/CHCl_3_); yield (82%); m.p. 196–198 °C; IR (KBr)/cm^−1^, ν: 3097 & 3067 (CH-aromatic), 2917 (CH-aliphatic), 2374, 1691 (ketonic-C═O), 1609 (lactamic-C═O), 1550 (C═N), 1484, 1404, 1375, 1343, 1298, 1268, 1223, 1091, 1005, 971, 893, 830, 778, 737, 702, 648, 601, 560, 510, 447; ^1^H NMR (300 MHz; DMSO-*d*_6_) *δ*_H_: 7.98 (d, 2H, *J* = 8.1 Hz, 2 × Ar-H), 7.76–7.54 (m, 6H, 6 × Ar-H), 7.40 (d, 2H, *J* = 7.8 Hz, 2 × Ar-H), 7.23 (dd, 1H, *J* = 9.0, 4.8 Hz, Ar-H), 4.73 (s, 2H, CH_2_), 2.42 (s, 3H, CH_3_); ^13^C NMR (75 MHz; CDCl_3_) *δ*_C_: 192.9 (ketonic-C═O), 159.8 (C_q_-F and lactamic-C═O), 155.9 (C═N), 143.9, 134.8, 134.6, 133.7, 131.3, 129.7, 129.3, 128.3, 123.6, 123.1, 120.3, 111.4, 110.7 (12 × CH-Ar, 5 × C_q_-Ar), 39.5 (CH_2_), 21.2 (CH_3_); MS (EI) *m*/*z* [M^+^ + 1] 438.94 (18.79), for C_23_H_16_ClFN_2_O_2_S. 430.01 (31/67), 414.12 (46/94), 401.47 (22.16), 365.34 (18.14), 340.86 (32.01), 326.47 (22.06), 282.95 (55.64), 262.98 (31.45), 255.24 (21.63), 243.22 (38.90), 193.65 (37.28), 180.36 (49.18), 146.18 (33.62), 137.89 (58.36), 117.76 (21.41), 102.88 (34.15), 89.15 (43.71), 73.85 (100.00), 65.19 (53.20), 55.97 (80.20), 40.74 (39.79).

*6-Fluoro-2-(2-oxo-2-p-tolyl-ethylsulfanyl)-3-phenyl-3H-quinazolin-4-one* **3g**

White powder (EtOH/CHCl_3_); yield (88%); m.p. 166–168 °C; IR (KBr)/cm^−1^, ν: 3063 (CH-aromatic), 2957 & 2918 (CH-aliphatic), 1685 (ketonic-C═O), 1601 (lactamic-C═O), 1545 (C═N), 1480, 1392, 1342, 1343, 1295, 1265, 1223, 1174, 1109, 1002, 972, 897, 820, 783, 747, 693, 640, 591, 560, 529, 503, 470, 409; ^1^H NMR (300 MHz; DMSO-*d*_6_) *δ*_H_: 7.98 (d, 2H, *J* = 8.4 Hz, 2 × Ar-H), 7.73 (dd, 1H, *J* = 8.4, 3.0 Hz, Ar-H), 7.69–7.59 (m, 3H, 3 × Ar-H), 7.54–7.48 (m, 2H, 2 × Ar-H), 7.40 (d, 2H, *J* = 8.1 Hz, Ar-H), 7.23 (dd, 1H, *J* = 9.0, 4.8 Hz, Ar-H), 4.71 (s, 2H, CH_2_), 2.43 (s, 3H, CH_3_); ^13^C NMR (150 MHz; CDCl_3_) *δ*_C_: 192.9 (ketonic-C═O), 160.1 (d, ^1^*J*_C–F_ = 245 Hz, *ipso*-C-F & lactamic-C═O), 155.8 (C═N), 144.5, 144.2, 135.5, 133.8, 130.3, 129.9, 129.4, 129.0, 128.5 (9 × CH-Ar, 4 × C_q_-Ar), 128.3 (d, ^3^*J*_C–F_ = 9.2 Hz, *meta*-CH), 123.0 (d, ^2^*J*_C–F_ = 24.2 Hz, *ortho*-CH), 120.9 (d, ^3^*J*_C–F_ = 9.3 Hz, *meta*-C_q_), 112.1 (d, ^2^*J*_C–F_ = 23.0 Hz, *ortho*-CH), 39.6 (CH_2_), 21.7 (CH_3_); MS (EI) *m*/*z* [M^+^] 404.14 (20.89) for C_23_H_17_FN_2_O_2_S, 381.92 (28.97), 375.62 (92.96), 368.47 (25.64), 342.22 (24.19), 326.15 (39.21), 305.61 (24.70), 274.55 (21.20), 243.09 (44.63), 229.95 (24.88), 214.96 (26.61), 190.16 (48.45), 149.94 (70.60), 134.07 (36.79), 85.17 (68.61), 76.90 (60.61), 60.02 (36.86), 54.98 (74.23), 51.11 (100.00).

*2-[2-(4-Bromo-phenyl)-2-oxo-ethylsulfanyl]-6-fluoro-3-phenyl-3H-quinazolin-4-one* **3h**

Beige powder (EtOH/CHCl_3_); yield (84%); m.p. 172–174 °C; IR (KBr)/cm^−1^, ν: 3064 (CH-aromatic), 2959 & 2899, 1690 (ketonic-C═O), 1581 (lactamic-C═O), 1547 (C═N), 1481, 1396, 1345, 1296, 1268, 1223, 1196, 1111, 1068, 987, 892, 835, 810, 779, 751, 698, 641, 556, 500, 449; ^1^H NMR (600 MHz; CDCl_3_) *δ*_H_: 7.91 (apparent s, 2H, 2 × Ar-H), 7.79 (apparent s, 1H, Ar-H), 7.64 (apparent s, 2H, 2 × Ar-H), 7.54 (apparent s, H, 3 × Ar-H), 7.32 (apparent s, 3H, 3 × Ar-H), 7.07 (apparent s, 1H, Ar-H), 4.46 (s, 2H, CH_2_); ^13^C NMR (150 MHz; CDCl_3_) *δ*_C_: 192.6 (ketonic-C═O), 160.2 (d, ^1^*J*_C–F_ = 246.2 Hz, *ipso*-C-F), 160.9 (lactamic-C═O), 155.5 (C═N), 144.1, 135.4, 135.1, 132.1, 130.3, 129.9, 129.0, 128.8 (9 × CH-Ar, 4 × C_q_-Ar), 128.1 (d, ^3^*J*_C–F_ = 6.9 Hz, *meta*-CH), 123.1 (d, ^2^*J*_C–F_ = 24.2 Hz, *ortho*-CH), 121.0 (d, ^3^*J*_C–F_ = 6.9 Hz, *meta*-C_q_), 112.2 (d, ^2^*J*_C–F_ = 24.2 Hz, *ortho*-CH), 39.3 (CH_2_); MS (EI) *m*/*z* [M^+^, ^81^Br & ^34^S] 472.19 (2.33), [M^+^ + H, ^81^Br] 470.74 (4.24) for C_22_H_14_BrFN_2_O_2_S, 469.72 (14.45), 468.51 (13.81), 452.22 (46.50), 450.28 (100.00), 419.80 (33.93), 410.86 (15.45), 394.69 (21.05), 383.33 (18.01), 359.21 (14.56), 307.82 (20.27), 293.33 (17.62), 267.79 (23.40), 242.46 (28.17), 221.55 (20.10), 175.34 (20.87), 139.02 (61.81), 109.50 (21.65), 97.31 (47.39), 81.31 (26.03), 74.60 (25.16), 61.61 (16.27).

#### 3.1.2. General Procedures for Synthesis of Schiff Bases **6a**–**d**

An equimolar mixture (0.002 mol) of 3-amino-2-methyl-3*H*-quinazolin-4-one **4a** or 3-amino-6,7-dimethoxy-2-methyl-3*H*-quinazolin-4-one **4b** and the appropriate aromatic aldehyde **5a**–**d**, namely 3-formyl benzoic acid, 3-formyl benzonitrile, iso vanillin or 5-iodovanillin, in absolute ethanol (20 mL) containing glacial acetic acid (3 mL, catalytic amount), was refluxed for 10 h. The reaction mixture was concentrated under reduced pressure or poured onto ice–water and the resulting solid was collected, washed with water, air-dried, and recrystallized from the appropriate solvent to afford the corresponding Schiff bases.

*(E)-3-[(2-Methyl-4-oxo-4H-quinazolin-3-ylimino)-methyl]-benzoic acid* **6a**

Obtained via concentration under reduced pressure as off-while fluffy powder (CHCl_3_); yield (92%), m.p. 271–273 °C; IR (KBr)/cm^−1^, ν: 3055 (CH-aromatic), 2926 (CH-aliphatic), 2369, 1721 (carboxylic-C═O), 1682 (amidic-C═O), 1598 (HC═N & C═N), 1478, 1437, 1368, 1340, 1260, 1211, 1157, 1111, 1037, 1035, 976, 937, 878, 772, 749, 660, 632, 555, 470; ^1^H-NMR (300 MH_Z_; DMSO-*d*_6_) *δ*_H_: 9.14 (s, 1H, CH═N), 8.51 (s, 1H, Ar-H), 8.19 (apparent ABq, 3H, *J* = 7.5 Hz 3 × Ar-H), 7.84 (t, 1H, *J* = 6.9 Hz, CH-Ar), 7.76–7.63 (m, 2H, 2 × Ar-H), 7.53 (t, 1H, *J* = 7.5 Hz, Ar-H), 2.57 (3H, s, CH_3_); ^13^C NMR (150 MHz; CDCl_3_) *δ*_C_: 169.1 (carboxylic-C═O), 167.0 (amidic-C═O), 157.8 (C_q_═N), 153.8 (HC═N), 146.7, 134.9, 133.6, 133.2, 133.2, 130.1, 129.7, 127.2, 127.1, 126.9, 121.4 (8 × CH-aromatic & 4 × C_q_-aromatic), 22.6 (CH_3_).

*(E)-3-[(2-Methyl-4-oxo-4H-quinazolin-3-ylimino)-methyl]-benzonitrile* **6b**

Obtained via concentration under reduced pressure as off-white powder (MeOH/CHCl_3_), yield (78%), m.p. 150–152 °C; IR (KBr)/cm^−1^, ν: 3059 & 3031 (CH-aromatic), 2929 (CH-aliphatic), 2371, 2231 (C≡N), 1674 (amidic-C═O), 1604 (C═N & HC═N), 1469, 1425, 1366, 1332, 1296, 1247, 1226, 1160, 1123, 1028, 960, 928, 880, 803, 771, 691, 662, 625, 554, 439; ^1^H-NMR (600 MH_Z_; CDCl_3_) *δ*_H_: 9.29 (s, 1H, CH═N), 8.30–8.14 (m, 2H, 2 × Ar-H), 8.05 (d, 1H, *J* = 1.2 Hz, Ar-H), 7.82–7.70 (m, 2H, 2 × Ar-H),), 7.68–7.56 (m, 2H, 2 × Ar-H), 7.45 (apparent s, 1H, Ar-H), 2.68 (3H, s, CH_3_); ^13^C NMR (150 MHz; CDCl_3_) *δ*_C_: 161.9 (amidic-C═O), 159.0 (C_q_═N), 154.1 (HC=N), 146.2, 135.0, 134.6, 132.7, 131.7, 130.3, 129.9, 127.3, 127.0, 126.7, 121.4, 117.9, 113.5 (8 × CH-Ar & 4 × C_q_-Ar & C≡N), 23.0 (CH_3_).

*(E)-3-((3-Hydroxy-4-methoxybenzylidene)amino)-6,7-dimethoxy-2-methylquinazolin-4(3H)-one* **6c**

Obtained via concentration under reduced pressure as off-white powder (MeOH); yield (84%); m.p. 228–230 °C; IR (KBr)/cm^−1^, ν: 3408 (OH), 3018 (CH-Ar), 2975 (CH-aliphatic), 2370, 2341, 1670 (amidic-C═O), 1611 (C═N & HC═N), 1507, 1466, 1444, 1397, 1332, 1282, 1250, 1212, 1177, 1113, 1021, 971, 864, 810, 768, 693, 595, 540, 513, 438; ^1^H-NMR (300 MH_Z_; CDCl_3_) *δ*_H_: 8.81 (s, 1H, CH═N), 7.63–7.56 (m, 2H, 2 × Ar-H), 7.33 (dd, 1H, *J* = 8.4, 2.0 Hz, Ar-H), 7.23–7.25 (m, 1H, Ar-H), 6.94 (apparent d, 1H, *J* = 8.4 Hz, Ar-H), 4.02, 3.99, 3.97 (3s, 9H, 3 × OCH_3_), 2.72 (s, 3H, CH_3_), 2.24 (br. s, 1H, OH); ^13^C NMR (150 MHz; CDCl_3_) *δ*_C_: 166.2 (amidic-C═O), 158.2 (C_q_═N), 154.9 (HC=N), 152.8, 150.3, 148.7, 146.0 (4 × C_q_-O), 142.8, 126.3, 123.4, 114.6, 113.0, 110.3, 107.2, 106.0 (5 × CH-Ar & 3 × C_q_-Ar), 55.3, 55.1 (3 × OCH_3_), 22.6 (CH_3_); MS (EI) *m*/*z* (%) [M^+^ + H] 369.68 (20.05) for C_19_H_19_N_3_O_5_, 358.39 (52.01), 347.66 (48.90), 342.84 (38.41), 310.46 (74.12), 298.44 (67.02), 274.70 (22.82), 257.89 (49.63), 237.46 (76.33), 216.08 (40.56), 205.40 (41.27), 186.26 (46.21), 160.10 (23.58), 134.26 (40.00), 123.99 (100.00), 110.10 (46.02), 98.00 (25.28), 79.10 (63.96), 43.12 (56.63).

*(E)-3-[(4-Hydroxy-3-iodo-5-methoxy-benzylidene)-amino]-6,7-dimethoxy-2-methyl-3H-quinazolin-4-one* **6d**

Obtained by pouring onto ice/H_2_O mixture as shinny beige powder (EtOH), m.p. 214–216 °C; IR (KBr)/cm^−1^, ν: 3007 (CH-Ar), 2976 (CH-aliphatic), 2371, 1670 (amidic-C═O), 1608 (HC═N and C═N), 1552, 1499, 1394, 1332, 1293, 1247, 1206. 1173, 1135, 1037, 987, 867, 842, 787, 719, 675, 601, 549, 518, 440; ^1^H-NMR (600 MHz; DMSO-*d*_6_) *δ*_H_: 10.49 (br. s, 1H, OH), 8.68 (s, 1H, CH═N), 7.63–7.56 (m, 2H, CH-Ar), 7.33 (dd, 1H, *J* = 8.4, 2.0 Hz, CH-Ar), 7.23–7.25 (m, 1H, CH-Ar), 7.80 (s, 1H, CH-Ar), 7.54 (s, 1H, CH-Ar), 7.39 (s, 1H, CH-Ar), 7.09 (s, 1H, CH-Ar), 3.88 & 3.84 (2s, 6H, OCH_3_), 2.44 (s, 3H, CH_3_); ^13^C NMR (150 MHz; DMSO-*d*_6_) *δ*_C_: 168.2 (amidic-C═O), 157.2 (C_q_═N), 155.0 (HC=N), 152.1, 151.0, 148.7, 147.7 (4 × C_q_-O), 142.9, 133.1, 125.9, 114.2, 110.6, 108.0, 105.9, (4 × CH-Ar & 3 × C_q_-Ar), 84.8 (C_q_-I), 55.7, 56.4, 56.1 (3 × OCH_3_), 22.5 (CH_3_); MS (DART-ToF) *m*/*z* [M^+^ + 1] 496.03963 for C_19_H_18_IN_3_O_5_ (495.03).

#### 3.1.3. Synthesis of *E*-*N’*-(6-Fluoro-2-methyl-4-oxoquinazolin-3(4*H*)-yl)-*N*,*N*-dimethylformimidamide **8**

A mixture of 3-amino-6-fluoro-2-methylquinazolin-4(3*H*)-one **5c** (0.0026 mol, 0.5 g) and *N*,*N*-dimethyl formamide dimethyl acetal **7** (5.39 equiv. 0.014 mol, 1.66 g, 1.85 mL) in absolute ethanol (15 mL) was refluxed for 5 h. The excess solvent was removed under reduced pressure. The formed solid was washed with *N*-hexane and recrystallized from ethanol to afford the *title compound* as white powder; yield (47%); m.p. 152–154 °C; IR (KBr)/cm^−1^, ν: 3079, 3039 & 3007 (CH-Ar), 2918 & 2819 (CH-aliphatic), 2370, 3425, 1675 (amidic-C═O), 1624 (C═N), 1587 (HC═N), 1482, 1429, 1331, 1271, 1232, 1188, 1119, 1071, 907, 873, 837, 802, 721, 652, 597, 570, 513, 454; ^1^H-NMR (300 MH_Z_; CDCl_3_) *δ*_H_: 7.78 (dd, 1H, ^3^*J*_H_^5^_-F_ = 8.6 & ^4^*J*_H_^5^_-H_^7^ = 3.2 Hz, CH^5^-Ar), 7.71 (s, 1H, HC═N), 7.56 (dd, 1H, ^3^*J*_H_^8^_-H_^7^ = 9.0 & ^4^*J*_H_^8^_-F_ = 5.1 Hz, CH^8^-Ar), 7.36 (td, 1H, ^3^*J*_H_^7^_-H_^8^ *=* ^3^*J*_H_^7^_-F_ *=* 8.7 & ^4^*J*_H_^7^_-H_^5^ = 3.0 Hz, CH^7^-Ar), 3.04, 3.09 (2s, 6H, N(CH_3_)_2_), 2.49 (s, 3H, CH_3_); ^13^C NMR (75 MHz; CDCl_3_) *δ*_C_: 161.6 (lactamic-C═O), 159.7 (d, ^1^*J*_C–F_ = 244.4 Hz, *ipso*-C-F), 159.1 (C_q_═N), 154.0 (HC═N), 142.9 (C_q8a_-quinazolinone), 128.5 (d, ^3^*J*_C–F_ = 8.0 Hz, *meta*-CH), 122.0 (C_q4a_-quinazolinone), 121.7 (d, ^2^*J*_C–F_ = 24.1 Hz, *ortho*-CH), 110.7 (d, ^2^*J*_C–F_ = 23.5 Hz, *ortho*-CH), 40.6 & 34.2 (2 × N-CH_3_), 22.2 (CH_3_-quinazolinone); MS (EI) *m*/*z* [M^+^ + H] 249.42 (30.51) and [M^+^] 248.00 (100.00) for C_12_H_13_FN_4_O, 203.94 (13.04), 177.93 (54.71), 135.89 (15.54), 108.14 (19.27), 71.13 (34.08), 54.86 (20.43), 42.44 (27.94).

#### 3.1.4. General Procedure for Synthesis of Carbamate Derivatives **10a**,**b**

A mixture of 3-amino-2-methyl-3*H*-quinazolin-4-one derivatives **4d**,**e** (0.0028 mol) and an excess of isobutyl chloroformate **9** (20.4 equiv., 0.571 mol, 7.8 g, 7.4 mL) was heated under reflux for 10 h. The excess reagent was evaporated under reduced pressure, and the residue was treated with diethyl ether to induce solidification. The precipitated solid was filtered, washed with water, air-dried, and recrystallized from the appropriate solvent to afford the pure product.

*Isobutyl (6-bromo-2-methyl-4-oxoquinazolin-3(4H)-yl)carbamate* **10a**

Copper powder (MeOH); yield (65%); m.p. 176–177 °C; IR (KBr)/cm^−1^, ν: 3250 (NH), 3089 (CH-Ar), 2961 & 2875 (CH-aliphatic), 2747, 1751 (OC═O), 1679 (amidic-C═O), 1610 (C═N), 1510, 1468, 1427, 1372, 1320, 1274, 1243, 1133, 1046, 967, 851, 794, 724, 673, 644, 572, 531, 469; ^1^H-NMR (300 MH_Z_; DMSO-*d*_6_) *δ*_H_: 10.63 (s, 1H, NH), 8.17 (d, 1H, d, *J* = 2.1 Hz, Ar-H), 7.97 (apparent dd, 1H, dd, *J* = 9.0, 2.1 Hz, Ar-H), 7.59 (d, 1H, *J* = 8.4 Hz, Ar-H), 3.96 (d, 2H, *J* = 6.0 Hz, O-CH_2_-CH-(CH_3_)_2_), 2.45 (3H, s, CH_3_-quinazolin-4(3*H*)-one), 1.95 (1H, apparent quintet, *J* = 6.6 Hz, O-CH_2_-CH-(CH_3_)_2_), 0.92 (d, 6H, *J* = 6.6 Hz, O-CH_2_-CH-(CH_3_)_2_); ^13^C NMR (75 MHz; DMSO-*d*_6_) *δ*_C_: 158.3 (C═O), 157.5 (C═O), 155.7 (C═N), 145.3, 138.0, 129.2, 128.5, 122.1, 119.3 (3 × CH-Ar & 3 × C_q_-Ar), 71.5 (O-CH_2_-CH-(CH_3_)_2_), 27.7 (O-CH_2_-CH-(CH_3_)_2_), 21.2 (CH_3_-quinazolin-4(3*H*)-one), 18.7 (O-CH_2_-CH-(CH_3_)_2_); MS (EI) *m*/*z* (%): [M^+^ + 2, ^81^Br] 357.41 (4.94), [M^+^ + 1, ^81^Br] 355.78 (14.35), [M^+^, ^81^Br] 354.97 (100.00), [M^+^ + 1, ^79^Br] 354.03 (27.91), [M^+^, ^79^Br] 353.03 (77.27) and [M^+^ − 1, ^79^Br] 352.02 (7.88) for C_14_H_16_BrN_3_O_3_, 339.93 (6.16), 311.94 (5.69), 297.19 (8.35), 279.65 (12.59), 266.85 (2.81), 253.16 (30.26), 224.90 (3.89), 208.01 (2.27), 197.72 (1.41), 183.45 (1.62), 170.22 (3.50), 154.18 (3.92), 143.60 (2.49), 117.12 (8.55), 89.17 (9.25), 75.16 (37.43), 68.48 (4.19), 57.19 (52.62), 41.26 (18.34).

*Isobutyl (2,6-dimethyl-4-oxoquinazolin-3(4H)-yl)carbamate* **10b**

Beige powder (Hexane); yield (63%), m.p. 101–102 °C; IR (KBr)/cm^−1^, ν: 3250 (NH), 3060 (CH-Ar), 2968 & 2875 (CH-aliphatic), 2273, 1752 (OC═O), 1702 (amidic-C═O), 1610, (C═N), 1491, 1381, 1292, 1267, 1233, 1124, 1038, 968, 926, 832, 770, 705, 663, 596, 541, 435; ^1^H-NMR (500 MH_Z_; DMSO-*d*_6_) *δ*_H_: 10.40 (s, 1H, NH), 7.89 (apparent s, 1H, Ar-H), 7.74 (dd, 1H, *J* = 8.5, 2.0 Hz, Ar-H), 7.56 (d, 1H, *J* = 8.5 Hz, Ar-H), 3.99 (d, 2H, *J* = 6.5 Hz, O-CH_2_-CH-(CH_3_)_2_), 2.40 & 2.38 (2s, 3H, 2 × CH_3_-quinazolin-4(3*H*)-one), 1.79 (apparent septet, 1H, *J* = 6.5 Hz, O-CH_2_-CH-(CH_3_)_2_), 0.72 (d, 6H, *J* = 7.0 Hz, O-CH_2_-CH-(CH_3_)_2_); ^13^C NMR (125 MHz; DMSO-*d*_6_) *δ*_C_: 158.5 (C═O), 153.8 (C═O), 150.0 (C═N), 144.6, 137.6, 137.3, 127.4, 126.1, 120.1 (3 × CH-Ar & 3 × C_q_-Ar), 74.0 (O-CH_2_-CH-(CH_3_)_2_), 27.5 (O-CH_2_-CH-(CH_3_)_2_), 21.0 & 20.7 (2 × CH_3_-quinazolin-4(3*H*)-one), 18.6 (O-CH_2_-CH-(CH_3_)_2_); MS (EI) *m*/*z* (%) [M^+^ + 2] 291.21 (4.57), [M^+^ + 1] 290.22 (26.29), and [M^+^] 289.11 (100.00) for C_15_H_19_N_3_O_3_, 287.78 (2.73), 216.37 (3.47), 189.20 (6.50), 174.00 (2.44), 160.36 (4.05), 131.33 (3.46), 103.92 (3.42), 89.32 (10.77), 77.32 (11.94), 63.29 (6.58), 57.14 (74.94), 43.18 (29.92), 41.11 (28.84).

### 3.2. Biological Evaluation

Detailed procedures for all biological expeiments (S3.2.), including anticancer screening (S3.2.1), flow cytometry analysis for cell cycle distribution of **3e**-trated A549 carcinoma cells (S3.2.2.), annexin-V-FITC assay for detection of apoptosis (S3.2.3.), quantitative reverse transcription-polymerase chain reaction (qRT-PCR) for analyzing the expression levels of apoptotic markers; *Bax*, *Bcl2*, *Caspase-9*, and *Caspase-3* genes in **3e**-treated A549 (S3.2.4.), In vitro anti-proliferative activities of compound **3e** against human normal lung fibroblast WI-38 cells (S3.2.5.), In vitro inhibition of TGF-βRI (ALK5) kinase assay (S3.2.6) are available in Supplementary Materials.

### 3.3. In Silico Studies

Detailed procedures for molecular docking studies (S3.3.1.), molecular dynamics (MD) simulations (S3.3.2.), and ADME predictions (S3.3.3.) are available in Supplementary Materials.

## 4. Conclusions

Fifteen new quinazolin-4(3*H*)-one derivatives were designed, synthesized, and evaluated against the NCI-60 human tumor cell panel. Compound **3e** emerged as the most active analogue, displaying GI_50_ values ranging from 2.63 to >100 µM (MID = 17.38 µM) and notable tumor-type selectivity (delta = 0.82, range = 1.58 log units). Enhanced sensitivity was observed in 31 cell lines (GI_50_ < 17.38 µM), of which A549 cells were selected for mechanistic investigations. In this model, compound **3e** (IC_50_ = 10.8 µM after 72 h) induced cell cycle perturbation characterized primarily by G2/M-phase arrest, together with apoptosis, as supported by increased *Bax*/*Bcl-2* ratio and upregulation of *caspase-9* and *caspase-3*. Transcriptomic profiling of the sensitive cell lines suggested TGF-βRI as a potential molecular target. Molecular docking showed favorable binding of compound **3e** within the TGF-βRI kinase domain through hydrogen-bonding and aromatic stacking interactions, while MD simulations demonstrated the stability of the ligand–receptor complex over a 100 ns trajectory. Consistent with these results, compound **3e** exhibited potent in vitro inhibition of TGF-βRI kinase activity (IC_50_ = 21.34 nM). Moreover, it showed limited cytotoxicity toward normal WI-38 fibroblasts (IC_50_ = 88.32 µM), resulting in favorable selectivity indices (SI = 5.13–33.58) across the sensitive cancer cell lines. ADME predictions suggested favorable oral absorption, although high plasma protein binding and potential CYP2C9 inhibition may require further optimization.

Collectively, these findings identify compound **3e** as a promising tumor-selective anticancer lead that induces cell cycle perturbation and apoptosis in A549 cells. Integrated target-identification, computational, and biochemical studies suggest TGF-βRI (ALK5) as a plausible molecular target; however, additional studies are warranted to further elucidate its mechanism of action.

## Figures and Tables

**Table 1:** Antiproliferative effects (G% < 50) of compounds **3a**–**3g**, **6d**, **8**, and **10b**.

Panel	Cell Line	3a	3b	3c	3d	3e	3f	3g	6d	8	10b
Leukemia	CCRF-CEM HL-60(TB) K-562 RPMI-8226 SR	-----^a^ -----^a^ -----^a^ -----^a^ -----^a^	-----^a^ -----^a^ -----^a^ -----^a^ -----^a^	-----^a^ -----^a^ -----^a^ -----^a^ -----^a^	-----^a^ -----^a^ -----^a^ -----^a^ -----^a^	-----^a^ -----^a^ 35.25 -----^a^ 40.43	-----^a^ -----^a^ -----^a^ -----^a^ -----^a^	-----^a^ -----^a^ -----^a^ -----^a^ -----^a^	49.53 -----^a^ -----^a^ 24.73 40.49	-----^a^ -----^a^ -----^a^ -----^a^ -----^a^	45.99 43.76 34.25 33.56 -----^a^
NSCLC	A549/ATCC EKVX HOP-62 HOP-92 NCI-H226 NCI-H23 NCI-H460 NCI-H522	-----^a^ -----^a^ 26.46 26.92 21.49 -----^a^ -----^a^ -----^a^	-----^a^ -----^a^ -----^a^ 44.61 37.97 -----^a^ -----^a^ -----^a^	-----^a^ -----^a^ -----^a^ -----^a^ -----^a^ -----^a^ -----^a^ -----^a^	-----^a^ -----^a^ -----^a^ -----^a^ -----^a^ -----^a^ -----^a^ -----^a^	16.78 24.84 −49.39 −6.91 17.38 −18.93 6.80 18.67	-----^a^ -----^a^ −17.14 −2.96 −21.89 46.04 22.42 -----^a^	-----^a^ -----^a^ -----^a^ -----^a^ -----^a^ -----^a^ -----^a^ -----^a^	-----^a^ -----^a^ -----^a^ -----^a^ -----^a^ -----^a^ -----^a^ -----^a^	-----^a^ -----^a^ -----^a^ -----^a^ -----^a^ -----^a^ -----^a^ -----^a^	-----^a^ -----^a^ -----^a^ -----^a^ -----^a^ -----^a^ -----^a^ -----^a^
Colon	COLO 205 HCC-2998 HCT-116 HT29 SW-620	-----^a^ -----^a^ 44.79 -----^a^ -----^a^	-----^a^ -----^a^ -----^a^ -----^a^ -----^a^	-----^a^ -----^a^ -----^a^ -----^a^ -----^a^	-----^a^ -----^a^ -----^a^ -----^a^ -----^a^	9.31 -----^a^ 10.28 8.82 44.47	-----^a^ -----^a^ 24.43 49.94 -----^a^	-----^a^ -----^a^ -----^a^ -----^a^ -----^a^	-----^a^ -----^a^ -----^a^ -----^a^ -----^a^	-----^a^ 38.61 -----^a^ -----^a^ -----^a^	-----^a^ -----^a^ -----^a^ -----^a^ -----^a^
CNS	SF-268 SF-295 SF-539 SNB-19 SNB-75 U251	-----^a^ -----^a^ 25.74 7.05 −20.71 48.82	-----^a^ -----^a^ -----^a^ -----^a^ -----^a^ -----^a^	-----^a^ -----^a^ -----^a^ 33.27 21.47 -----^a^	-----^a^ -----^a^ -----^a^ 45.71 41.65 -----^a^	-----^a^ 11.41 −16.23 33.90 -----^a^ −34.75	28.90 4.69 2.03 ND ^b^ −19.41 19.60	-----^a^ -----^a^ -----^a^ -----^a^ 45.03 -----^a^	-----^a^ -----^a^ -----^a^ -----^a^ -----^a^ -----^a^	-----^a^ -----^a^ -----^a^ -----^a^ -----^a^ -----^a^	-----^a^ -----^a^ -----^a^ -----^a^ -----^a^ -----^a^
MEL ^c^	LOX IMVI MALME-3M M14 SK-MEL-28 UACC-62	-----^a^ -----^a^ -----^a^ -----^a^ -----^a^	-----^a^ -----^a^ -----^a^ -----^a^ -----^a^	-----^a^ -----^a^ -----^a^ -----^a^ -----^a^	-----^a^ -----^a^ -----^a^ -----^a^ -----^a^	20.77 42.35 29.41 27.84 3.64	-----^a^ 39.64 -----^a^ -----^a^ -----^a^	-----^a^ -----^a^ -----^a^ -----^a^ -----^a^	48.44 -----^a^ -----^a^ -----^a^ -----^a^	-----^a^ -----^a^ -----^a^ -----^a^ -----^a^	-----^a^ -----^a^ -----^a^ -----^a^ -----^a^
Ovarian	IGROV1 OVCAR-4 OVCAR-8 NCI/ADR-RES SK-OV-3	-----^a^ -----^a^ 32.45 -----^a^ -----^a^	-----^a^ -----^a^ -----^a^ -----^a^ -----^a^	-----^a^ -----^a^ -----^a^ -----^a^ -----^a^	-----^a^ -----^a^ -----^a^ -----^a^ -----^a^	49.86 21.72 0.01 3.80 -----^a^	-----^a^ 30.97 37.76 28.27 45.95	-----^a^ -----^a^ -----^a^ -----^a^ -----^a^	-----^a^ -----^a^ -----^a^ -----^a^ -----^a^	-----^a^ -----^a^ -----^a^ -----^a^ -----^a^	-----^a^ -----^a^ -----^a^ -----^a^ -----^a^
Renal	786-0 ACHN CAKI-1 RXF 393 SN12C TK-10 UO-31	34.72 37.79 −21.56 18.82 -----^a^ -----^a^ 39.65	-----^a^ -----^a^ -----^a^ -----^a^ -----^a^ -----^a^ -----^a^	-----^a^ 47.53 -----^a^ -----^a^ -----^a^ -----^a^ -----^a^	48.97 -----^a^ -----^a^ -----^a^ -----^a^ -----^a^ -----^a^	36.31 5.25 -----^a^ −17.74 -----^a^ 7.44 -----^b^	10.73 33.34 10.14 −10.65 -----^a^ -----^a^ -----^a^	-----^a^ -----^a^ -----^a^ -----^a^ -----^a^ -----^a^ -----^a^	-----^a^ -----^a^ -----^a^ -----^a^ 42.91 -----^a^ -----^a^	-----^a^ -----^a^ -----^a^ -----^a^ -----^a^ -----^a^ -----^a^	-----^a^ -----^a^ -----^a^ -----^a^ -----^a^ -----^a^ -----^a^
Prostate	PC-3	-----^a^	-----^a^	-----^a^	-----^a^	46.81	25.92	-----^a^	-----^a^	-----^a^	-----^a^
Breast	MCF7 MDA-MB-231/ ^d^ HS 578T BT-549 T-47D MDA-MB-468	-----^a^ -----^a^ 25.70 31.98 -----^a^ -----^a^	-----^a^ -----^a^ 47.99 -----^a^ -----^a^ -----^a^	-----^a^ -----^a^ 34.47 -----^a^ -----^a^ -----^a^	-----^a^ -----^a^ 40.73 -----^a^ -----^a^ -----^a^	39.25 -----^b^ 15.05 23.97 20.71 43.86	-----^a^ 13.66 −18.8911.44 -----^a^ 44.71	-----^a^ -----^a^ 45.61 -----^a^ -----^a^ -----^a^	-----^a^ -----^a^ -----^a^ -----^a^ -----^a^ -----^a^	-----^a^ -----^a^ -----^a^ -----^a^ -----^a^ -----^a^	-----^a^ -----^a^ -----^a^ -----^a^ -----^a^ -----^a^

^a^: Only G% values < 50 are shown; ----- indicates that the compound did not produce a G% value below 50 in the corresponding cell line; ND ^b^: Not determined; MEL ^c^: denotes Melanoma; ^d^: ATCC.

**Table 2:** Cell lines most sensitive to compound **3e**, showing GI_50_ values values below the mean graph midpoint (MG-MID; 17.38 µM, log_10_ −4.76) together with their selectivity indices (SI) and TGF-βRI expression Z-scores.

Panel	Cell Line	GI_50_ (µM)/SI ^a^	TGF-βRI Expression Z-Scores ^d^
Leukemia	K-562	6.44/13.71	−7.847
	SR	3.54/24.95	−3.398
NSCLC	A549/ATCC	10.8/8.18	4.689
	EKVX	7.41/11.92	8.267
	HOP-62	14.7/6.01	10.138
	HOP-92	16.2/5.45	3.760
	NCI-H23	3.18/27.77	2.793
	NCI-H460	6.40/13.8	−7.024
Colon Cancer	HCT-116	4.22/20.93	−2.242
	HCT-15	10.8/8.18	−10.638
CNS Cancer	SF-295	6.01/14.70	10.589
	SF-539	2.63/33.58	14.025
	SNB-75	17.1/5.17	12.106
	U251	11.5/7.68	11.153
Melanoma	LOX IMVI	9.69/9.11	4.839
	UACC-62	10.1/8.75	3.650
Ovarian Cancer	IGROV1	7.64/11.56	−2.956
	OVCAR-4	16.1/5.49	5.759
	OVCAR-5	13.5/6.54	−8.923
	OVCAR-8	7.84/11.27	8.821
	NCI/ADR-RES	6.17/14.31	Not listed in the HPA
Renal Cancer	ACHN	13.2/6.69	5.522
	CAKI-1	15.6/5.66	3.870
	RXF 393	16.8/5.26	Not listed in the HPA
	SN12C	15.7/5.63	Not listed in the HPA
	TK-10	16.4/5.39	2.727
	UO-31	5.89/15.00	10.287
Breast Cancer	MCF7	15.6/5.66	−3.98
	MDA-MB-231/ATCC	13.3/6.64	2.487
	HS 578T	17.2/5.13	15.326
	BT-549	13.3/6.64	2.818
MID for GI_50_	17.38 µM		
Delta ^b^ for GI_50_	0.82 log unit		
Range ^c^ for GI_50_	1.58 log unit		

SI ^a^: The selectivity index (SI) was calculated as the ratio of the IC_50_ value in normal human lung fibroblast WI-38 cells (88.32 µM) to the IC_50_ value in the respective cancer cell line; Delta ^b^: indicates that the most responsive cell line differs from the mean inhibitory dose (MG-MID) by about 6.63-fold. Range ^c^: reflects approximately a 38-fold variation in sensitivity across the tested cell lines; TGF-βRI expression Z-scores ^d^: standardized measures of TGF-βRI expression levels in various cell lines retrieved from the Human Protein Atlas (HPA); positive Z-scores indicate above-average expression, whereas negative Z-scores indicate below-average expression.

**Table 3:** Cell cycle distribution of A549 cells after treatment with compound **3e** (10.8 µM, 72 h) compared with untreated control cells.

Sample	Cell Cycle Distribution (%)			
	Sub-G1	G1	S	G2/M
A549	4.13	57.17	11.74	26.96
**3e**/A549	17.06	10.03	13.06	59.58

**Table 4:** Flow cytometric analysis of apoptosis and necrosis in A549 cells following treatment with compound **3e** (10.8 µM, 72 h), as assessed by Annexin V-FITC/PI dual staining.

Sample	Viable % (Q3)	Apoptotic Cells %		Necrosis % (Q1)
		Early (Q4)	Late (Q2)	
A549	98.66	0.11	0.19	1.04
**3e**/A549	49.71	43.94	2.99	3.36

**Table 5:** Relative expression of mRNA of *Bax*, *Bcl-2*, *caspase-3*, and *caspase-9* genes in A549 cells treated with **3e** for 72 h at its IC_50_ concentration (10.8 µM) compared to negative control (A549).

Sample	Gene Expression (Fold Change) ^a^				
	*Bax*	*Bcl-2*	*Bax*/*Bcl-2* Ratio	*Caspase-9*	*Caspase-3*
A549	1.00 ± 0.11	1.00 ± 0.03	1.00 ± 0.11	1.00 ± 0.05	1.00 ± 0.10
**3e**/A549	4.83 ± 0.86 *	0.60 ± 0.02 ***	7.91 ± 1.36 **	2.51 ± 0.32 **	4.56 ± 0.66 ***

^a^ Values are given as changes from the corresponding control (A549) group, which is set to ‘1’; * *p* < 0.05, ** *p* < 0.01 and *** *p* < 0.001 indicate statistically significant differences from the corresponding control in unpaired *t*-tests.

**Table 6:** Docking scores and binding interactions of Co–crystal ligand (RepSox) and compound **3e** within the active site of TGF-ꞵ type I receptor kinase domain.

Compound	Docking Score (Kcal/Mol)	Binding Interactions	
		Hydrogen Bonds	Hydrophobic and Other Interactions
**RepSox** **(E-616452)**	−10.3	Lys232 His283 Asp351	Ile211, Val219, Ala230, Tyr249, Leu260, Phe262, Leu278, Leu340, Ala350
**3e**	−9.5	Lys232 His283	Ile211, Gly212, Lys213, Gly214, Val219, Ala230, Tyr249, Leu260, Leu278, Val279, Ser280, Tyr282, Gly286, Ser287, Leu340, Ala350, Asp351

**Table 7:** In silico predictions of ADME-related parameters for the most active compound **3e**.

ADME Parameter	Predicted Value
HIA	98
Caco2	43
MDCK	0.02
BBB	0.46
PPB	100
CYP2C19 inhibition	Non
CYP2C9 inhibition	Inhibitor
CYP2D6 inhibition	Non
CYP2D6 substrate	Non
CYP3A4 inhibition	Non
CYP3A4 substrate	Substrate
Pgp inhibition	Inhibitor

## Data Availability

The original contributions presented in this study are included in the article/Supplementary Materials. Further inquiries can be directed to the corresponding author.

## Supplementary Materials

The following supporting information can be downloaded at https://www.mdpi.com/article/10.3390/ph19070996/s1. Figure S1: The activity of cancer-related pathways inferred using PROGENy and CytoSig cytokines (from the Human Protein Atlas) for the most responsive human cancer cell lines having GI_50_ values below the MID (17.38 µM); Figure S2: (A) 3D representation of the superimposition of docking pose (cyan) and the co-crystallized pose (mulberry) of RepSox (E-616452) in the TGF-β type I receptor active site. (B) 2D interaction diagrams showing RepSox docking pose interactions with the key amino acids in the TGF-β type I receptor active site (PDB ID: 1VJY); Figure S3: (**A**) 3D and (**B**) 2D interaction diagrams showing compound **3e** docking pose within the TGF-ꞵ type I receptor kinase domain (PDB ID: 1VJY); Figure S4: Graph of log(concentration) of compound **3e** versus the percentage viability of normal human lung fibroblast WI-38 cells; Table S1: Energy minimization calculations for *E* and *Z*-isomers of compounds **6a**–**6d** and **8**; Table S2: Analysis of mean graph data for compounds **1a**,**b**; **3a**–**h**; **6a**–**d**; **8**; and **10a**,**b** as obtained from the NCI-60 one-dose cell line screen (at 10 µM); Table S3: The sequences for primers used in quantitative real-time qRT-PCR; S3.2. Biological evaluation; S3.2.1. Anticancer screening; S3.2.2. Flow cytometry analysis for cell cycle distribution of **3e**-treated A549 carcinoma cells; S3.2.3. Annexin-V-FITC assay for detection of apoptosis; S3.2.4. Quantitative reverse transcription-polymerase chain reaction (qRT-PCR) for analyzing the expression levels of apoptotic markers; *Bax*, *Bcl-2*, *Caspase-9*, and *Caspase-3* genes in **3e**-treated A549 cells; S3.2.5. In vitro anti-proliferative activities of compound **3e** against normal human lung fibroblast WI-38 cells; S3.2.6. In vitro TGF-βRI (ALK5) kinase inhibition assay; S3.3. In silico studies; S3.3.1. Molecular docking studies; S3.3.2. Molecular dynamics (MD) simulations; S3.3.3. ADME prediction; and Spectroscopic data of selected representative compounds [60,61,62,63,64,65,66,67,68,69,70,71,72,73,74,75,76,77,78,79,80].

## Appendix B

The complete five-component screening data package for compound **3e** includes.

**Table A1:** Primary data sheet showing percent growth values at five test concentrations and the corresponding GI_50_, TGI, and LC_50_ values across the NCI-60 cancer cell line panel.
