# Supplementary material for: Synthesis and Anticancer Activity of New Quinazolin-4(3H)-one Derivatives: Identification of a Tumor-Selective Anticancer Agent with Potential Inhibition of TGF-βRI (ALK5)
Source: Pharmaceuticals (Basel). 2026 Jun 26;19(7):996. doi: 10.3390/ph19070996 (PMC13416033; doi:10.3390/ph19070996)
Supplement: Supplementary file 1 [file pharmaceuticals-19-00996-s001.zip › Proofread Supplementary Materials/Appendix B.pdf]

National Cancer Institute Developmental Therapeutics Program
In-Vitro Screening Data Review Checklist

**NSC:** D - 838963 / 1	**Experiment ID:** 2212NS79
**Test Date:** December 12, 2022	**Review Date:** January 13, 2023

Pending Action by the NCI for this experiment

1. __X__ None
2. ______ Repeat testing in the Primary Screen
3. ______ Refer to Biological Evaluation Committee
4. ______ Currently under Review by Biological Evaluation Committee

National Cancer Institute Developmental Therapeutics Program
In-Vitro Testing Results

NSC : D - 838963 / 1	Experiment ID : 2212NS79	Test Type : 08	Units : Molar
Report Date : December 11, 2023	Test Date : December 12, 2022	QNS :	MC :
COMI : Q34	Stain Reagent : SRB Dual-Pass Related	SSPL : 1CJS	

			Log10 Concentration												
	Time		Mean Optical Densities					Percent Growth							
Panel/Cell Line	Zero	Ctrl	-8.0	-7.0	-6.0	-5.0	-4.0	-8.0	-7.0	-6.0	-5.0	-4.0	GI50	TGI	LC50
**Leukemia**															
CCRF-CEM	0.822	2.910	2.886	2.943	2.908	2.496	1.667	99	102	100	80	40	5.74E-5	> 1.00E-4	> 1.00E-4
HL-60(TB)	0.590	2.654	2.475	2.400	2.347	2.034	1.887	91	88	85	70	63	> 1.00E-4	> 1.00E-4	> 1.00E-4
K-562	0.212	1.854	1.840	1.842	1.794	0.853	0.625	99	99	96	39	25	6.44E-6	> 1.00E-4	> 1.00E-4
MOLT-4	0.458	2.226	2.222	2.273	2.169	2.243	0.664	100	103	97	101	12	3.72E-5	> 1.00E-4	> 1.00E-4
RPMI-8226	0.816	2.676	2.735	2.732	2.701	2.472	1.613	103	103	101	89	43	7.00E-5	> 1.00E-4	> 1.00E-4
SR	0.290	1.328	1.356	1.301	1.184	0.502	0.387	103	97	86	20	9	3.54E-6	> 1.00E-4	> 1.00E-4
**Non-Small Cell Lung Cancer**															
A549/ATCC	0.378	2.279	2.188	2.167	2.111	1.360	0.467	95	94	91	52	5	1.08E-5	> 1.00E-4	> 1.00E-4
EKVX	0.547	1.527	1.416	1.451	1.351	0.990	0.526	89	92	82	45	-4	7.41E-6	8.32E-5	> 1.00E-4
HOP-62	0.610	2.203	2.141	2.047	2.021	1.706	0.343	96	90	89	69	-44	1.47E-5	4.08E-5	> 1.00E-4
HOP-92	1.317	2.101	2.013	1.997	2.013	1.871	0.951	89	87	89	71	-28	1.62E-5	5.22E-5	> 1.00E-4
NCI-H226	1.106	3.031	2.973	2.941	2.927	2.408	1.193	97	95	95	68	5	1.90E-5	> 1.00E-4	> 1.00E-4
NCI-H23	0.670	1.745	1.764	1.718	1.700	0.720	0.319	102	98	96	5	-52	3.18E-6	1.21E-5	9.05E-5
NCI-H322M	0.815	2.220	2.124	2.087	2.036	1.885	1.224	93	91	87	76	29	3.59E-5	> 1.00E-4	> 1.00E-4
NCI-H460	0.449	3.247	3.270	3.267	3.265	1.508	0.390	101	101	101	38	-13	6.40E-6	5.50E-5	> 1.00E-4
NCI-H522	1.515	3.106	3.002	3.112	3.038	2.614	2.137	93	100	96	69	39	4.31E-5	> 1.00E-4	> 1.00E-4
**Colon Cancer**															
COLO 205	0.544	1.849	1.892	1.934	1.906	1.539	0.797	103	107	104	76	19	2.89E-5	> 1.00E-4	> 1.00E-4
HCC-2998	0.789	2.183	2.185	2.040	2.088	1.948	1.320	100	90	93	83	38	5.44E-5	> 1.00E-4	> 1.00E-4
HCT-116	0.284	2.481	2.518	2.504	2.471	0.730	0.241	102	101	100	20	-15	4.22E-6	3.74E-5	> 1.00E-4
HCT-15	0.269	1.677	1.604	1.562	1.570	0.987	0.577	95	92	92	51	22	1.08E-5	> 1.00E-4	> 1.00E-4
HT29	0.348	2.283	2.294	2.205	2.093	1.587	0.480	101	96	90	64	7	1.76E-5	> 1.00E-4	> 1.00E-4
KM12	0.767	3.176	3.242	3.170	3.176	3.123	2.749	103	100	100	98	82	> 1.00E-4	> 1.00E-4	> 1.00E-4
SW-620	0.373	2.374	2.348	2.314	2.311	1.624	0.758	99	97	97	63	19	1.95E-5	> 1.00E-4	> 1.00E-4
**CNS Cancer**															
SF-268	0.989	2.817	2.706	2.757	2.714	2.339	1.466	94	97	94	74	26	3.16E-5	> 1.00E-4	> 1.00E-4
SF-295	0.813	2.703	2.611	2.573	2.558	1.531	0.618	95	93	92	38	-24	6.01E-6	4.10E-5	> 1.00E-4
SF-539	0.710	2.193	2.199	2.134	2.142	0.608	0.108	100	96	97	-14	-85	2.63E-6	7.41E-6	3.20E-5
SNB-19	0.750	2.415	2.291	2.268	2.259	1.979	0.691	93	91	91	74	-8	1.96E-5	8.01E-5	> 1.00E-4
SNB-75	1.426	2.388	2.149	2.115	2.132	2.104	1.173	75	72	73	70	-18	1.71E-5	6.29E-5	> 1.00E-4
U251	0.494	2.283	2.203	2.233	2.172	1.485	0.332	96	97	94	55	-33	1.15E-5	4.24E-5	> 1.00E-4
**Melanoma**															
LOX IMVI	0.433	2.354	2.275	2.227	2.230	1.382	0.383	96	93	94	49	-12	9.69E-6	6.46E-5	> 1.00E-4
MALME-3M	0.802	1.430	1.466	1.368	1.366	1.301	0.569	106	90	90	79	-29	1.87E-5	5.39E-5	> 1.00E-4
M14	0.487	2.035	1.974	1.958	1.969	1.691	0.793	96	95	96	78	20	3.01E-5	> 1.00E-4	> 1.00E-4
MDA-MB-435	0.696	2.892	2.855	2.833	2.859	2.508	1.532	98	97	98	83	38	5.39E-5	> 1.00E-4	> 1.00E-4
SK-MEL-2	1.291	2.241	2.255	2.308	2.290	2.125	1.403	102	107	105	88	12	3.14E-5	> 1.00E-4	> 1.00E-4
SK-MEL-28	0.779	2.566	2.574	2.523	2.495	2.084	0.648	100	98	96	73	-17	1.80E-5	6.50E-5	> 1.00E-4
SK-MEL-5	0.864	3.304	3.274	3.235	3.224	3.184	1.577	99	97	97	95	29	4.83E-5	> 1.00E-4	> 1.00E-4
UACC-257	0.946	2.460	2.362	2.339	2.321	2.201	1.246	94	92	91	83	20	3.32E-5	> 1.00E-4	> 1.00E-4
UACC-62	0.889	3.064	3.002	2.945	2.930	1.982	0.598	97	95	94	50	-33	1.01E-5	4.03E-5	> 1.00E-4
**Ovarian Cancer**															
IGROV1	0.539	2.333	2.309	2.202	2.222	1.332	0.379	99	93	94	44	-30	7.64E-6	3.96E-5	> 1.00E-4
OVCAR-3	0.644	2.150	2.147	2.155	2.253	1.796	1.039	100	100	107	77	26	3.37E-5	> 1.00E-4	> 1.00E-4
OVCAR-4	0.981	2.156	2.125	2.097	2.100	1.715	1.011	97	95	95	62	3	1.61E-5	> 1.00E-4	> 1.00E-4
OVCAR-5	0.479	1.733	1.610	1.504	1.504	1.205	0.471	90	82	82	58	-2	1.35E-5	9.34E-5	> 1.00E-4
OVCAR-8	0.441	2.284	2.218	2.301	2.258	1.257	0.453	96	101	99	44	1	7.84E-6	> 1.00E-4	> 1.00E-4
NCI/ADR-RES	0.567	1.631	1.624	1.610	1.570	0.974	0.215	99	98	94	38	-62	6.17E-6	2.40E-5	7.57E-5
SK-OV-3	1.065	2.259	2.244	2.247	2.204	1.999	1.172	99	99	95	78	9	2.55E-5	> 1.00E-4	> 1.00E-4
**Renal Cancer**															
786-0	0.907	2.793	2.680	2.705	2.729	2.834	0.806	94	95	97	102	-11	2.89E-5	7.97E-5	> 1.00E-4
A498	1.395	2.339	2.299	2.348	2.234	2.319	1.543	96	101	89	98	16	3.82E-5	> 1.00E-4	> 1.00E-4
ACHN	0.297	1.296	1.264	1.291	1.297	0.968	0.077	97	99	100	67	-74	1.32E-5	2.98E-5	6.74E-5
CAKI-1	1.340	2.936	2.776	2.783	2.783	2.508	0.709	90	90	90	73	-47	1.56E-5	4.06E-5	> 1.00E-4
RXF 393	0.915	1.661	1.622	1.523	1.545	1.436	0.749	95	82	84	70	-18	1.68E-5	6.21E-5	> 1.00E-4
SN12C	0.704	2.738	2.691	2.664	2.651	1.904	0.977	98	96	96	59	13	1.57E-5	> 1.00E-4	> 1.00E-4
TK-10	0.942	2.137	2.081	2.071	2.053	1.827	0.588	95	94	93	74	-38	1.64E-5	4.60E-5	> 1.00E-4
UO-31	0.570	2.146	1.977	1.926	1.910	1.194	0.310	89	86	85	40	-46	5.89E-6	2.91E-5	> 1.00E-4
**Prostate Cancer**															
PC-3	0.597	2.473	2.446	2.396	2.468	1.961	0.984	99	96	100	73	21	2.73E-5	> 1.00E-4	> 1.00E-4
DU-145	0.446	1.878	1.963	1.857	1.900	1.614	0.623	106	99	102	82	12	2.86E-5	> 1.00E-4	> 1.00E-4
**Breast Cancer**															
MCF7	0.299	1.544	1.493	1.428	1.415	1.077	0.293	96	91	90	62	-2	1.56E-5	9.31E-5	> 1.00E-4
MDA-MB-231/ATCC	0.639	1.281	1.284	1.276	1.266	1.048	0.345	100	99	98	64	-46	1.33E-5	3.80E-5	> 1.00E-4
HS 578T	1.227	2.320	2.281	2.257	2.223	1.958	1.166	96	94	91	67	-5	1.72E-5	8.52E-5	> 1.00E-4
BT-549	1.173	2.306	2.296	2.308	2.281	1.844	0.988	99	100	98	59	-16	1.33E-5	6.16E-5	> 1.00E-4
T-47D	1.085	2.860	2.806	2.746	2.722	2.630	1.485	97	94	92	87	23	3.75E-5	> 1.00E-4	> 1.00E-4

National Cancer Institute Developmental Therapeutics Program<br>Dose Response Curves	NSC: D - 838963 / 1	SSPL: 1CJS	EXP. ID: 2212NS79
	Report Date: December 11, 2023	Test Date: December 12, 2022	

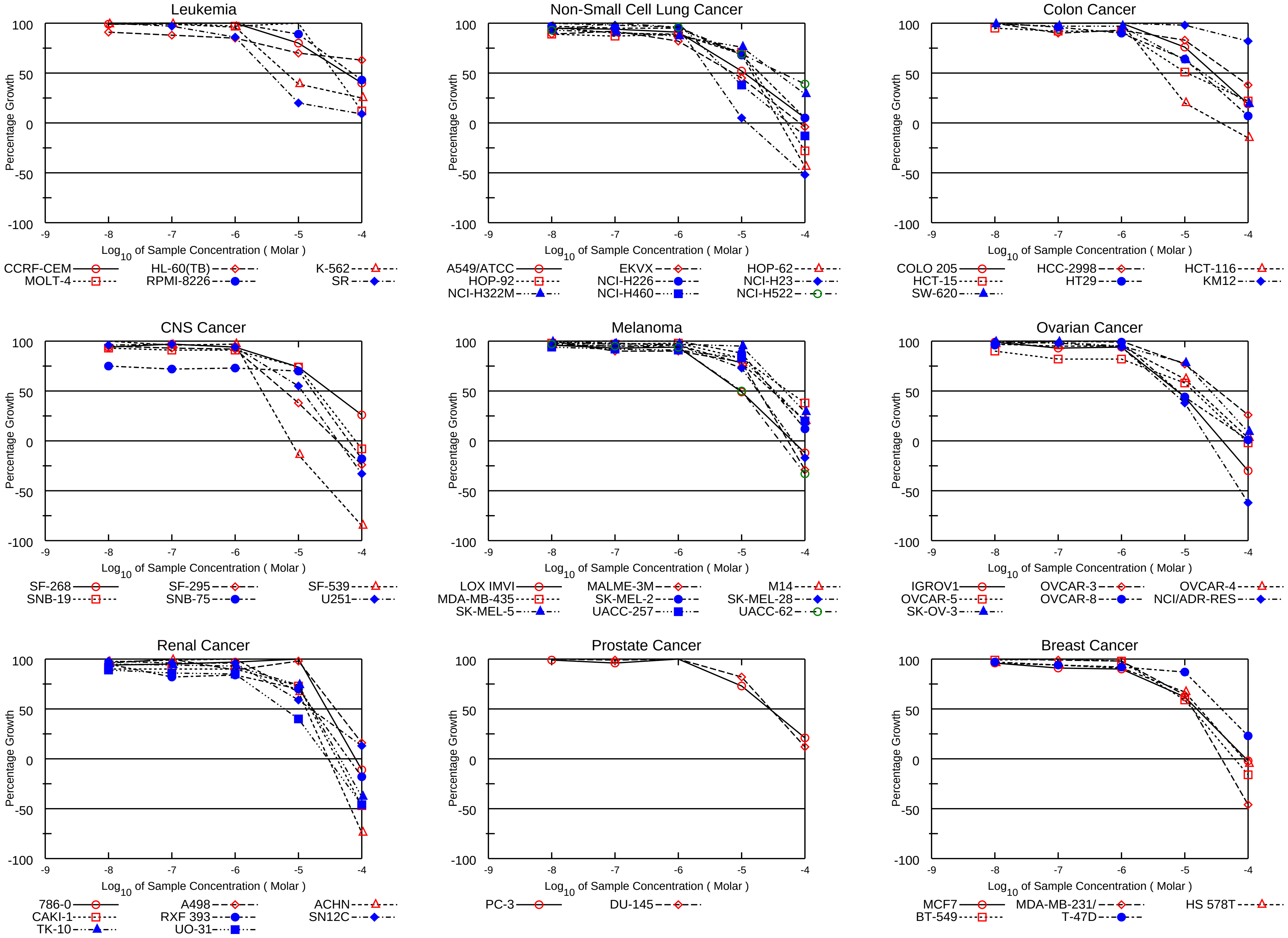

National Cancer Institute Developmental Therapeutics Program Mean Graphs	NSC : D - 838963/1	Units :Molar	SSPL :1CJS	EXP. ID :2212NS79
	Report Date :December 11, 2023		Test Date :December 12, 2022	

Panel/Cell Line	Log_{10}GI50	Log_{10}TGI	Log_{10}LC50
**Leukemia**			
CCRF-CEM	-4.24	> -4.00	> -4.00
HL-60(TB)	> -4.00	> -4.00	> -4.00
K-562	-5.19	> -4.00	> -4.00
MOLT-4	-4.43	> -4.00	> -4.00
RPMI-8226	-4.15	> -4.00	> -4.00
SR	-5.45	> -4.00	> -4.00
**Non-Small Cell Lung Cancer**			
A549/ATCC	-4.96	> -4.00	> -4.00
EKVX	-5.13	-4.08	> -4.00
HOP-62	-4.83	-4.39	> -4.00
HOP-92	-4.79	-4.28	> -4.00
NCI-H226	-4.72	> -4.00	> -4.00
NCI-H23	-5.50	-4.92	-4.04
NCI-H322M	-4.44	> -4.00	> -4.00
NCI-H460	-5.19	-4.26	> -4.00
NCI-H522	-4.37	> -4.00	> -4.00
**Colon Cancer**			
COLO 205	-4.54	> -4.00	> -4.00
HCC-2998	-4.26	> -4.00	> -4.00
HCT-116	-5.37	-4.43	> -4.00
HCT-15	-4.97	> -4.00	> -4.00
HT29	-4.75	> -4.00	> -4.00
KM12	> -4.00	> -4.00	> -4.00
SW-620	-4.71	> -4.00	> -4.00
**CNS Cancer**			
SF-268	-4.50	> -4.00	> -4.00
SF-295	-5.22	-4.39	> -4.00
SF-539	-5.58	-5.13	-4.49
SNB-19	-4.71	-4.10	> -4.00
SNB-75	-4.77	-4.20	> -4.00
U251	-4.94	-4.37	> -4.00
**Melanoma**			
LOX IMVI	-5.01	-4.19	> -4.00
MALME-3M	-4.73	-4.27	> -4.00
M14	-4.52	> -4.00	> -4.00
MDA-MB-435	-4.27	> -4.00	> -4.00
SK-MEL-2	-4.50	> -4.00	> -4.00
SK-MEL-28	-4.74	-4.19	> -4.00
SK-MEL-5	-4.32	> -4.00	> -4.00
UACC-257	-4.48	> -4.00	> -4.00
UACC-62	-5.00	-4.39	> -4.00
**Ovarian Cancer**			
IGROV1	-5.12	-4.40	> -4.00
OVCAR-3	-4.47	> -4.00	> -4.00
OVCAR-4	-4.79	> -4.00	> -4.00
OVCAR-5	-4.87	-4.03	> -4.00
OVCAR-8	-5.11	> -4.00	> -4.00
NCI/ADR-RES	-5.21	-4.62	-4.12
SK-OV-3	-4.59	> -4.00	> -4.00
**Renal Cancer**			
786-0	-4.54	-4.10	> -4.00
A498	-4.42	> -4.00	> -4.00
ACHN	-4.88	-4.53	-4.17
CAKI-1	-4.81	-4.39	> -4.00
RXF 393	-4.77	-4.21	> -4.00
SN12C	-4.80	> -4.00	> -4.00
TK-10	-4.78	-4.34	> -4.00
UO-31	-5.23	-4.54	> -4.00
**Prostate Cancer**			
PC-3	-4.56	> -4.00	> -4.00
DU-145	-4.54	> -4.00	> -4.00
**Breast Cancer**			
MCF7	-4.81	-4.03	> -4.00
MDA-MB-231/ATCC	-4.88	-4.42	> -4.00
HS 578T	-4.77	-4.07	> -4.00
BT-549	-4.88	-4.21	> -4.00
T-47D	-4.43	> -4.00	> -4.00
_MID	-4.76	-4.16	-4.01
Delta	0.82	0.97	0.48
Range	1.58	1.13	0.49

GI50

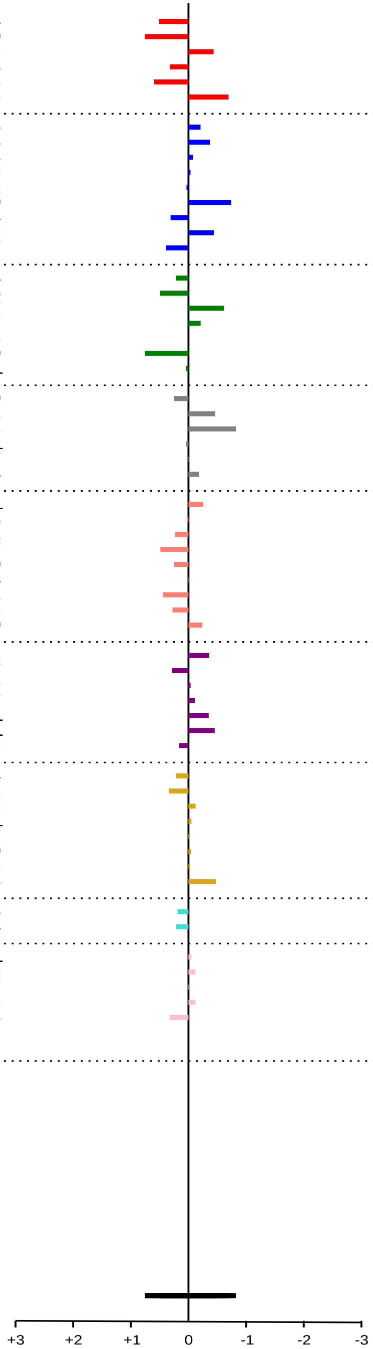

TGI

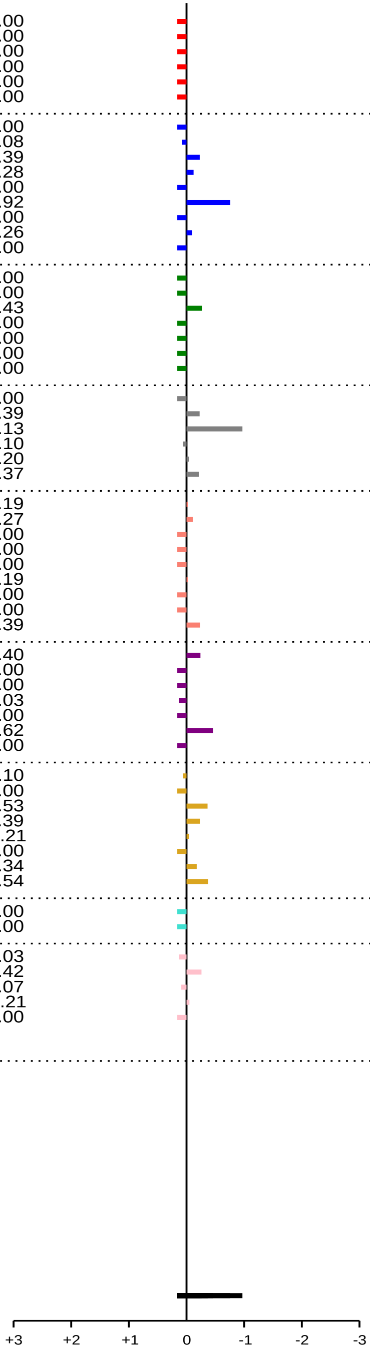

LC50

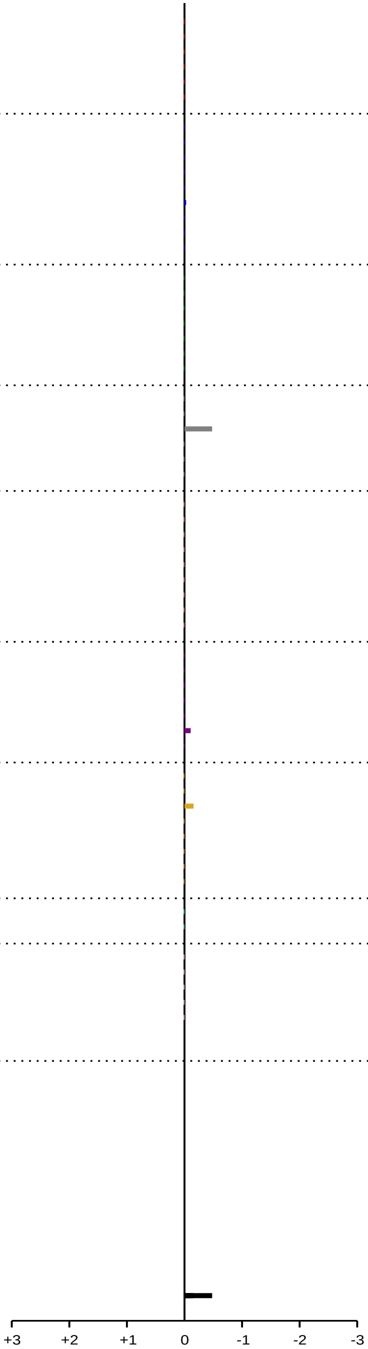

National Cancer Institute Developmental Therapeutics Program Dose Response Curves	NSC : D - 838963/1	SSPL:1CJS	EXP. ID:2212NS79
	Report Date:December 11, 2023	Test Date:December 12, 2022	

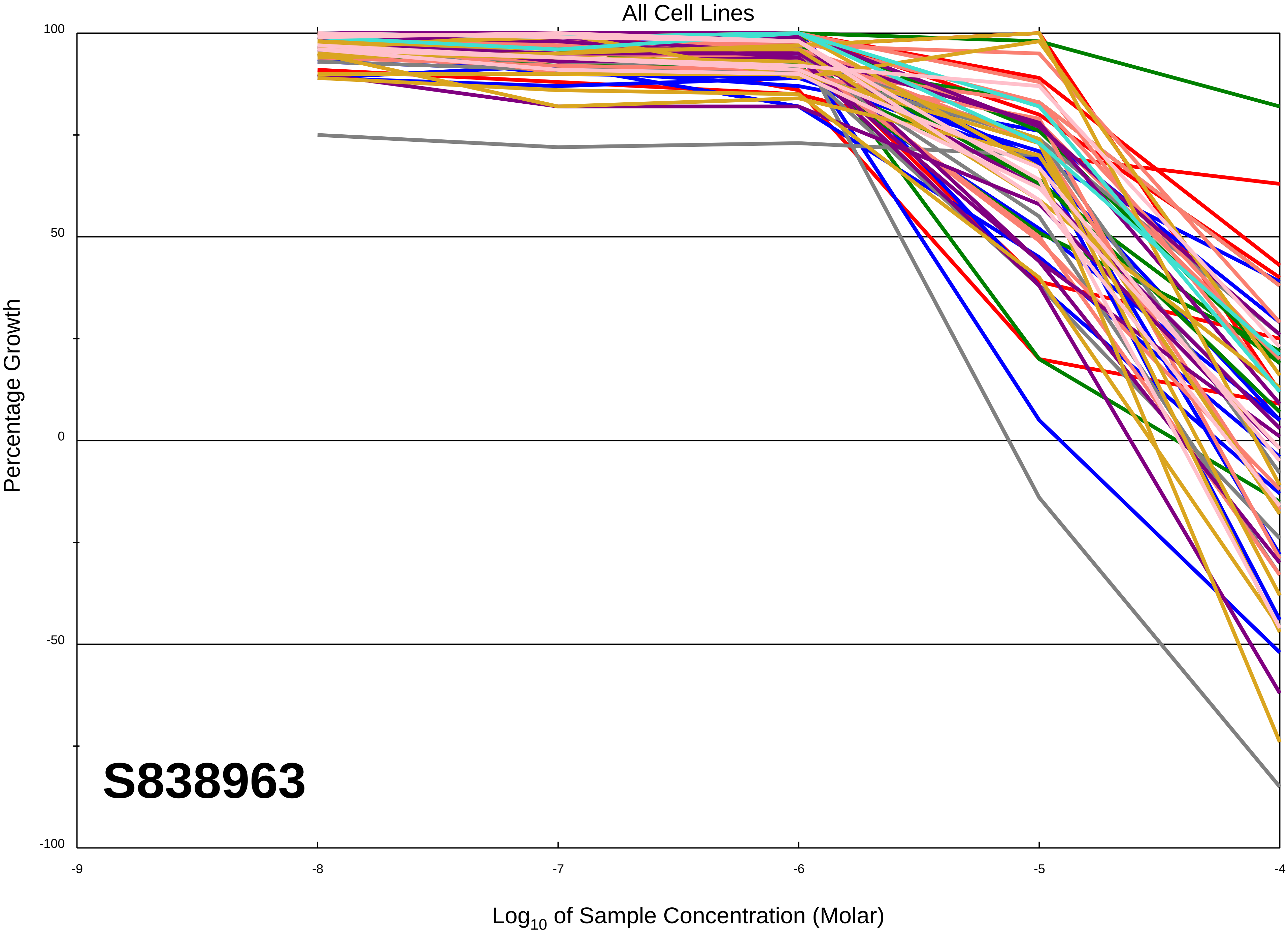

National Cancer Institute Developmental Therapeutics Program<br>Waterfall Graph GI50	NSC : D - 838963/1	Units :Molar	SSPL :1CJS	EXP. ID :2212NS79
	Report Date :December 11, 2023		Test Date :December 12, 2022	

Panel	Cell Name	Hollow Fiber	GI50
CNS Cancer	SF-539		-5.58
Non-Small Cell Lung Cancer	NCI-H23	*	-5.50
Leukemia	SR		-5.45
Colon Cancer	HCT-116		-5.37
Renal Cancer	UO-31		-5.23
CNS Cancer	SF-295	*	-5.22
Ovarian Cancer	NCI/ADR-RES		-5.21
Non-Small Cell Lung Cancer	NCI-H460		-5.19
Leukemia	K-562		-5.19
Non-Small Cell Lung Cancer	EKVX		-5.13
Ovarian Cancer	IGROV1		-5.12
Ovarian Cancer	OVCAR-8		-5.11
Melanoma	LOX IMVI	*	-5.01
Melanoma	UACC-62	*	-5.00
Colon Cancer	HCT-15		-4.97
Non-Small Cell Lung Cancer	A549/ATCC		-4.96
CNS Cancer	U251	*	-4.94
Renal Cancer	ACHN		-4.88
Breast Cancer	BT-549		-4.88
Breast Cancer	MDA-MB-231/ATCC	*	-4.88
Ovarian Cancer	OVCAR-5	*	-4.87
Non-Small Cell Lung Cancer	HOP-62		-4.83
Renal Cancer	CAKI-1		-4.81
Breast Cancer	MCF7		-4.81
Renal Cancer	SN12C		-4.80
Ovarian Cancer	OVCAR-4		-4.79
Non-Small Cell Lung Cancer	HOP-92		-4.79
Renal Cancer	TK-10		-4.78
Renal Cancer	RXF 393		-4.77
CNS Cancer	SNB-75		-4.77
Breast Cancer	HS 578T		-4.77
Colon Cancer	HT29		-4.75
Melanoma	SK-MEL-28		-4.74
Melanoma	MALME-3M		-4.73
Non-Small Cell Lung Cancer	NCI-H226		-4.72
Colon Cancer	SW-620	*	-4.71
CNS Cancer	SNB-19		-4.71
Ovarian Cancer	SK-OV-3		-4.59
Prostate Cancer	PC-3		-4.56
Prostate Cancer	DU-145		-4.54
Renal Cancer	786-0		-4.54
Colon Cancer	COLO 205	*	-4.54
Melanoma	M14		-4.52
Melanoma	SK-MEL-2		-4.50
CNS Cancer	SF-268		-4.50
Melanoma	UACC-257		-4.48
Ovarian Cancer	OVCAR-3	*	-4.47
Non-Small Cell Lung Cancer	NCI-H322M		-4.44
Leukemia	MOLT-4		-4.43
Breast Cancer	T-47D		-4.43
Renal Cancer	A498		-4.42
Non-Small Cell Lung Cancer	NCI-H522	*	-4.37
Melanoma	SK-MEL-5		-4.32
Melanoma	MDA-MB-435	*	-4.27
Colon Cancer	HCC-2998		-4.26
Leukemia	CCRF-CEM		-4.24
Leukemia	RPMI-8226		-4.15
Leukemia	HL-60(TB)		> -4.00
Colon Cancer	KM12		> -4.00

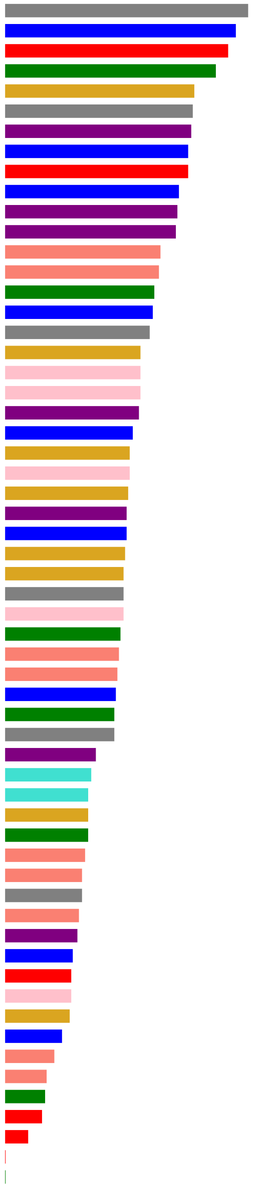

Log10 High Conc :-4.0

National Cancer Institute Developmental Therapeutics Program<br>Waterfall Graph TGI	NSC : D - 838963/1	Units :Molar	SSPL :1CJS	EXP. ID :2212NS79
	Report Date :December 11, 2023		Test Date :December 12, 2022	

Panel	Cell Name	Hollow Fiber	TGI
CNS Cancer	SF-539		-5.13
Non-Small Cell Lung Cancer	NCI-H23	*	-4.92
Ovarian Cancer	NCI/ADR-RES		-4.62
Renal Cancer	UO-31		-4.54
Renal Cancer	ACHN		-4.53
Colon Cancer	HCT-116		-4.43
Breast Cancer	MDA-MB-231/ATCC	*	-4.42
Ovarian Cancer	IGROV1		-4.40
Melanoma	UACC-62	*	-4.39
Renal Cancer	CAKI-1		-4.39
Non-Small Cell Lung Cancer	HOP-62		-4.39
CNS Cancer	SF-295	*	-4.39
CNS Cancer	U251	*	-4.37
Renal Cancer	TK-10		-4.34
Non-Small Cell Lung Cancer	HOP-92		-4.28
Melanoma	MALME-3M		-4.27
Non-Small Cell Lung Cancer	NCI-H460		-4.26
Breast Cancer	BT-549		-4.21
Renal Cancer	RXF 393		-4.21
CNS Cancer	SNB-75		-4.20
Melanoma	LOX IMVI	*	-4.19
Melanoma	SK-MEL-28		-4.19
Renal Cancer	786-0		-4.10
CNS Cancer	SNB-19		-4.10
Non-Small Cell Lung Cancer	EKVX		-4.08
Breast Cancer	HS 578T		-4.07
Breast Cancer	MCF7		-4.03
Ovarian Cancer	OVCAR-5	*	-4.03
Leukemia	CCRF-CEM		> -4.00
Leukemia	HL-60(TB)		> -4.00
Leukemia	K-562		> -4.00
Leukemia	MOLT-4		> -4.00
Leukemia	RPMI-8226		> -4.00
Leukemia	SR		> -4.00
Non-Small Cell Lung Cancer	A549/ATCC		> -4.00
Non-Small Cell Lung Cancer	NCI-H226		> -4.00
Non-Small Cell Lung Cancer	NCI-H322M		> -4.00
Non-Small Cell Lung Cancer	NCI-H522	*	> -4.00
Colon Cancer	COLO 205	*	> -4.00
Colon Cancer	HCC-2998		> -4.00
Colon Cancer	HCT-15		> -4.00
Colon Cancer	HT29		> -4.00
Colon Cancer	KM12		> -4.00
Colon Cancer	SW-620	*	> -4.00
CNS Cancer	SF-268		> -4.00
Melanoma	M14		> -4.00
Melanoma	MDA-MB-435	*	> -4.00
Melanoma	SK-MEL-2		> -4.00
Melanoma	SK-MEL-5		> -4.00
Melanoma	UACC-257		> -4.00
Ovarian Cancer	OVCAR-3	*	> -4.00
Ovarian Cancer	OVCAR-4		> -4.00
Ovarian Cancer	OVCAR-8		> -4.00
Ovarian Cancer	SK-OV-3		> -4.00
Renal Cancer	A498		> -4.00
Renal Cancer	SN12C		> -4.00
Prostate Cancer	PC-3		> -4.00
Prostate Cancer	DU-145		> -4.00
Breast Cancer	T-47D		> -4.00

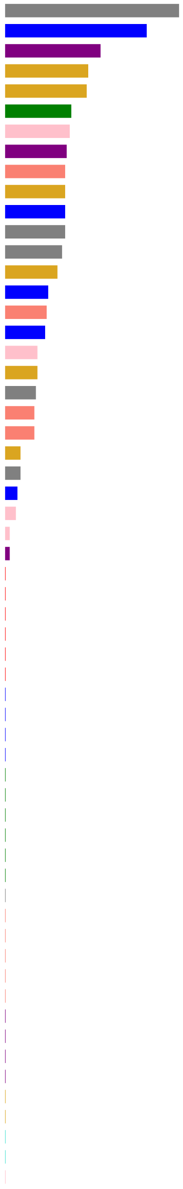

Log10 High Conc : -4.0

National Cancer Institute Developmental Therapeutics Program<br>Waterfall Graph LC50	NSC : D - 838963/1	Units :Molar	SSPL :1CJS	EXP. ID :2212NS79
	Report Date :December 11, 2023		Test Date :December 12, 2022	

Panel	Cell Name	Hollow Fiber	LC50
CNS Cancer	SF-539		-4.49
Renal Cancer	ACHN		-4.17
Ovarian Cancer	NCI/ADR-RES		-4.12
Non-Small Cell Lung Cancer	NCI-H23	*	-4.04
Leukemia	CCRF-CEM		> -4.00
Leukemia	HL-60(TB)		> -4.00
Leukemia	K-562		> -4.00
Leukemia	MOLT-4		> -4.00
Leukemia	RPMI-8226		> -4.00
Leukemia	SR		> -4.00
Non-Small Cell Lung Cancer	A549/ATCC		> -4.00
Non-Small Cell Lung Cancer	EKVX		> -4.00
Non-Small Cell Lung Cancer	HOP-62		> -4.00
Non-Small Cell Lung Cancer	HOP-92		> -4.00
Non-Small Cell Lung Cancer	NCI-H226		> -4.00
Non-Small Cell Lung Cancer	NCI-H322M		> -4.00
Non-Small Cell Lung Cancer	NCI-H460		> -4.00
Non-Small Cell Lung Cancer	NCI-H522	*	> -4.00
Colon Cancer	COLO 205	*	> -4.00
Colon Cancer	HCC-2998		> -4.00
Colon Cancer	HCT-116		> -4.00
Colon Cancer	HCT-15		> -4.00
Colon Cancer	HT29		> -4.00
Colon Cancer	KM12		> -4.00
Colon Cancer	SW-620	*	> -4.00
CNS Cancer	SF-268		> -4.00
CNS Cancer	SF-295	*	> -4.00
CNS Cancer	SNB-19		> -4.00
CNS Cancer	SNB-75		> -4.00
CNS Cancer	U251	*	> -4.00
Melanoma	LOX IMVI	*	> -4.00
Melanoma	MALME-3M		> -4.00
Melanoma	M14		> -4.00
Melanoma	MDA-MB-435	*	> -4.00
Melanoma	SK-MEL-2		> -4.00
Melanoma	SK-MEL-28		> -4.00
Melanoma	SK-MEL-5		> -4.00
Melanoma	UACC-257		> -4.00
Melanoma	UACC-62	*	> -4.00
Ovarian Cancer	IGROV1		> -4.00
Ovarian Cancer	OVCAR-3	*	> -4.00
Ovarian Cancer	OVCAR-4		> -4.00
Ovarian Cancer	OVCAR-5	*	> -4.00
Ovarian Cancer	OVCAR-8		> -4.00
Ovarian Cancer	SK-OV-3		> -4.00
Renal Cancer	786-0		> -4.00
Renal Cancer	A498		> -4.00
Renal Cancer	CAKI-1		> -4.00
Renal Cancer	RXF 393		> -4.00
Renal Cancer	SN12C		> -4.00
Renal Cancer	TK-10		> -4.00
Renal Cancer	UO-31		> -4.00
Prostate Cancer	PC-3		> -4.00
Prostate Cancer	DU-145		> -4.00
Breast Cancer	MCF7		> -4.00
Breast Cancer	MDA-MB-231/ATCC	*	> -4.00
Breast Cancer	HS 578T		> -4.00
Breast Cancer	BT-549		> -4.00
Breast Cancer	T-47D		> -4.00

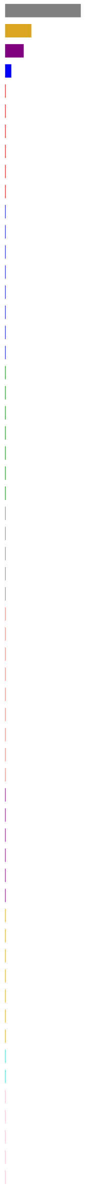

Log10 High Conc : -4.0
