# Supplementary material for: Synthesis and Anticancer Activity of New Quinazolin-4(3H)-one Derivatives: Identification of a Tumor-Selective Anticancer Agent with Potential Inhibition of TGF-βRI (ALK5)
Source: Pharmaceuticals (Basel). 2026 Jun 26;19(7):996. doi: 10.3390/ph19070996 (PMC13416033; doi:10.3390/ph19070996)
Supplement: Supplementary file 1 [file pharmaceuticals-19-00996-s001.zip › Proofread Supplementary Materials/Supplementary Materials-24 June 26.pdf]

Synthesis and Anticancer Activity of New Quinazolin-4(3*H*)-one Derivatives: Identification of a Tumor-Selective Anticancer Agent with Potential Inhibition of TGF-βRI (ALK5)

Nahed N. E. El-Sayed [1,*], Sami A. Al-Hussain [2,*], Marwa A. Ibrahim [3], Mohamed R. Elnagar [4,5], Zainab M. Almarhoon [6] and Magdi E. A. Zaki [2]

[1] Egyptian Drug Authority (EDA), 51 Wezaret El-Zeraa St., Giza 35521, Egypt
[2] Chemistry Department, Faculty of Science, Imam Mohammad Ibn Saud Islamic University (IMSIU), Riyadh 11623, Saudi Arabia; mezaki@imamu.edu.sa (M.E.A.Z.)
[3] Department of Pharmaceutical Chemistry, Faculty of Pharmacy, Cairo University, Cairo 11562, Egypt; marwa.abdelaziz@pharma.cu.edu.eg
[4] Department of Pharmacology and Toxicology, Faculty of Pharmacy, Al-Azhar University, Cairo 011823, Egypt; ohamed.r.elnagar@azhar.edu.eg
[5] Department of Pharmacology, College of Pharmacy, The Islamic University, Najaf 54001, Iraq
[6] Department of Chemistry, College of Sciences, King Saud University, P.O. Box 2455, Riyadh 11451, Saudi Arabia; zalmarhoon@ksu.edu.sa
* Correspondence: nahed.elsayed@edaegypt.gov.eg (N.N.E.E.); sahussain@imamu.edu.sa (S.A.A.)

Supplementary Materials

Table S1: Energy minimization calculations for E and Z isomers of compounds 6a–6d d and 8		
Comp.#	*E*-isomer	*Z*-isomer
**6a**	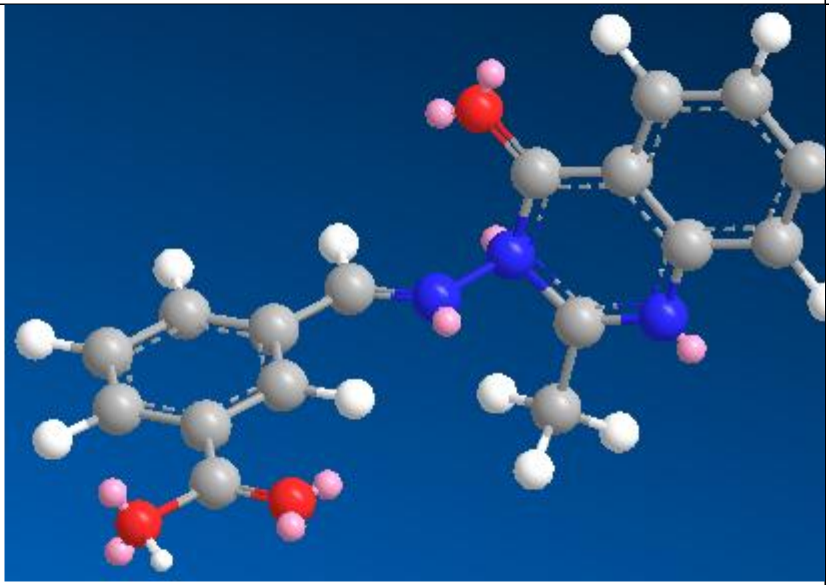 Total Energy: 16.8457 kcal/mol	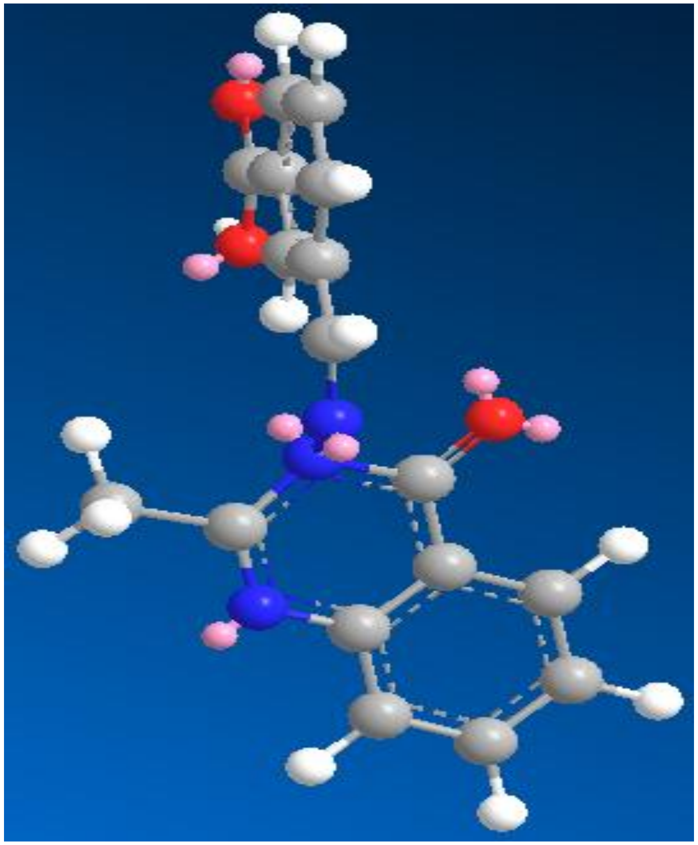 Total Energy: 20.3540 kcal/mol

**6b**	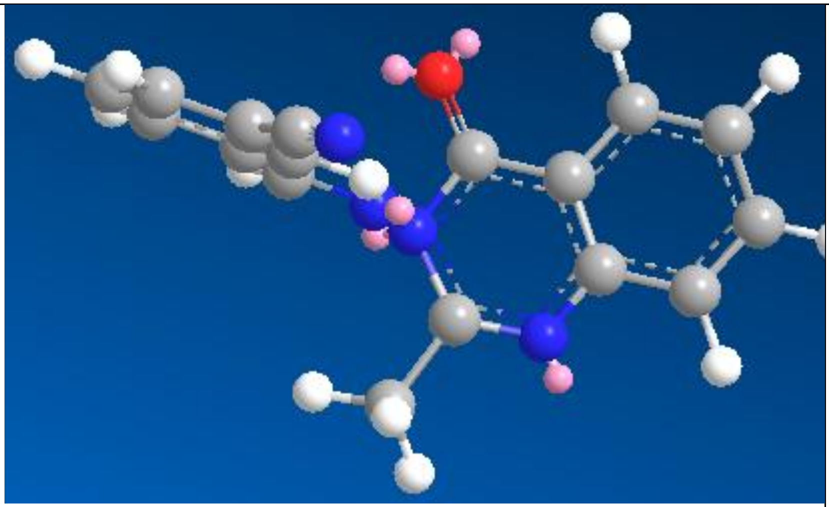<br>Total Energy: 13.3047 kcal/mol	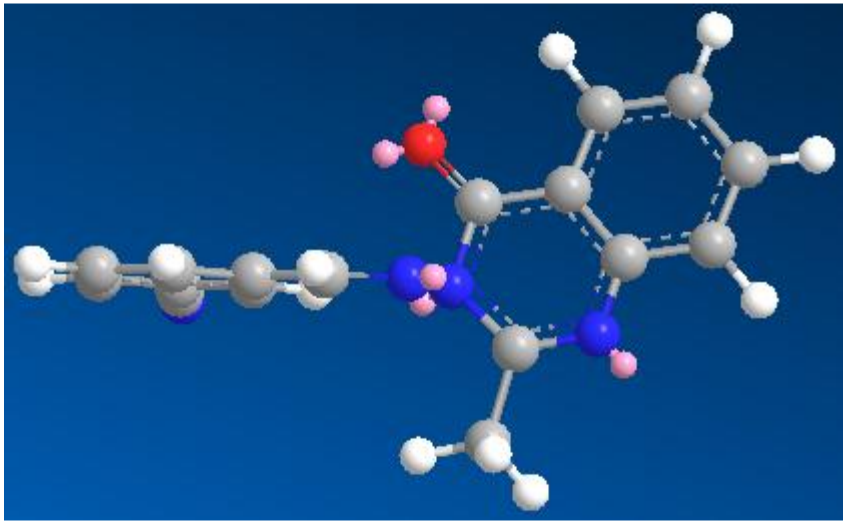<br>Total Energy: 16.8546 kcal/mol
**6c**	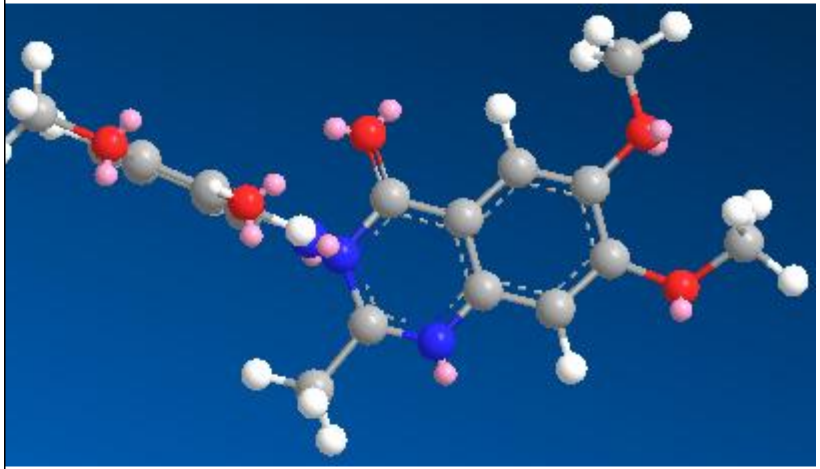<br>Total Energy: 25.2409 kcal/mol	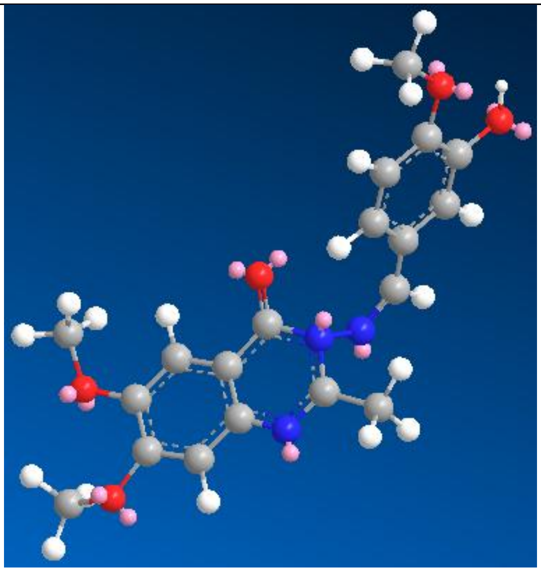<br>Total Energy: 28.8004 kcal/mol

<table>
<tr><td>6d</td><td>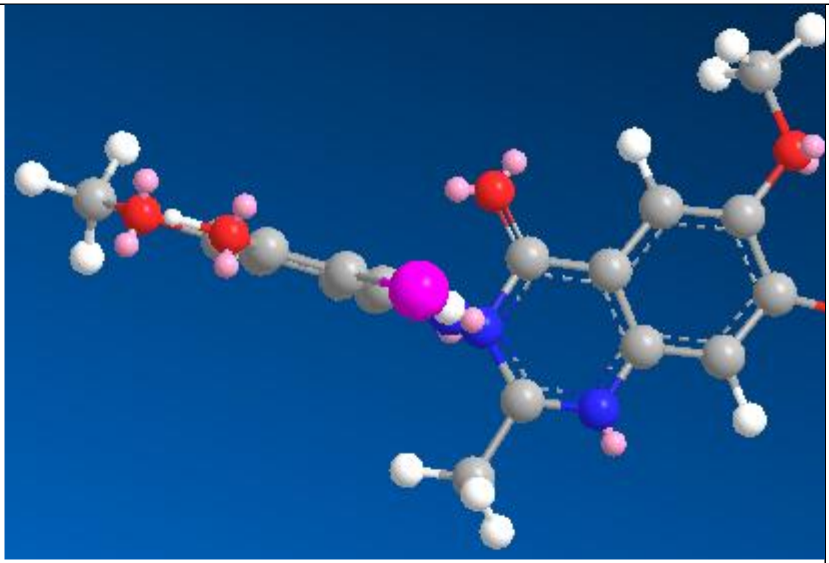
Total Energy: 26.4570 kcal/mol</td><td>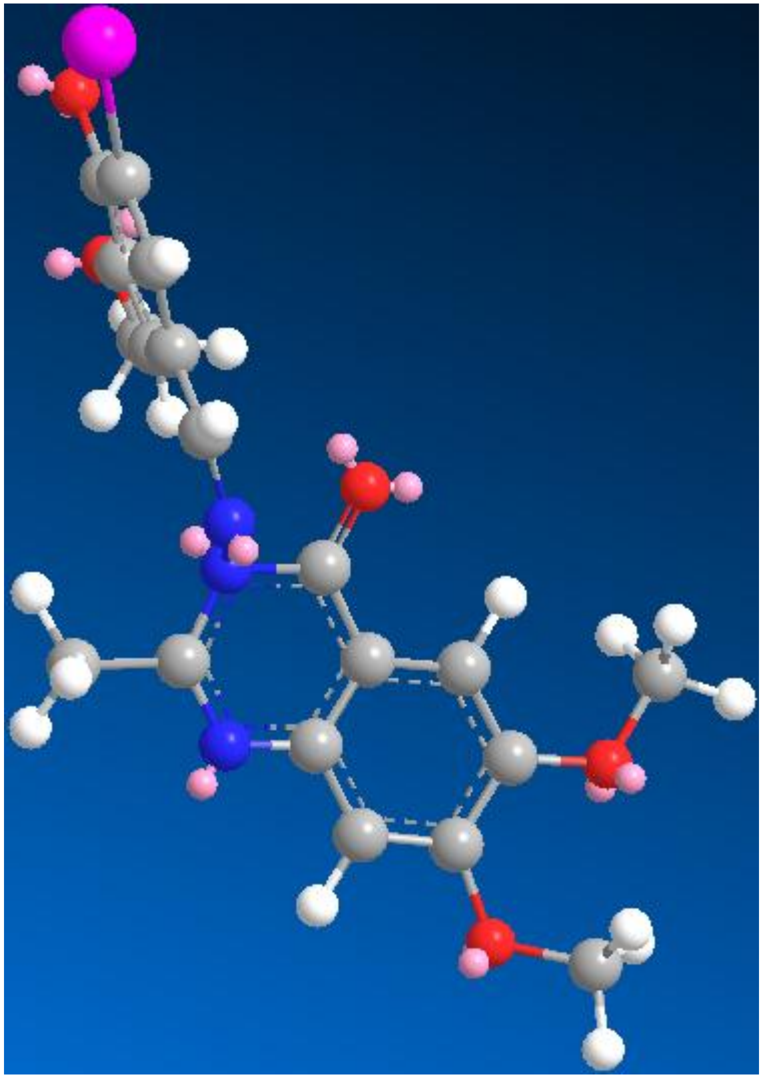
Total Energy: 29.8715 kcal/mol</td></tr>
</table>

**8**	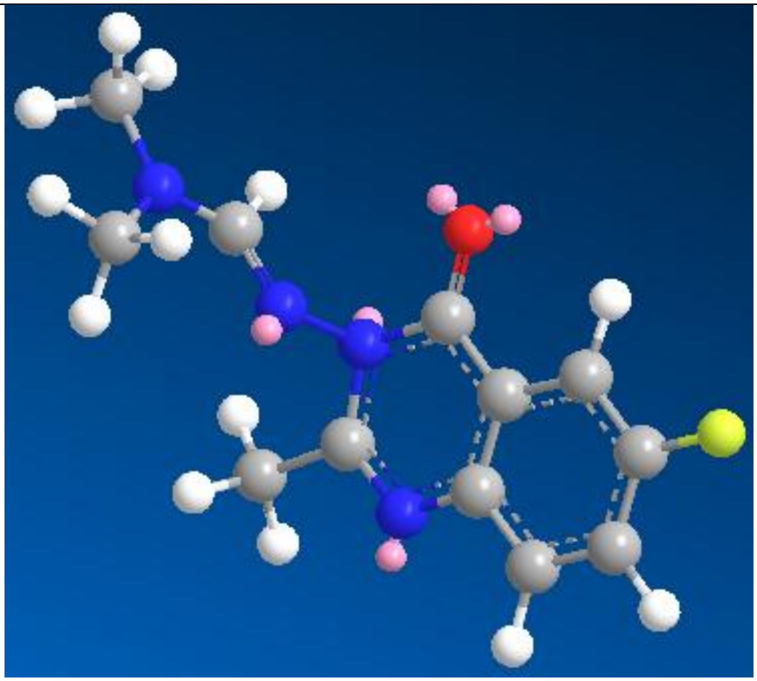<br>Total Energy: 17.4647 kcal/mol	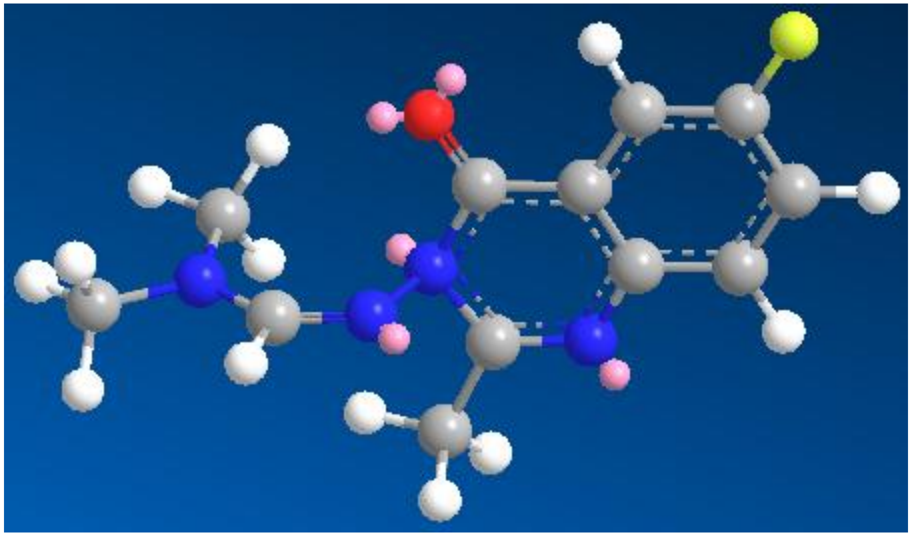<br>Total Energy: 19.9799 kcal/mol

Table S2. Analysis of mean graph data for compounds **1a**,**b**; **3a–h**; **6a–d**; **8**; and **10a**,**b** as obtained from the NCI-60 one-dose cell line screen (at 10 μM).

Comp.	Mean G% [a]	Delta [b]	Range [c]	Most sensitive line(s) (G%)	Resistant Line(s) (G%)	Cell line(s) showing cytotoxic response (G% < 0)	Cell lines showing cytostatic response (0 < G% ≤ 10)	Cell lines showing cytostatic response (10% < G% < 50)
**1a**	103.03	23.42	44.72	UO-31 (79.61)	HCC-2998 (124.33)	Non	Non	Non
**1b**	106.82	21.13	38.75	UO-31 (85.69)	A498 (124.44)	Non	Non	Non
**3a**	62.58	84.14	130.01	SNB-19 (7.05)	TK-10 (108.45)	SNB-75 (−20.71); and CAKI-1 (−21.56).	Non	HOP-62 (26.46); HOP-92 (26.92); NCI-H226 (21.49); HCT-116 (44.79); SF-539 (25.74); SNB-19/7.05U251 (48.82); OVCAR-8 (32.45); 786-0 (34.72); ACHN (37.79); RXF 393 (18.82); UO-31 (39.65); HS 578T (25.70); and BT-549 (31.98)
**3b**	81.44	43.47	80.25	NCI-H226 (37.97)	A498 (118.22)	Non	Non	HOP-92 (44.61); NCI-H226 (37.97); and HS 578T (47.99)
**3c**	80.05	58.58	118.64	SNB-75 (21.47)	A498 (140.11)	Non	Non	SNB-19 (33.27); SNB-75 (21.47); ACHN (47.53); and HS 578T (34.47)
**3d**	87.49	46.76	79.82	HS 578T (40.73)	A498 (120.55	Non	Non	SNB-19 (45.71); SNB-75 (41.65); 786-0 (48.97), and HS 578T (40.73)
**3e**	33.47	82.86	144.86	HOP-62 (−49.39)	KM12 (95.47)	HOP-62 (−49.39); HOP-92 (−6.91); NCI-H23 (−18.93); SF-539 (−16.23); U251 (−34.75); and RXF 393 (−17.74)	NCI-H460/6.80; COLO 205/9.31; HT29/8.82; UACC-62/3.64; OVCAR-8/0.01; NCI/ADR-RES/3.80; ACHN/5.25; and TK-10/7.44	K-562/35.25; SR (40.43); A549/ATCC (16.78); EKVX (24.84); NCI-H226 (17.38); NCI-H522 (18.67); HCT-116 (10.28); SW-620 (44.47); ; SF-295 (11.41); SNB-19 (33.90); LOX IMVI (20.77); MALME-3M (42.35); M14 (29.41); SK-MEL-28 (27.84); IGROV1 (49.86); OVCAR-4 (21.72); 786-0 (36.31); PC-3 (46.81); ; MCF7 (39.25); HS 578T (15.05); BT-549 (23.97); T-47D (20.71); MDA-MB-468 (43.86)
**3f**	48.84	70.73	139.39	NCI-H226 (−21.89)	HL-60(TB) (117.50)	HOP-62 (−17.14); HOP-92 (−2.96); NCI-H226 (−21.89); SNB-75 (−19.41); RXF 393 (−10.65); and HS 578T (−18.89)	SF-295 (4.69); SF-539 (2.03); and BT-549 (1.44)	NCI-H23/46.04; NCI-H460 (22.42); HCT-116 (24.43); HT29 (49.94); SF-268 (28.90); U251 (19.60); MALME-3M (39.64); OVCAR-4 (30.97); OVCAR-8 (37.76); NCI/ADR-RES (28.27); SK-OV-3: 45.95; 786-0 (10.73); ACHN (33.34); CAKI-1 (10.14); PC-3 (25.92); MDA-MB-231/ATCC (13.66); and MDA-MB-468 (44.71)
**3g**	87.63	42.60	80.77	SNB-75 (45.03)	A498 (125.80)	Non	Non	SNB-75 (45.03) and HS 578T (45.61)
**3h**	99.52	28.87	55.86	UACC-62 (70.65)	M14 (126.51)	Non	Non	Non
**6a**	97.86	32.66	57.84	MOLT-4 (65.20)	SK-MEL-5 (123.04)	Non	Non	Non
**6b**	103.50	39.89	69.36	MDA-MB-468 (63.61)	COLO 205 (132.97)	Non	Non	Non
**6c**	93.06	34.18	48.26	SR (58.88)	COLO 205 (107.14)	Non	Non	Non
**6d**	76.79	52.06	78.85	RPMI-8226 (24.73)	TK-10 (103.58)	Non	Non	CCRF-CEM (49.53); RPMI-8226 (24.73); SR (40.49); LOXIMVI (48.44); and SN12C (42.91)
**8**	104.28	65.67	85.39	HCC-2998 (38.61)	BT-549 (124.00)	Non	Non	HCC-2998 (38.61)
**10a**	99.21	25.18	41.61	HL-60(TB) (74.03)	TK-10 and OVCAR-5 (115.64)	Non	Non	Non
**10b**	81.13	47.57	77.72	RPMI-8226 (33.56)	SK-MEL-28 (111.28)	Non	Non	CCRF-CEM (45.99); HL-60(TB) (43.76); K-562 (34.25); and RPMI-8226/33.56

Mean G% [a]: reflects potency; Delta [b]: reflects response heterogeneity, Range [c]: reflects tumor-type selectivity (larger gap between most sensitive and most resistant cell lines).

Table S3. The sequences for primers used in quantitative real time qRT-PCR.

Gene	Primer sequence
Bax	F: 5′-CAAGAAGCTGAGCGAGTGTCT-3′<br>R: 5′- CAATCATCCTCTGCAGCTCCATATT-3′
Bcl-2	F: 5′-TGCGCTCAGCCCTGTG-3′<br>R: 5′- GGTAGCGACGAGAGAAGTCATC-3′
Caspase-3	F: 5′-TGTGAGGCGGTTGTAGAAGTT-3′<br>R: 5′-CGCTTCCATGTATGATCTTTGGTT-3′
Caspase-9	F: 5′-TGGAGACTCGAGGGAGTCAG-3′<br>R: 5′- TCGACAACTTTGCTGCTTGC-3′
GAPDH	F: 5′-AGAGGCAGGGATGTTCTG-3′<br>R: 5′- GACTCATGACCACAGTCCATGC-3′

Figure S1. The activity of cancer-related pathways inferred using PROGENy and CytoSig cytokines (from the Human Protein Atlas) for the most responsive human cancer cell lines having GI_{50} values below the MID (17.38 μM).

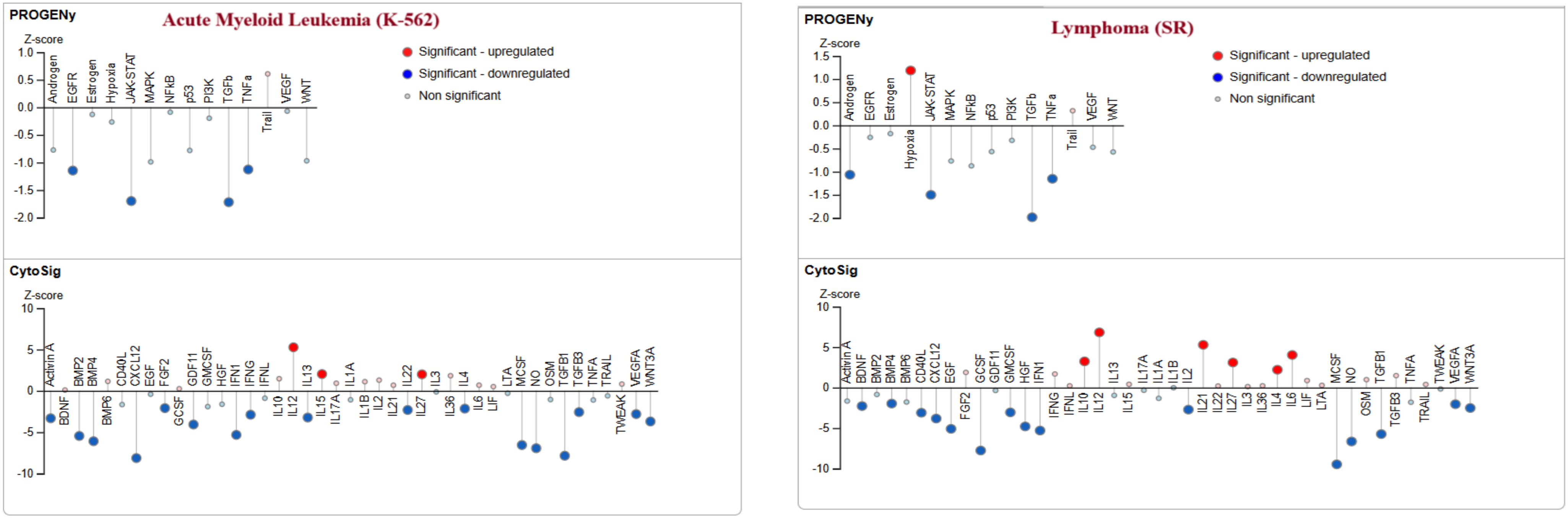

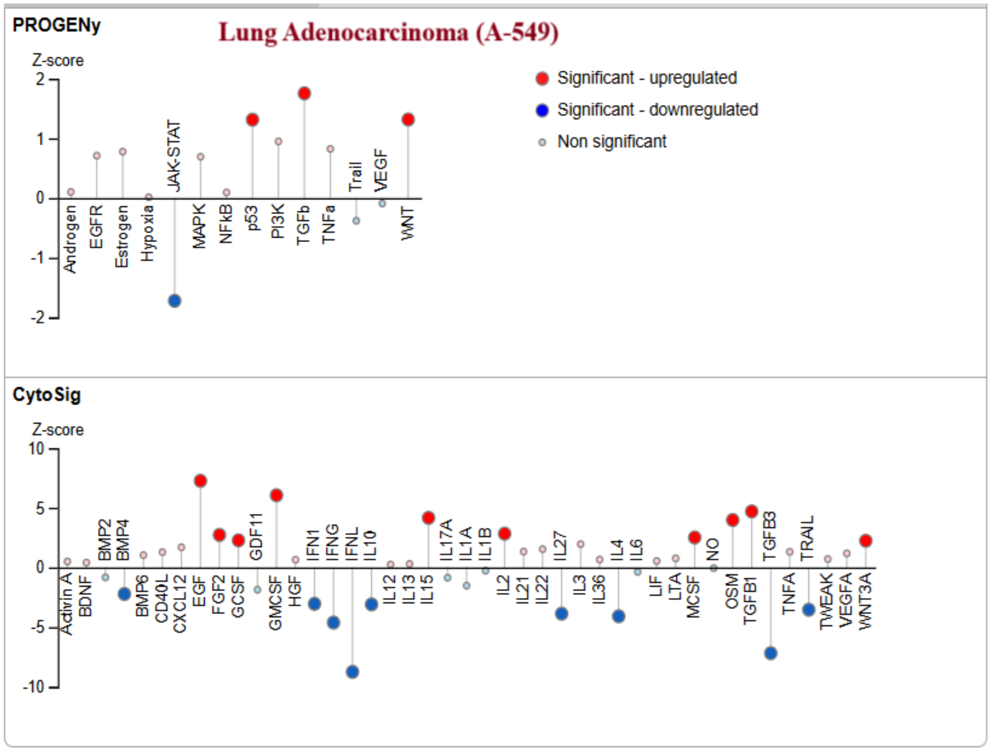
PROGENy
Lung Adenocarcinoma (A-549)
Z-score
Significant - upregulated
Significant - downregulated
Non significant
Androgen
EGFR
Estrogen
Hypoxia
JAK-STAT
MAPK
NFkB
p53
PI3K
TGFb
TNFa
Trail
VEGF
WNT
CytoSig
Z-score
Activin A
BDNF
BMP2
BMP4
BMP6
CD40L
CXCL12
EGF
FGF2
GCSF
GDF11
GMCSF
HGF
IFN1
IFNG
IFNL
IL10
IL12
IL13
IL15
IL17A
IL1A
IL1B
IL2
IL21
IL22
IL27
IL3
IL36
IL4
IL6
LIF
LTA
MCSF
NO
OSM
TGFB1
TGFB3
TNFA
TRAIL
TWEAK
VEGFA
WNT3A

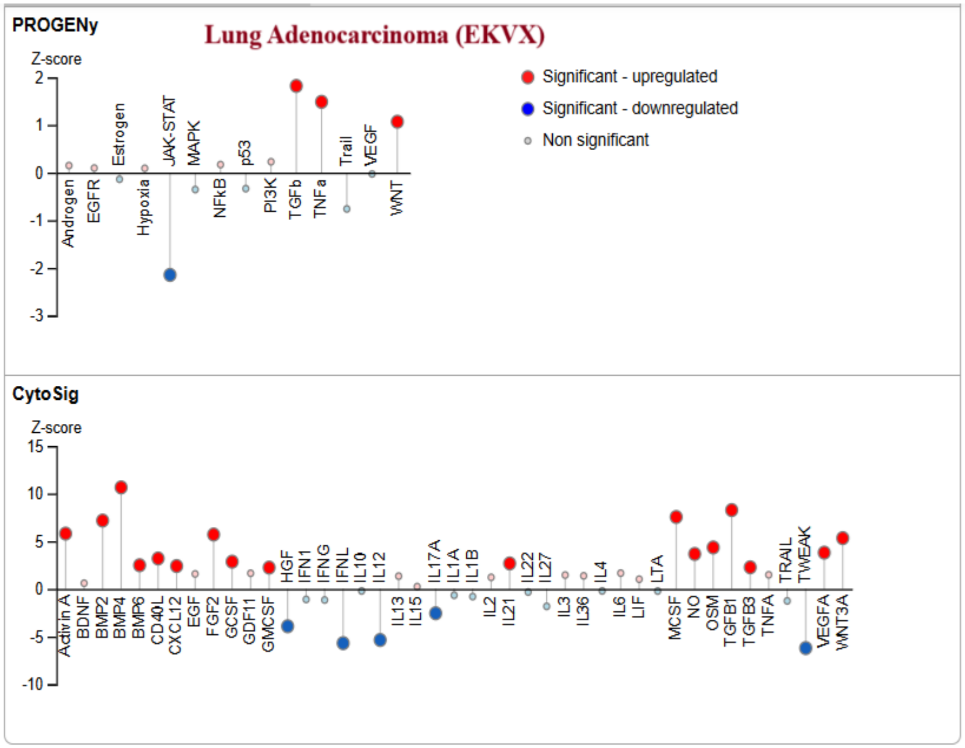
PROGENy
Lung Adenocarcinoma (EKVX)
Z-score
Significant - upregulated
Significant - downregulated
Non significant
Androgen
EGFR
Estrogen
Hypoxia
JAK-STAT
MAPK
NFkB
p53
PI3K
TGFb
TNFa
Trail
VEGF
WNT
CytoSig
Z-score
Activin A
BDNF
BMP2
BMP4
BMP6
CD40L
CXCL12
EGF
FGF2
GCSF
GDF11
GMCSF
HGF
IFN1
IFNG
IFNL
IL10
IL12
IL13
IL15
IL17A
IL1A
IL1B
IL2
IL21
IL22
IL27
IL3
IL36
IL4
IL6
LIF
LTA
MCSF
NO
OSM
TGFB1
TGFB3
TNFA
TRAIL
TWEAK
VEGFA
WNT3A

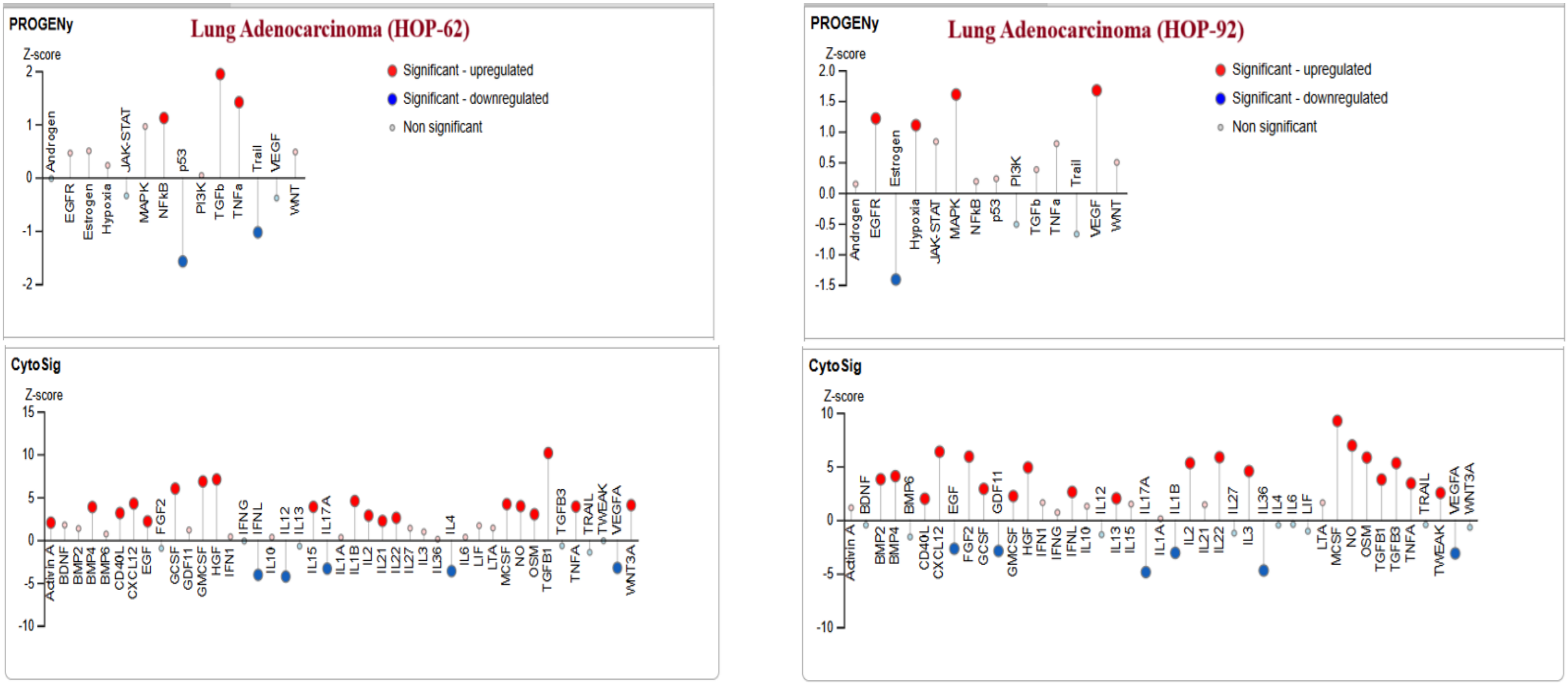
PROGENy
Lung Adenocarcinoma (HOP-62)
Z-score
Significant - upregulated
Significant - downregulated
Non significant
Androgen
EGFR
Estrogen
Hypoxia
JAK-STAT
MAPK
NFkB
p53
PI3K
TGFb
TNFa
Trail
VEGF
WNT
CytoSig
Z-score
Activin A
BDNF
BMP2
BMP4
BMP6
CD40L
CXCL12
EGF
FGF2
GCSF
GDF11
GMCSF
HGF
IFN1
IFNG
IFNL
IL10
IL12
IL13
IL15
IL17A
IL1A
IL1B
IL2
IL21
IL22
IL27
IL3
IL36
IL4
IL6
LIF
LTA
MCSF
NO
OSM
TGFB1
TGFB3
TNFA
TRAIL
TWEAK
VEGFA
WNT3A
PROGENy
Lung Adenocarcinoma (HOP-92)
Z-score
Significant - upregulated
Significant - downregulated
Non significant
CytoSig
Z-score

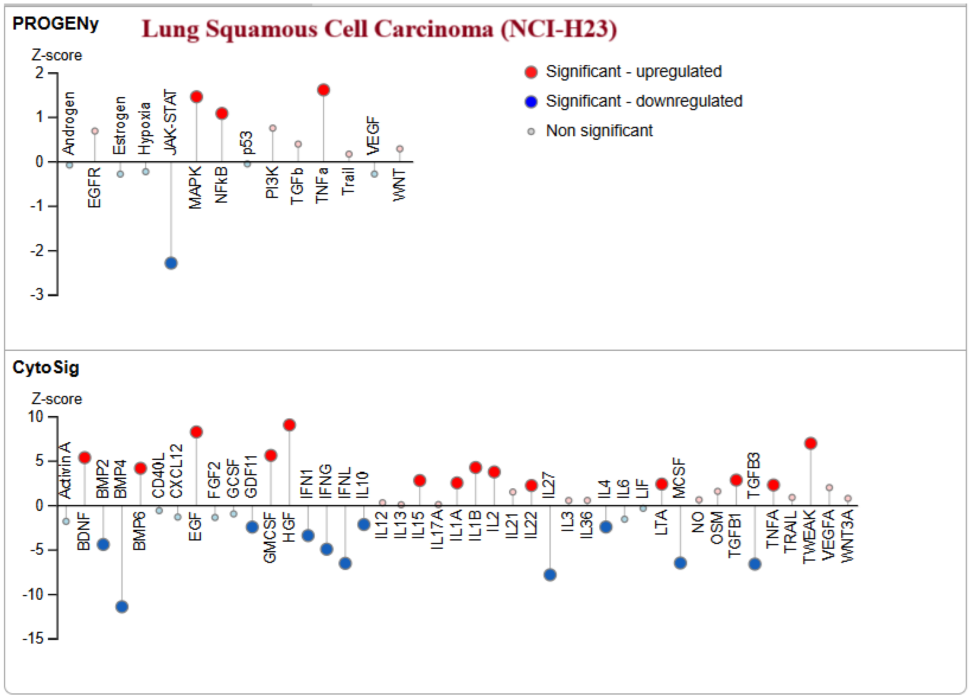
PROGENy
Lung Squamous Cell Carcinoma (NCI-H23)
Z-score
Significant - upregulated
Significant - downregulated
Non significant
CytoSig
Z-score

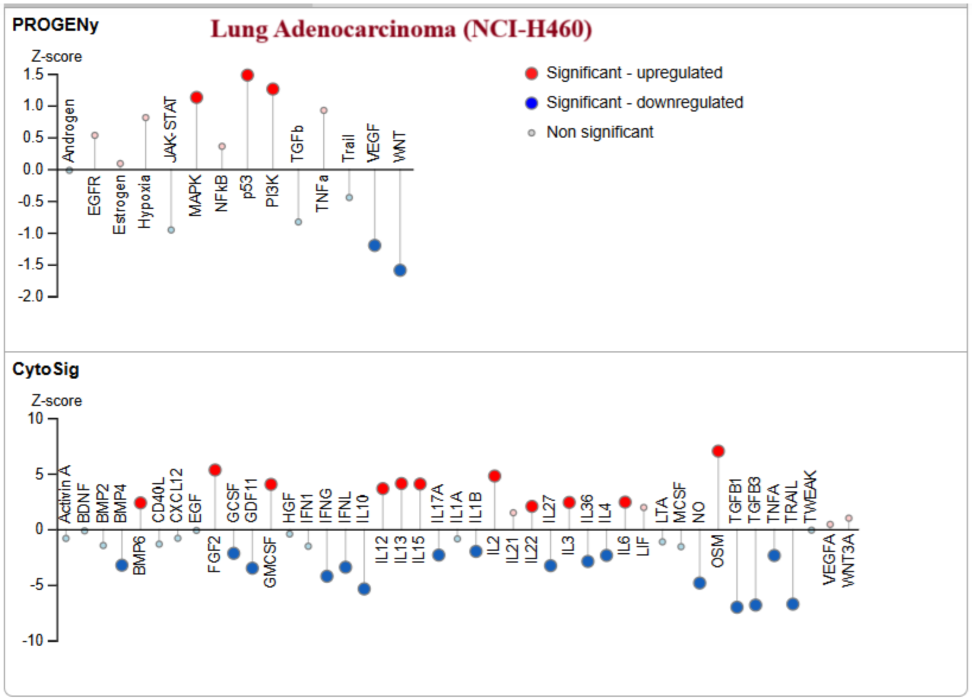
PROGENy
Lung Adenocarcinoma (NCI-H460)
Z-score
Significant - upregulated
Significant - downregulated
Non significant
CytoSig
Z-score

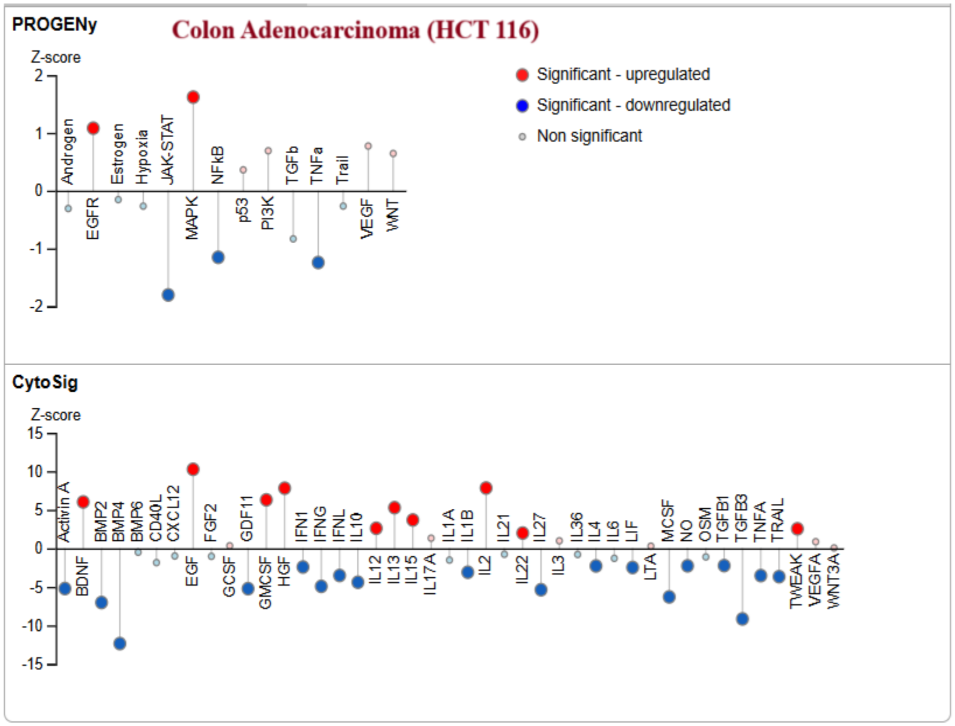
PROGENy
Colon Adenocarcinoma (HCT 116)
Z-score
Significant - upregulated
Significant - downregulated
Non significant
Androgen
EGFR
Estrogen
Hypoxia
JAK-STAT
MAPK
NFkB
p53
PI3K
TGFb
TNFa
Trail
VEGF
WNT
CytoSig
Z-score
Activin A
BDNF
BMP2
BMP4
BMP6
CD40L
CXCL12
EGF
FGF2
GCSF
GDF11
GMCSF
HGF
IFN1
IFNG
IFNL
IL10
IL12
IL13
IL15
IL17A
IL1A
IL1B
IL2
IL21
IL22
IL27
IL3
IL36
IL4
IL6
LIF
LTA
MCSF
NO
OSM
TGFB1
TGFB3
TNFA
TRAIL
TWEAK
VEGFA
WNT3A

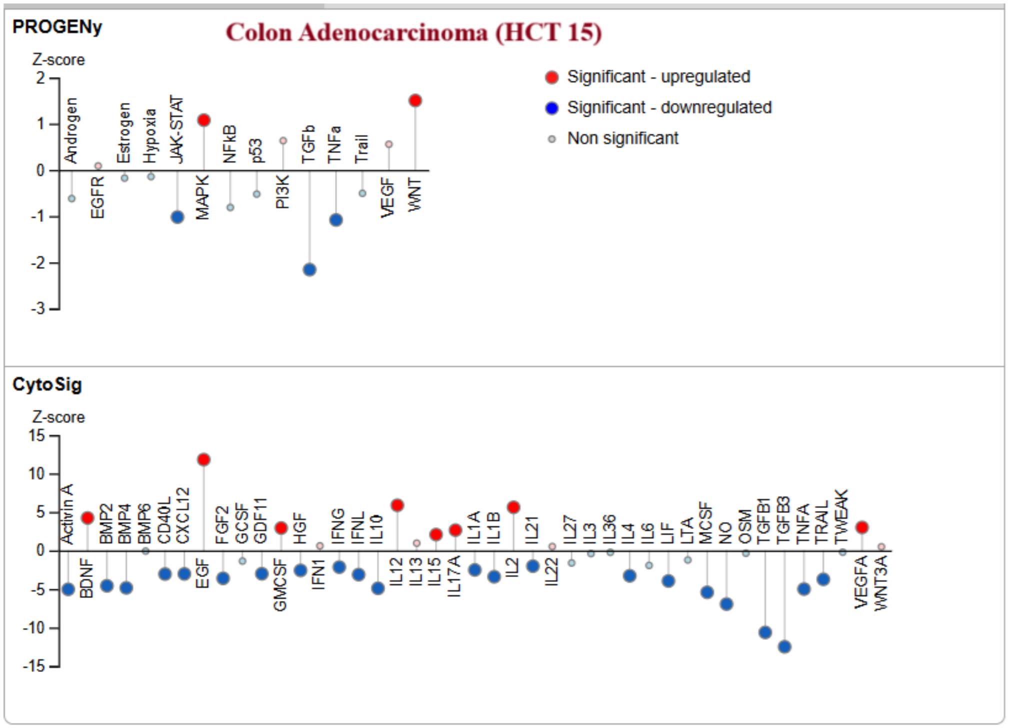
PROGENy
Colon Adenocarcinoma (HCT 15)
Z-score
Significant - upregulated
Significant - downregulated
Non significant
Androgen
EGFR
Estrogen
Hypoxia
JAK-STAT
MAPK
NFkB
p53
PI3K
TGFb
TNFa
Trail
VEGF
WNT
CytoSig
Z-score
Activin A
BDNF
BMP2
BMP4
BMP6
CD40L
CXCL12
EGF
FGF2
GCSF
GDF11
GMCSF
HGF
IFN1
IFNG
IFNL
IL10
IL12
IL13
IL15
IL17A
IL1A
IL1B
IL2
IL21
IL22
IL27
IL3
IL36
IL4
IL6
LIF
LTA
MCSF
NO
OSM
TGFB1
TGFB3
TNFA
TRAIL
TWEAK
VEGFA
WNT3A

CNS Cancer

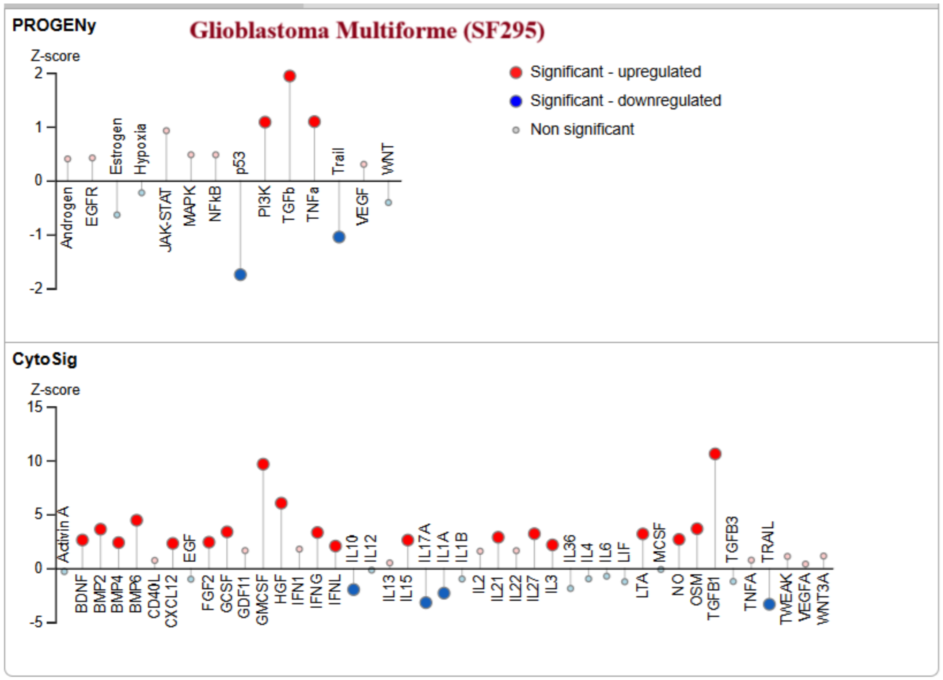
PROGENy
Glioblastoma Multiforme (SF295)
Z-score
Significant - upregulated
Significant - downregulated
Non significant
Androgen EGFR Estrogen Hypoxia JAK-STAT MAPK NFkB p53 PI3K TGFb TNFa Trail VEGF WNT
CytoSig
Z-score
Activin A BDNF BMP2 BMP4 BMP6 CD40L CXCL12 EGF FGF2 GCSF GDF11 GMCSF HGF IFN1 IFNG IFNL IL10 IL12 IL13 IL15 IL17A IL1A IL1B IL2 IL21 IL22 IL27 IL3 IL36 IL4 IL6 LIF LTA MCSF NO OSM TGFB1 TGFB3 TNFA TRAIL TWEAK VEGFA WNT3A

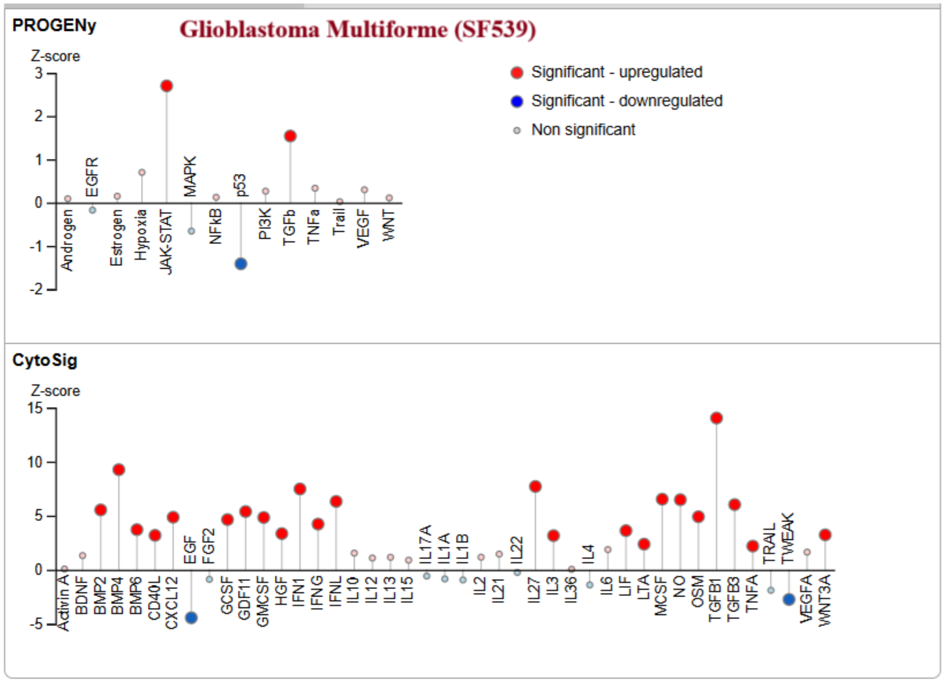
PROGENy
Glioblastoma Multiforme (SF539)
Z-score
Significant - upregulated
Significant - downregulated
Non significant
Androgen EGFR Estrogen Hypoxia JAK-STAT MAPK NFkB p53 PI3K TGFb TNFa Trail VEGF WNT
CytoSig
Z-score
Activin A BDNF BMP2 BMP4 BMP6 CD40L CXCL12 EGF FGF2 GCSF GDF11 GMCSF HGF IFN1 IFNG IFNL IL10 IL12 IL13 IL15 IL17A IL1A IL1B IL2 IL21 IL22 IL27 IL3 IL36 IL4 IL6 LIF LTA MCSF NO OSM TGFB1 TGFB3 TNFA TRAIL TWEAK VEGFA WNT3A

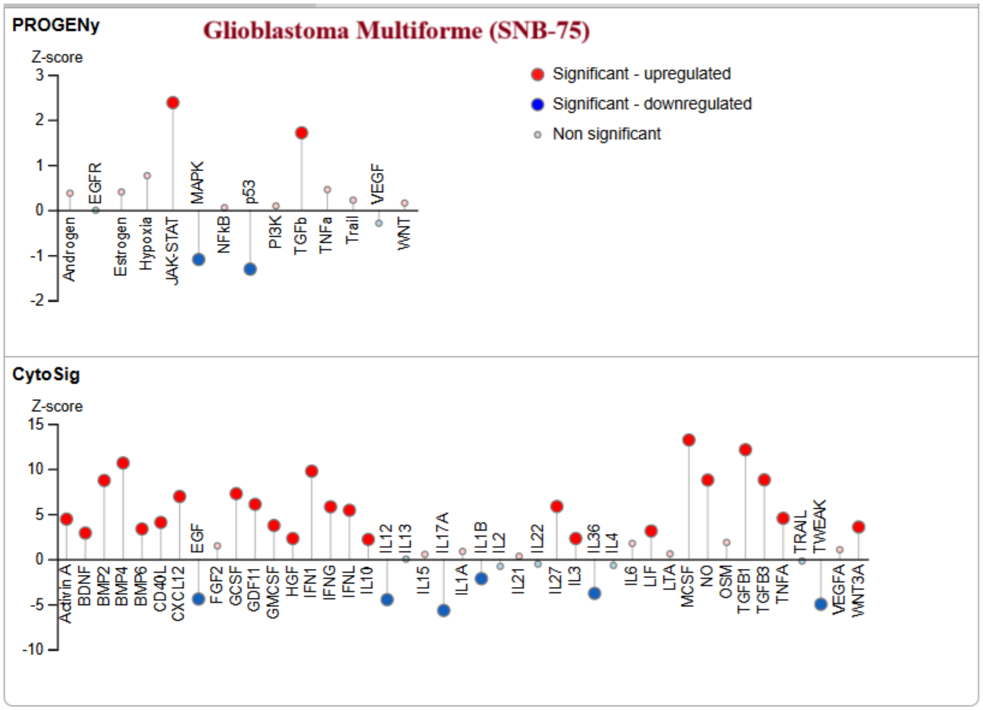
PROGENy
Glioblastoma Multiforme (SNB-75)
Z-score
Significant - upregulated
Significant - downregulated
Non significant
Androgen
EGFR
Estrogen
Hypoxia
JAK-STAT
MAPK
NFkB
p53
PI3K
TGFb
TNFa
Trail
VEGF
WNT
CytoSig
Z-score
Activin A
BDNF
BMP2
BMP4
BMP6
CD40L
CXCL12
EGF
FGF2
GCSF
GDF11
GMCSF
HGF
IFN1
IFNG
IFNL
IL10
IL12
IL13
IL15
IL17A
IL1A
IL1B
IL2
IL21
IL22
IL27
IL3
IL36
IL4
IL6
LIF
LTA
MCSF
NO
OSM
TGFB1
TGFB3
TNFA
TRAIL
TWEAK
VEGFA
WNT3A

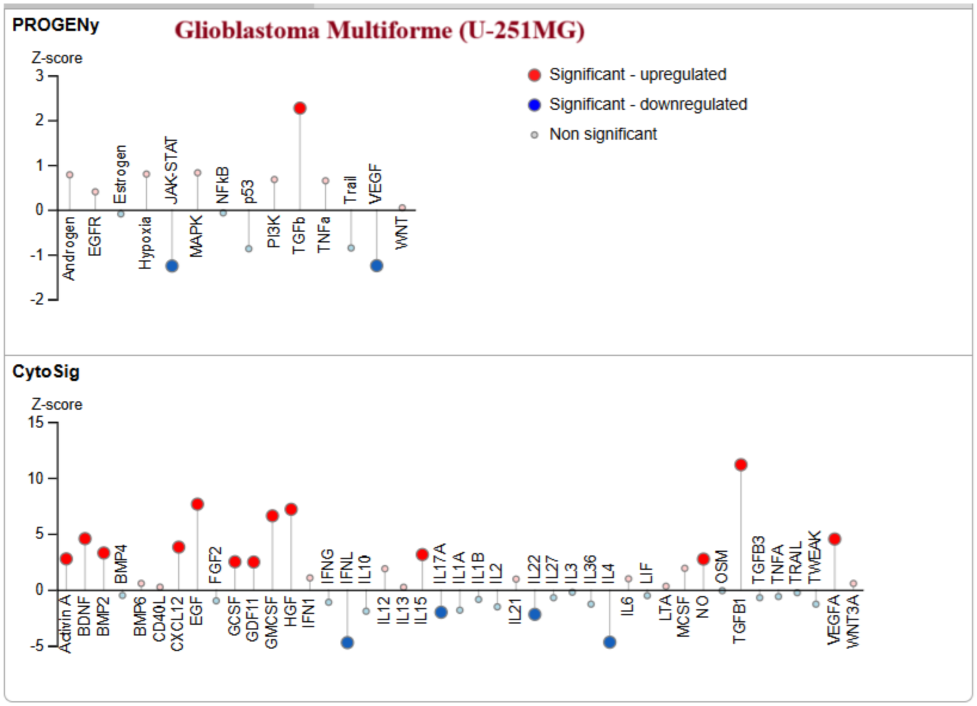
PROGENy
Glioblastoma Multiforme (U-251MG)
Z-score
Significant - upregulated
Significant - downregulated
Non significant
Androgen
EGFR
Estrogen
Hypoxia
JAK-STAT
MAPK
NFkB
p53
PI3K
TGFb
TNFa
Trail
VEGF
WNT
CytoSig
Z-score
Activin A
BDNF
BMP2
BMP4
BMP6
CD40L
CXCL12
EGF
FGF2
GCSF
GDF11
GMCSF
HGF
IFN1
IFNG
IFNL
IL10
IL12
IL13
IL15
IL17A
IL1A
IL1B
IL2
IL21
IL22
IL27
IL3
IL36
IL4
IL6
LIF
LTA
MCSF
NO
OSM
TGFB1
TGFB3
TNFA
TRAIL
TWEAK
VEGFA
WNT3A

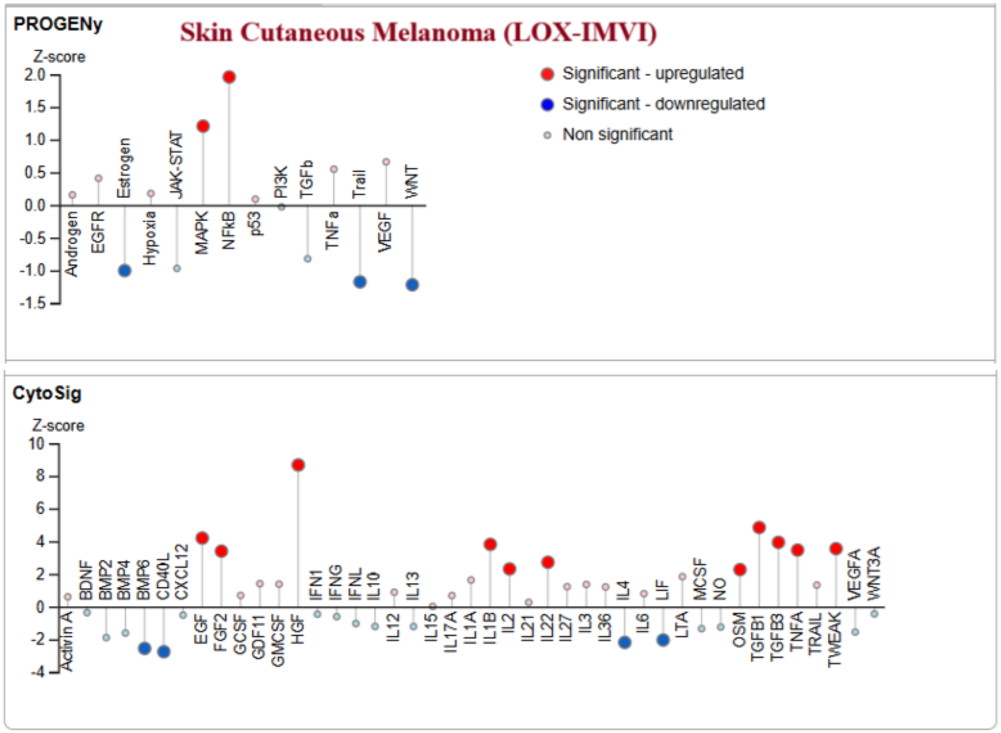
PROGENy
Skin Cutaneous Melanoma (LOX-IMVI)
Z-score
Significant - upregulated
Significant - downregulated
Non significant
Androgen
EGFR
Estrogen
Hypoxia
JAK-STAT
MAPK
NFkB
p53
PI3K
TGFb
TNFa
Trail
VEGF
WNT
CytoSig
Z-score
Activin A
BDNF
BMP2
BMP4
BMP6
CD40L
CXCL12
EGF
FGF2
GCSF
GDF11
GMCSF
HGF
IFN1
IFNG
IFNL
IL10
IL12
IL13
IL15
IL17A
IL1A
IL1B
IL2
IL21
IL22
IL27
IL3
IL36
IL4
IL6
LIF
LTA
MCSF
NO
OSM
TGFB1
TGFB3
TNFA
TRAIL
TWEAK
VEGFA
WNT3A

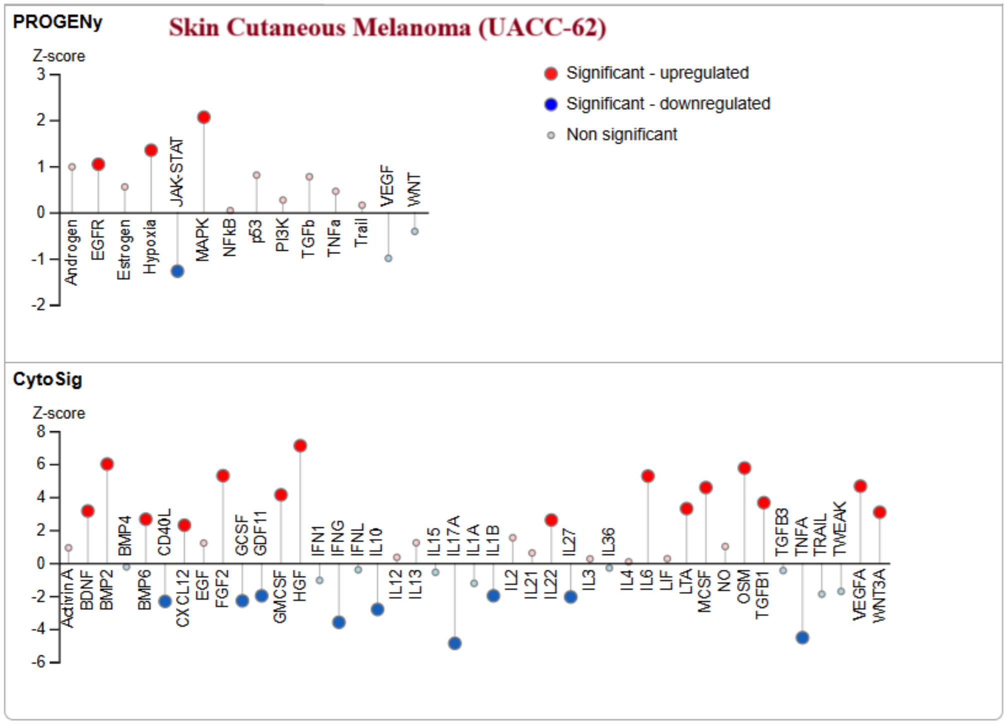
PROGENy
Skin Cutaneous Melanoma (UACC-62)
Z-score
Significant - upregulated
Significant - downregulated
Non significant
Androgen
EGFR
Estrogen
Hypoxia
JAK-STAT
MAPK
NFkB
p53
PI3K
TGFb
TNFa
Trail
VEGF
WNT
CytoSig
Z-score
Activin A
BDNF
BMP2
BMP4
BMP6
CD40L
CXCL12
EGF
FGF2
GCSF
GDF11
GMCSF
HGF
IFN1
IFNG
IFNL
IL10
IL12
IL13
IL15
IL17A
IL1A
IL1B
IL2
IL21
IL22
IL27
IL3
IL36
IL4
IL6
LIF
LTA
MCSF
NO
OSM
TGFB1
TGFB3
TNFA
TRAIL
TWEAK
VEGFA
WNT3A

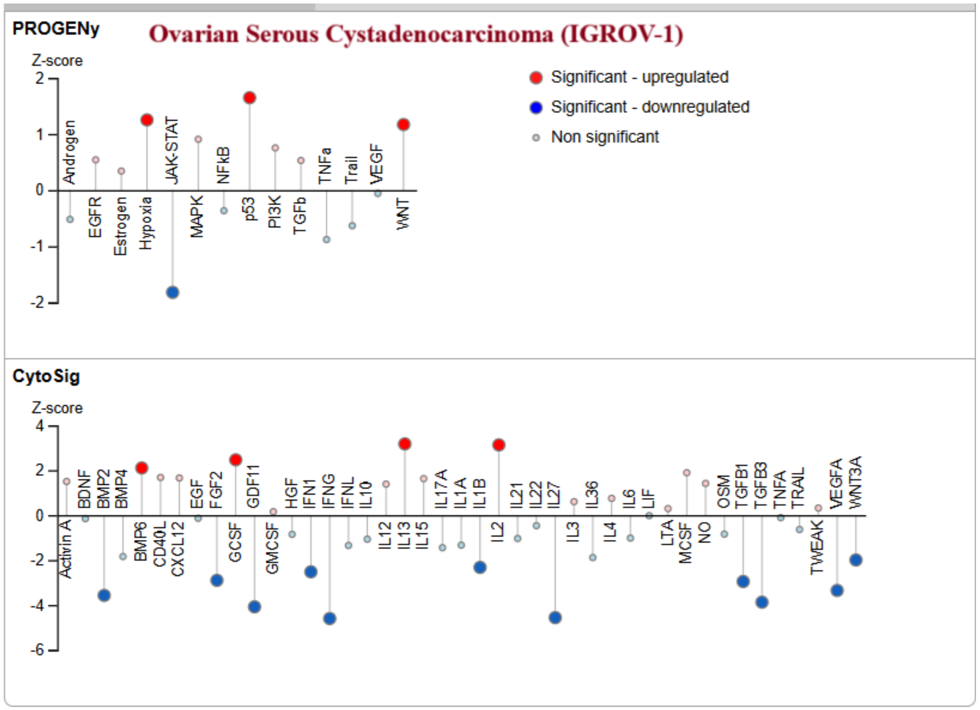
PROGENy
Ovarian Serous Cystadenocarcinoma (IGROV-1)
Z-score
Significant - upregulated
Significant - downregulated
Non significant
Androgen EGFR Estrogen Hypoxia JAK-STAT MAPK NFkB p53 PI3K TGFb TNFa Trail VEGF WNT
CytoSig
Z-score
Activin A BDNF BMP2 BMP4 BMP6 CD40L CXCL12 EGF FGF2 GCSF GDF11 GMCSF HGF IFN1 IFNG IFNL IL10 IL12 IL13 IL15 IL17A IL1A IL1B IL2 IL21 IL22 IL27 IL3 IL36 IL4 IL6 LIF LTA MCSF NO OSM TGFB1 TGFB3 TNFA TRAIL TWEAK VEGFA WNT3A

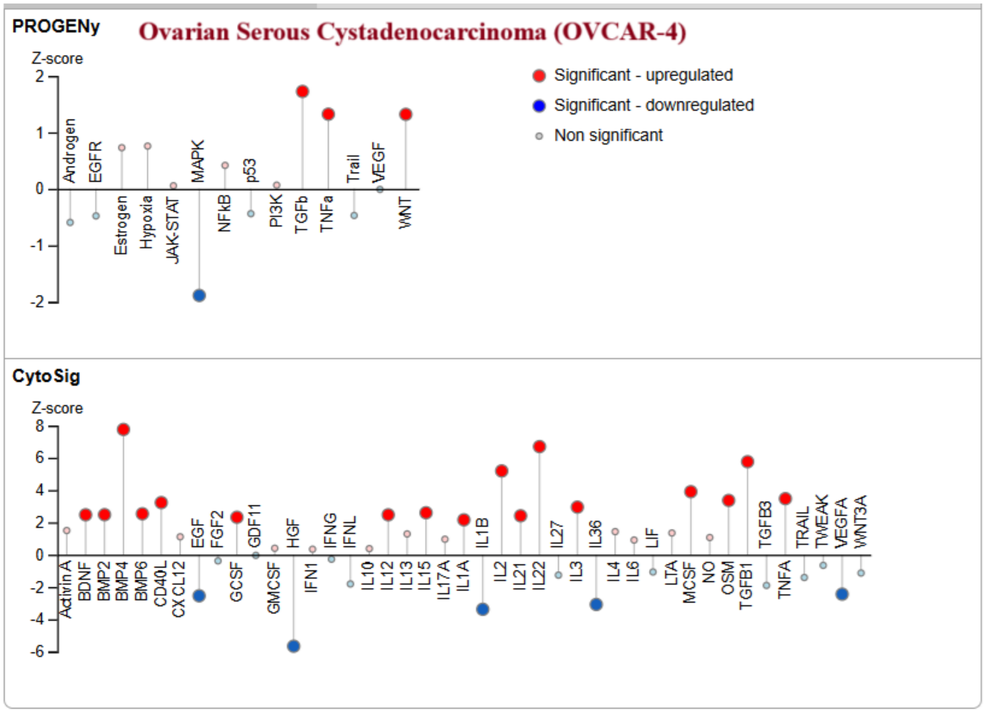
PROGENy
Ovarian Serous Cystadenocarcinoma (OVCAR-4)
Z-score
Significant - upregulated
Significant - downregulated
Non significant
Androgen EGFR Estrogen Hypoxia JAK-STAT MAPK NFkB p53 PI3K TGFb TNFa Trail VEGF WNT
CytoSig
Z-score
Activin A BDNF BMP2 BMP4 BMP6 CD40L CXCL12 EGF FGF2 GCSF GDF11 GMCSF HGF IFN1 IFNG IFNL IL10 IL12 IL13 IL15 IL17A IL1A IL1B IL2 IL21 IL22 IL27 IL3 IL36 IL4 IL6 LIF LTA MCSF NO OSM TGFB1 TGFB3 TNFA TRAIL TWEAK VEGFA WNT3A

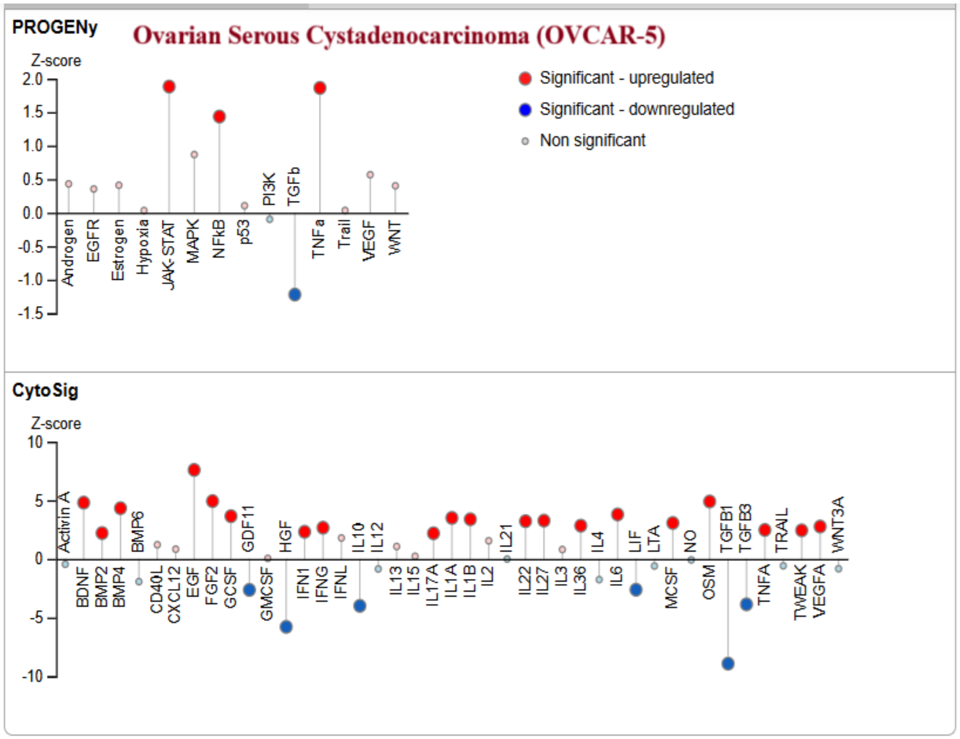
PROGENy
Ovarian Serous Cystadenocarcinoma (OVCAR-5)
Z-score
2.0
1.5
1.0
0.5
0.0
-0.5
-1.0
-1.5
Significant - upregulated
Significant - downregulated
Non significant
Androgen
EGFR
Estrogen
Hypoxia
JAK-STAT
MAPK
NFkB
p53
PI3K
TGFb
TNFa
Trail
VEGF
WNT
CytoSig
Z-score
10
5
0
-5
-10
Activin A
BDNF
BMP2
BMP4
BMP6
CD40L
CXCL12
EGF
FGF2
GCSF
GDF11
GMCSF
HGF
IFN1
IFNG
IFNL
IL10
IL12
IL13
IL15
IL17A
IL1A
IL1B
IL2
IL21
IL22
IL27
IL3
IL36
IL4
IL6
LIF
LTA
MCSF
NO
OSM
TGFB1
TGFB3
TNFA
TRAIL
TWEAK
VEGFA
WNT3A

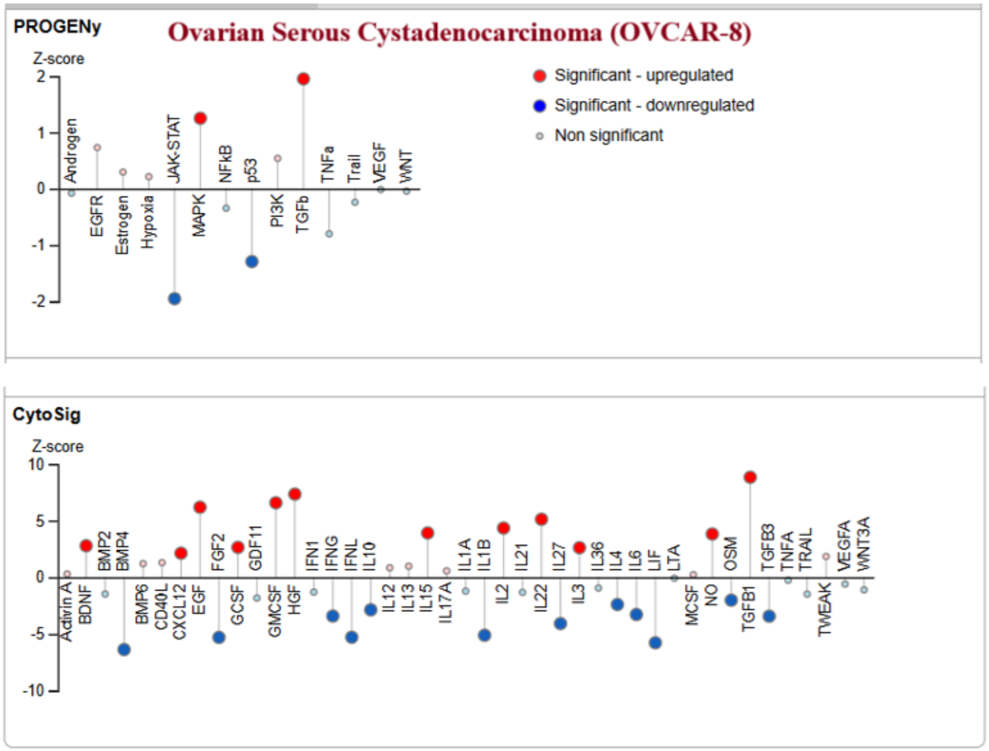
PROGENy
Ovarian Serous Cystadenocarcinoma (OVCAR-8)
Z-score
2
1
0
-1
-2
Significant - upregulated
Significant - downregulated
Non significant
Androgen
EGFR
Estrogen
Hypoxia
JAK-STAT
MAPK
NFkB
p53
PI3K
TGFb
TNFa
Trail
VEGF
WNT
CytoSig
Z-score
10
5
0
-5
-10
Activin A
BDNF
BMP2
BMP4
BMP6
CD40L
CXCL12
EGF
FGF2
GCSF
GDF11
GMCSF
HGF
IFN1
IFNG
IFNL
IL10
IL12
IL13
IL15
IL17A
IL1A
IL1B
IL2
IL21
IL22
IL27
IL3
IL36
IL4
IL6
LIF
LTA
MCSF
NO
OSM
TGFB1
TGFB3
TNFA
TRAIL
TWEAK
VEGFA
WNT3A

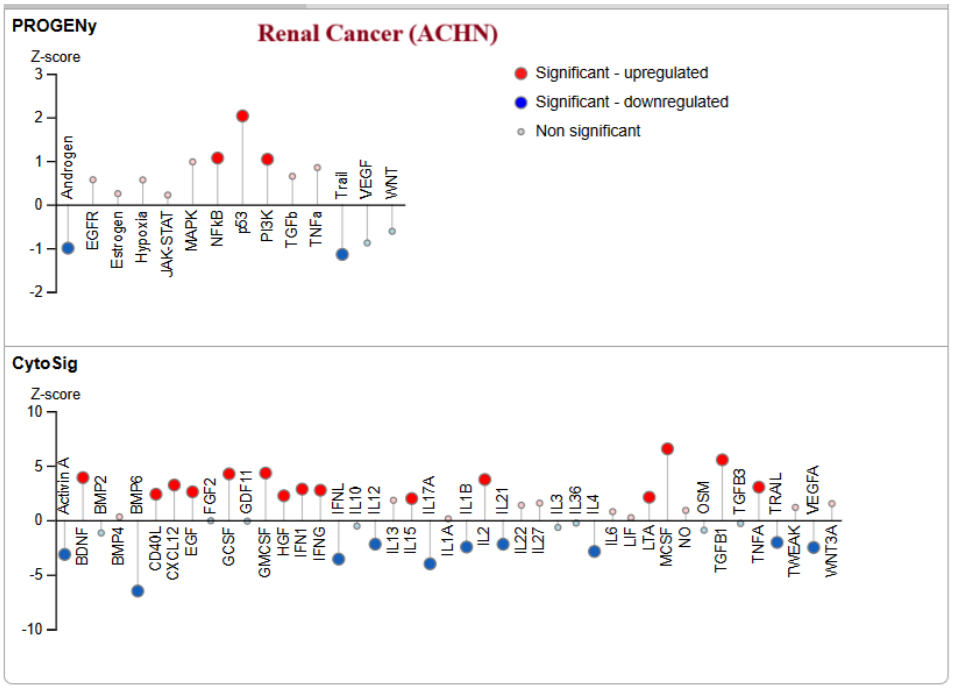
PROGENy
Renal Cancer (ACHN)
Z-score
3
2
1
0
-1
-2
Androgen
EGFR
Estrogen
Hypoxia
JAK-STAT
MAPK
NFkB
p53
PI3K
TGFb
TNFa
Trail
VEGF
WNT
Significant - upregulated
Significant - downregulated
Non significant
CytoSig
Z-score
10
5
0
-5
-10
ActivinA
BDNF
BMP2
BMP4
BMP6
CD40L
CXCL12
EGF
FGF2
GCSF
GDF11
GMCSF
HGF
IFN1
IFNG
IFNL
IL10
IL12
IL13
IL15
IL17A
IL1A
IL1B
IL2
IL21
IL22
IL27
IL3
IL36
IL4
IL6
LIF
LTA
MCSF
NO
OSM
TGFB1
TGFB3
TNFA
TRAIL
TWEAK
VEGFA
WNT3A

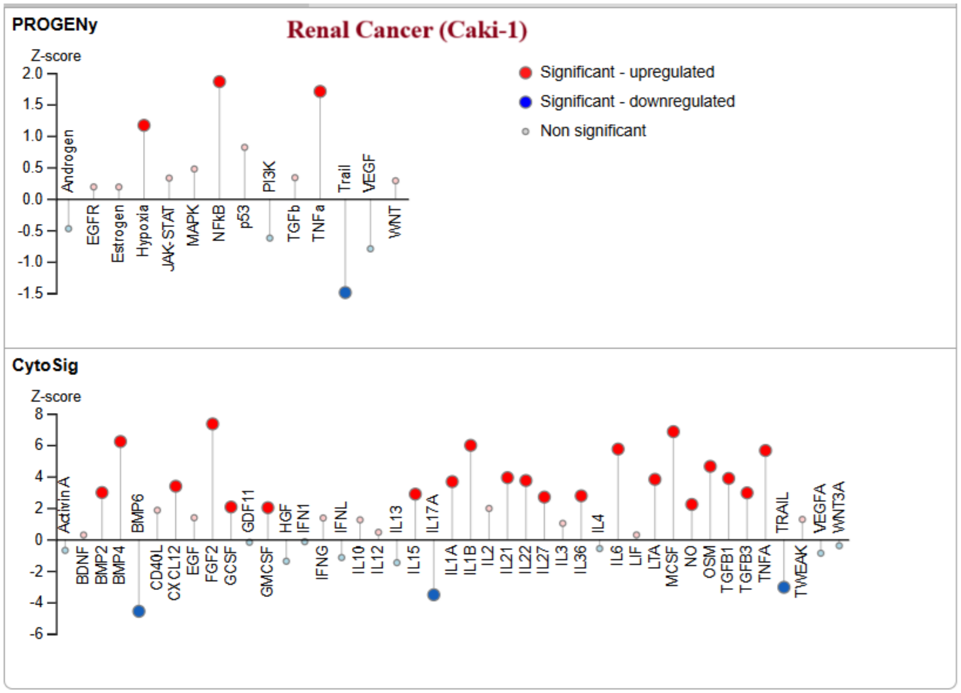
PROGENy
Renal Cancer (Caki-1)
Z-score
2.0
1.5
1.0
0.5
0.0
-0.5
-1.0
-1.5
Androgen
EGFR
Estrogen
Hypoxia
JAK-STAT
MAPK
NFkB
p53
PI3K
TGFb
TNFa
Trail
VEGF
WNT
Significant - upregulated
Significant - downregulated
Non significant
CytoSig
Z-score
8
6
4
2
0
-2
-4
-6
ActivinA
BDNF
BMP2
BMP4
BMP6
CD40L
CXCL12
EGF
FGF2
GCSF
GDF11
GMCSF
HGF
IFN1
IFNG
IFNL
IL10
IL12
IL13
IL15
IL17A
IL1A
IL1B
IL2
IL21
IL22
IL27
IL3
IL36
IL4
IL6
LIF
LTA
MCSF
NO
OSM
TGFB1
TGFB3
TNFA
TRAIL
TWEAK
VEGFA
WNT3A

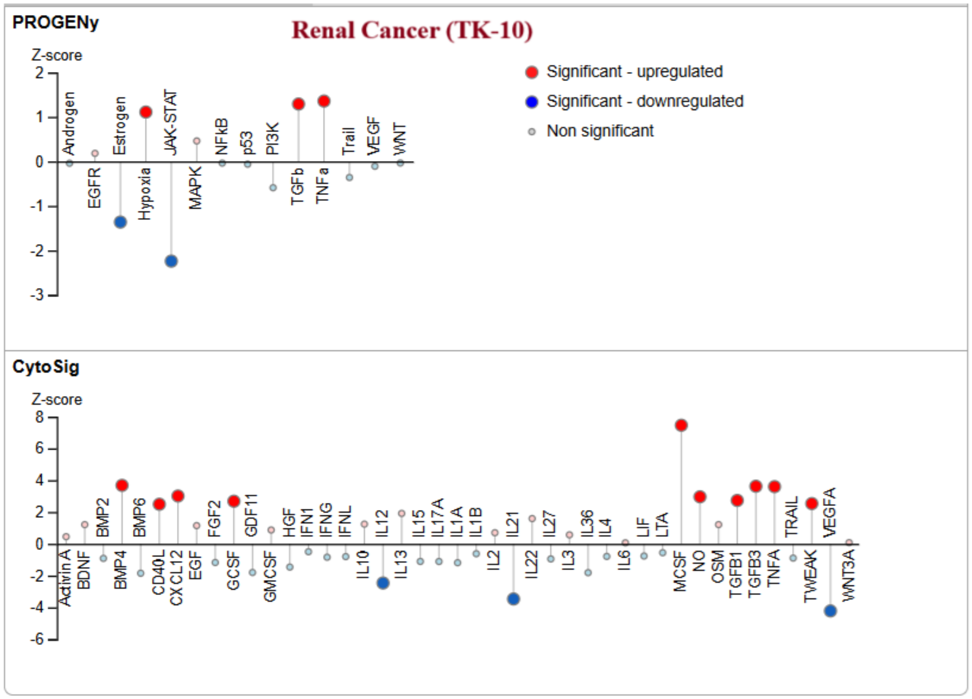
PROGENy
Renal Cancer (TK-10)
Z-score
Significant - upregulated
Significant - downregulated
Non significant
Androgen EGFR Estrogen Hypoxia JAK-STAT MAPK NFkB p53 PI3K TGFb TNFa Trail VEGF WNT
CytoSig
Z-score
Activin A BDNF BMP2 BMP4 BMP6 CD40L CXCL12 EGF FGF2 GCSF GDF11 GMCSF HGF IFN1 IFNG IFNL IL10 IL12 IL13 IL15 IL17A IL1A IL1B IL2 IL21 IL22 IL27 IL3 IL36 IL4 IL6 LIF LTA MCSF NO OSM TGFB1 TGFB3 TNFA TRAIL TWEAK VEGFA WNT3A

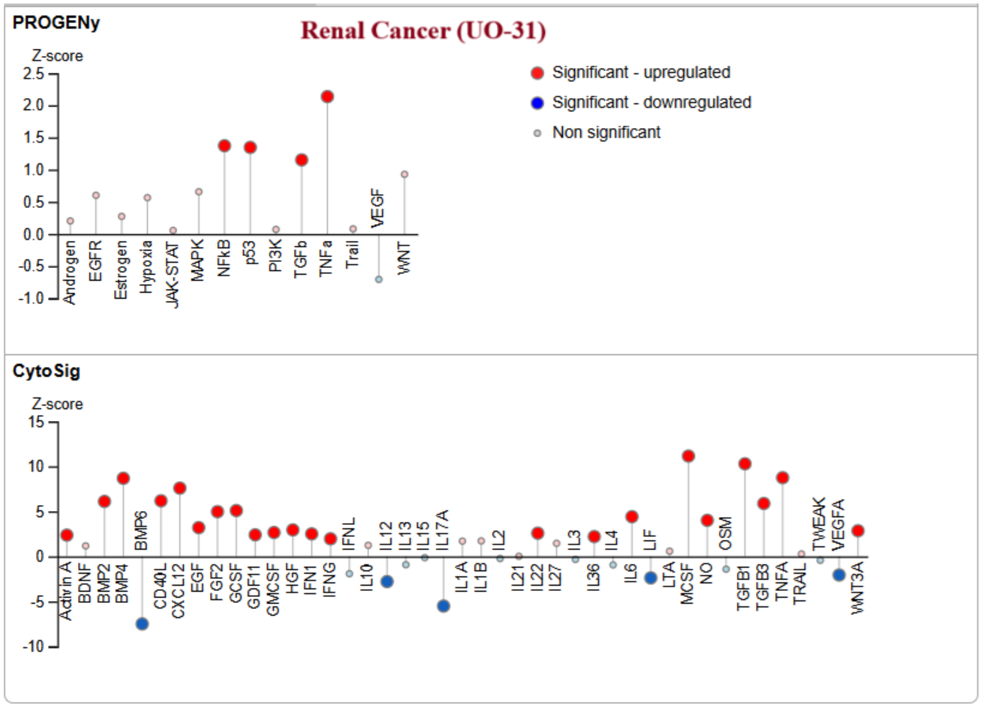
PROGENy
Renal Cancer (UO-31)
Z-score
Significant - upregulated
Significant - downregulated
Non significant
Androgen EGFR Estrogen Hypoxia JAK-STAT MAPK NFkB p53 PI3K TGFb TNFa Trail VEGF WNT
CytoSig
Z-score
Activin A BDNF BMP2 BMP4 BMP6 CD40L CXCL12 EGF FGF2 GCSF GDF11 GMCSF HGF IFN1 IFNG IFNL IL10 IL12 IL13 IL15 IL17A IL1A IL1B IL2 IL21 IL22 IL27 IL3 IL36 IL4 IL6 LIF LTA MCSF NO OSM TGFB1 TGFB3 TNFA TRAIL TWEAK VEGFA WNT3A

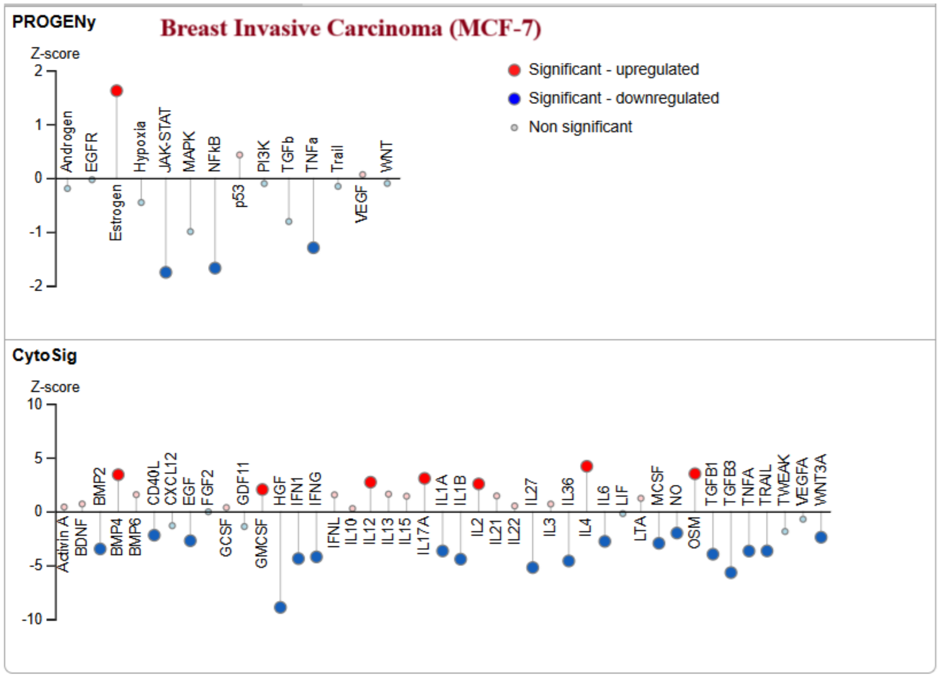
PROGENy
Breast Invasive Carcinoma (MCF-7)
Z-score
Significant - upregulated
Significant - downregulated
Non significant
Androgen
EGFR
Estrogen
Hypoxia
JAK-STAT
MAPK
NFkB
p53
PI3K
TGFb
TNFa
Trail
VEGF
WNT
CytoSig
Z-score
Activin A
BDNF
BMP2
BMP4
BMP6
CD40L
CXCL12
EGF
FGF2
GCSF
GDF11
GMCSF
HGF
IFN1
IFNG
IFNL
IL10
IL12
IL13
IL15
IL17A
IL1A
IL1B
IL2
IL21
IL22
IL27
IL3
IL36
IL4
IL6
LIF
LTA
MCSF
NO
OSM
TGFB1
TGFB3
TNFA
TRAIL
TWEAK
VEGFA
WNT3A

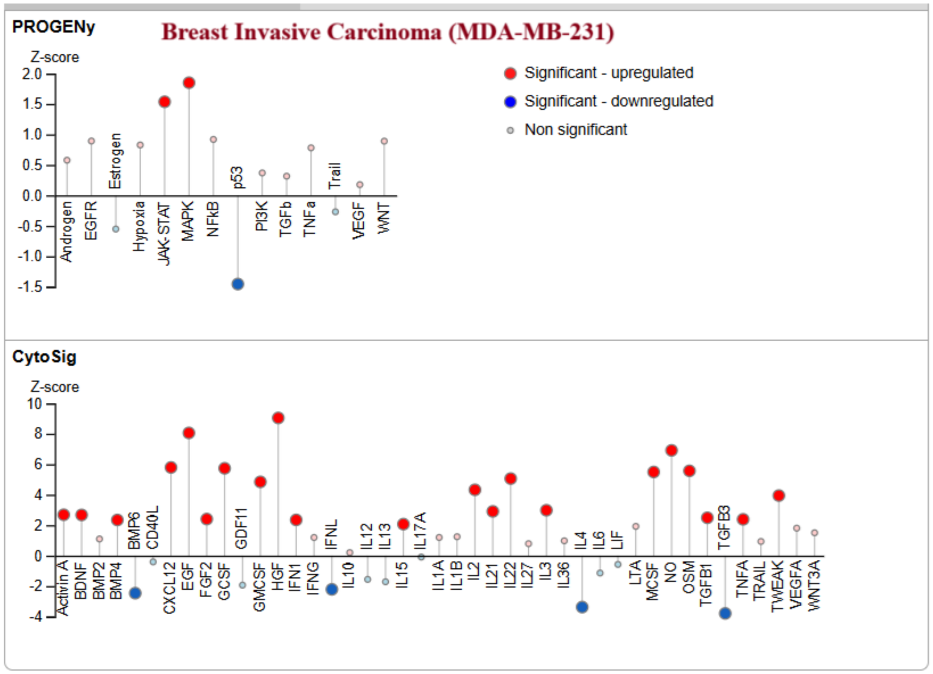
PROGENy
Breast Invasive Carcinoma (MDA-MB-231)
Z-score
Significant - upregulated
Significant - downregulated
Non significant
Androgen
EGFR
Estrogen
Hypoxia
JAK-STAT
MAPK
NFkB
p53
PI3K
TGFb
TNFa
Trail
VEGF
WNT
CytoSig
Z-score
Activin A
BDNF
BMP2
BMP4
BMP6
CD40L
CXCL12
EGF
FGF2
GCSF
GDF11
GMCSF
HGF
IFN1
IFNG
IFNL
IL10
IL12
IL13
IL15
IL17A
IL1A
IL1B
IL2
IL21
IL22
IL27
IL3
IL36
IL4
IL6
LIF
LTA
MCSF
NO
OSM
TGFB1
TGFB3
TNFA
TRAIL
TWEAK
VEGFA
WNT3A

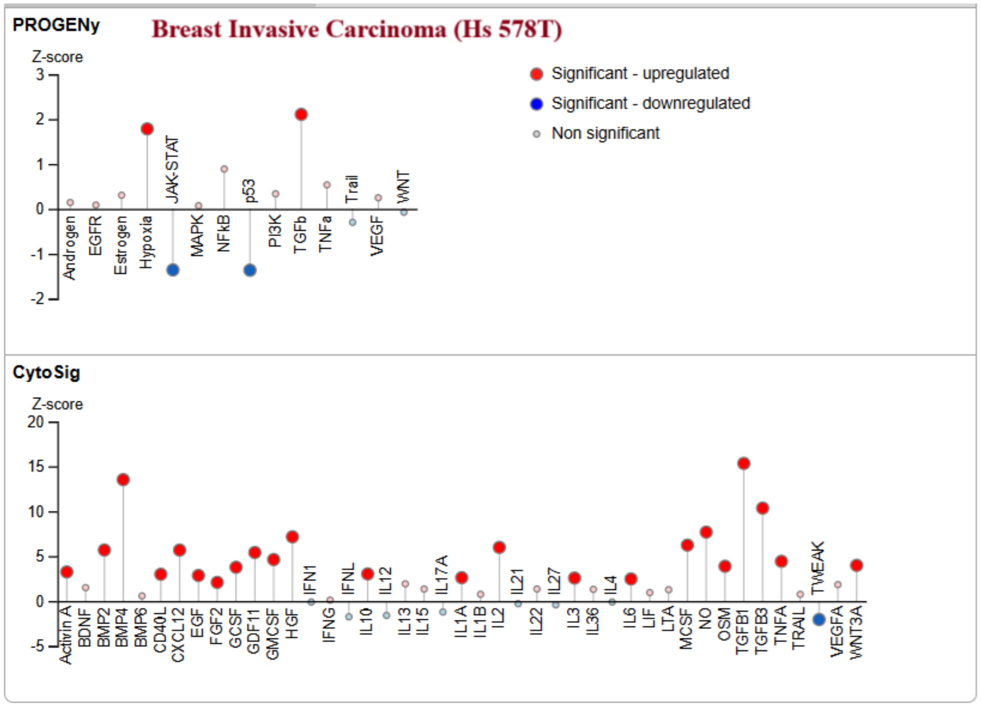
PROGENy
Breast Invasive Carcinoma (Hs 578T)
Z-score
Significant - upregulated
Significant - downregulated
Non significant
Androgen
EGFR
Estrogen
Hypoxia
JAK-STAT
MAPK
NFkB
p53
PI3K
TGFb
TNFa
Trail
VEGF
WNT
CytoSig
Z-score
Activin A
BDNF
BMP2
BMP4
BMP6
CD40L
CXCL12
EGF
FGF2
GCSF
GDF11
GMCSF
HGF
IFN1
IFNG
IFNL
IL10
IL12
IL13
IL15
IL17A
IL1A
IL1B
IL2
IL21
IL22
IL27
IL3
IL36
IL4
IL6
LIF
LTA
MCSF
NO
OSM
TGFB1
TGFB3
TNFA
TRAIL
TWEAK
VEGFA
WNT3A

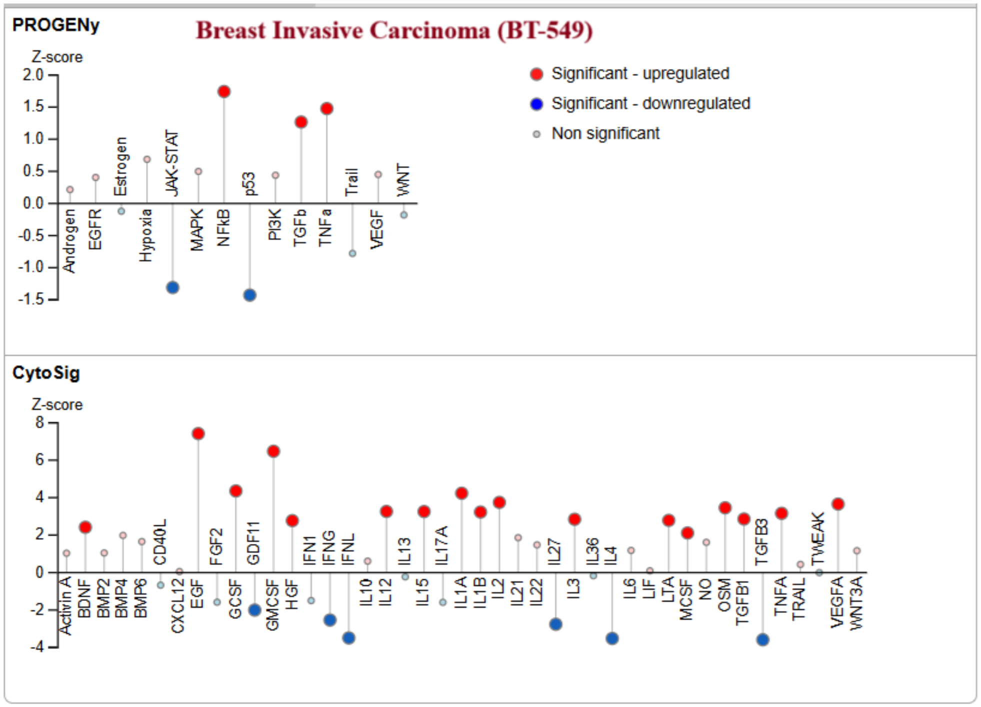
PROGENy
Breast Invasive Carcinoma (BT-549)
Z-score
Significant - upregulated
Significant - downregulated
Non significant
Androgen
EGFR
Estrogen
Hypoxia
JAK-STAT
MAPK
NFkB
p53
PI3K
TGFb
TNFa
Trail
VEGF
WNT
CytoSig
Z-score
Activin A
BDNF
BMP2
BMP4
BMP6
CD40L
CXCL12
EGF
FGF2
GCSF
GDF11
GMCSF
HGF
IFN1
IFNG
IFNL
IL10
IL12
IL13
IL15
IL17A
IL1A
IL1B
IL2
IL21
IL22
IL27
IL3
IL36
IL4
IL6
LIF
LTA
MCSF
NO
OSM
TGFB1
TGFB3
TNFA
TRAIL
TWEAK
VEGFA
WNT3A

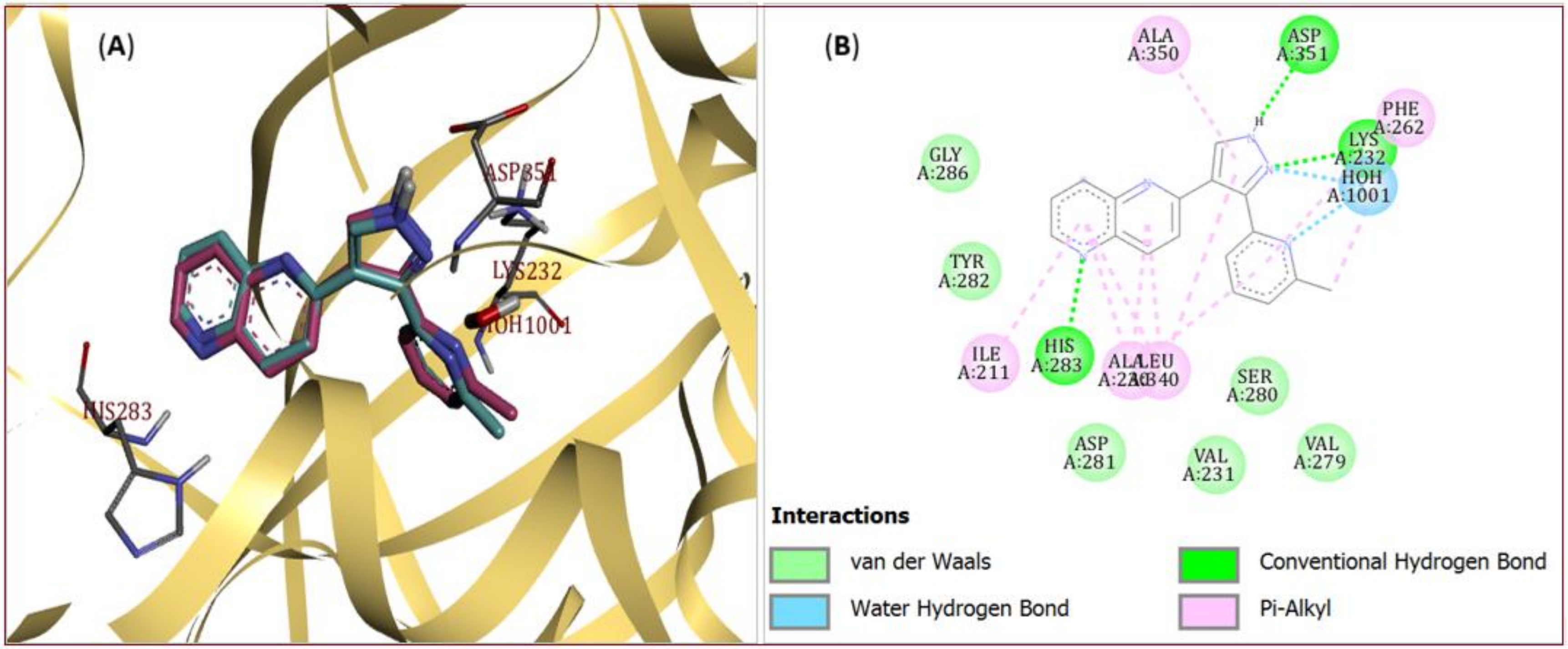

Figure S2. (**A**) 3D representation of the superimposition of docking pose (cyan) and the co-crystallized pose (mulberry) of RepSox (E-616452) in the TGF-β type I receptor active site. (B) 2D interaction diagrams showing RepSox docking pose interactions with the key amino acids in the TGF-β type I receptor active site (PDB ID: 1VJY).

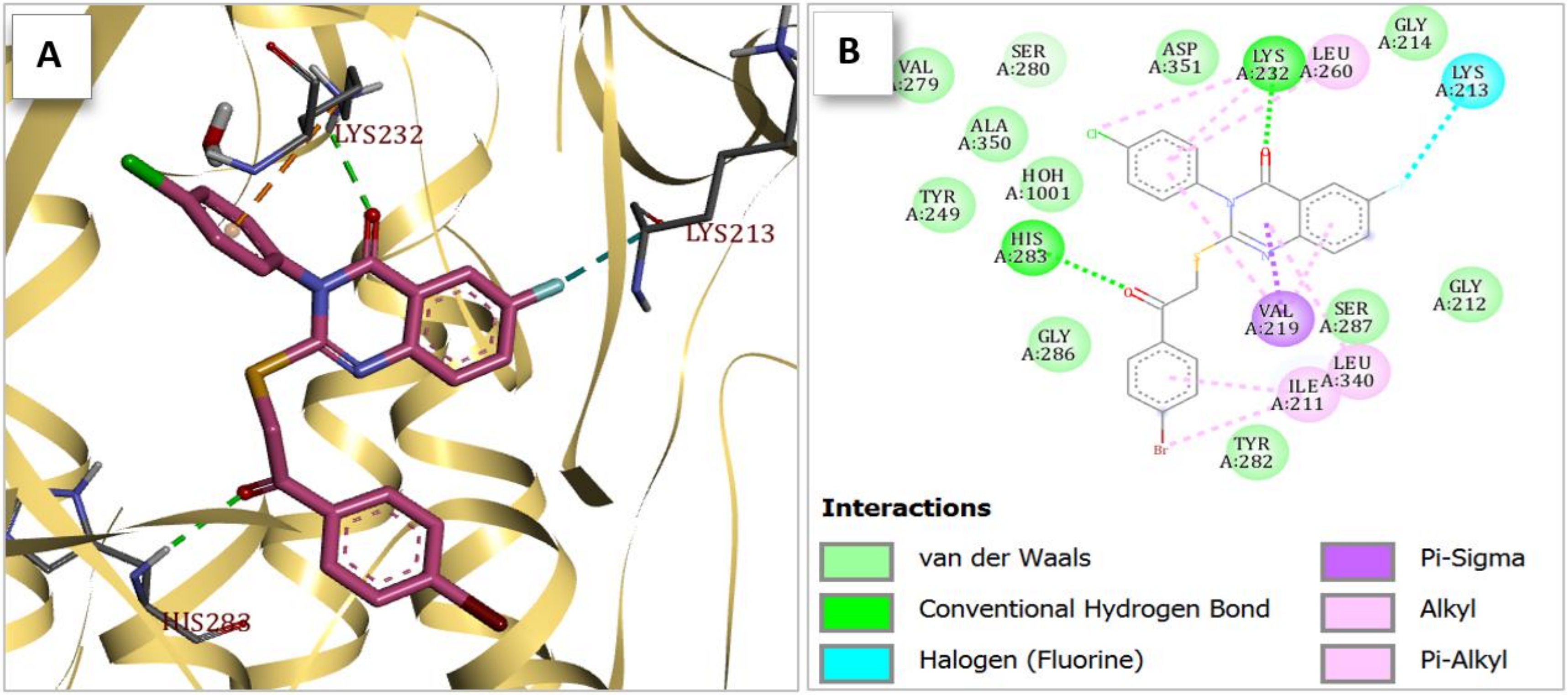

Figure S3. (**A**) 3D and (**B**) 2D interaction diagrams showing compound **3e** docking pose within the TGF-β type I receptor kinase domain (PDB ID: 1VJY).

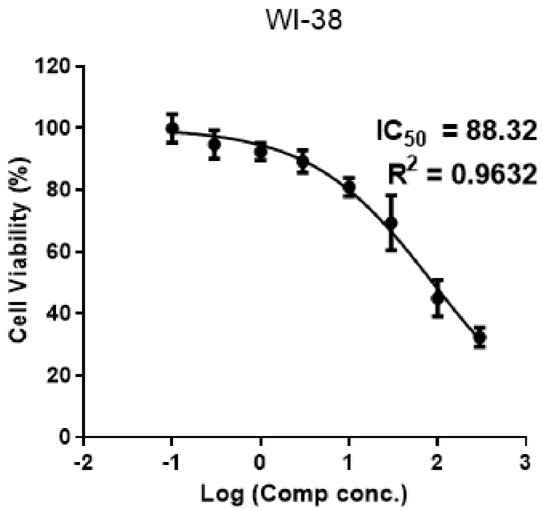

Figure S4. Graph of log(concentration) of compound **3e** versus the percentage viability of normal human lung fibroblast WI-38 cells.

S3.2. Biological evaluation

S3.2.1. Anticancer screening

Initially, the anticancer evaluation of the studied compounds was performed by the National Cancer Institute (NCI), Bethesda, MD, USA, at a single dose (10 μM) in the full NCI 60 Human Tumor Cell Line Panel (derived from different nine neoplastic diseases) assay according to the protocol of the Drug Evaluation Branch, NCI. Compound **3e**, which demonstrated significant growth inhibition in the one-dose screen and satisfied the NCI threshold criterion (at least 8 cell lines exhibiting G% values below 10), was advanced to the standard five-dose evaluation across the 60-cell line panel. The compound was tested at five concentrations spanning $\log_{10}$ −4 to −8 M (10^{-4}–10^{-8} M; 100 to 0.01 μM) [1–4].

S3.2.2. Flow cytometry analysis for cell cycle distribution of **3e**-treated A549 carcinoma cells

Cell cycle distribution of A549 carcinoma cells (Holding company for biological products and vaccines, VACSERA, Giza, Egypt) was evaluated using Propidium Iodide Flow Cytometry Kit for Cell Cycle Analysis (Abcam-UK). Briefly, A549 cells were seeded in six-well plates at a density of 2×10^5 cells per well and incubated for 24 h at 37 °C under 5% CO_2. The cells were treated with compound **3e** (prepared in DMSO, 1% v/v) at its IC_{50} value of 10.8 μM for 72 h, then washed with cold phosphate buffered saline (PBS), fixed in 70% ethanol, and washed again with PBS. The fixed cells were stained with propidium iodide DNA fluorochrome and incubated at 37 °C for 15 minutes in the dark. The DNA levels of the stained cells were measured through flow cytometry analysis and cell cycle distribution was analyzed using an Epics XL-MCL™ Flow Cytometer (Beckman Coulter), with data processing performed using Flowing software (version 2.5.1, Turku Centre for Biotechnology, Turku, Finland). A549 carcinoma cells treated with DMSO served as the negative control group.

S3.2.3. Annexin-V-FITC assay for detection of apoptosis

The distribution of early and late apoptotic cells, as well as necrotic cells in **3e**-treated A549 carcinoma cells culture were detected using Annexin V-FITC/PI Apoptosis Detection Kit (BioVision Research Products, Mountain View, CA, USA). Concisely, A549 cells were plated and incubated for 24 hours, then treated with compound **3e** at a concentration of 10.8 μM for 72 h. Cells were harvested, washed three times with PBS, and fixed using ice-cold 70% ethanol. The fixed cellular preparations were stained with Annexin V-FITC and propidium iodide (PI) and kept in the dark for 20 minutes. Apoptotic cell populations were analyzed using an Epics XL-MCL flow cytometer. A549 cells treated with vehicle (DMSO) served as the control.

S3.2.4. Quantitative reverse transcription-polymerase chain reaction (qRT-PCR) for analyzing the expression levels of apoptotic markers; *Bax*, *Bcl-2*, *Caspase-9*, and *Caspase-3* genes in **3e**-treated A549 cells

The Real-time PCR was used to assess the expressional levels of *Bax*, *Bcl-2*, *Caspase-9*, and *Caspase-3* mRNA in **3e**-treated/untreated A549 cells as follows: cells were seeded in T-25 cm^2 tissue culture flasks 24 h before treatment with DMSO vehicle (0.01%) or compound **3e** at a concentration of 10.8 μM for an additional 72 h. Total RNA from both samples was extracted from both untreated and treated A549 cells as per the manufacturer's instructions (RNeasy mini kit, Qiagen, Germany. Amplification of target cDNA for apoptosis markers and GAPDH [as a normalization (housekeeping) gene] was done using the one-step RT-PCR SYBR® Green kit Master Mix (Bio-Rad Laboratories, USA) on Rotor-Gene Q real-time PCR thermal cycler instrument (Qiagen, USA). cDNA (2 μL aliquots) was mixed with 1 μL of forward primer, 1 μL reverse primer (Table S3), 10 μL master mixture, and the reaction volume was completed to 20 μL with nuclease-free water. Glyceraldehyde 3-phosphate dehydrogenase (GAPDH) was the internal reference.

The $2^{-\Delta\Delta Ct}$ method [5] was used to calculate the relative expression of the target genes, and the results were statistically analyzed. All experiments were performed in triplicates. Data obtained from three independent experiments, are presented as a ratio of target gene/GAPDH expression (relative mRNA levels) and represent the mean ± SEM. Normalized data are expressed as the fold changes, with the control set to '1'.

*p < 0.05, **p < 0.01 and ***p < 0.001 indicate statistically significant differences from the corresponding control in unpaired t-tests.

S3.2.5. In vitro anti-proliferative activities of compound **3e** against human normal lung fibroblast WI-38 cells

The cytotoxicity of the most active anticancer candidate **3e** on normal WI-38 fibroblasts was evaluated using 3-(4,5-dimethylthiazol-2yl)-2,5- diphenyltetrazolium bromide (MTT) assay [6]. Concisely, 2–2.2 × 10^4 cells were added to each well of a 96-well tissue culture plate containing the Eagle's modified minimum essential media (EMEM) supplemented with 10% FBS and antibiotics (100 units/mL penicillin and 100 μg/mL streptomycin). Cells were grown for 24 h at 37 °C in a 5% CO_2 incubator. Compound stock solution was prepared in DMSO. Eight concentrations (300, 100, 30, 10, 3, 1, 0.3, and 0.1 μM) were prepared for **3e** in the growth media, and cells were treated for 72 h. Freshly prepared MTT salt (5 mg/mL; Sigma) was then added to each well to give a final concentration of 0.5 μg/μL. The MTT solution was carefully removed and an equal volume of (200 μL) DMSO was added to each well and incubated for 30-45 min while shaking. Cell proliferation was detected by measuring the absorbance of each well at 590 nm using Multiskan® EX (Thermo Scientific, USA) MicroPlate Reader. The experiment was performed three times in triplicates. The half maximal inhibitory concentration (IC_{50}) value was determined using non-linear regression analysis of data set obtained from three independent experiments, each performed in triplicate wells for each concentration, using GraphPad Prism version 8.0 (GraphPad Software, San Diego, CA, USA).

S3.2.6. In vitro TGF-βRI (ALK5) kinase inhibition assay

The TGF-βRI (ALK5) Kinase Assay Kit (BPS Bioscience, Cat. #78819, 96 reactions) was used to determine the IC_{50} value of compound **3e** for ALK5 inhibition according to the manufacturer's protocol. The assay measures ALK5 serine/threonine kinase activity using an ADP-Glo® luminescent detection system in a 96-well format. Briefly, the test compound, dissolved in DMSO, was diluted in kinase buffer and added to a 96-well plate. Recombinant TGF-βRI enzyme was then added, and the mixture was pre-incubated for 10–15 min at room temperature. The kinase reaction was initiated by adding substrate and ATP solution, followed by incubation for 30–60 min at 30 °C. After completion of the reaction, ADP-Glo reagent was added to terminate the kinase reaction and deplete the remaining ATP. Subsequently, kinase detection reagent was added, and the plate was incubated at room temperature for 30 min. Luminescence was measured using a microplate reader. The IC_{50} value, defined as the compound concentration required to inhibit 50% of kinase activity relative to control wells, was determined for compound **3e** and **A83-01** (used as a reference inhibitor) using multiple concentrations prepared by serial dilution. The percentage inhibition of kinase activity was calculated relative to vehicle control wells at each concentration, and dose-response curves were generated by plotting TGF-βRI kinase activity (%) versus the logarithm of compound concentration (μM). IC_{50} values were calculated by nonlinear regression analysis of the dose–response curves.

3.3. In silico studies

3.3.1. Molecular docking studies

The molecular docking study of the promising congener **3e** was conducted to evaluate its binding affinity for the predicted anticancer target, the TGF-β type I receptor kinase domain. The target compound **3e** was drawn using Marvin Sketch (https://chemaxon.com/marvin), converted to a 3D structure, and energy-minimized using Avogadro software [7]. Meanwhile, the X-ray co-crystallographic structure of the TGF-β type I receptor in complex with the pyrazole derivative E-616452 (PDB ID: 1VJY) was retrieved from the RCSB Protein Data Bank (https://www.rcsb.org/). Using Discovery Studio Visualizer, all water molecules, except for W1001, were removed. The 'Dock Prep' function in UCSF Chimera v1.17.3 [8] was then used to

prepare both the protein and **3e** for docking and validation, adding hydrogen atoms, assigning Gasteiger charges, and performing minimization via the steepest descent and conjugate gradient algorithms. Docking simulations were carried out with AutoDock Vina [9,10], setting the number of iterations to 100. A grid box was defined based on the coordinates of the co-crystallized ligand in the PDB file. Analysis of protein–ligand interactions, including hydrogen bonding, hydrophobic, and Van der Waals interactions, was performed using Discovery Studio 2021.

The docking protocol was preliminarily validated by redocking the co-crystallized ligand (E-616452) into the active site, yielding a docking pose with an energy score (S) of −10.3 kcal/mol and an RMSD [11] of 0.323 Å relative to the crystallized ligand pose (Figure S2-A). The redocked pose revealed the same binding pattern with the hotspot amino acids Lys232, His283, and Asp351 through hydrogen bonding. Additionally, the pyridyl-*N* was engaged in a water hydrogen bond with HOH1001. It also displayed hydrophobic interactions with Ile211, Ala230, Phe262, Leu340 and Ala350 amino acids (Figure S2-B).

S3.3.2. Molecular dynamics (MD) simulations

MD simulation was carried out using GROMACS 2025.2 to assess the stability of compound **3e** in the TGF-βI binding site [12]. System preparation included generating topology files for both the protein and ligand using the Amber ff99SB force field [13]. The system was placed in a triclinic box with a 1 nm buffer around the solute and solvated using the TIP3P water model. Counterions were added to neutralize the system. Energy minimization was performed using the steepest descent algorithm (maximum 50,000 steps) until the maximum force dropped below 1000 $kJ \cdot mol^{-1} \cdot nm^{-1}$. Equilibration proceeded in two phases: a 100 ps NVT phase to stabilize temperature at 300 K using the modified Berendsen thermostat (V-rescale, $\tau = 0.1$ ps) [14], followed by a 100 ps NPT phase to equilibrate pressure at 1 bar using Berendsen pressure coupling ($\tau = 2$ ps, isotropic) [15]. During both phases, positional restraints were applied to all atoms except solvent molecules. Subsequently, a 100 ns production run was executed under periodic boundary conditions. Throughout the production phase, the system utilized the V-rescale thermostat featuring a time constant of 0.1 ps along with the Parrinello–Rahman barostat with a time constant of 2 ps [16,17]. The long-range electrostatic interactions were addressed using the Particle Mesh Ewald (PME) method. Long-range electrostatics were handled alongside short-range van der Waals, both with a cutoff set to 1 nm [18]. Trajectory analyses were performed to evaluate the root mean square deviation (RMSD), root mean square fluctuation (RMSF), and radius of gyration (Rg) to assess the structural stability and compactness of the complex over time [19–21].

S3.3.3. ADME predictions

The PreADME online web tool (https://preadmet.qsarhub.com) was utilized to predict the in silico ADME properties of the most active compound **3e**.

References

1. Shoemaker, R.H. The NCI60 human tumour cell line anticancer drug screen. *Nat. Rev. Cancer* **2006**, *6*, 813–823. https://doi.org/10.1038/nrc1951.
2. Monks, A.; Scudiero, D.; Skehan, P.; Shoemaker, R.; Paull, K.; Vistica, D.; Hose, C.; Langley, J.; Cronise, P.; Vaigro-Wolff, A.; et al. Feasibility of a high-flux anticancer drug screen using a diverse panel of cultured human tumor cell lines. *J. Natl. Cancer Inst.* **1991**, *83*, 757–766. https://doi.org/10.1093/jnci/83.11.757.
3. Grever, M.R.; Schepartz, S.A.; Chabner, B.A. The National Cancer Institute: Cancer drug discovery and development program. *Semin. Oncol.* **1992**, *19*, 622–638.
4. Alley, M.C.; Scudiero, D.A.; Monks, A.; Hursey, M.L.; Czerwinski, M.J.; Fine, D.L.; Abbott, B.J.; Mayo, J.G.; Shoemaker, R.H.; Boyd, M.R. Feasibility of drug screening with panels of human tumor cell lines using a microculture tetrazolium assay. *Cancer Res.* **1988**, *48*, 589–601.

5. Livak, K.J.; Schmittgen, T.D. Analysis of relative gene expression data using real-time quantitative PCR and the $2^{-\Delta\Delta C_T}$ Method. *Methods* **2001**, *25*, 402–408. https://doi.org/10.1006/meth.2001.1262.
6. Mosmann, T. Rapid colorimetric assay for cellular growth and survival: Application to proliferation and cytotoxicity assays. *J. Immunol. Methods* **1983**, *65*, 55–63. https://doi.org/10.1016/0022-1759(83)90303-4.
7. Hanwell, M.D.; Curtis, D.E.; Lonie, D.C.; Vandermeersch, T.; Zurek, E.; Hutchison, G.R. Avogadro: An advanced semantic chemical editor, visualization, and analysis platform. *J. Cheminform.* **2012**, *4*, 17. http://www.jcheminf.com/content/4/1/17.
8. Pettersen, E.F.; Goddard, T.D.; Huang, C.C.; Couch, G.S.; Greenblatt, D.M.; Meng, E.C.; Ferrin, T.E. UCSF Chimera—A visualization system for exploratory research and analysis. *J. Comput. Chem.* **2004**, *25*, 1605–1612. https://doi.org/10.1002/jcc.20084.
9. Eberhardt, J.; Santos-Martins, D.; Tillack, A.F.; Forli, S. AutoDock Vina 1.2.0: New docking methods, expanded force field, and Python bindings. *J. Chem. Inf. Model.* **2021**, *61*, 3891–3898. https://doi.org/10.1021/acs.jcim.1c00203.
10. Trott, O.; Olson, A.J. AutoDock Vina: Improving the speed and accuracy of docking with a new scoring function, efficient optimization, and multithreading. *J. Comput. Chem.* **2010**, *31*, 455–461. https://doi.org/10.1002/jcc.21334.
11. Bell, E.W.; Zhang, Y. DockRMSD: An open-source tool for atom mapping and RMSD calculation of symmetric molecules through graph isomorphism. *J. Cheminform.* **2019**, *11*, 40. https://doi.org/10.1186/s13321-019-0362-7.
12. Abraham, M.J.; Murtola, T.; Schulz, R.; Páll, S.; Smith, J.C.; Hess, B.; Lindahl, E. GROMACS: High performance molecular simulations through multi-level parallelism from laptops to supercomputers. *SoftwareX* **2015**, *1–2*, 19–25. https://doi.org/10.1016/j.softx.2015.06.001.
13. Lindorff-Larsen, K.; Piana, S.; Palmo, K.; Maragakis, P.; Klepeis, J.L.; Dror, R.O.; Shaw, D.E. Improved side-chain torsion potentials for the Amber ff99SB protein force field. *Proteins: Struct. Funct. Bioinform.* **2010**, *78*, 1950–1958. https://doi.org/10.1002/prot.22711.
14. Bussi, G.; Donadio, D.; Parrinello, M. Canonical sampling through velocity rescaling. *J. Chem. Phys.* **2007**, *126*, 014101. https://doi.org/10.1063/1.2408420.
15. Berendsen, H.J.C.; Postma, J.P.M.; van Gunsteren, W.F.; DiNola, A.; Haak, J.R. Molecular dynamics with coupling to an external bath. *J. Chem. Phys.* **1984**, *81*, 3684–3690. https://doi.org/10.1063/1.448118.
16. Hess, B.; Bekker, H.; Berendsen, H.J.C.; Fraaije, J.G.E.M. LINCS: A linear constraint solver for molecular simulations. *J. Comput. Chem.* **1997**, *18*, 1463–1472. https://doi.org/10.1002/(SICI)1096-987X(199709)18:12<1463::AID-JCC4>3.0.CO;2-H.
17. Parrinello, M.; Rahman, A. Polymorphic transitions in single crystals: A new molecular dynamics method. *J. Appl. Phys.* **1981**, *52*, 7182–7190. https://doi.org/10.1063/1.328693.
18. Darden, T.; York, D.; Pedersen, L. Particle mesh Ewald: An N·log(N) method for Ewald sums in large systems. *J. Chem. Phys.* **1993**, *98*, 10089–10092. https://doi.org/10.1063/1.464397.
19. Damm, K.L.; Carlson, H.A. Gaussian-weighted RMSD superposition of proteins: A structural comparison for flexible proteins and predicted protein structures. *Biophys. J.* **2006**, *90*, 4558–4573. https://doi.org/10.1529/biophysj.105.066654.
20. Fuglebakk, E.; Echave, J.; Reuter, N. Measuring and comparing structural fluctuation patterns in large protein datasets. *Bioinformatics* **2012**, *28*, 2431–2440. https://doi.org/10.1093/bioinformatics/bts445.
21. Ali, H.; Hassan, R.M.; El Kerdawy, A.M.; Abo-Elfadl, M.T.; Abdallah, H.M.I.; Sciandra, F.; Ghannam, I.A.Y. Novel thiazolidin-4-one benzenesulfonamide hybrids as PPARγ agonists: Design, synthesis and in vivo anti-diabetic evaluation. *Eur. J. Med. Chem.* **2024**, *269*, 116279. https://doi.org/10.1016/j.ejmech.2024.116279.

Spectroscopic data of selected representative compounds

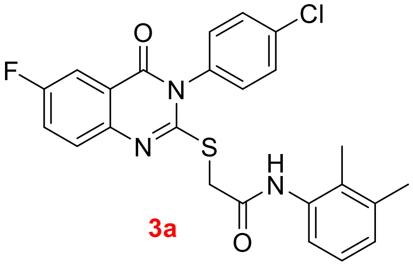

3a

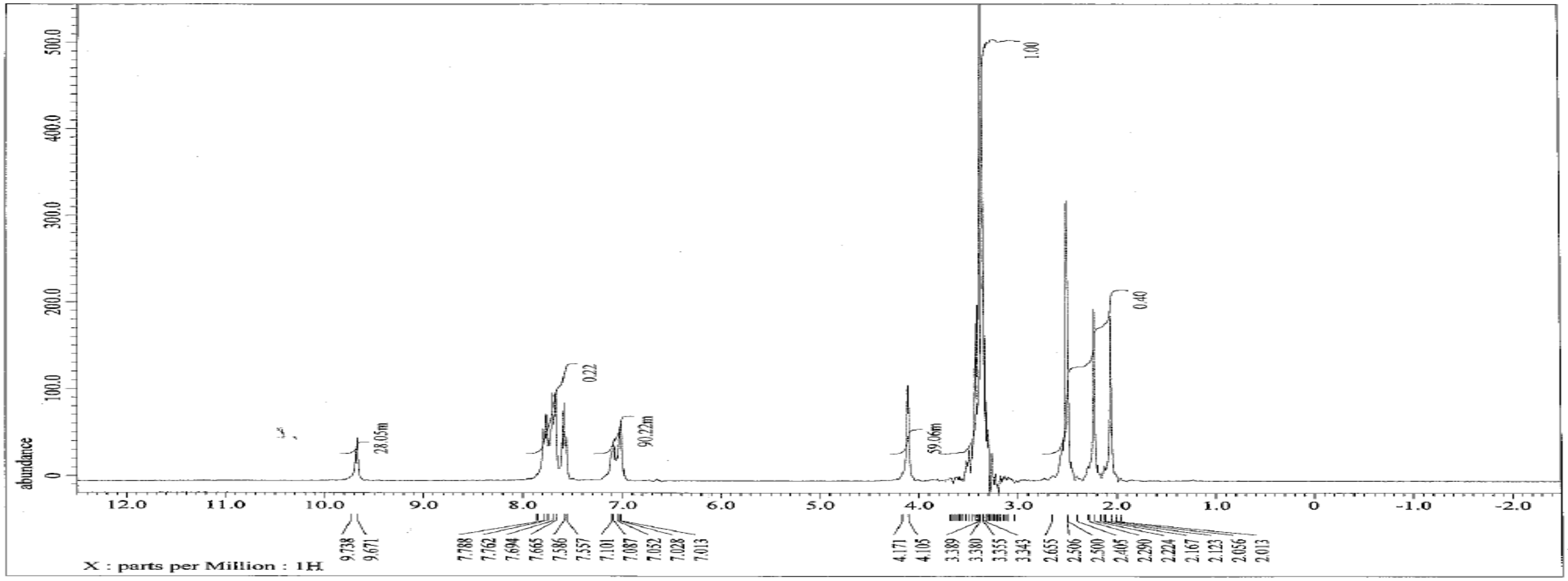

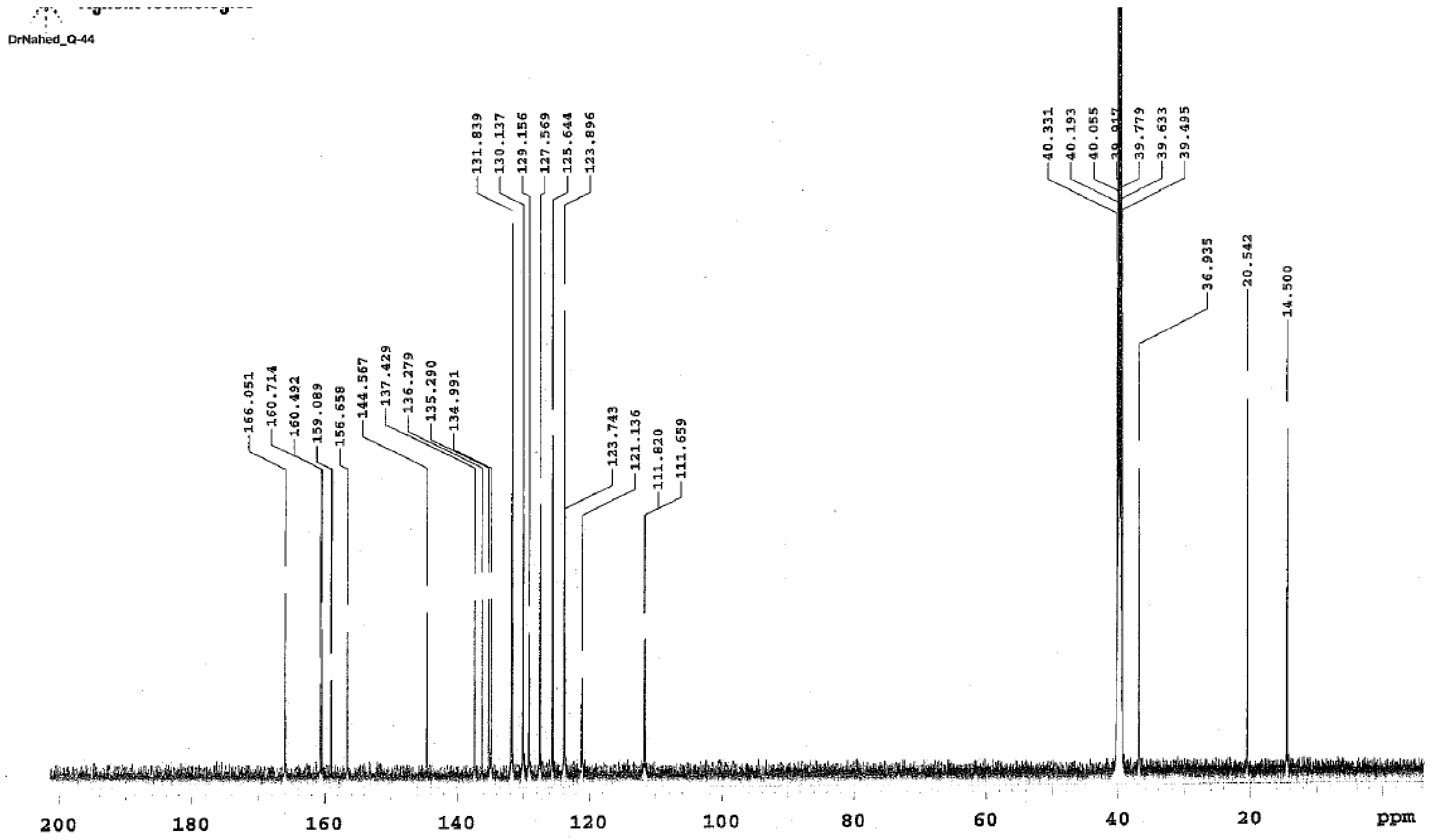
DrNahed_Q-44
166.051
160.714
160.492
159.089
156.658
144.567
137.429
136.279
135.290
134.991
131.839
130.137
129.156
127.569
125.644
123.896
123.743
121.136
111.820
111.659
40.331
40.193
40.055
39.779
39.633
39.495
36.935
20.542
14.500
200
180
160
140
120
100
80
60
40
20
ppm

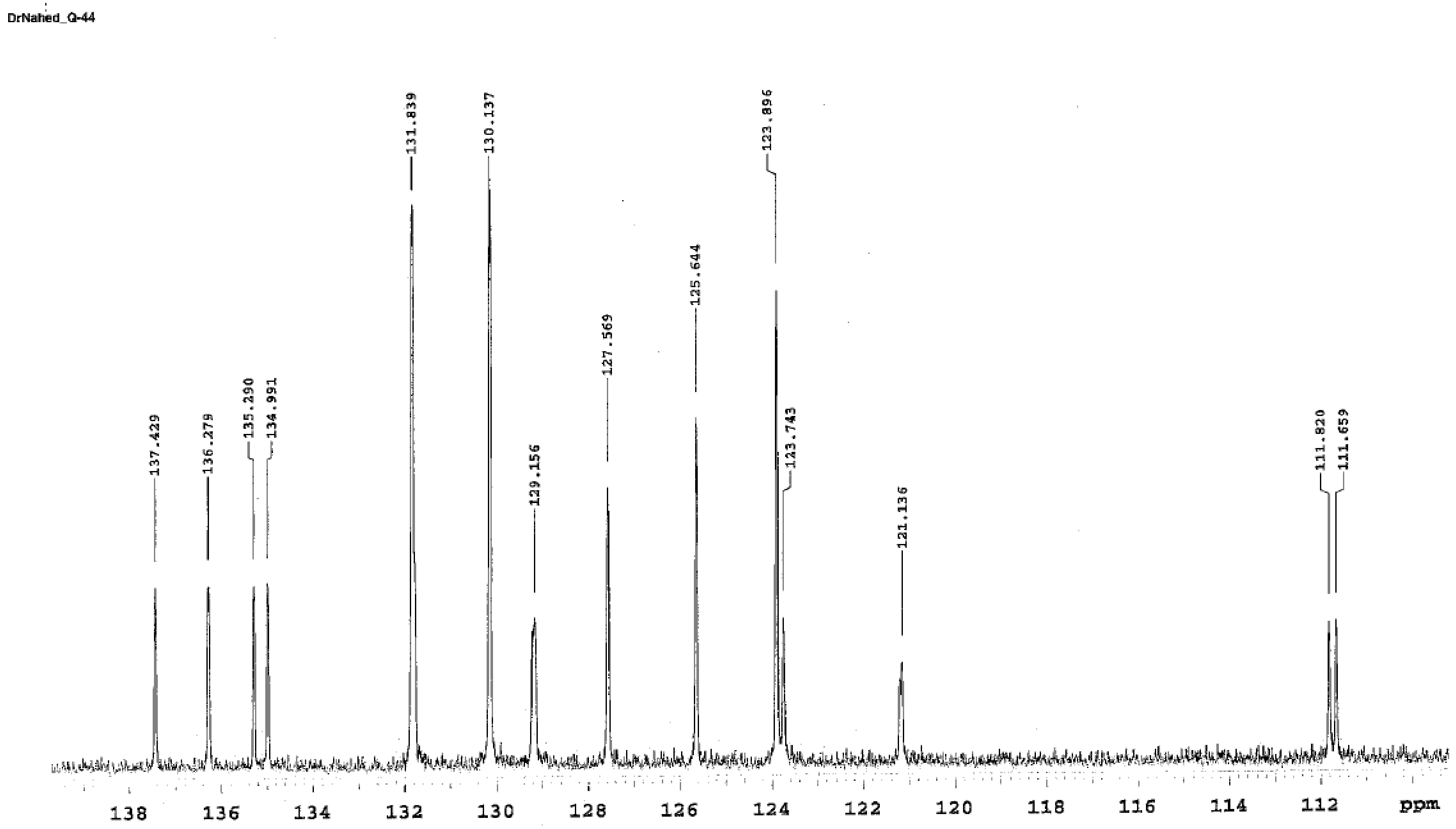

Expanded ^{13}C NMR spectrum ^{13}C NMR (150 MHz; DMSO-d_6) of compound **3a** showing ^{13}C–^{19}F couplings.

DrNahed_Q-44

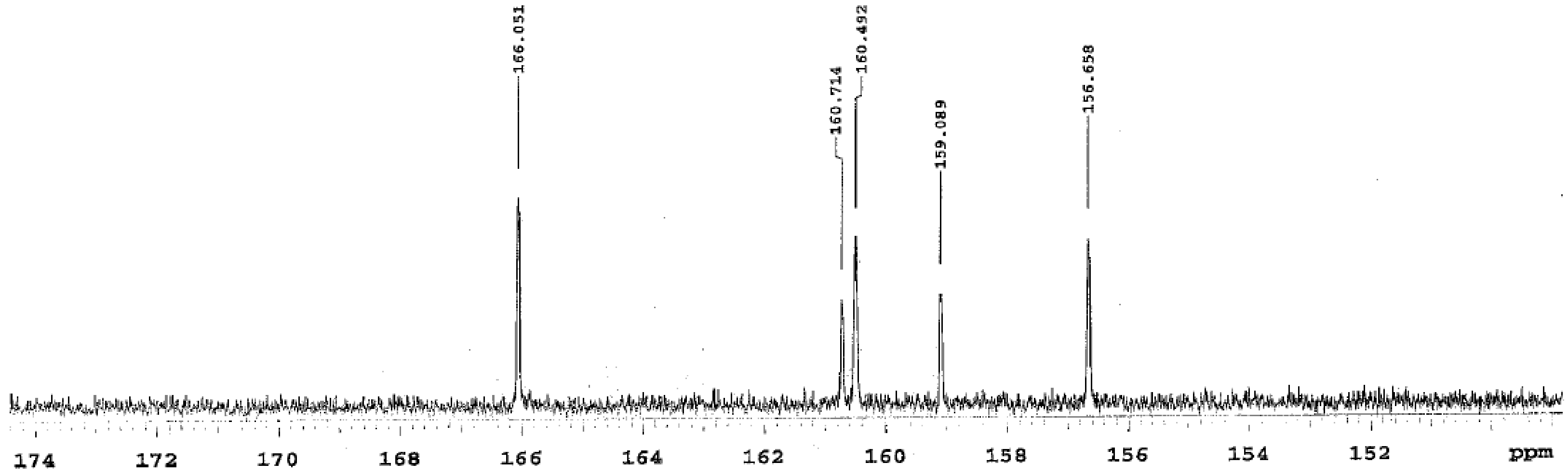

Expanded ^{13}C NMR spectrum ^{13}C NMR (150 MHz; DMSO-d_6) of compound **3a** showing ^{13}C–^{19}F couplings.

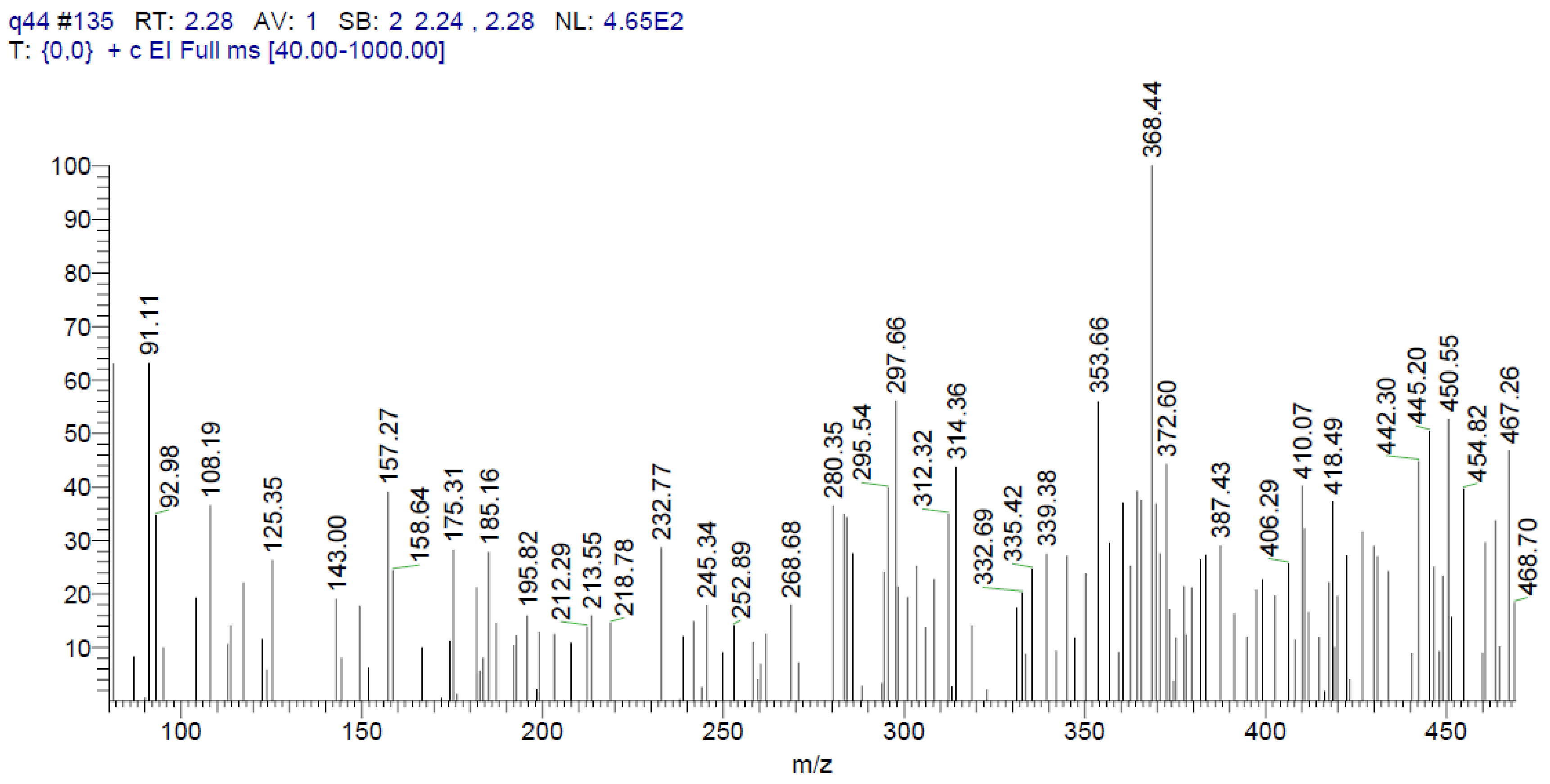
q44 #135 RT: 2.28 AV: 1 SB: 2 2.24 , 2.28 NL: 4.65E2
T: {0,0} + c EI Full ms [40.00-1000.00]
m/z

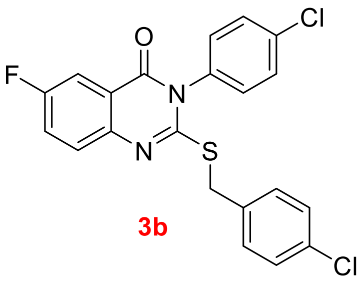
O
F
N
N
S
Cl
Cl

3b

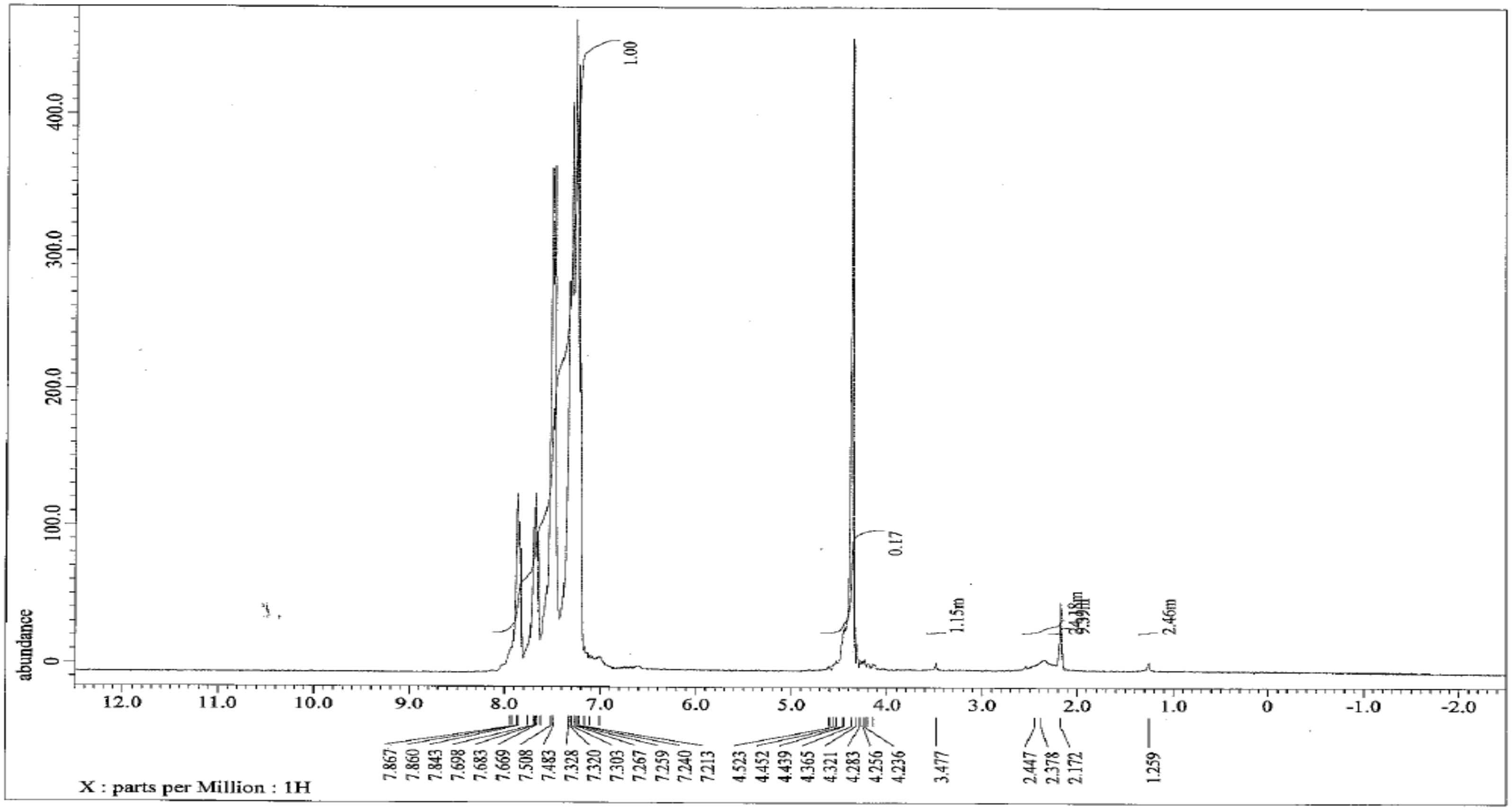
abundance
400.0
300.0
200.0
100.0
0
12.0
11.0
10.0
9.0
8.0
7.0
6.0
5.0
4.0
3.0
2.0
1.0
0
-1.0
-2.0
1.00
0.17
1.15m
2.46m
7.867
7.860
7.843
7.698
7.683
7.669
7.508
7.483
7.328
7.320
7.303
7.267
7.259
7.240
7.213
4.523
4.452
4.439
4.365
4.321
4.283
4.256
4.236
3.477
2.447
2.378
2.172
1.259
X : parts per Million : 1H

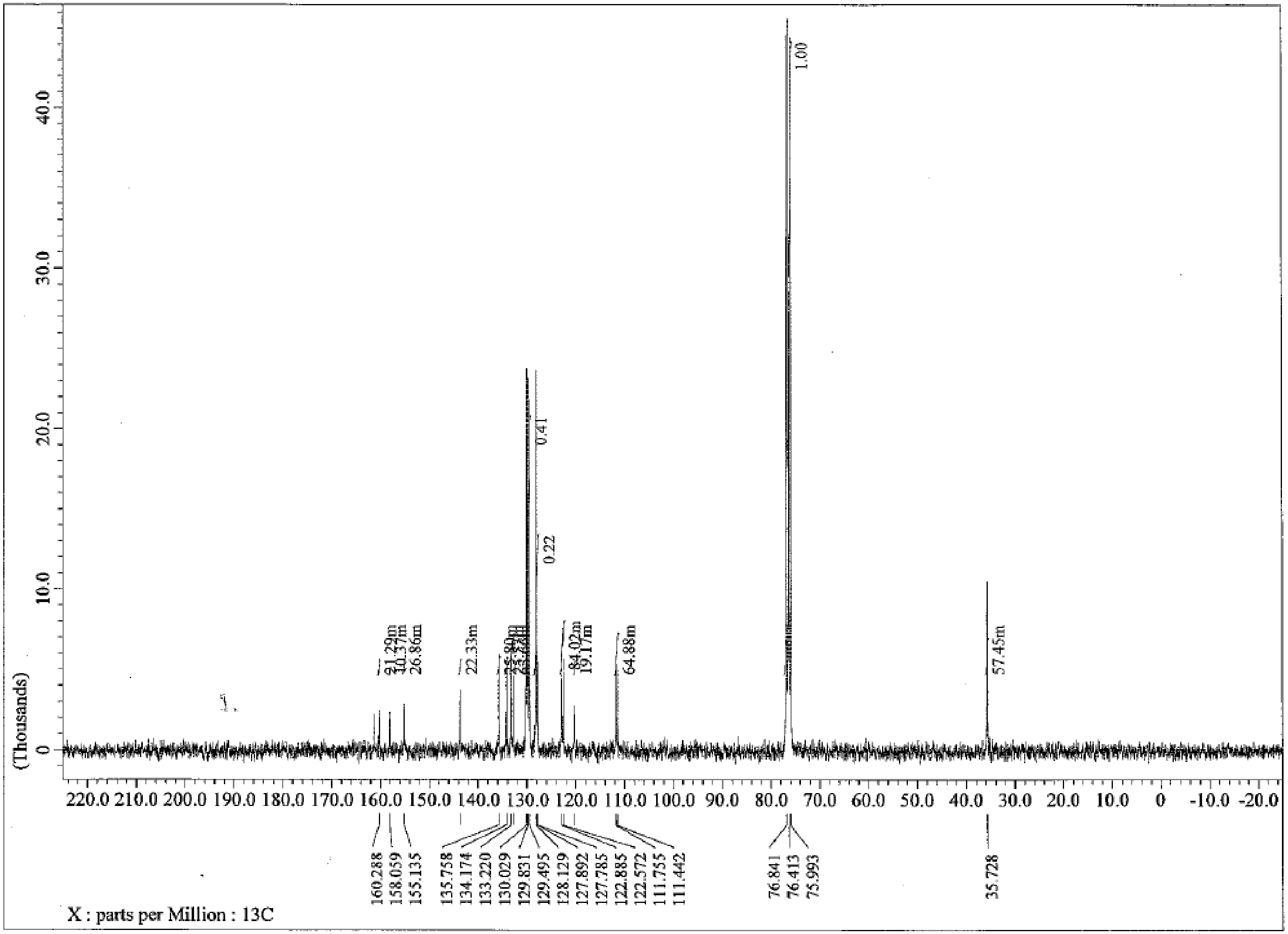
(Thousands)
X : parts per Million : 13C
160.288
158.059
155.135
135.758
134.174
133.220
130.029
129.831
129.495
128.129
127.892
127.785
122.885
122.572
111.755
111.442
76.841
76.413
75.993
35.728

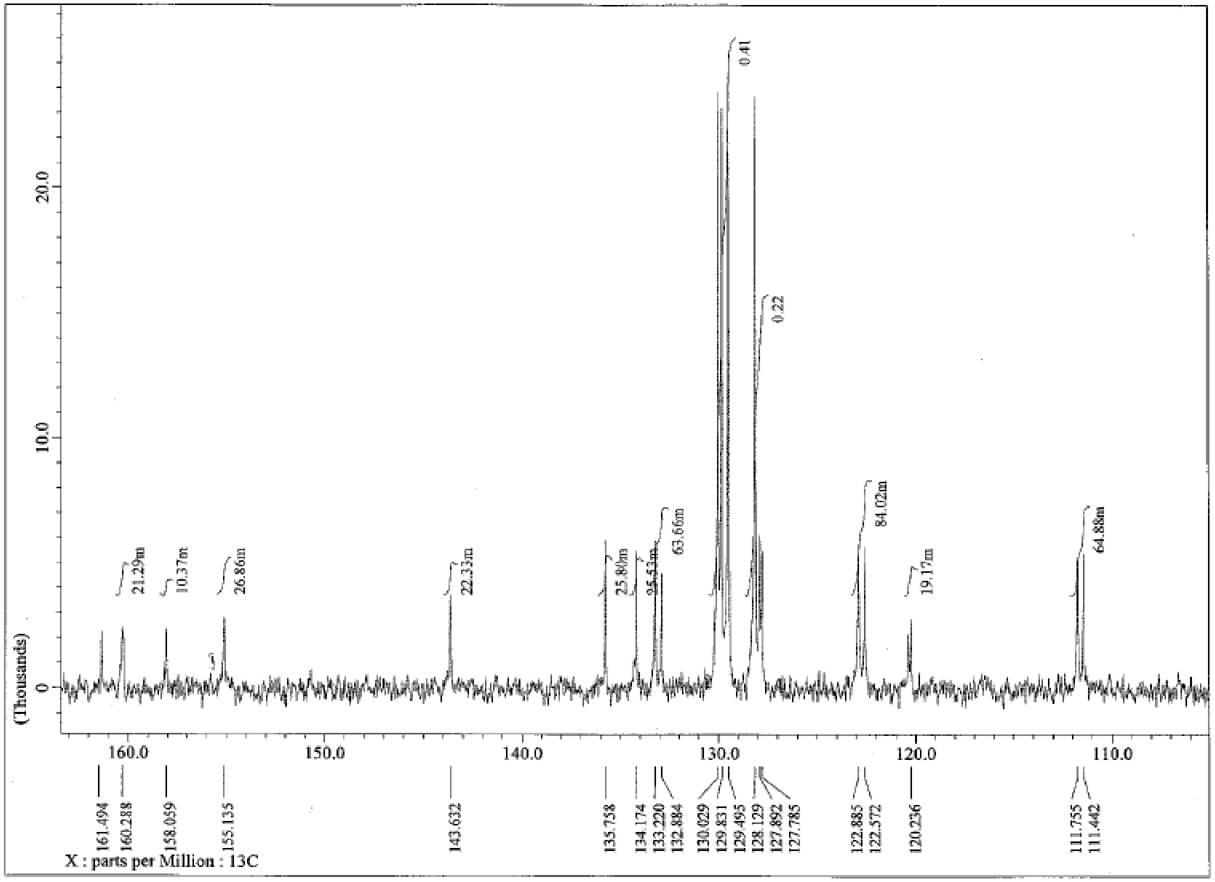

Expanded ^{13}C NMR spectrum (75 MHz; $CDCl_3$) of compound **3b** showing ^{13}C–^{19}F couplings.

q35 #23-24 RT: 0.40-0.42 AV: 2 SB: 26 1.21-1.34 , 0.87-1.14 NL: 3.93E2
T: {0,0} + c EI Full ms [40.00-1000.00]

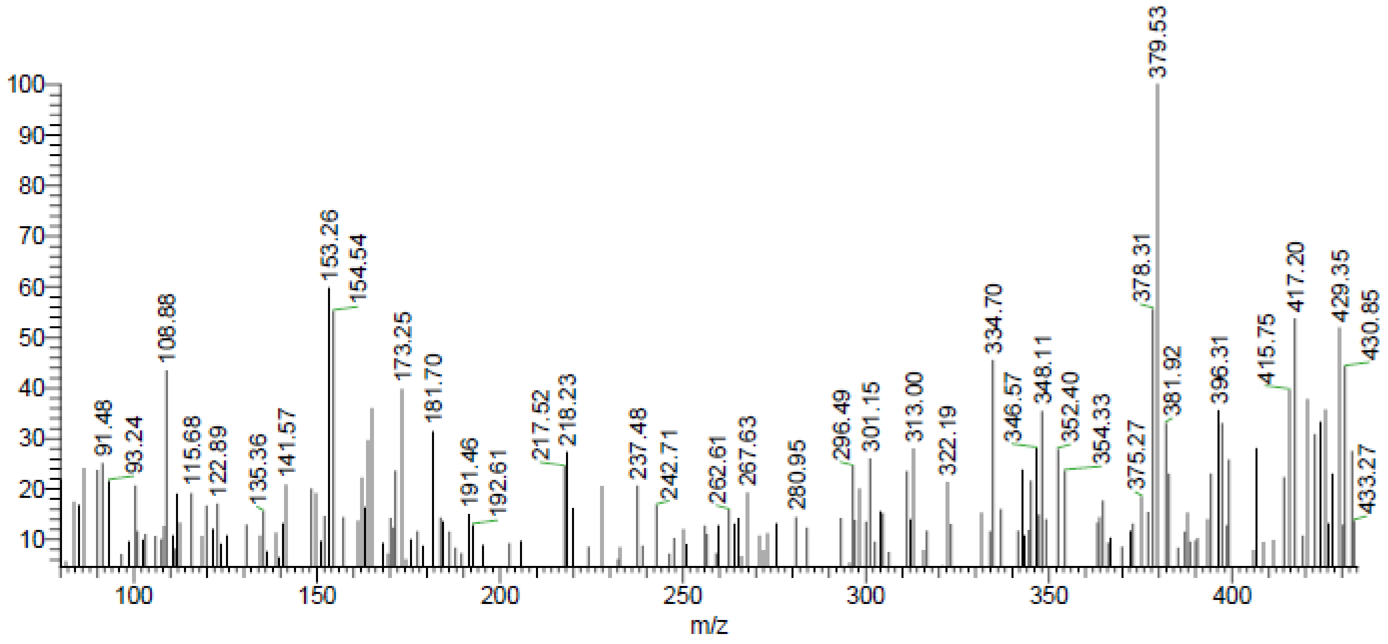

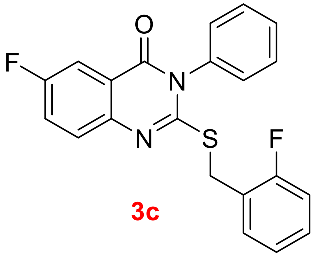
O
F
N
N
S
F

3c

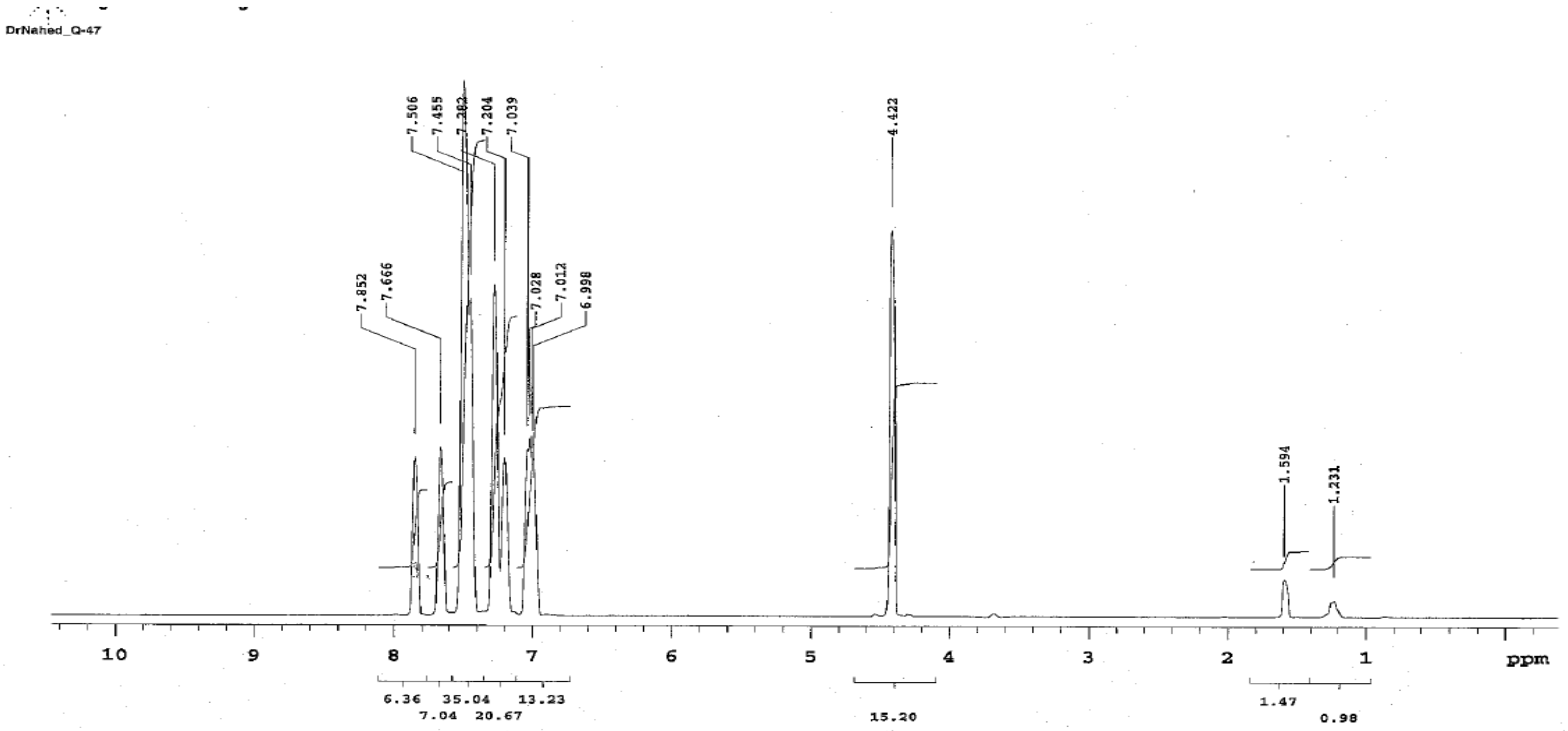
DrNahed_Q-47
7.852
7.666
7.506
7.455
7.282
7.204
7.039
7.028
7.012
6.998
4.422
1.594
1.231
10
9
8
7
6
5
4
3
2
1
ppm
6.36
7.04
35.04
20.67
13.23
15.20
1.47
0.98

DrNahed_Q-47

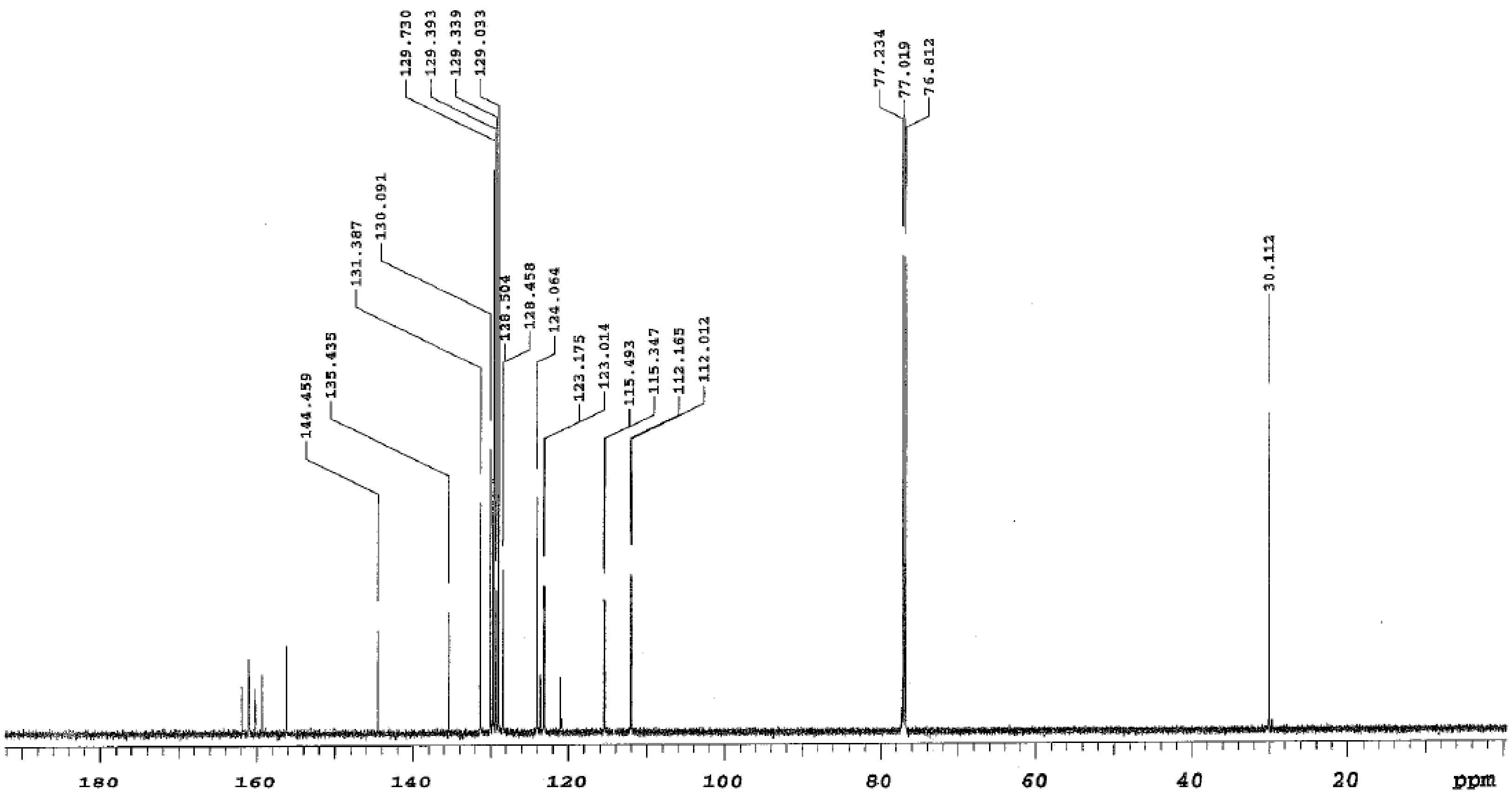

129.730
129.393
129.339
129.033
77.234
77.019
76.812
130.091
131.387
128.504
128.458
124.064
30.112
135.435
144.459
123.175
123.014
115.493
115.347
112.165
112.012
180
160
140
120
100
80
60
40
20
ppm

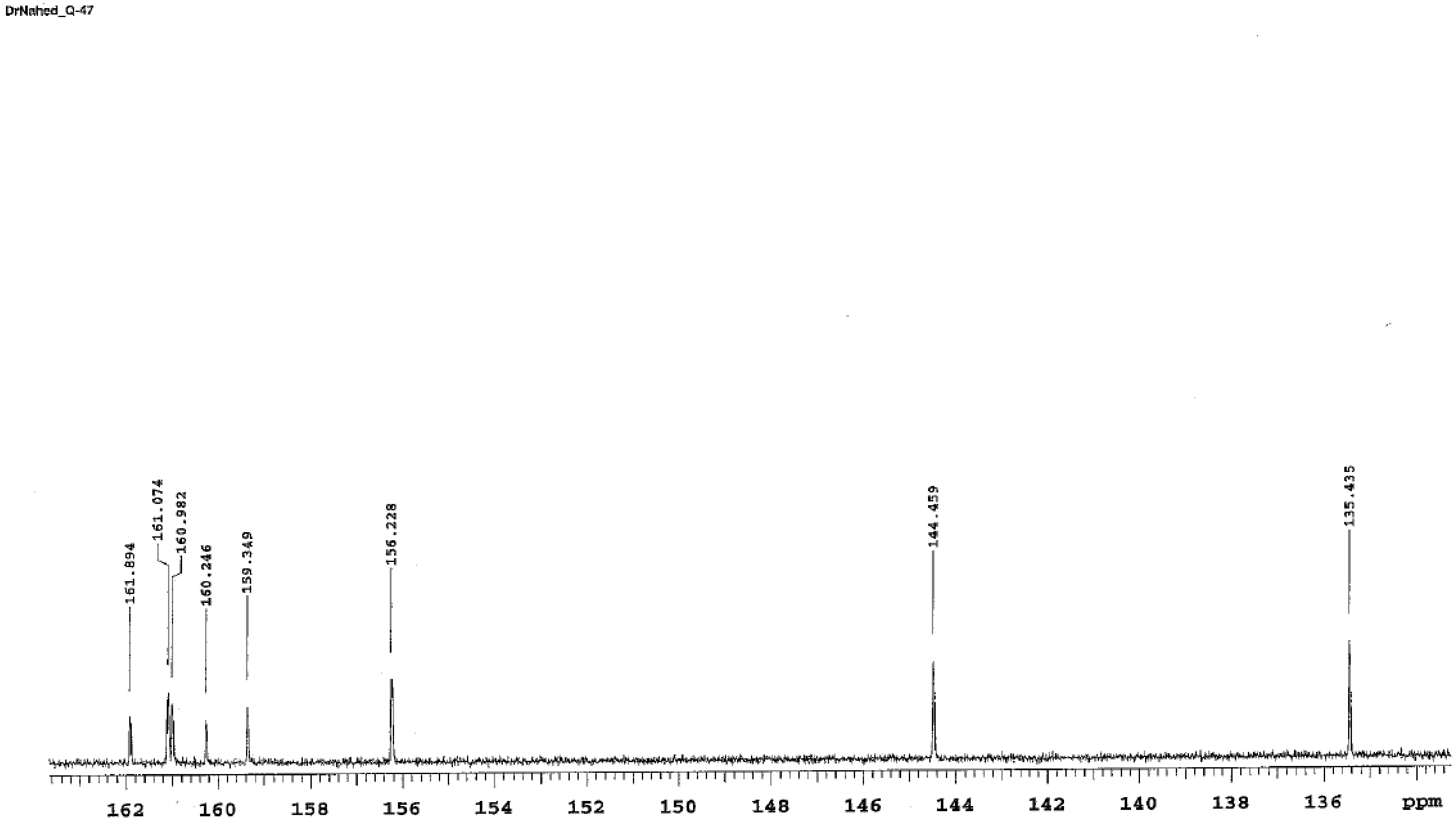

Expanded ^{13}C NMR (150 MHz; $CDCl_3$) of compound **3c** showing ^{13}C–^{19}F couplings.

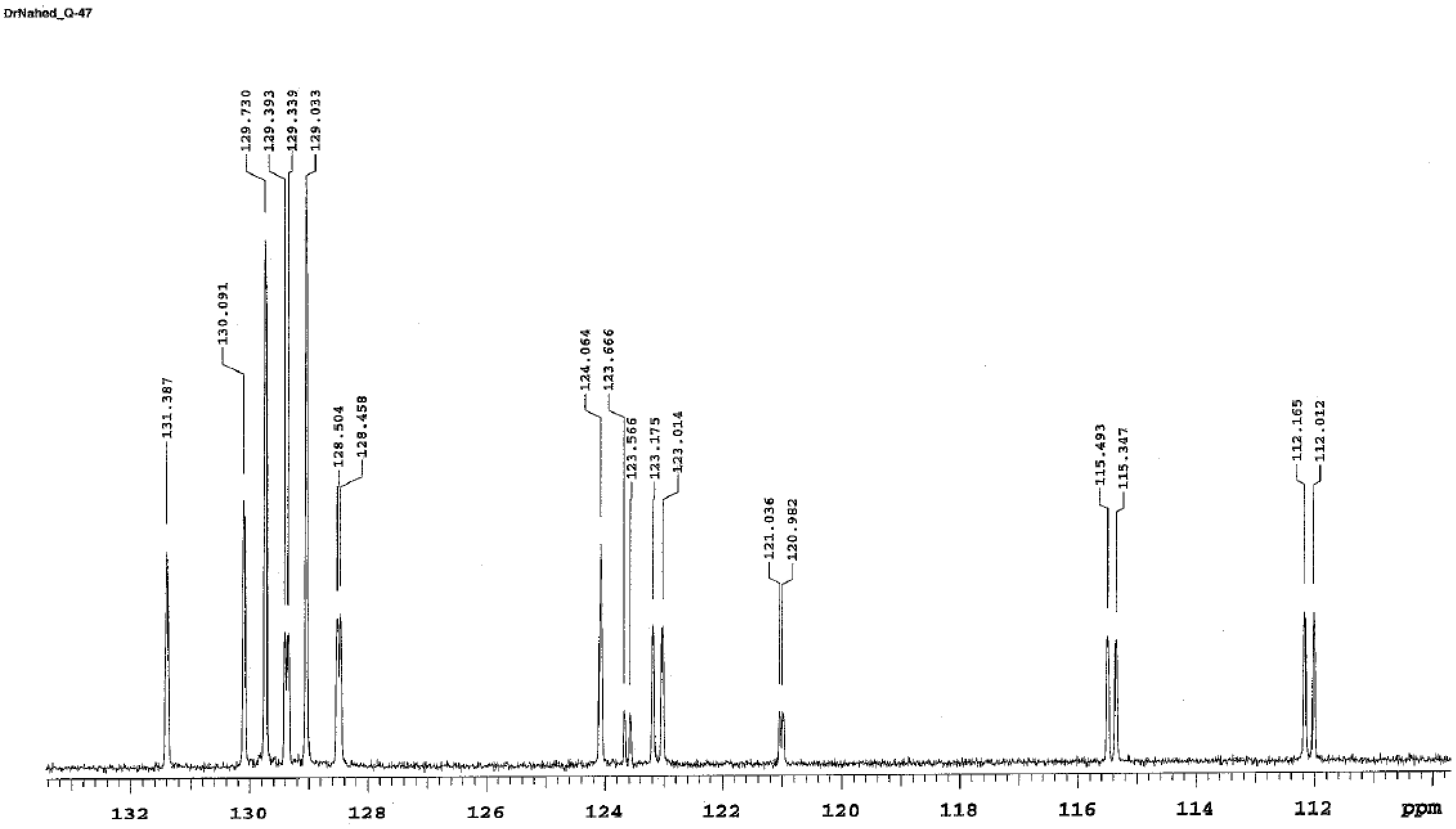

Expanded ^{13}C NMR (150 MHz; $CDCl_3$) of compound **3c** showing ^{13}C–^{19}F couplings.

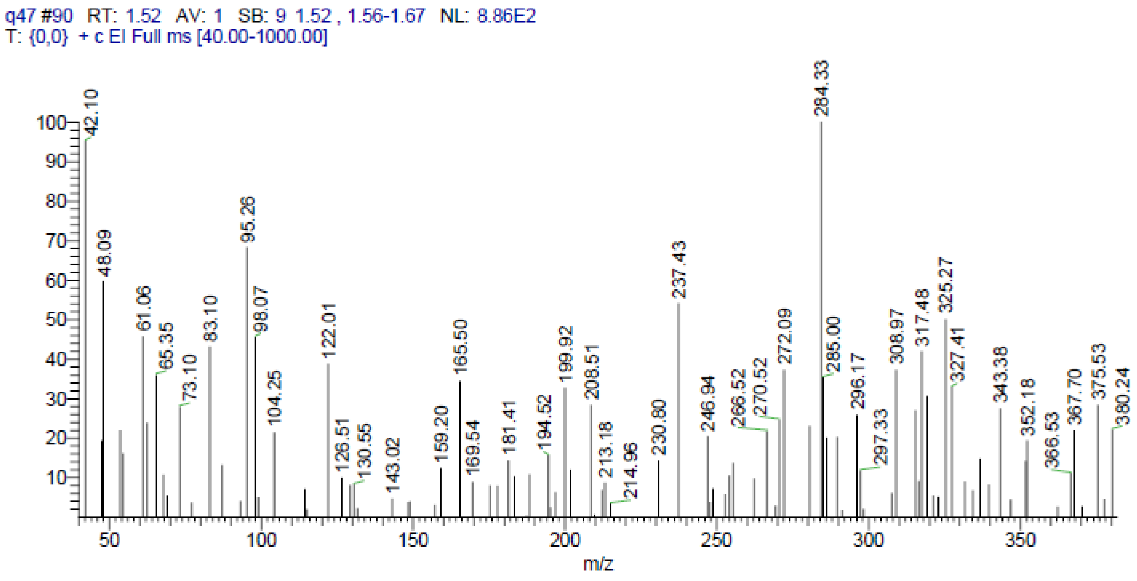
q47 #90 RT: 1.52 AV: 1 SB: 9 1.52 , 1.56-1.67 NL: 8.86E2
T: {0,0} + c EI Full ms [40.00-1000.00]
42.10
48.09
61.06
65.35
73.10
83.10
95.26
98.07
104.25
122.01
126.51
130.55
143.02
159.20
165.50
169.54
181.41
194.52
199.92
208.51
213.18
214.96
230.80
237.43
246.94
266.52
270.52
272.09
284.33
285.00
296.17
297.33
308.97
317.48
325.27
327.41
343.38
352.18
366.53
367.70
375.53
380.24
m/z

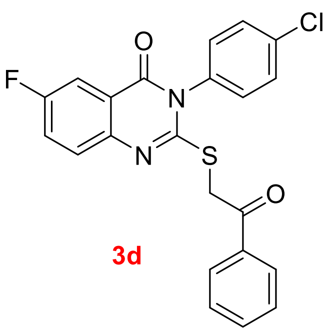
F
O
N
Cl
N
S
O

3d

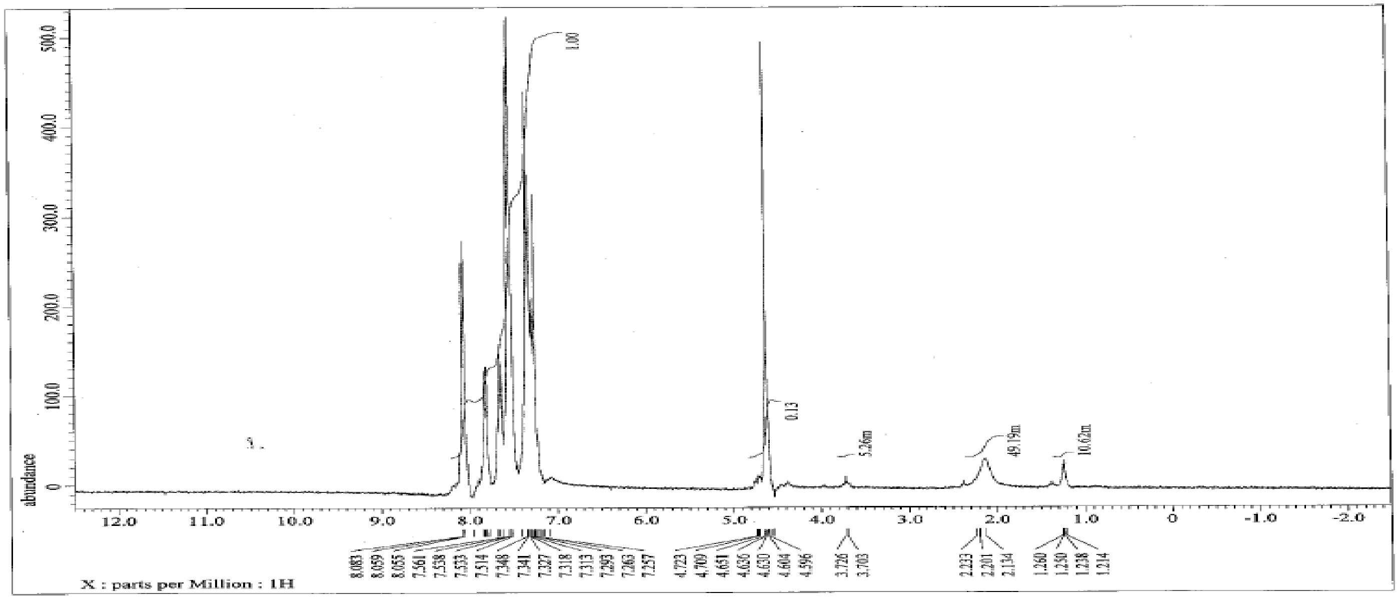
abundance
500.0
400.0
300.0
200.0
100.0
0
1.00
0.13
5.26m
49.19m
10.62m
12.0
11.0
10.0
9.0
8.0
7.0
6.0
5.0
4.0
3.0
2.0
1.0
0
-1.0
-2.0
8.083
8.059
8.055
7.561
7.538
7.533
7.514
7.348
7.341
7.327
7.318
7.313
7.293
7.263
7.257
4.723
4.709
4.651
4.636
4.630
4.604
4.596
3.726
3.703
2.223
2.201
2.134
1.260
1.250
1.238
1.214
X : parts per Million : 1H

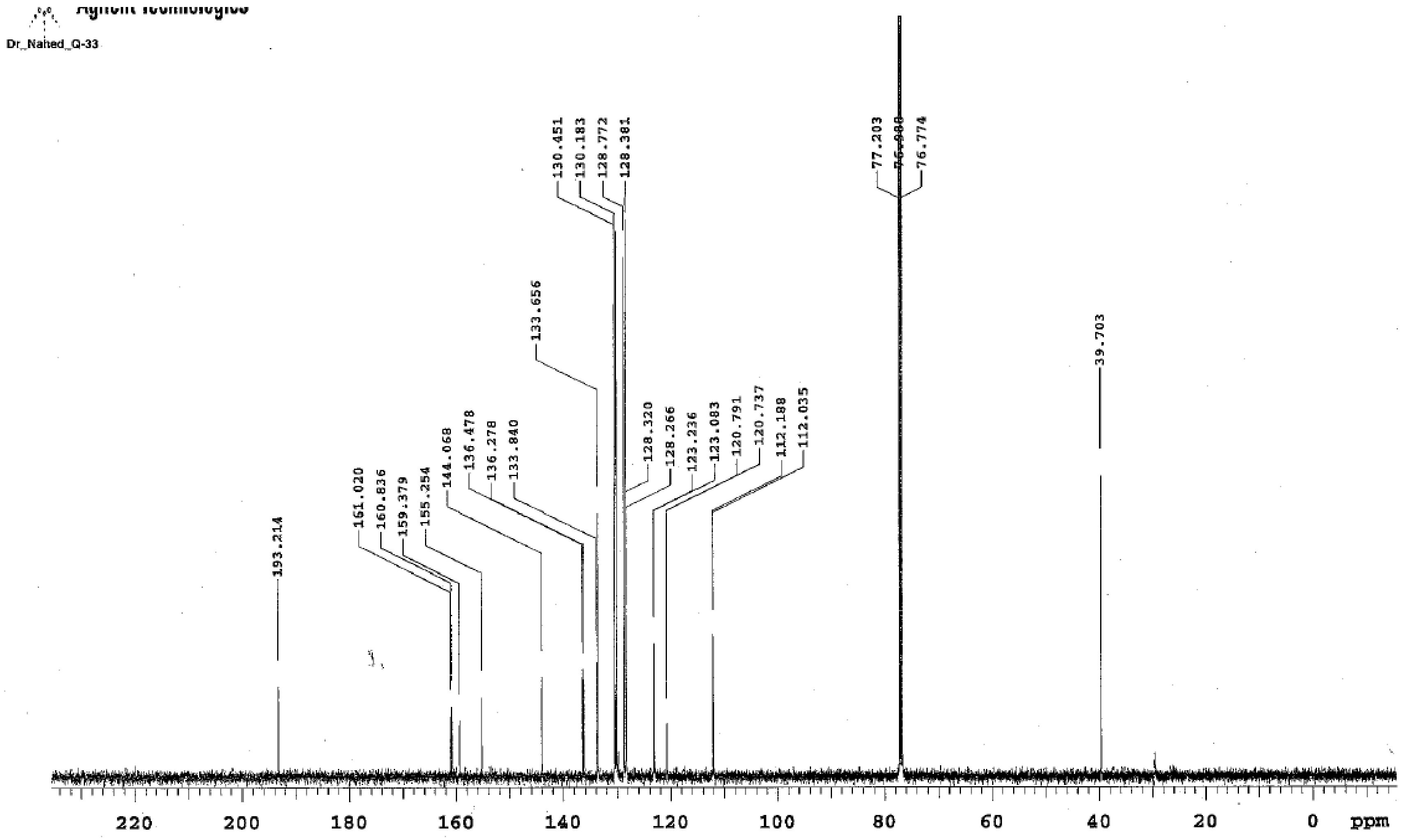
Dr_Nahed_Q-33
193.214
161.020
160.836
159.379
155.254
144.068
136.478
136.278
133.840
133.656
130.451
130.183
128.772
128.381
128.320
128.266
123.236
123.083
120.791
120.737
112.188
112.035
77.203
76.774
39.703
220
200
180
160
140
120
100
80
60
40
20
0
ppm

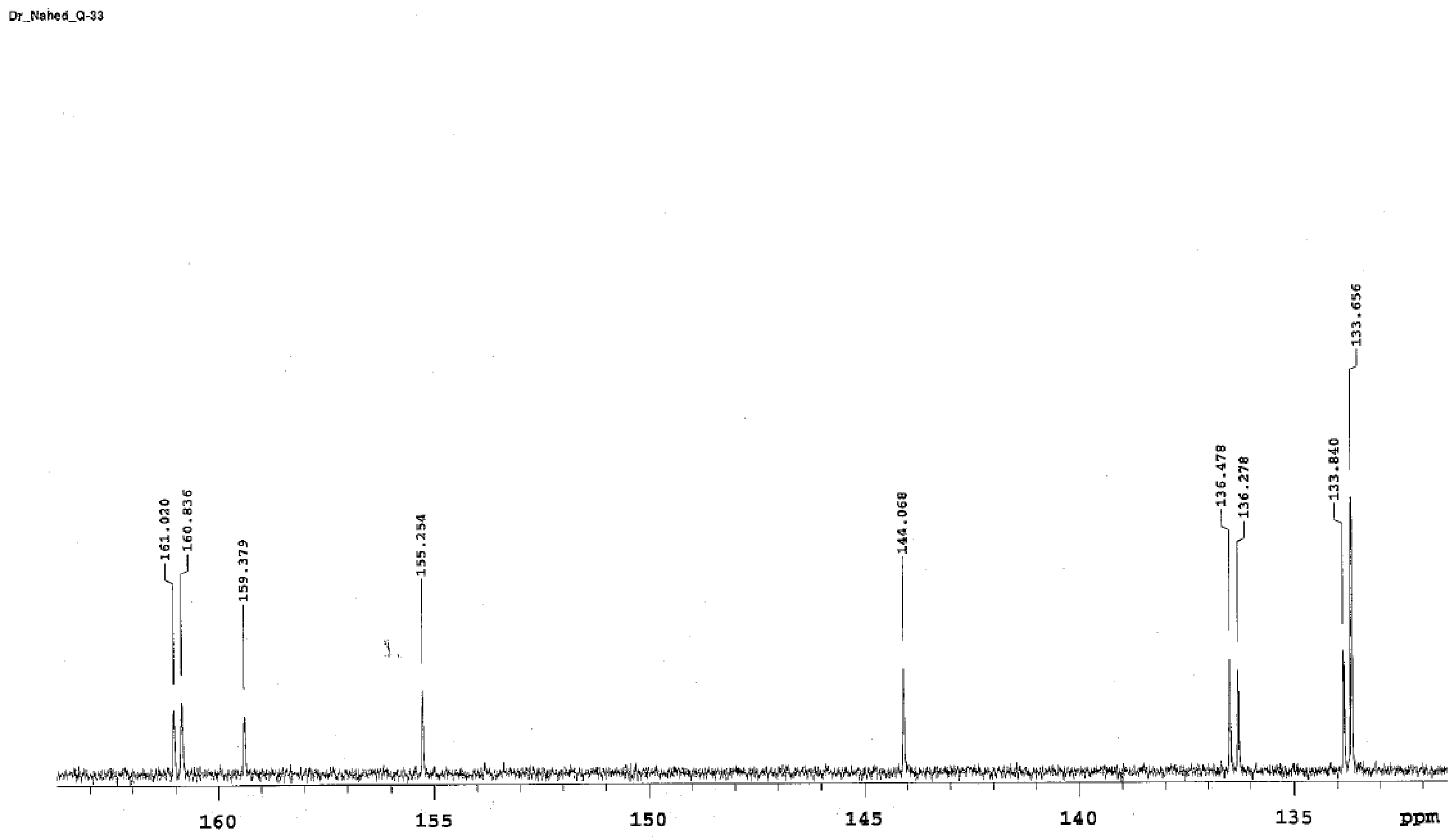

Expanded ^{13}C NMR spectrum (150 MHz; $CDCl_3$) of compound **3d** showing ^{13}C–^{19}F couplings.

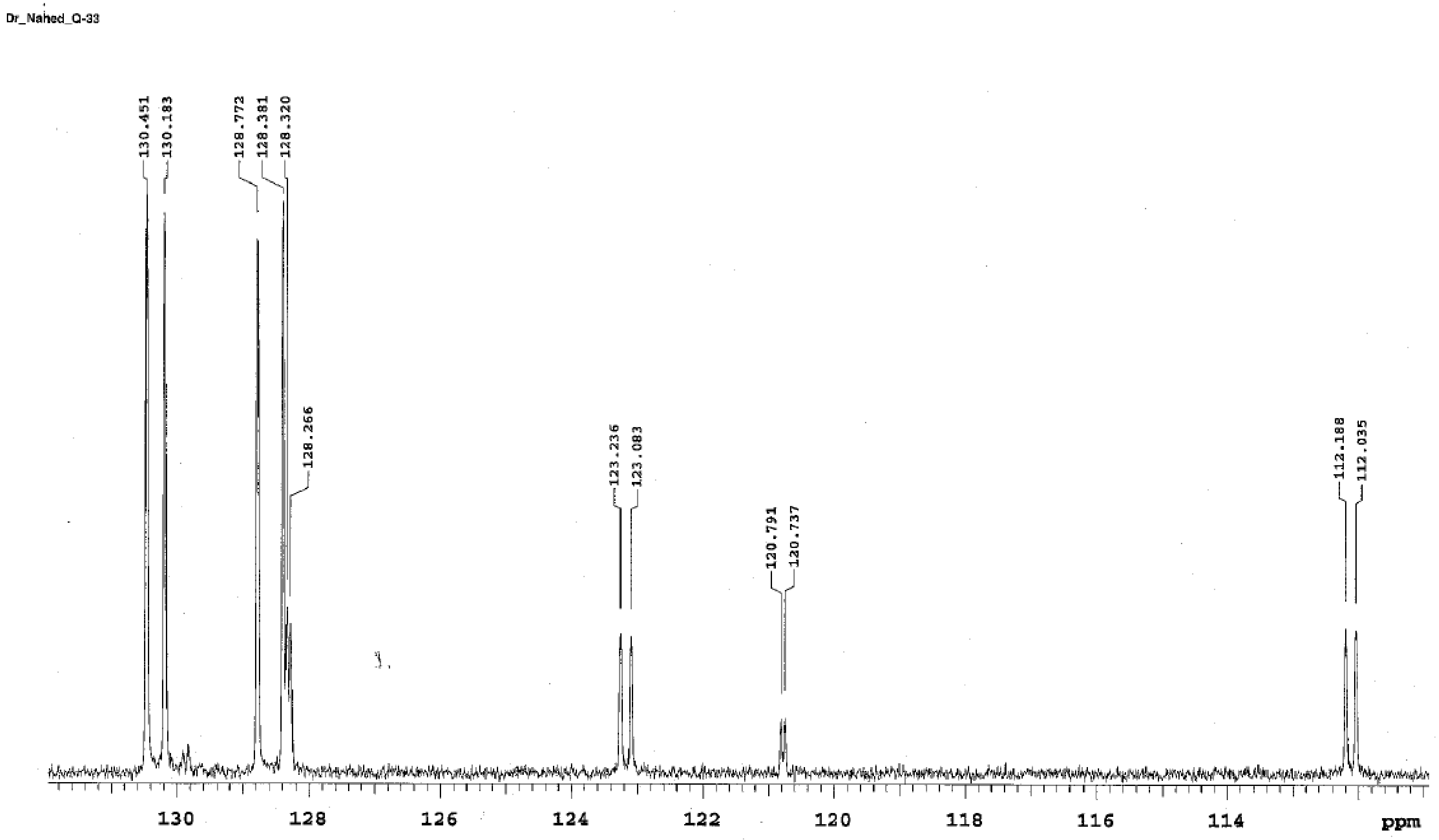

Expanded ^{13}C NMR spectrum (150 MHz; $CDCl_3$) of compound **3d** showing ^{13}C–^{19}F couplings.

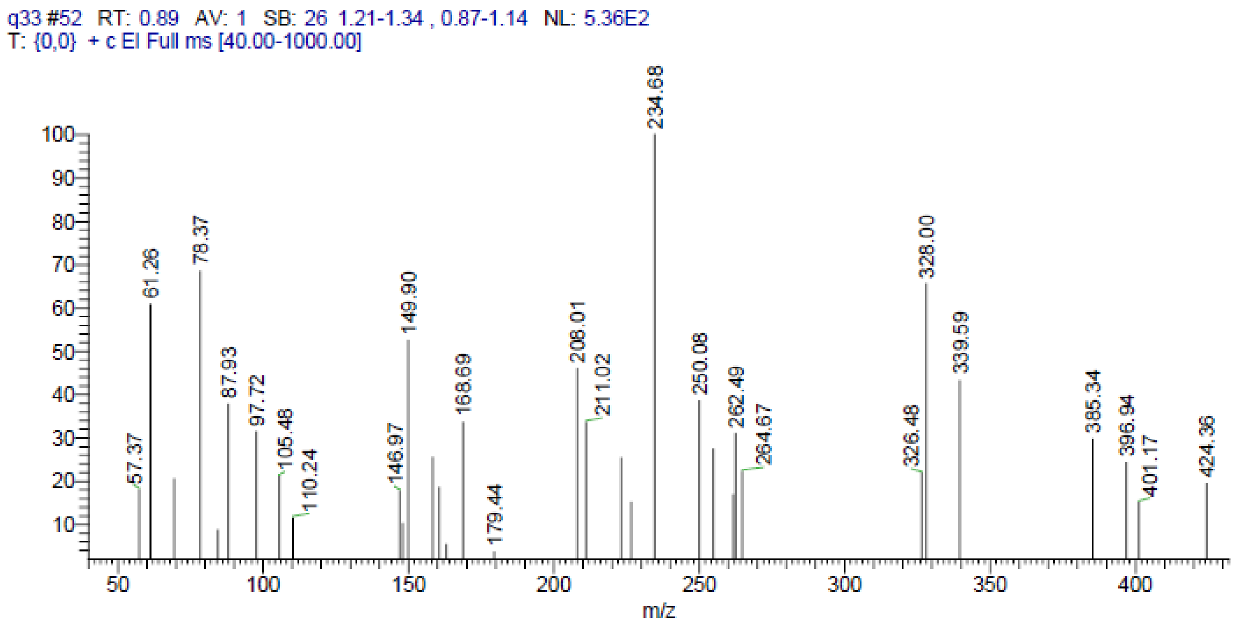
q33 #52 RT: 0.89 AV: 1 SB: 26 1.21-1.34 , 0.87-1.14 NL: 5.36E2
T: {0,0} + c EI Full ms [40.00-1000.00]
234.68
328.00
78.37
61.26
149.90
208.01
339.59
87.93
97.72
168.69
211.02
250.08
262.49
264.67
385.34
396.94
105.48
110.24
146.97
326.48
401.17
424.36
57.37
179.44
m/z

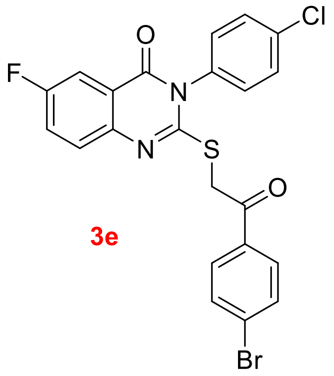

3e

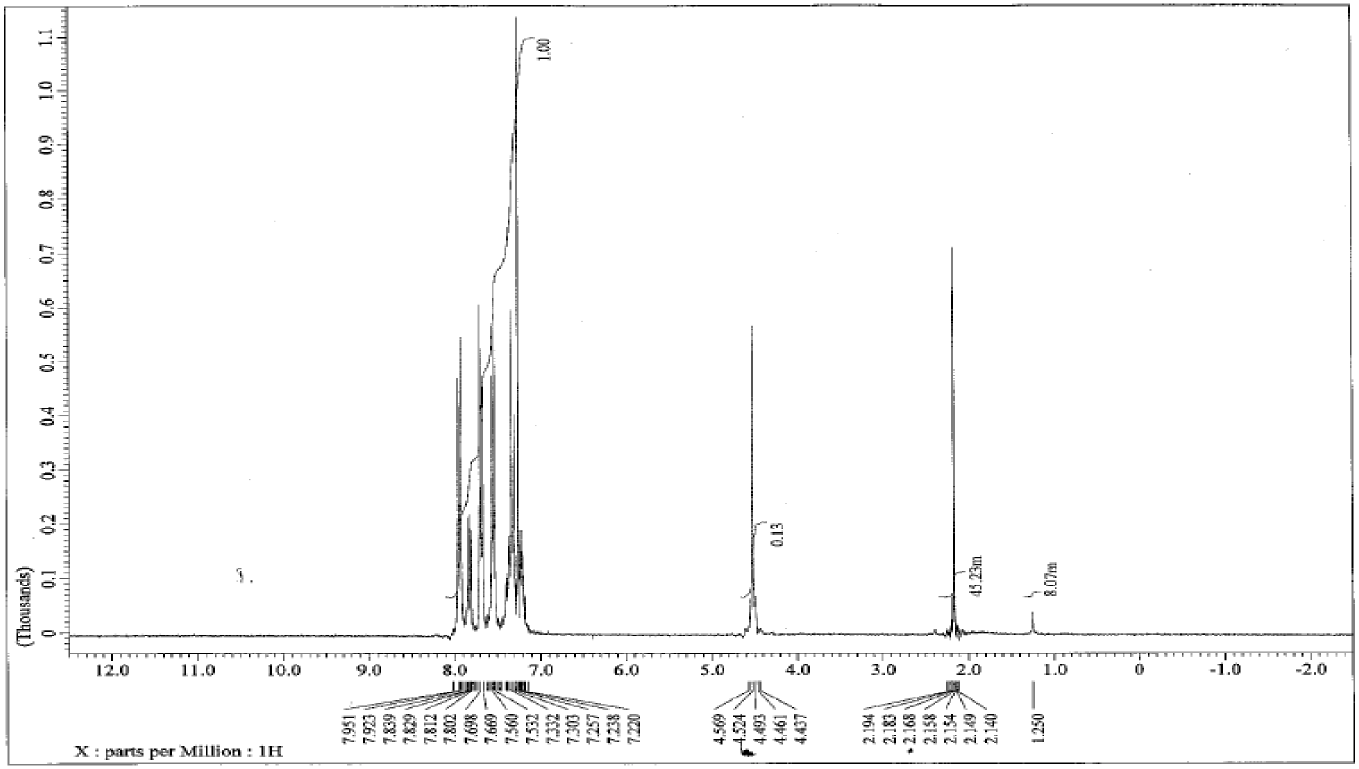
(Thousands)
1.00
0.13
45.23m
8.07m
12.0
11.0
10.0
9.0
8.0
7.0
6.0
5.0
4.0
3.0
2.0
1.0
0
-1.0
-2.0
7.951 7.923 7.839 7.829 7.812 7.802 7.698 7.669 7.560 7.532 7.332 7.303 7.257 7.238 7.220
4.569 4.524 4.493 4.461 4.437
2.194 2.183 2.168 2.158 2.154 2.149 2.140
1.250
X : parts per Million : 1H

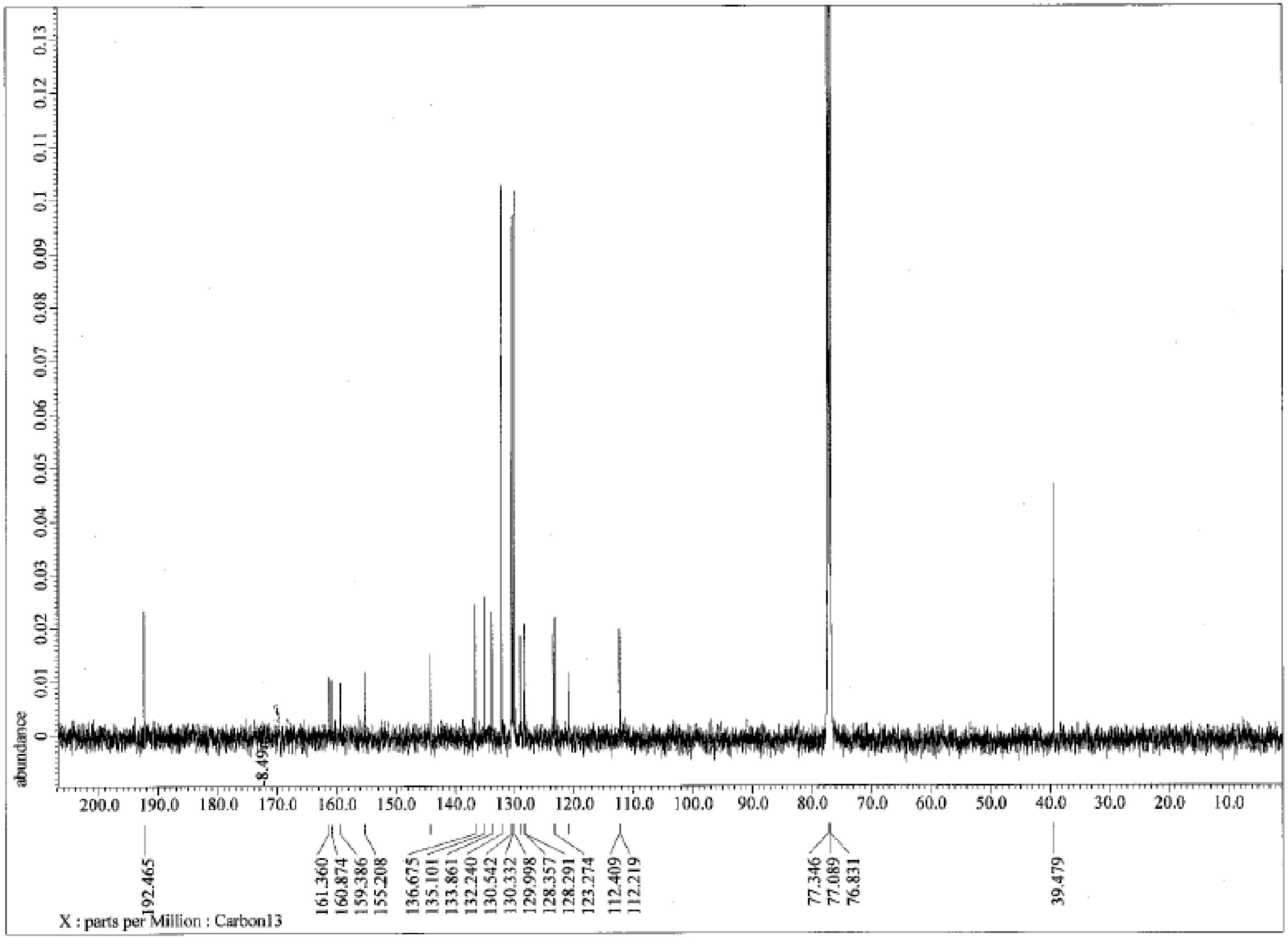
abundance
X : parts per Million : Carbon13
192.465
161.360
160.874
159.386
155.208
136.675
135.101
133.861
132.240
130.542
130.332
129.998
128.357
128.291
123.274
112.409
112.219
77.346
77.089
76.831
39.479

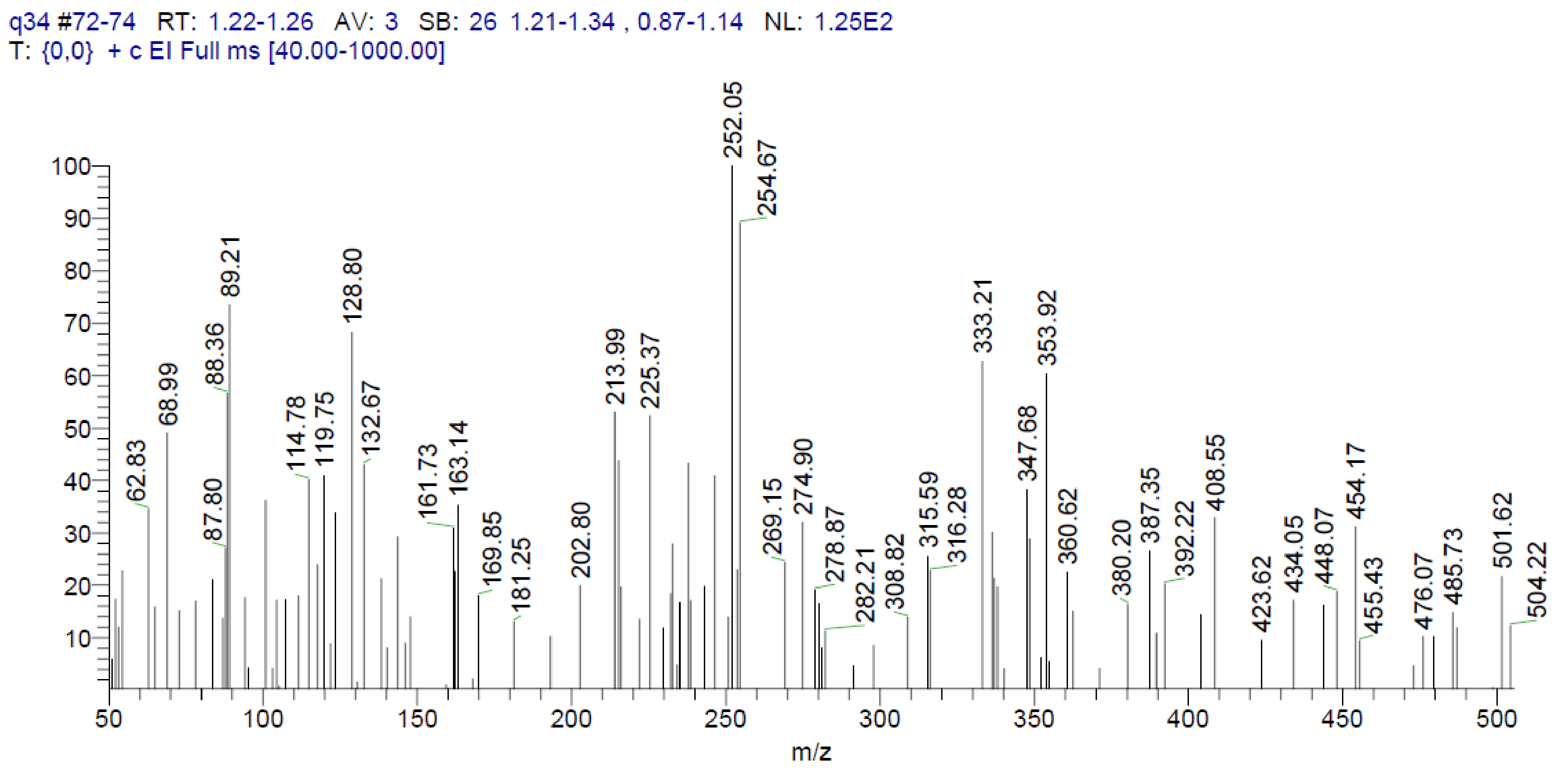
q34 #72-74 RT: 1.22-1.26 AV: 3 SB: 26 1.21-1.34 , 0.87-1.14 NL: 1.25E2
T: {0,0} + c EI Full ms [40.00-1000.00]
252.05
254.67
89.21
128.80
88.36
68.99
333.21
353.92
213.99
225.37
114.78
119.75
132.67
62.83
87.80
161.73
163.14
169.85
181.25
202.80
269.15
274.90
278.87
282.21
308.82
315.59
316.28
347.68
360.62
380.20
387.35
392.22
408.55
423.62
434.05
448.07
454.17
455.43
476.07
485.73
501.62
504.22
m/z

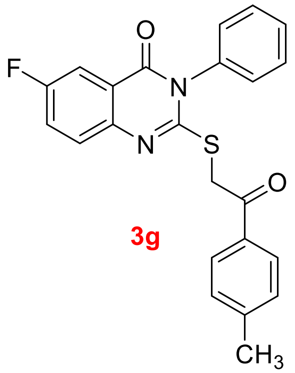

3g

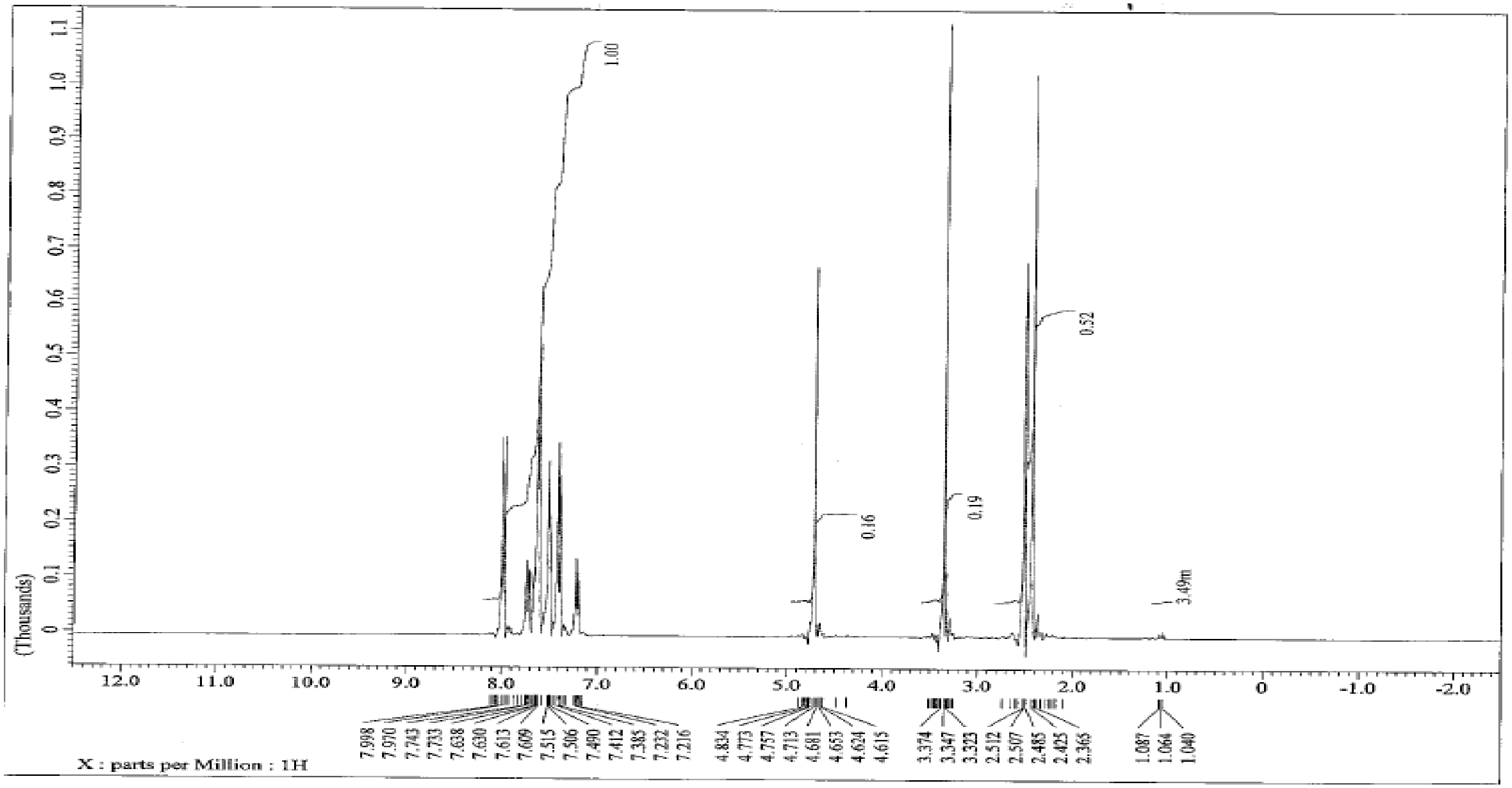
X : parts per Million : 1H
(Thousands)
1.00
0.16
0.19
0.52
3.49m
7.998
7.970
7.743
7.733
7.638
7.630
7.613
7.609
7.515
7.506
7.490
7.412
7.385
7.232
7.216
4.834
4.773
4.757
4.713
4.681
4.653
4.624
4.615
3.374
3.347
3.323
2.512
2.507
2.485
2.425
2.365
1.087
1.064
1.040

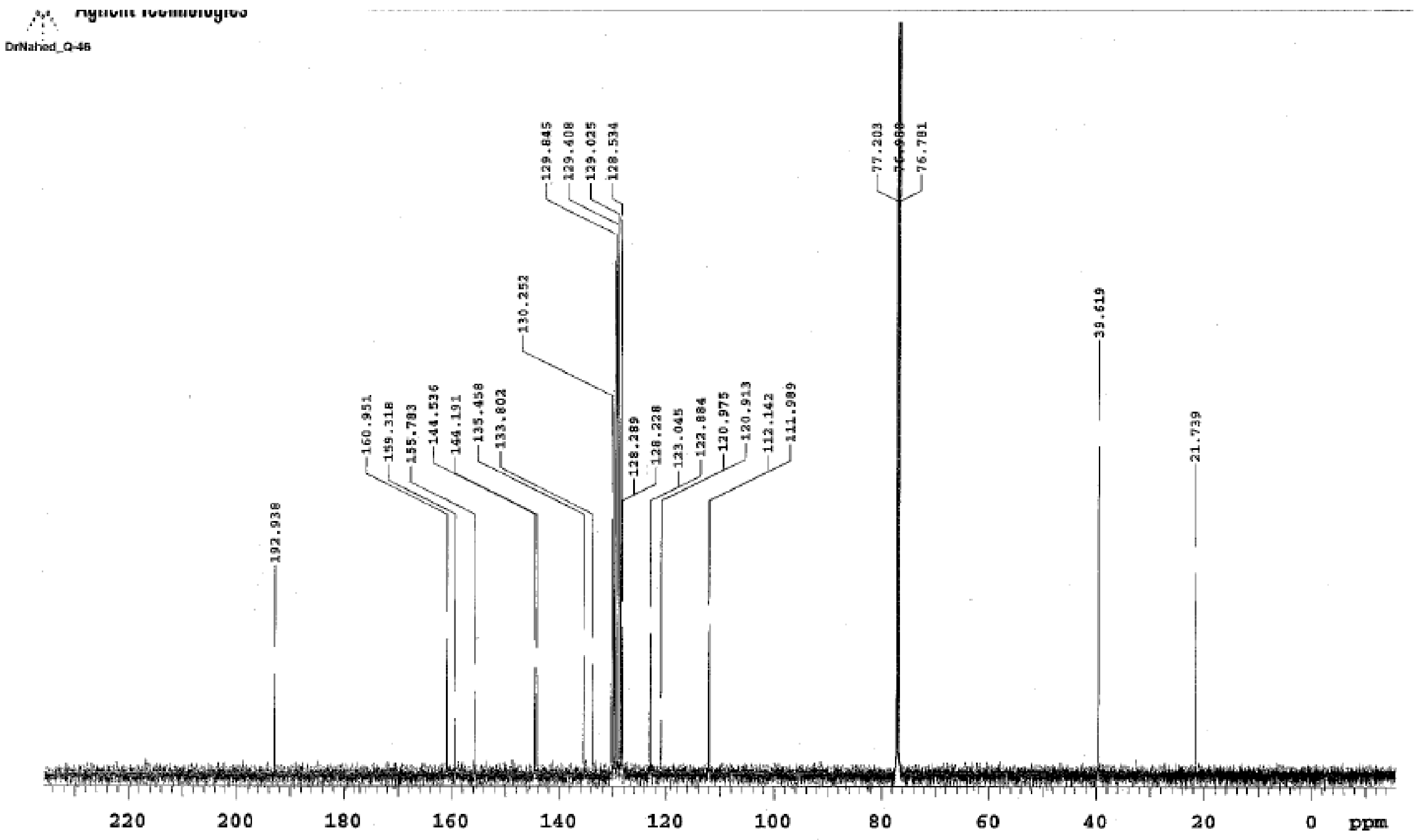

Agilent Technologies
DrNahed_Q-46
192.938
160.951
159.318
155.783
144.536
144.191
135.458
133.802
130.252
129.845
129.408
129.025
128.534
128.289
128.228
123.045
122.884
120.975
120.913
112.142
111.989
77.203
76.781
39.619
21.739
220
200
180
160
140
120
100
80
60
40
20
0
ppm

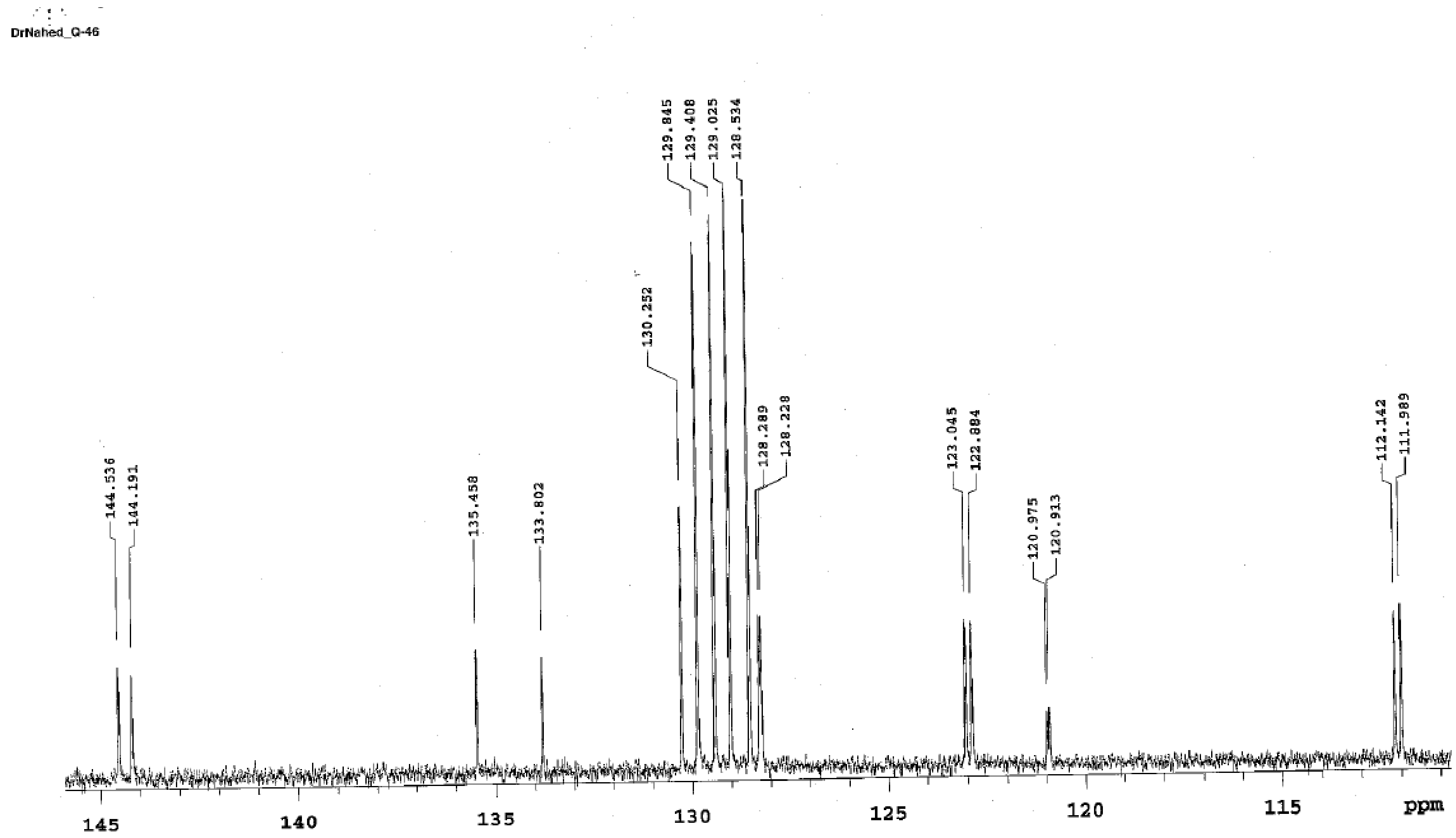

Expanded ^{13}C NMR (150 MHz; $CDCl_3$) of compound **3g** showing ^{13}C–^{19}F couplings.

q46 #101-103 RT: 1.71-1.74 AV: 3 SB: 21 1.51 , 1.29-1.61 NL: 2.17E2
T: {0,0} + c EI Full ms [40.00-1000.00]

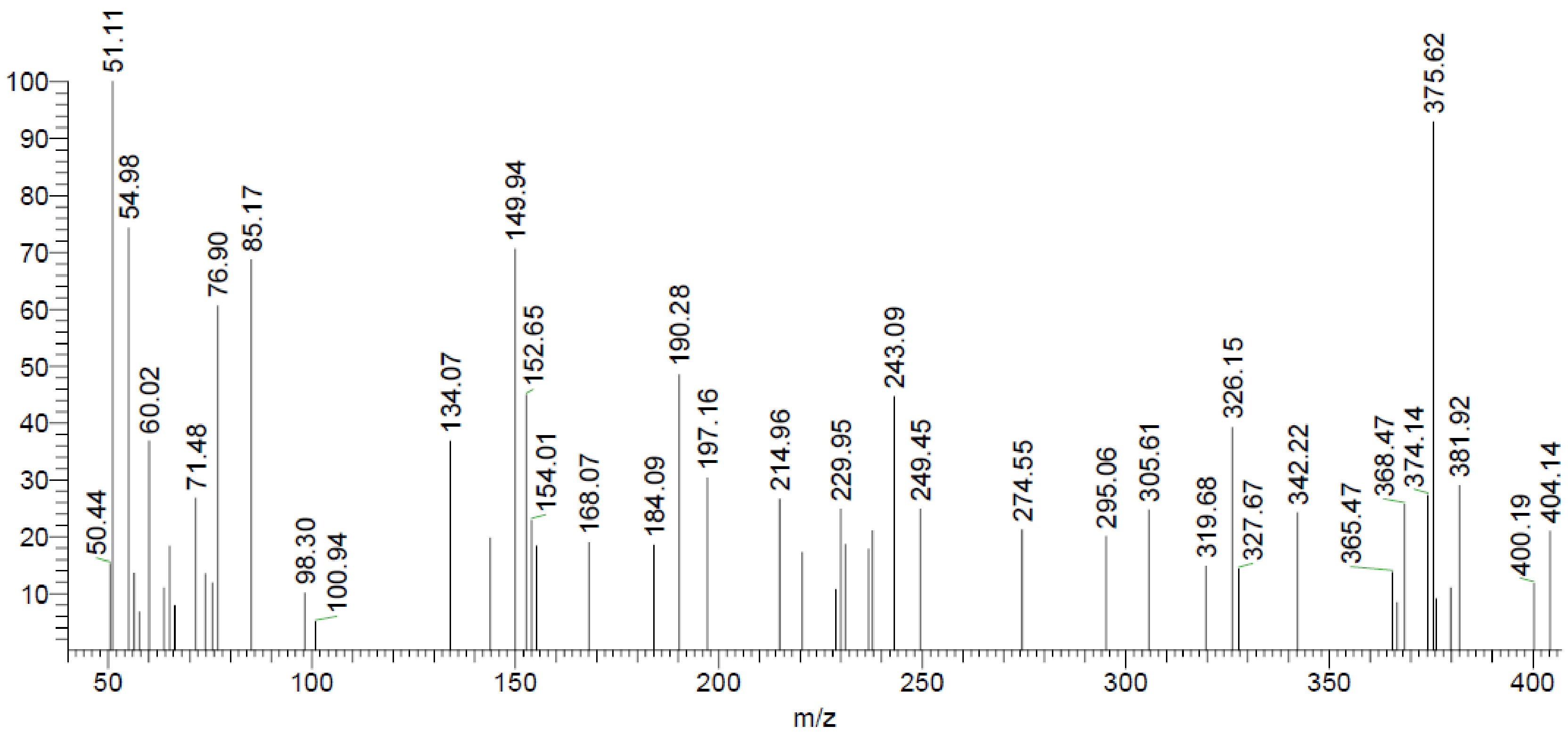

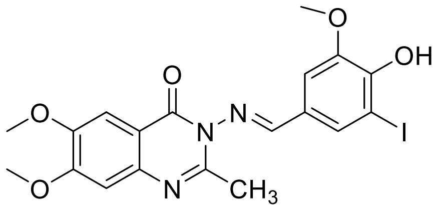
O
OH
O
N
N
I
O
O
N
CH3

6d

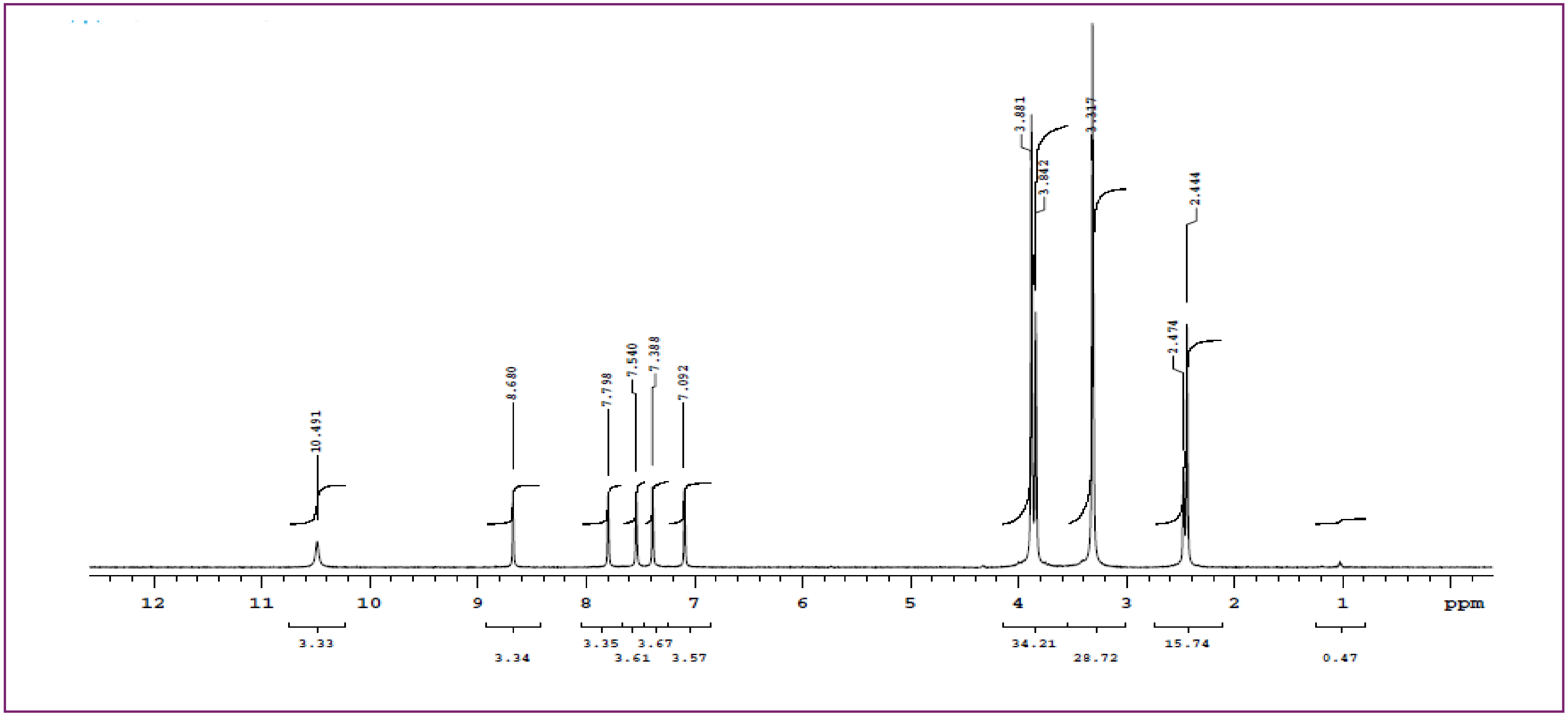
10.491
8.680
7.798
7.540
7.388
7.092
3.881
3.842
3.317
2.474
2.444
12
11
10
9
8
7
6
5
4
3
2
1
ppm
3.33
3.34
3.35
3.61
3.67
3.57
34.21
28.72
15.74
0.47

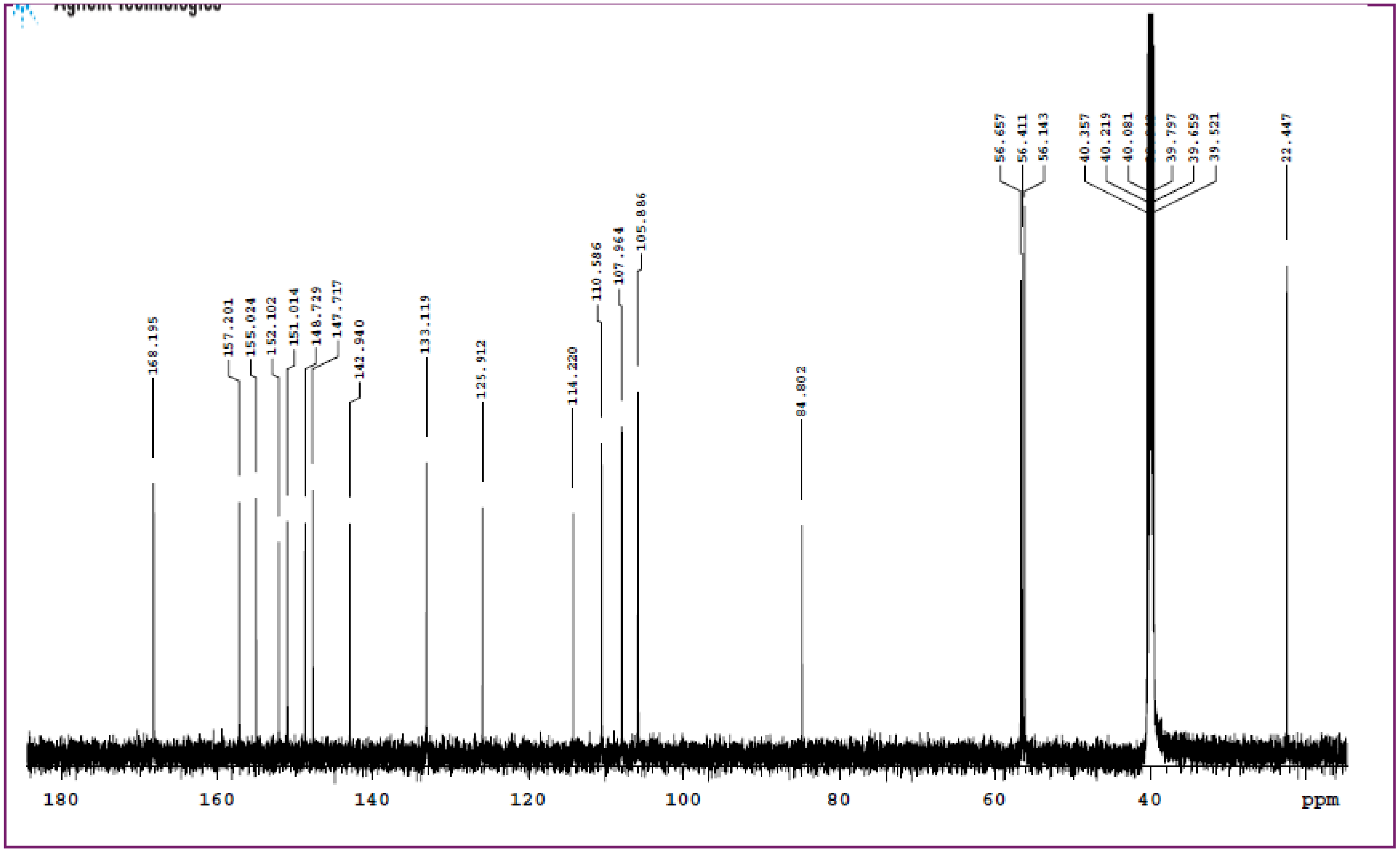
168.195
157.201
155.024
152.102
151.014
148.729
147.717
142.940
133.119
125.912
114.220
110.586
107.964
105.886
84.802
56.657
56.411
56.143
40.357
40.219
40.081
39.797
39.659
39.521
22.447
180
160
140
120
100
80
60
40
ppm

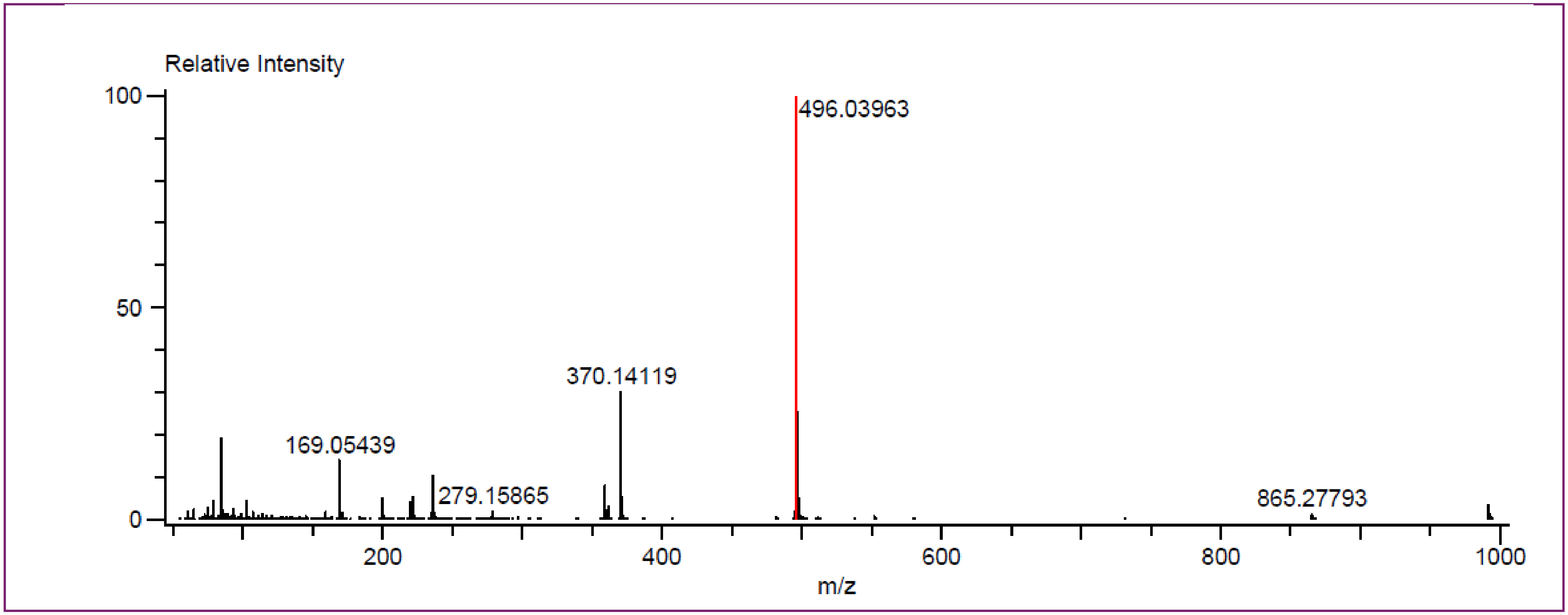
Relative Intensity
100
50
0
496.03963
370.14119
169.05439
279.15865
865.27793
200
400
600
800
1000
m/z

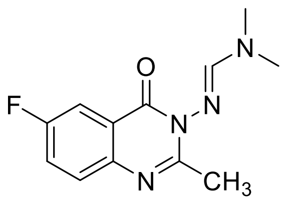

8

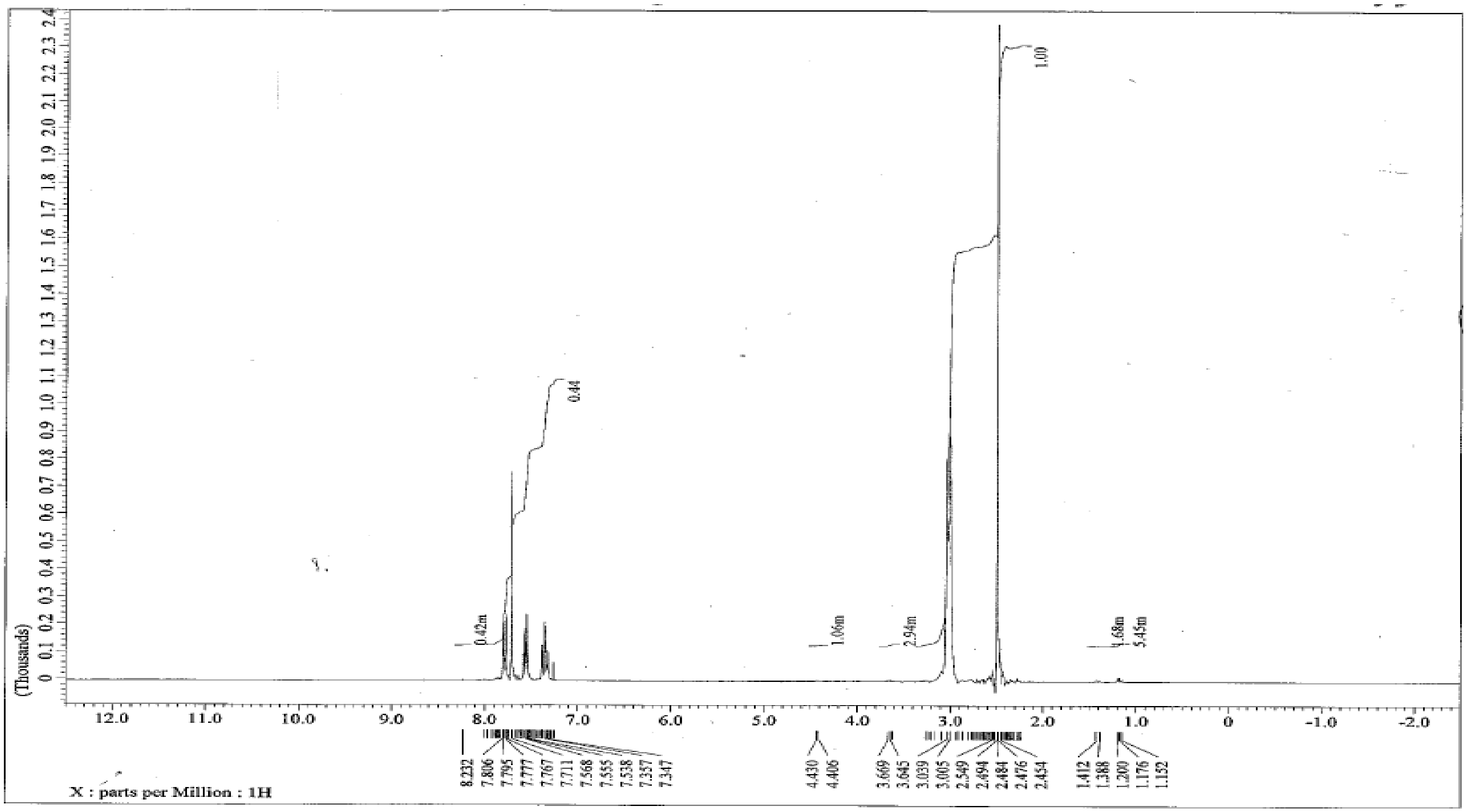
(Thousands)
X : parts per Million : 1H
0.42m
0.44
1.06m
2.94m
1.00
1.68m
5.45m
8.232
7.806
7.795
7.777
7.767
7.711
7.568
7.555
7.538
7.357
7.347
4.430
4.406
3.669
3.645
3.039
3.005
2.549
2.494
2.484
2.476
2.454
1.412
1.388
1.200
1.176
1.152

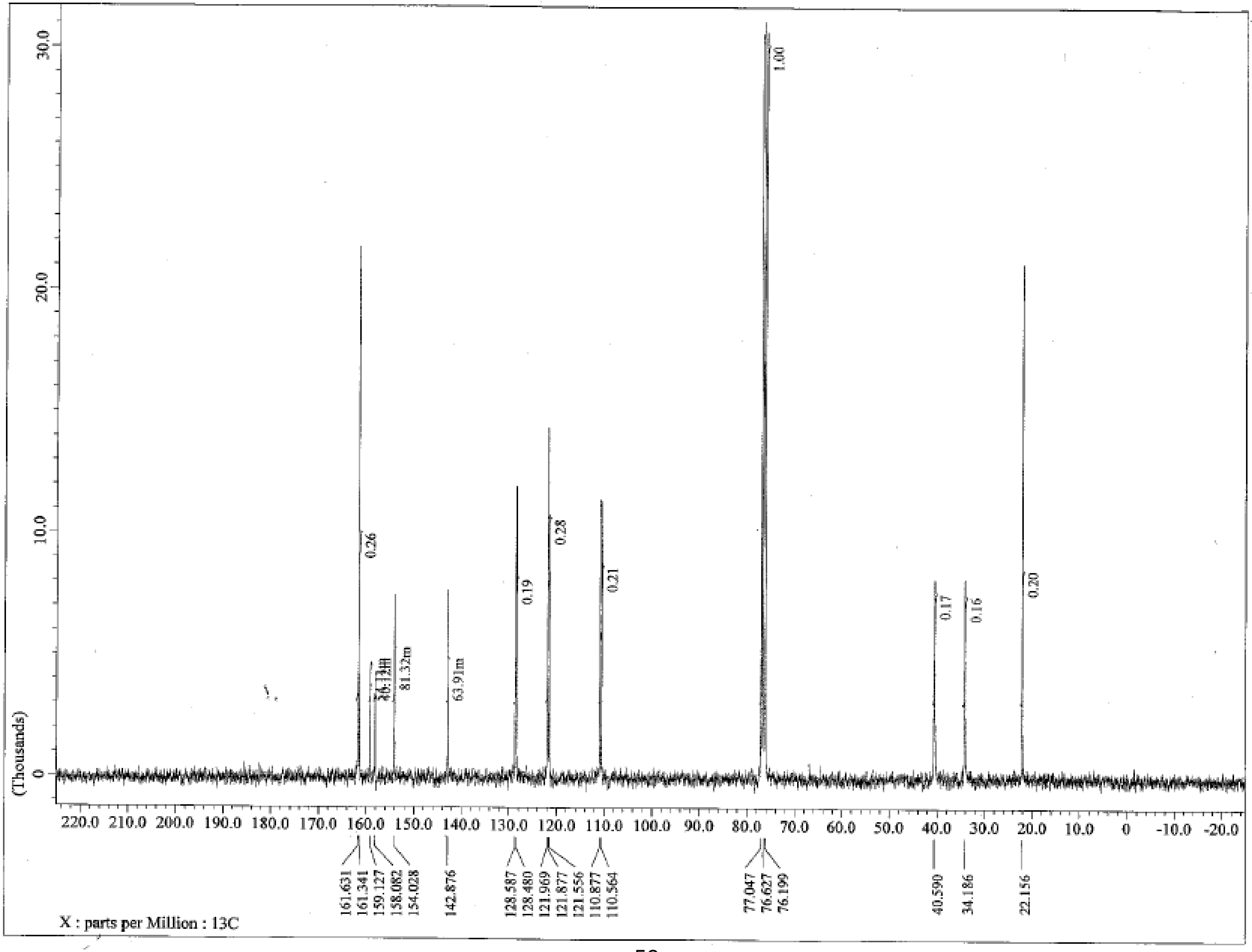

(Thousands)
30.0
20.0
10.0
0
1.00
0.26
0.28
0.19
0.21
0.17
0.16
0.20
81.32m
63.91m
220.0 210.0 200.0 190.0 180.0 170.0 160.0 150.0 140.0 130.0 120.0 110.0 100.0 90.0 80.0 70.0 60.0 50.0 40.0 30.0 20.0 10.0 0 -10.0 -20.0
161.631
161.341
159.127
158.082
154.028
142.876
128.587
128.480
121.969
121.877
121.556
110.877
110.564
77.047
76.627
76.199
40.590
34.186
22.156
X : parts per Million : 13C

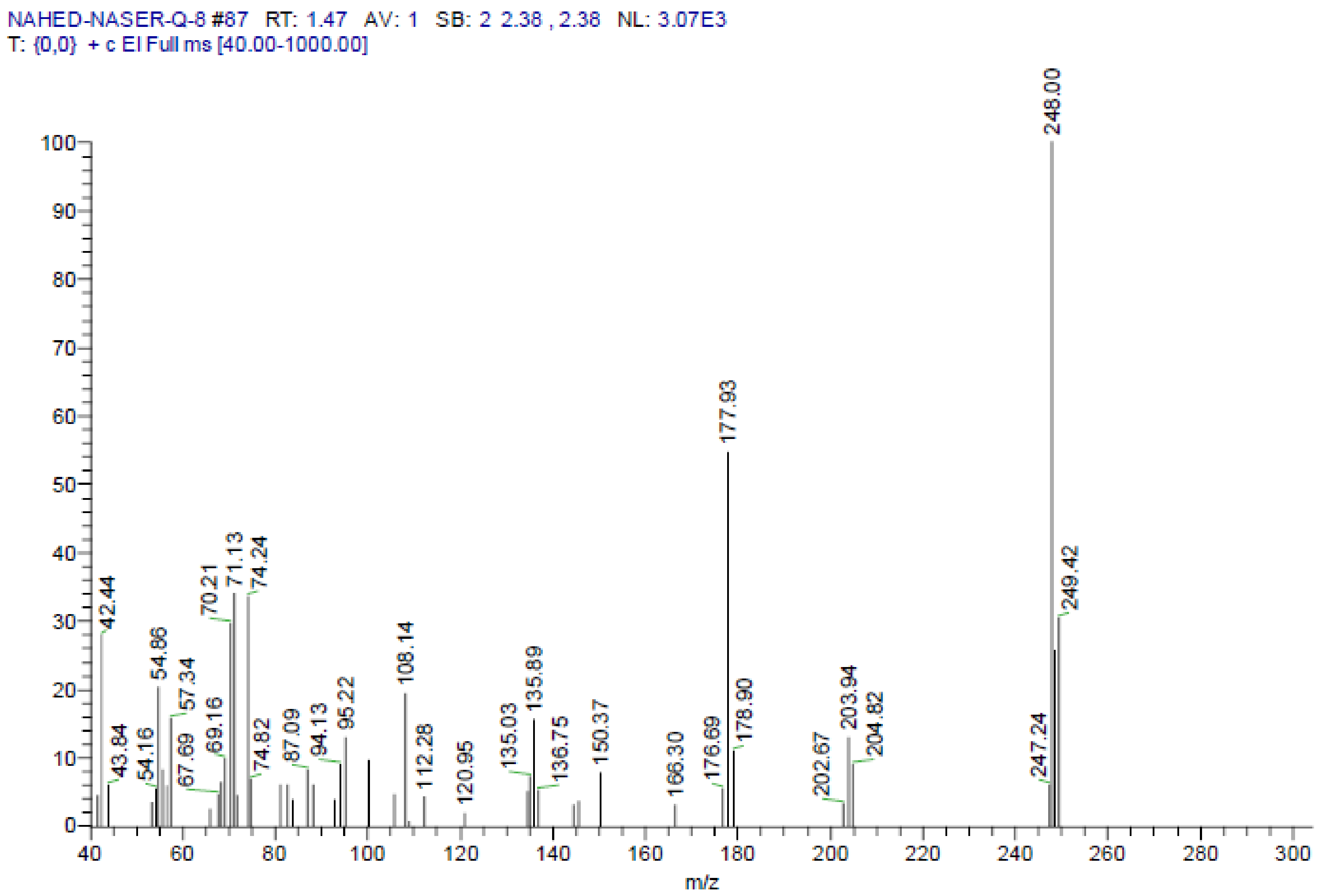
NAHED-NASER-Q-8 #87 RT: 1.47 AV: 1 SB: 2 2.38 , 2.38 NL: 3.07E3
T: {0,0} + c EI Full ms [40.00-1000.00]
248.00
177.93
249.42
42.44
43.84
54.16
54.86
57.34
67.69
69.16
70.21
71.13
74.24
74.82
87.09
94.13
95.22
108.14
112.28
120.95
135.03
135.89
136.75
150.37
166.30
176.69
178.90
202.67
203.94
204.82
247.24
m/z

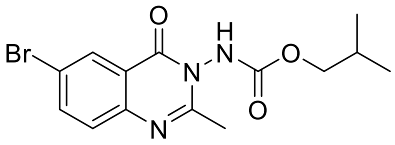
O
H
Br
N
N
O
N
O

10a

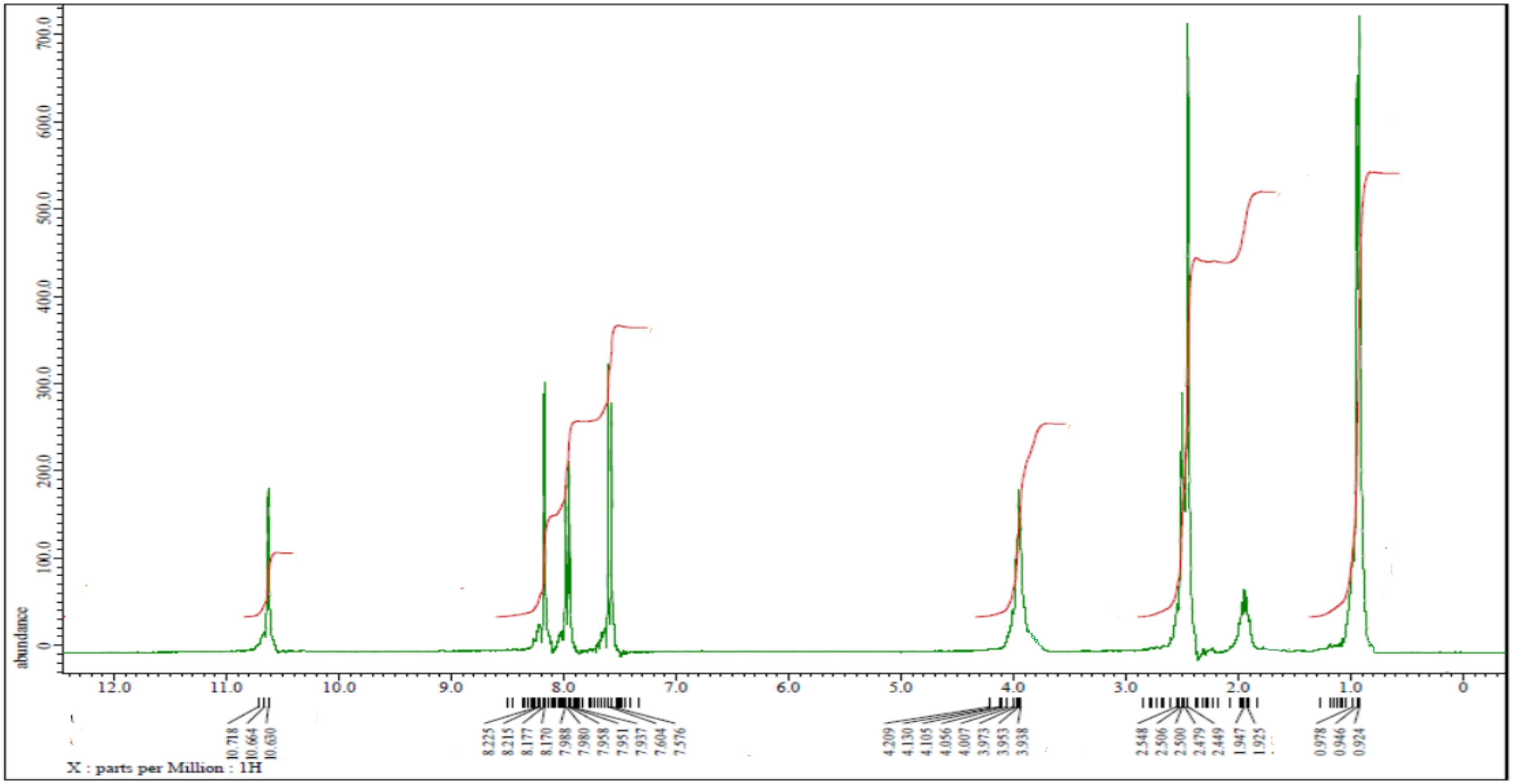
abundance
0
100.0
200.0
300.0
400.0
500.0
600.0
700.0
12.0
11.0
10.0
9.0
8.0
7.0
6.0
5.0
4.0
3.0
2.0
1.0
0
10.718
10.664
10.630
8.225
8.215
8.177
8.170
7.988
7.980
7.958
7.951
7.937
7.604
7.576
4.209
4.130
4.105
4.056
4.007
3.973
3.953
3.938
2.548
2.506
2.500
2.479
2.449
1.947
1.925
0.978
0.946
0.924
X : parts per Million : 1H

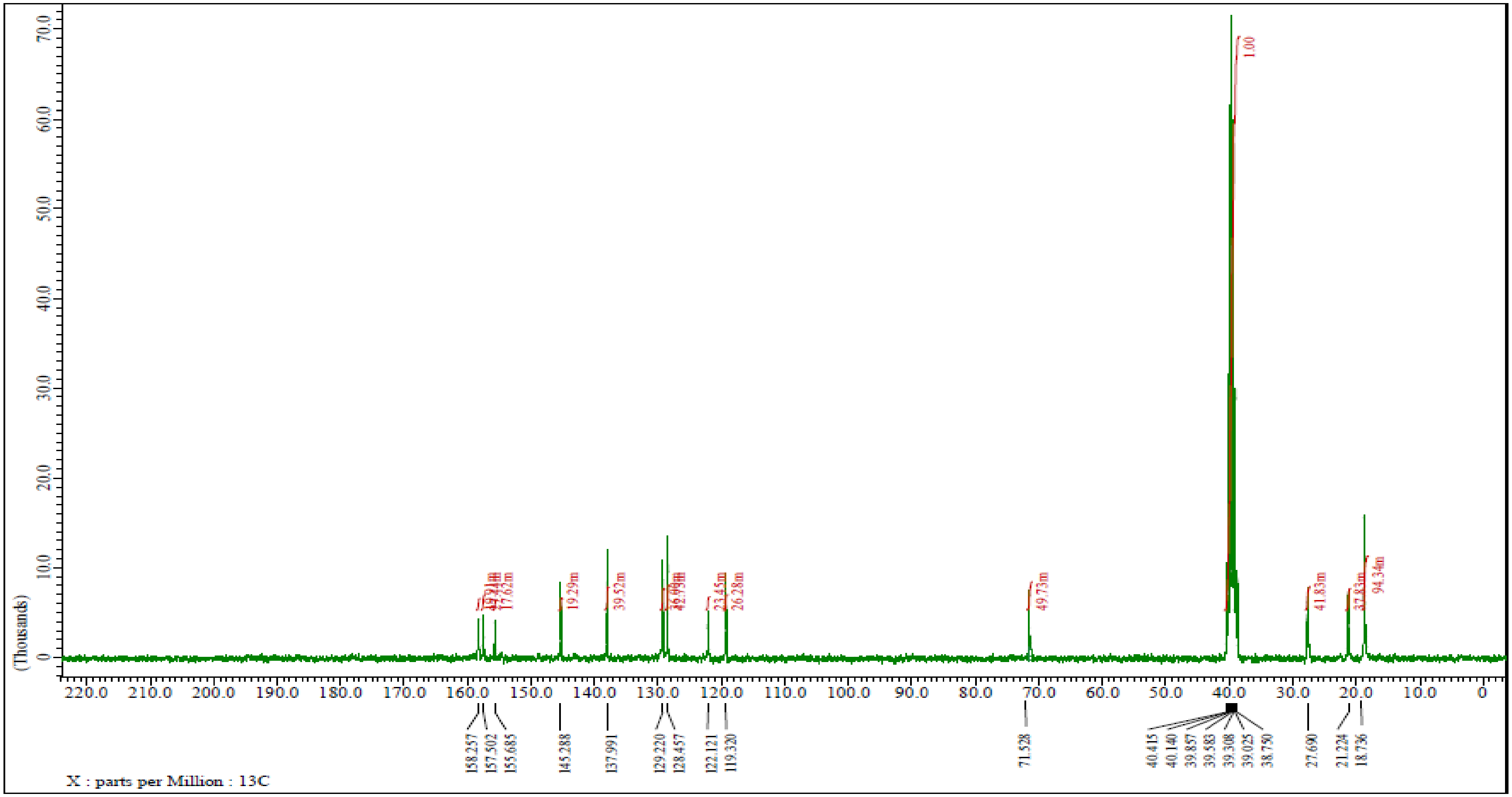
(Thousands)
X : parts per Million : 13C
158.257
157.502
155.685
145.288
137.991
129.220
128.457
122.121
119.320
71.528
40.415
40.140
39.857
39.583
39.308
39.025
38.750
27.690
21.224
18.736
1.00

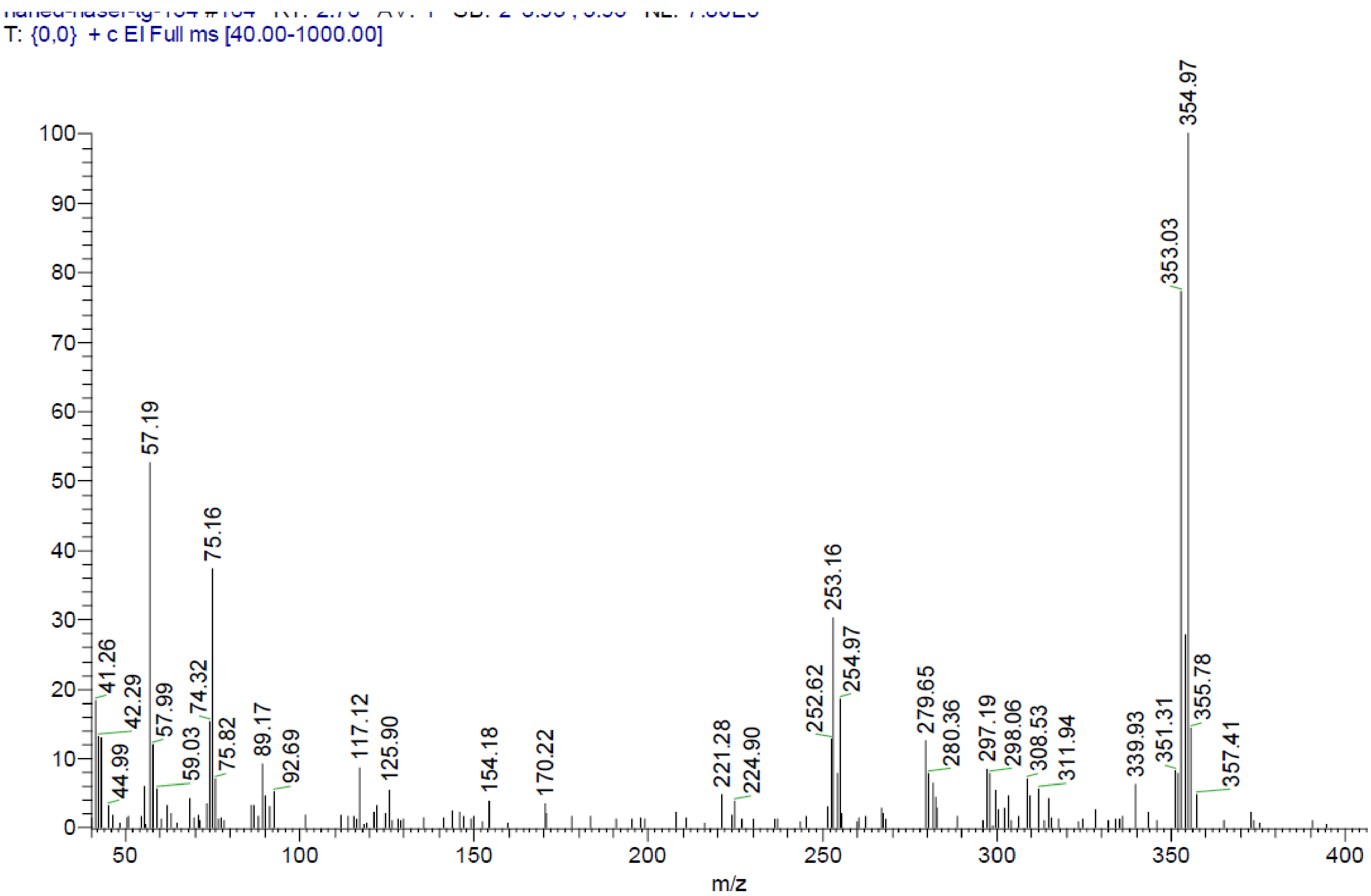
T: {0,0} + c EI Full ms [40.00-1000.00]
354.97
353.03
57.19
75.16
253.16
41.26
42.29
44.99
57.99
59.03
74.32
75.82
89.17
92.69
117.12
125.90
154.18
170.22
221.28
224.90
252.62
254.97
279.65
280.36
297.19
298.06
308.53
311.94
339.93
351.31
355.78
357.41
m/z
