# Supplementary figures and images for: Synthesis and Anticancer Activity of New Quinazolin-4(3H)-one Derivatives: Identification of a Tumor-Selective Anticancer Agent with Potential Inhibition of TGF-βRI (ALK5)
Source: Pharmaceuticals (Basel). 2026 Jun 26;19(7):996. doi: 10.3390/ph19070996 (PMC13416033; doi:10.3390/ph19070996)

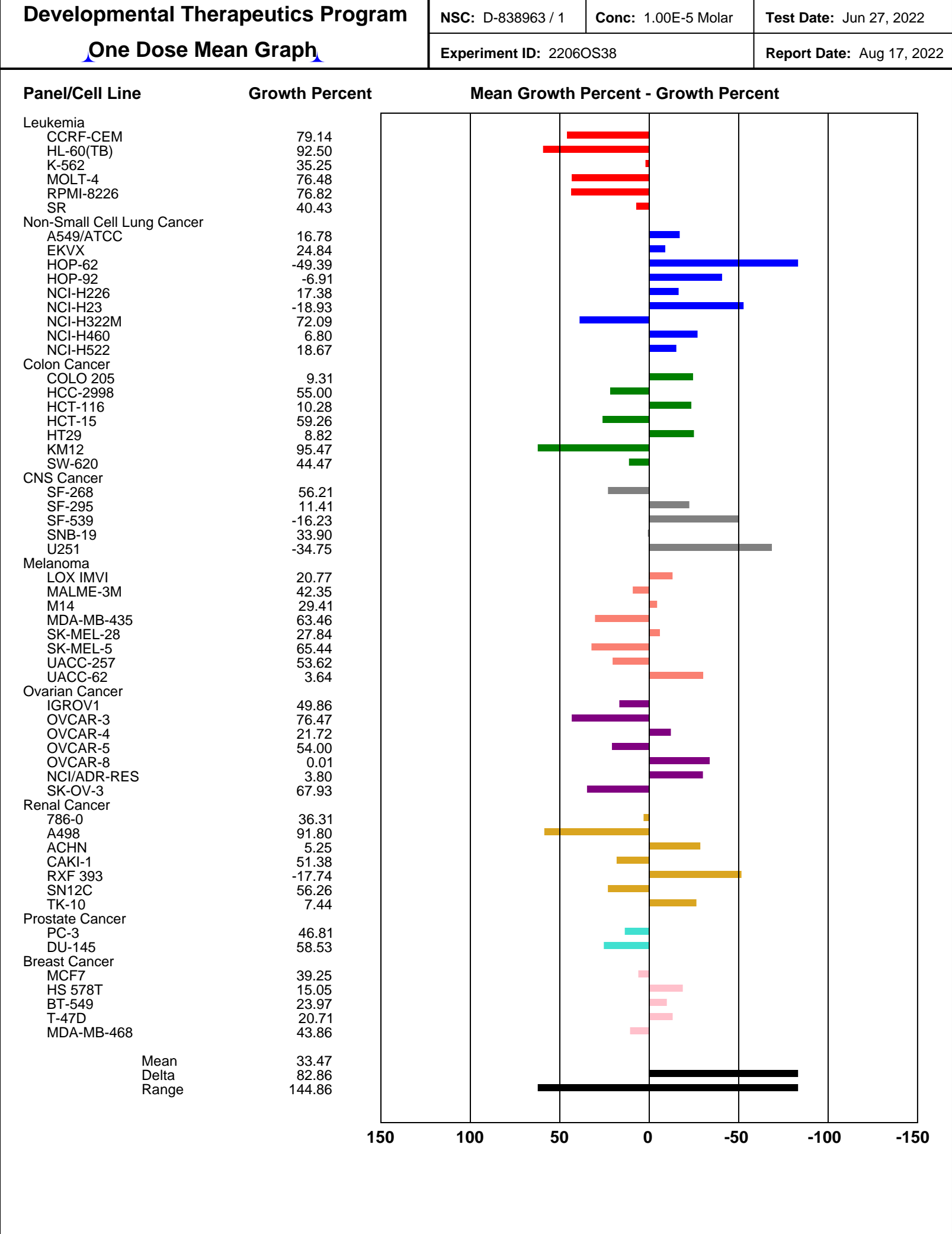

Supplement: Supplementary file 1 [file pharmaceuticals-19-00996-s001.zip › Proofread Supplementary Materials/Appendix A.pdf]
